# Site‐Specific Quadruple‐Functionalised Antibodies

**DOI:** 10.1002/anie.202417620

**Published:** 2024-11-16

**Authors:** Toby Journeaux, Michael B. Geeson, Thomas V. Murray, Monika A. Papworth, Matt Gothard, Jason G. Kettle, Aldrin V. Vasco, Gonçalo J. L. Bernardes

**Affiliations:** ^1^ Yusuf Hamied Department of Chemistry University of Cambridge Lensfield Road Cambridge CB2 1EW UK; ^2^ Biologics Engineering, Oncology R&D AstraZeneca Cambridge UK; ^3^ Analytical Sciences, Biopharmaceutical Development, BioPharmaceuticals R&D AstraZeneca Cambridge UK; ^4^ Medicinal Chemistry, Oncology R&D AstraZeneca Cambridge UK

**Keywords:** Antibodies, Antibody-drug conjugates, Multifunctionalized antibody–drug conjugates, Site-specific protein modification, Bioconjugation

## Abstract

Antibody–drug conjugates (ADCs) are a growing class of chemotherapeutic agents that have yielded striking clinical successes. However, the efficacy of ADCs often suffers from issues associated with tumor heterogeneity and resistance. To overcome these problems, a new generation of ADCs comprising a single monoclonal antibody with multiple different payloads attached, termed multi‐payload ADCs, have been developed. Here we deploy multiple orthogonal site‐specific protein modification strategies to generate highly homogeneous multi‐functionalised antibody conjugates comprising up to four different functionalities installed at four unique sites on the antibody. This work, which includes the use of a site‐specific cyclopropenone (CPO)‐based reagent, represents the first example of a homogeneous multi‐payload ADC with a payload count greater than two, and thereby facilitates the development of the next generation of ADCs.

## Graphical abstract

The incorporation of multiple payloads within the same scaffold is a growing tendency in last generation Antibody–drug conjugates (ADCs). This paper reports the first strategy for the quadruple functionalization of therapeutic antibodies using four unique functionalities installed at four unique sites on the antibody. This is possible from multiple orthogonal site‐specific protein modification strategies that result in highly homogeneous multi‐functionalised antibody with retained affinity for the target.

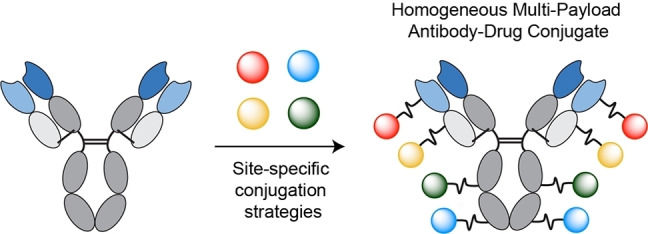

## Introduction

Antibody‐drug conjugates (ADCs) are a class of targeted therapeutics developed predominately for the treatment of cancer. Comprising a tumour targeting monoclonal antibody with a payload attached via a chemical linker, ADCs enable the targeted delivery of highly cytotoxic payloads to malignant cells, maximising on‐tumour efficacy while limiting off‐target toxicities. Critically, this highly specific delivery results in both an increased maximum tolerated dose (MTD) and lower minimum effective dose (MED) relative to standard chemotherapeutic approaches, widening the therapeutic window and allowing ADCs to emerge as a valuable class of therapeutics.[^1^, ^2^, ^3^, ^4^, ^5^]

Despite initial success, the duration of objective response or clinical benefit derived from single‐payload ADCs is often limited due to intratumor heterogeneity and the emergence of resistance mechanisms.^[6]^ As such, in an attempt to replicate the combination regimes used in small molecule cancer chemotherapies, which produce synergistic anti‐cancer effects and slow the development of drug‐resistant cell populations, the field of ADCs has recently moved towards generating antibody constructs with multiple unique payloads attached, termed multi‐payload ADCs.[^7^, ^8^, ^9^, ^10^, ^11^] Such constructs provide greater treatment effects and survival benefit in xenograft mouse models representing intratumor heterogeneity and enhanced drug resistance, when compared to co‐administration of the corresponding single drug variants.[^9^, ^12^]

To date, research into strategies for generating multi‐payload ADCs is limited and many of the current strategies require payloads to be clustered on branched heterotrifunctional linkers, thus creating localized highly hydrophobic areas. Alternatively, stochastic conjugation leads to unfavorable biophysical characteristics of the final multi‐payload ADC. Furthermore, the number of unique payloads attached to a single antibody has, to the best of our knowledge, been limited to two.[^10^, ^13^, ^14^, ^15^]

In this work, we combine multiple site‐specific conjugation methods to produce highly homogeneous, multi‐functionalised antibodies with up to four unique functionalities. We comprehensively assessed the performance of several site‐specific protein modification strategies in combination on a single IgG scaffold. We carefully evaluated aggregation, conjugation conditions (e.g. buffer, temperature and reagent equivalents) and conjugation efficiency for each method individually, and describe an optimised strategy for generating homogeneous antibody conjugates comprised of up to four unique functionalities installed at four unique, predetermined sites.

## Results and Discussion

### Screening Site‐Selective Modification Strategies

To establish a method for generating multi site‐specific ADCs, first a model monoclonal antibody—anti‐CD33—gemtuzumab, was selected. This antibody has the advantage of being well characterised and clinically relevant for ADC generation. An anti‐CD33 variant comprising a humanized IgG1 scaffold with a FcγR silencing triple mutation (L234F, L235E, P331S) in the Fc region was used. The silencing of FcγR binding is a desirable component for ADCs due to emerging data suggesting that potential dose‐limiting toxicity comes from their non‐specific uptake through binding of the Fc of the ADC to cells expressing Fc receptor (FcRs) and the activation of immune cells.[^16^, ^17^]

**Figure 1:**
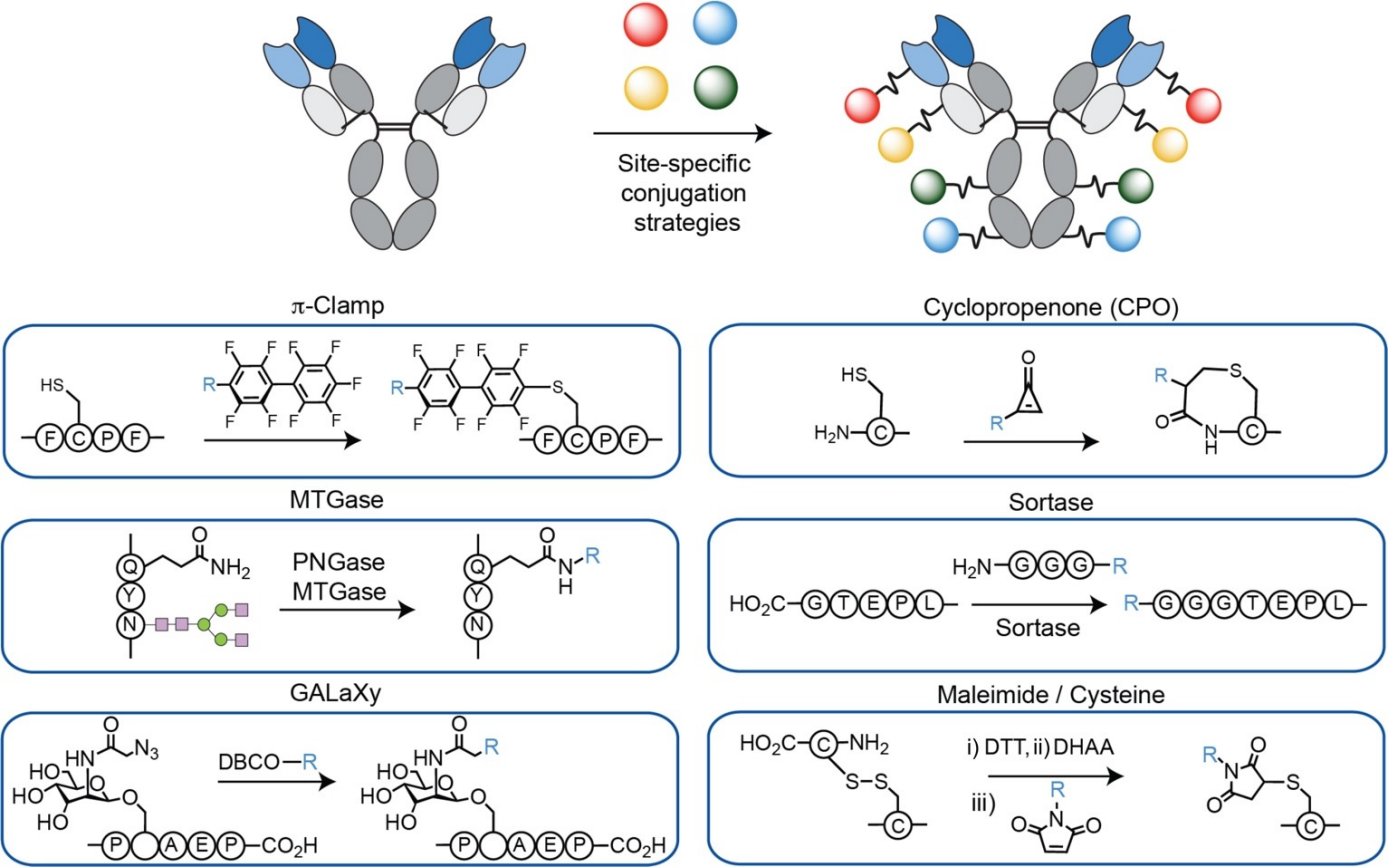
Illustration of multi site‐specific ADCs concept and the site‐specific strategies explored in this work.

In total, six site‐specific modification strategies were assessed, which include both i) chemical methods (Cyclopropenone (CPO), maleimide, Π‐Clamp and GALaXy) and ii) enzymatic methods (sortase, MTGase).[^18^, ^19^, ^20^, ^21^, ^22^] These strategies (Figure 1) were selected for their chemical orthogonality and the ability to perform conjugation at distinct sites of the antibody. Because each conjugation strategy requires antibody engineering to install a suitable reactive site, we individually tested the expression, conjugation efficiency, and the biophysical characteristics for each approach individually. For several of the strategies, it is the first time they have been applied for making ADCs. Following the optimisation and evaluation of each strategy, we selected the best performing and succeeded in preparing homogeneous, multi‐payload antibodies.

**Figure 2:**
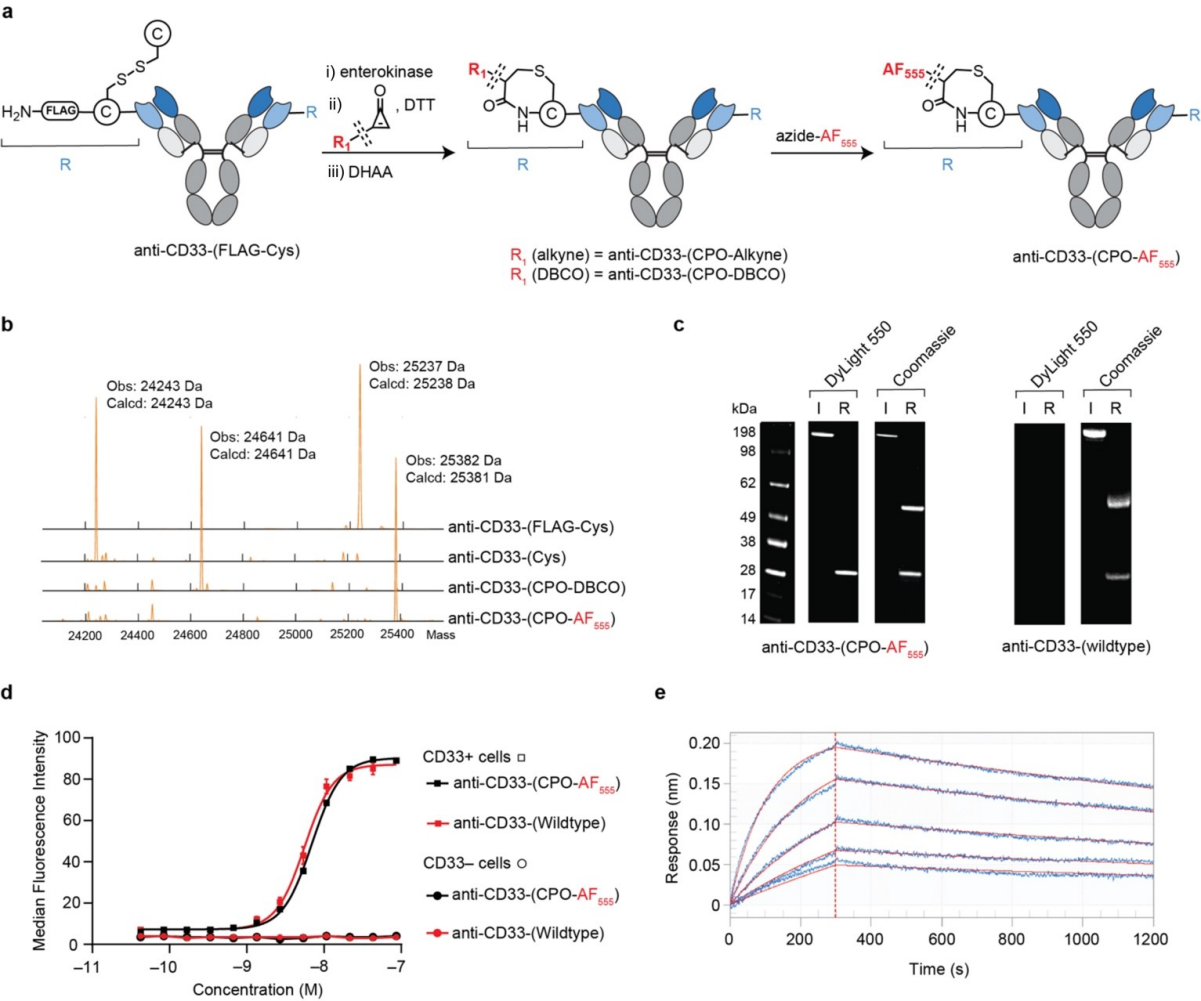
CPO‐based chemistry for N‐terminal‐specific antibody conjugation. **a**, Reaction Scheme for generating anti‐CD33‐(CPO‐AF555). **b**, Deconvoluted mass spectra obtained from LC–MS analysis of the light chain following each step of anti‐CD33‐(CPO‐DBCO) synthesis. **c**, Images of SDS‐PAGE gel with Dylight550 filter and then stained with Coomassie (I=Intact, R: reducing) of anti‐CD33‐(CPO‐AF555) and anti‐CD33‐(wildtype). **d**, Median fluorescence values obtained from titrated FACS analysis of engineered CD33+ and CD33− HEK293 cell lines. **e**, Sensorgrams obtained following BLI analysis of anti‐CD33‐(CPO‐AF555) (immobilized) with CD33 (50–3.125 nM, 2‐fold dilution series).

**Cyclopropenone (CPO)**: CPOs selectively react with N‐terminal cysteine residues in peptides and proteins containing multiple cysteine residues.^[18]^ However, such reactivity has not been assessed in full length antibodies of relevance for the preparation of therapeutic ADCs. To explore this strategy a construct comprising an N‐terminal cysteine was required. Direct incorporation of a cysteine residue at the N‐terminus can be detrimental to leader sequence cleavage, with both missed or incorrect cleavage being reported, leading to extremely low expression yields[^23^, ^24^, ^25^, ^26^] To avoid these issues, a cleavable tag was designed which could be enzymatically removed to generate a cysteine residue at the N‐terminus. Initially, an anti‐CD33 construct containing the cleavage recognition sequence of tobacco etch virus (TEV) protease (ENLYFQ↓C; arrow indicates the cleavage site) preceding a cysteine at the N‐terminus of the light chain (LC) was generated, termed anti‐CD33‐(TEV‐Cys). Anti‐CD33‐(TEV‐Cys) was isolated (73 mg/L, comparable to a wildtype control) with the expected mass and with minimal aggregation (<5 % aggregates by HP‐SEC, supplementary results Figure S7). However, the construct was not cleaved at the TEV sequence following overnight incubation with TEV protease at both 4 °C and 30 °C (Figures S9 and S10). Given that TEV protease has successfully been used to generate N‐terminal cysteines in a range of other non‐IgG proteins, this result suggests that the N‐terminus of the LC is not accessible to the TEV protease.^[27]^ In light of this result, an alternative strategy was attempted that involved the introduction of a short glycine‐serine linker preceded by a cysteine residue and a FLAG tag (DYKDDDDK↓CGGSGG) at the N‐terminus of the LC (Figure 2a). The subsequent construct, termed anti‐CD33‐(FLAG‐Cys), was isolated in moderate yields (30 mg/L) with minimal aggregation (<5 % aggregates by HP‐SEC, Figures S11 and S12). Overnight incubation with enterokinase, which recognizes and cleaves after its recognition sequence DDDDK↓, resulted in complete removal of the FLAG tag and successfully generated an antibody with an N‐terminal cysteine residue, termed anti‐CD33‐(Cys), that was employed in the CPO conjugation after buffer exchange into PBS via Ultrafiltration/Diafiltration (UF/DF). LC–MS analysis of the reduced and digested product (Figure 2b) demonstrated that the enterokinase cleavage is both highly efficient and highly specific for the tag sequence; while the LC undergoes the expected mass decrease, the mass of the heavy chain (HC) remained unchanged (Figure S14).

With a method for generating an N‐terminal cysteine construct in hand, its conjugation with CPO‐alkyne was performed. Given that CPO reactions are kinetically compatible with DTT and decysteinylation, CPO‐alkyne conjugation was performed in a single step. LC–MS analysis of the subsequent reaction mixture revealed a mass increase from 24243 to 24595 Da (expected change: 24243 to 24596), corresponding to a single CPO‐alkyne modification localized to the LC with no conjugation observed for the HC (Figure S17). The CPO‐alkyne modified product, termed anti‐CD33‐(CPO‐alkyne), was purified via size exclusion chromatography (SEC) with a protein recovery of 94 % and minimal aggregates (<5 % aggregates by HP‐SEC, Figure S19). A negative control reaction where CPO‐alkyne was incubated with anti‐CD33‐(FLAG‐Cys) under otherwise identical conditions resulted in no conjugation (Figure S13). This suggests that the site of modification is at the introduced N‐terminal cysteine residue.

Next, anti‐CD33‐(CPO‐alkyne) was used for binding and stability analysis. Given the proximity of the N‐terminus to the complementarity‐determining regions (CDRs), which mediate binding specificity, the capacity of anti‐CD33‐(CPO‐alkyne) to bind its antigen was assessed by bio‐layer interferometry (BLI) and compared to an anti‐CD33 parent control, termed anti‐CD33‐(wildtype). This analysis resulted in binding affinities (*K*_D_) of 0.82 nM and 0.87 nM for anti‐CD33‐(CPO‐alkyne) and anti‐CD33‐(wildtype) respectively (Figure S18), indicating that modification was achieved without altering antibody binding affinity to its target. Next, the stability of the CPO modification was assessed by incubation of anti‐CD33‐(CPO‐alkyne) with reduced glutathione at 37 °C for 48 hours. Subsequent LC–MS analysis indicated anti‐CD33‐(CPO‐alkyne) was stable in the presence of free thiols with less than 5 % deconjugation observed (Figure S20). This is the first assessment of the stability of the CPO conjugation and serves to support the prediction that N‐terminal cysteine modification by CPO yields a stable 7‐membered amide containing ring as previously described.^[18]^

To explore the generality of the CPO conjugation and to avoid the need to use toxic copper for further functionalisation, the conjugation was repeated with CPO‐DBCO. Akin to the results of CPO‐alkyne, a mass increase of 398 Da corresponding to a single CPO‐DBCO was observed on the light chain, with no change observed for the HC (Figure 2b). Additionally, subsequent affinity determination via BLI confirmed no significant change to target binding affinity (*K*_D_=0.77 nM) compared to anti‐CD33‐(wildtype) (*K*_D_=0.87 nM) (Figure S22). These analyses indicate that CPO‐DBCO conjugation occurs with high conversion efficiency (>90 % by LC–MS) and causes no significant change to antigen binding. The similarity of the biophysical data between the two CPO reagents validated the generality of this approach.

CPO technology has not been applied to large proteins such as antibodies before, and so the position of modification was validated. Given the N‐terminal cysteine is followed by a glycine‐serine spacer, incubation with GlySERIAS (Genovis), an enzyme that specifically cleaves glycine‐serine linkers, was used to confirm the site of modification. Separate incubation of anti‐CD33‐(Cys) and anti‐CD33‐(CPO‐DBCO) with GlySERIAS resulted in the same reduced protein mass being observed for both constructs (Figures S27 and S28). A negative control, comprising anti‐CD33‐(wildtype) treated with GlySERIAS, revealed no mass decrease (Figure S26). Taken together, these data confirmed that the location of CPO conjugation was as expected at the N‐terminus of the LC.

Further evidence as to the selectivity of this approach was provided through conjugation of the newly installed DBCO group with a fluorophore, which was achieved by incubating anti‐CD33‐(CPO‐DBCO) with azide‐AF555 (AzAF555, 5 equiv., 1 h) in PBS. LC–MS analysis of the subsequent conjugate, termed anti‐CD33‐(CPO‐AF555), revealed conjugation of a single AzAF555 molecule to the LC only (Figure 2b). This result was further validated by SDS‐PAGE, which when imaged using a 550 nm filter showed a band corresponding to the mass of the LC only (Figure 2c). In contrast, anti‐CD33‐(wildtype) showed no observable fluorescent band following identical conjugation and analytical conditions. From these results it can thus be concluded that CPO‐DBCO conjugation and subsequent AzAF555 functionalisation occurs selectively at the cysteine residue located at the N‐terminus of the LC.

Given the proximity of the modification site to the CDRs, the functionality of anti‐CD33‐(CPO‐AF555) was assessed using two binding assays: BLI and fluorescent‐activated cell sorting (FACS). FACS analysis was conducted using engineered CD33 positive (overexpression; CD33+) and CD33 negative (CD33−) HEK293 cell lines and confirmed binding to CD33 with EC50 values of 6.99±0.05 nM and 5.61±0.36 nM for anti‐CD33‐(CPO‐AF555) and anti‐CD33‐(wildtype), respectively. No binding to CD33‐ cells for either of the constructs (Figure 2d). These results suggest that CPO modification and subsequent conjugation did not impact the specificity or binding affinity of the antibody. This conclusion was further validated by BLI analysis, yielding similar *K*_D_ values for both constructs (anti‐CD33‐(wildtype): 0.87 nM, anti‐CD33‐(CPO‐AF555): 1.77 nM, Figure 2e). Overall, these data demonstrate the utility of this CPO‐based modification strategy.

#### Sortase

**Figure 3:**
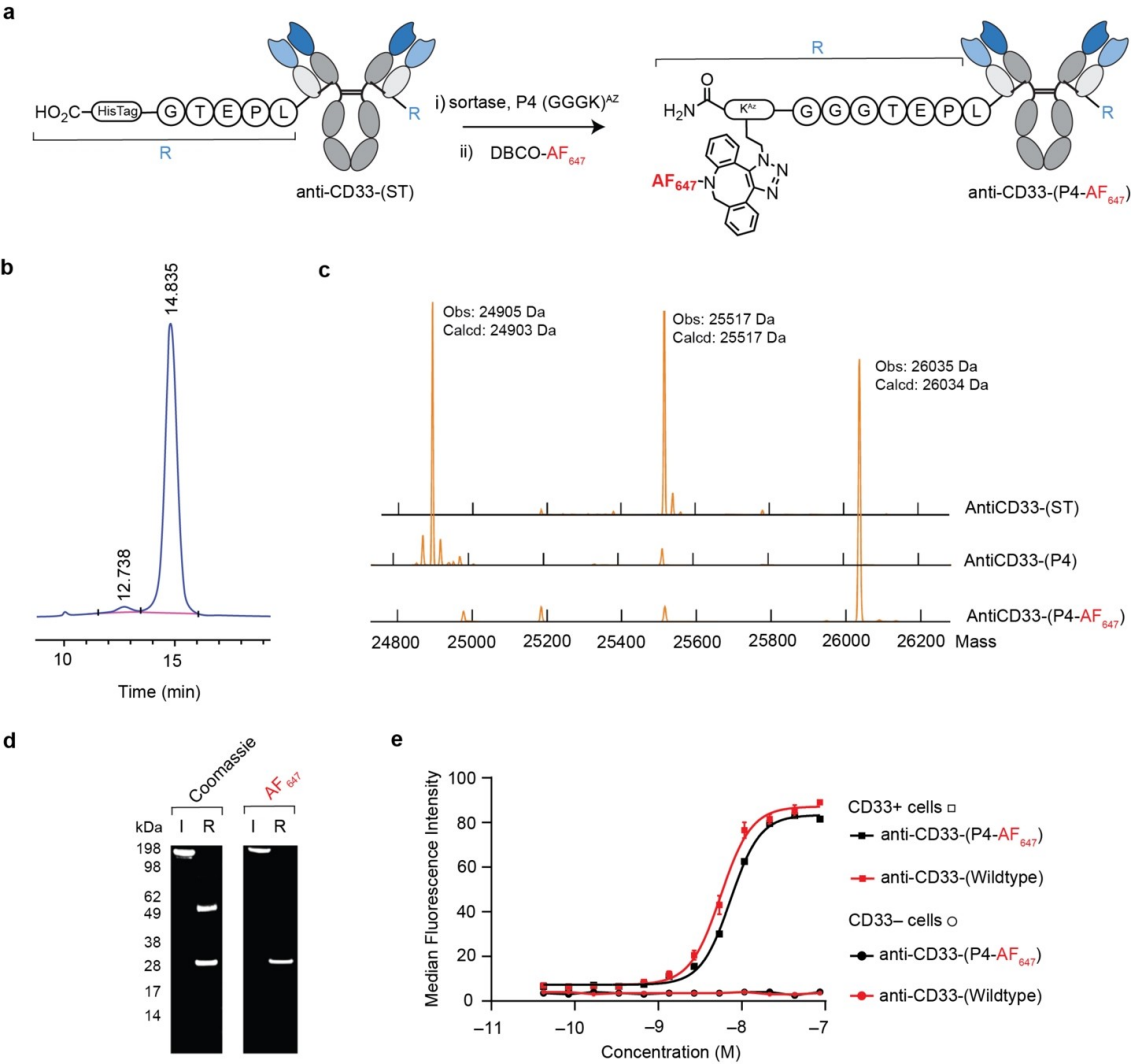
Sortase‐mediated C‐terminal‐specific antibody conjugation. **a**, Reaction scheme for generating anti‐CD33‐(P4‐AF647). **b**, UV chromatogram obtained from HP‐SEC analysis of anti‐CD33‐(ST). **c**, Deconvoluted mass spectra obtained from LC–MS analysis of the light chain following each step of anti‐CD33‐(P4‐AF647) synthesis. **d**, Images of SDS‐PAGE gel with AF647 filter and then stained with Coomassie (I=Intact, R: reducing) of anti‐CD33‐(P4‐AF647). **e**, Median fluorescence values obtained from titrated FACS analysis of engineered CD33+ and CD33− HEK293 cell lines.

Sortases, which catalyse the formation of an amide bond between the carboxyl‐group of a threonine contained within a pentapeptide sortase tag (LPXTG), abbreviated to “ST” from herein, and an amino group, have been used for site‐specific protein modification.^[22]^ Given the aim of combining multiple strategies, the ST was genetically incorporated at the C‐terminus of the LC so as to be compatible with complementary strategies used herein (Figure 3a). The subsequent construct, termed anti‐CD33‐(ST), was isolated with an expression yield of 63 mg L^−1^ with minimal aggregation (<5 % aggregates. Figure 3c), which produced the expected masses following SDS‐PAGE and LC–MS analysis (expected masses: 25517 Da, 50367 Da, SDS‐PAGE: mass bands approx. 25 kDa and 50 kDa, LC–MS: 25517 Da, 50368 Da, Figure S40).

Incubation of anti‐CD33‐(ST) with sortase and P4, a peptide comprising three glycine residues and an azido‐lysine (GGGK^Az^), resulted in P4 conjugation to the C‐terminus of the LC, which following purification via UF/DF was then functionalized using click chemistry with DBCO‐AF647. The functionalised product, termed anti‐CD33‐(P4‐AF647), was purified by SEC, resulting in a protein recovery of 88 % with minimal aggregates (<5 % by HP‐SEC, 7.1.4.4). Characterisation of anti‐CD33‐(P4‐AF647), revealed that conjugation was achieved with high conversion efficiency (>90 % by LC–MS, Figure 3c) and specificity (modification of LC only, Figure 3d). BLI and FACS assays showed that the modification was achieved without impacting the specificity or binding affinity of the antibody (*K*_D_: 0.75 nM by BLI, EC_50_: 7.3 nM by FACS, Figure 3e and Table 1). Finally, a stability study showed that the modification resisted deconjugation (<5 % by LC–MS, Figure S54) following incubation with reduced glutathione at 37 °C for 48 h. This is to be expected given sortase mediated conjugation involves the formation of a stable amide bond. Collectively these results, which are summarised in Table 1, demonstrate that sortase‐mediated conjugation strategy may be a useful technique for generating a homogeneous multi‐site multi‐payload ADC.

**Table 1:** Summary of expression, conjugation and biophysical data obtained for anti‐CD33‐(P4‐AF647).

Parameter	anti‐CD33‐ (P4‐AF647)	anti‐CD33‐(wildtype)
Normalised expression yield (mg L^−1^)	63^[a]^	96
Aggregates post expression (%)	<5^[a]^	<5
Conjugation efficiency (%)	>90^[b]^	N/A
Protein recovery post conjugation (%)	88	N/A
CD33 binding affinity (BLI) (nM)	0.75	0.87

^[a]^ Values for anti‐CD33‐(ST). ^[b]^ Value for anti‐CD33‐P4.

#### Microbial Transglutaminases

**Figure 4:**
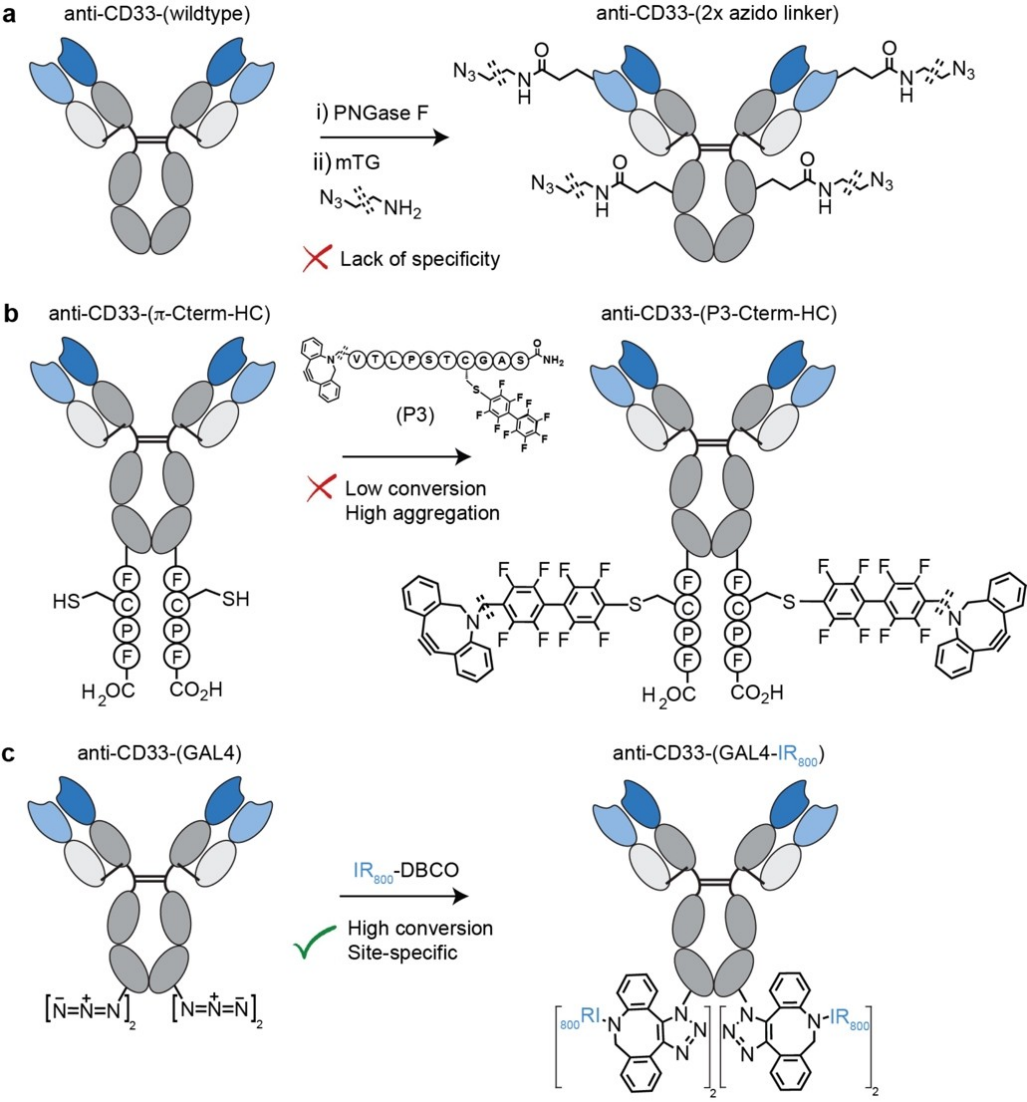
Reaction Schemes for anti‐CD33 modification using **a**, mTG‐based, **b**, π‐clamp‐based and c, GALaXy‐based approaches.

Microbial transglutaminases (mTG) can be used to selectively install payloads at position Q295 of the Fc region by catalysing the formation of a stable iso‐peptide amide bond between the γ‐carboxyamide group of glutamine residues and a nucleophilic primary amine substrate.^[28]^ Following glycan removal with PNGase F (Figure S56) and UF/DF purification, mTG mediated conjugation of an 11‐azido‐3,6,9‐trioxaundecan‐1‐amine (AzidoLinker) was performed followed by a second UF/DF purification. Interestingly, mass changes corresponding to AzidoLinker were observed for both the HC and LC (Figures 4a and S57). This was unexpected given that Q295 was the only expected site of conjugation and that the transglutaminase method of conjugation to this site is well established in the field.^[20]^ To probe this observation further, peptide mapping was performed on both anti‐CD33‐(wildtype) and anti‐CD33‐(2XAzidoLinker). This analysis revealed anti‐CD33‐(2XAzidoLinker) to be modified at position Q295‐HC and Q58‐LC in CDR2 of the LC (Figure S58). From this result, it can be concluded that specificity of mTG conjugation can extend beyond Q295 in certain circumstances, such as in mAbs with a reactive glutamine residue in the variable region. Such modification, although not previously described in an anti‐CD33 construct, has been reported in an anti‐HER2 construct at position Q3‐LC, close to CDR1 region of the LC.^[29]^ The lack of specificity for Q295 in this case resulted in our excluding this method from subsequent experiments.

#### Π‐Clamp

The π‐clamp is a tag‐based approach based on a hydrophobic recognition mechanism. The cysteine residue embedded in the π‐clamp sequence (FCPF) selectively reacts with perfluorinated biphenyl ligands in proteins that contain multiple endogenous cysteine residues.^[21]^ To assess this methodology, an anti‐CD33 antibody with a π‐clamp motif positioned at the N‐termini of the LC, termed anti‐CD33‐(π‐NTerm‐LC), was expressed. This construct was found to express poorly (normalised expression yield: 22 mg L^−1^) and with significant aggregation (42 % aggregates by HP‐SEC, Figure S31). This is potentially due to the high hydrophobicity of the inserted motif. Following purification by SEC, anti‐CD33‐(π‐NTerm‐LC) was isolated with a yield of 5 mg L^−1^ and minimal aggregates (<5 % aggregates by HP‐SEC, Figure S32). The resulting purified protein was incubated with a peptide containing both a fluorinated biphenyl ligand and DBCO moiety, termed P3 (Figure 4b). Despite a previous report indicating that the conjugation of fluorinated biphenyl ligands to the π‐clamp sequence occurs throughout the length of a peptide chain, in this study no conjugation of P3 was observed (SI, Section 7.3.3).^[21]^ The reason for the lack of π‐clamp reactivity at the N‐terminus is unclear. However, a separate study which attempted π‐clamp‐mediated conjugation at the N‐terminus of a scFv−Fc also reported a lack of reactivity, citing poor solvent accessibility as a possible cause.^[30]^ An alternative explanation is that the aromatic DBCO moiety interferes with the π–π stacking that drives the selectivity. In an attempt to optimize this system, an alternative construct with the FCPF tag at the more accessible C terminus of the HC was expressed. This construct, termed anti‐CD33‐(π‐CTerm‐HC), also suffered low expression yields and propensity for aggregation (normalised yield 17 mg L^−1^, 34 % aggregates by HP‐SEC, Figure S34). Following SEC purification and subsequent incubation with P3, conversion (20–30 %) to a mass corresponding to a single P3 conjugation was observed by LC–MS (Figure S37). From this result, we conclude that solvent accessibility limited the N‐terminus approach and not interference by DBCO. However, despite the addition of ammonium sulfate (2 M), which has been reported to increase the rate of conjugation, conversion to the modified product could not be increased beyond 20–30 %.^[31]^ HP‐SEC analysis of the reaction mixture also revealed reformation of protein aggregates (40 % aggregates, Figure S38) Given the poor expression, aggregation and the need for selectivity and full conversion, the π‐clamp strategy was not investigated any further.

#### GALaXy

Through combination of a widely applicable five amino acid tag (referred to as a GALaXy tag) with a metabolically engineered UDP‐galactose‐4‐eperimase (GALE) knock‐out cell line, on‐demand azide containing O‐glycosylation can be incorporated at a designated threonine within the GALaXy tag (Figure 4c).^[19]^ To explore the utility of this technology, an antibody bearing two concatenated GALaXy tags at the C‐terminus of each HC was expressed. Subsequent incubation with a DBCO‐bearing fluorophore in PBS followed by UF/DF purification yielded an DAR4 antibody construct of high homogeneity (>90 % conversion by LC–MS, Figure S62) and minimal aggregation (<5 % by HP‐SEC). This work, which represents the first example of GALaXy tags being deployed in a concatenated manner, demonstrates that GALaXy technology offers a facile route to producing homogeneous ADCs of tunable DAR.

### Dual‐Functionalized Site‐Specific Antibody Conjugates

**Figure 5:**
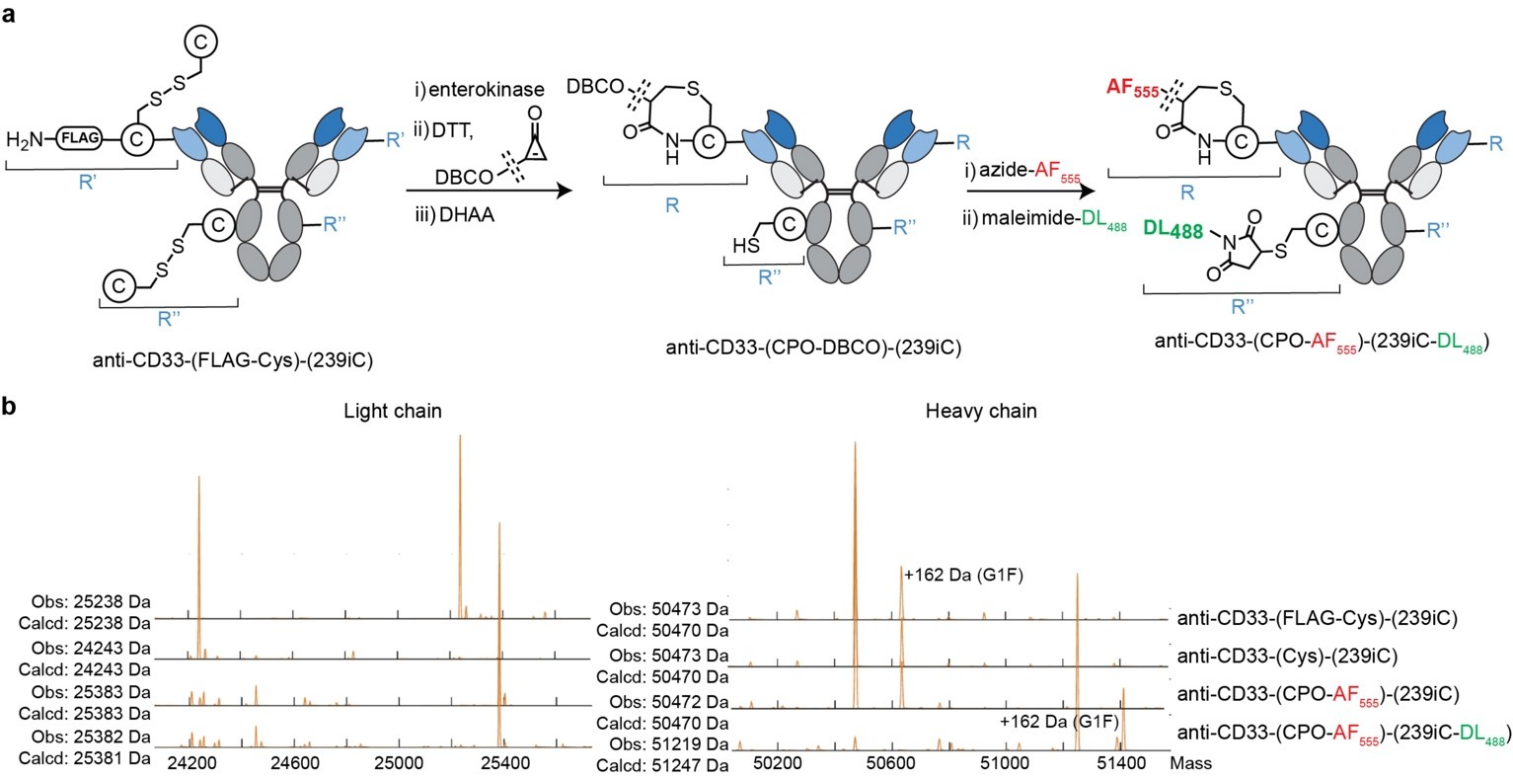
Preparation of a dual site‐specific modified antibody using CPO and maleimide strategies. **a** Reaction scheme. **b**, Deconvoluted mass spectra obtained from LC–MS analysis following each step of anti‐CD33‐(CPO‐AF555)‐(239iC‐DL488) synthesis.

Following, evaluation and optimisation of the site‐specific modification strategies on an individual basis, the most promising strategies were then combined to form dual site‐specific ADCs. Initially, the modification of an N‐terminal cysteine with a CPO reagent was coupled with an internal engineered cysteine approach (Figure 5a). To provide a comprehensive assessment of the compatibility and specificity of CPO reagents for N‐terminal cysteines we designed three constructs, each with a cysteine preceding a FLAG tag at the N‐terminus of the LC and an internal engineered cysteine for maleimide‐based conjugation at a different position in HC. The three constructs, anti‐CD33‐(FLAG‐Cys)‐(239iC), anti‐CD33‐(FLAG‐Cys)‐(T289C) and anti‐CD33‐(FLAG‐Cys)‐(A327C), were isolated in high yields (Table 2) with minimal aggregation (<5 % for all three constructs).

**Table 2:** Summary of expression, conjugation and biophysical data obtained for dual CPO‐maleimide site‐specific antibody constructs.

	anti‐CD33‐ (CPO‐AF555)‐(239iC‐DL488)	anti‐CD33‐ (CPO‐AF555)‐(T289C‐DL488)	anti‐CD33‐ (CPO‐AF555)‐(A327C‐DL488)	anti‐CD33‐(wildtype)
Normalised expression yield prior to conjugation (mg L^−1^)	50	29	42	96
Conversion to dual modified construct (LC–MS) (%)	>90	>90	80–90	N/A
Post purification recovery (%)	89	14	21	N/A
CD33 binding affinity (BLI) (nM)	2.07±0.01	1.75±0.01	1.14±0.08	0.86±0.01
CD33 binding EC50 (FACS) (nM)	5.67±0.24	7.57±0.16	6.79±0.02	5.61±0.36
FcRn binding *K*_D_ (nM)	102±13.0	62±8.7	77±6.8	46±4.3
FcgR binding *K*_D_ (nM)	N.d.	N.d.	N.d.	N.d.

N.d., Not detected.

Next, the three antibodies underwent the following reaction sequence: 1) enterokinase (16 h, 37 °C), 2) CPO‐DBCO+DTT (15/10 equiv., 25 °C, 4 h), 2) DHAA (20 equiv., 25 °C, 1 h) and 3) maleimide‐DL488+azide‐AF555 (10/5 equiv., 25 °C, 1 h) (Figure 5a). The subsequent antibody constructs were termed anti‐CD33‐(CPO‐AF555)‐(XXXC‐DL488), where XXX represents the position of cysteine incorporation. LC–MS analysis at each step showed all three constructs were converted to the expected dual‐site dual payload ADCs with high homogeneity (>90 % conversion for 239iC and T289C variants (Figures 5b, S70 and S80), 80–90 % conversion for A327C variant (Figure S90)), as summarised in Table 2. Subsequent purification by SEC revealed that anti‐CD33‐(CPO‐AF555)‐(T289C‐DL488) and anti‐CD33‐(CPO‐AF555)‐(A327C‐DL488) had significant levels of protein aggregation, leading to post‐SEC production yields of 14 % and 21 % respectively. In contrast, production of anti‐CD33‐(CPO‐AF555)‐(239iC‐DL488) resulted in minimal protein aggregation leading to a post‐SEC purification yield of 89 %. Although protein aggregation is a result of numerous factors, cysteine positioning is reported in the literature to significantly affect levels of aggregation. In this case, the differences in aggregation and thus production yield may be due to the differences in the solvent exposure of the three cysteine positions as both T289C and A327C occupy more exposed positions than 239iC. Despite the differences in yields, HP‐SEC analysis confirmed all three constructs could be isolated as intact monomeric species given an adequate purification strategy was devised (<5 % aggregates, Figures S71, S81 and S91).

BLI and FACS binding assays revealed all three antibody constructs showed no significant loss in binding affinity for CD33, when compared to the anti‐CD33‐(wildtype) (Table 2). In addition to high target‐binding specificity, the efficacy of an ADC product is also reliant on several Fc‐dependent binding mechanisms. These include neonatal Fc receptor (FcRn) and Fc gamma receptor (FcγR) binding, which regulate antibody half‐life and effector function, respectively. Given the sites of modification are proximal to the Fc binding domains, FcRn and FcγR binding assays were performed using surface plasmon resonance (SPR). This analysis yielded similar FcRn *K*_D_ values for the three dual‐fluorophore compounds and anti‐CD33‐(wildtype), suggesting the modifications had no impact on FcRn binding (Table 2). Regarding binding to FcγR, anti‐CD33‐(wildtype) displayed minimal FcγR binding and the three dual‐fluorophore constructs displayed no binding. This was to be expected given that all of the tested constructs contain a FcγR silencing triple mutation (L234F, L235E, P331S) in the Fc region.

**Figure 6:**
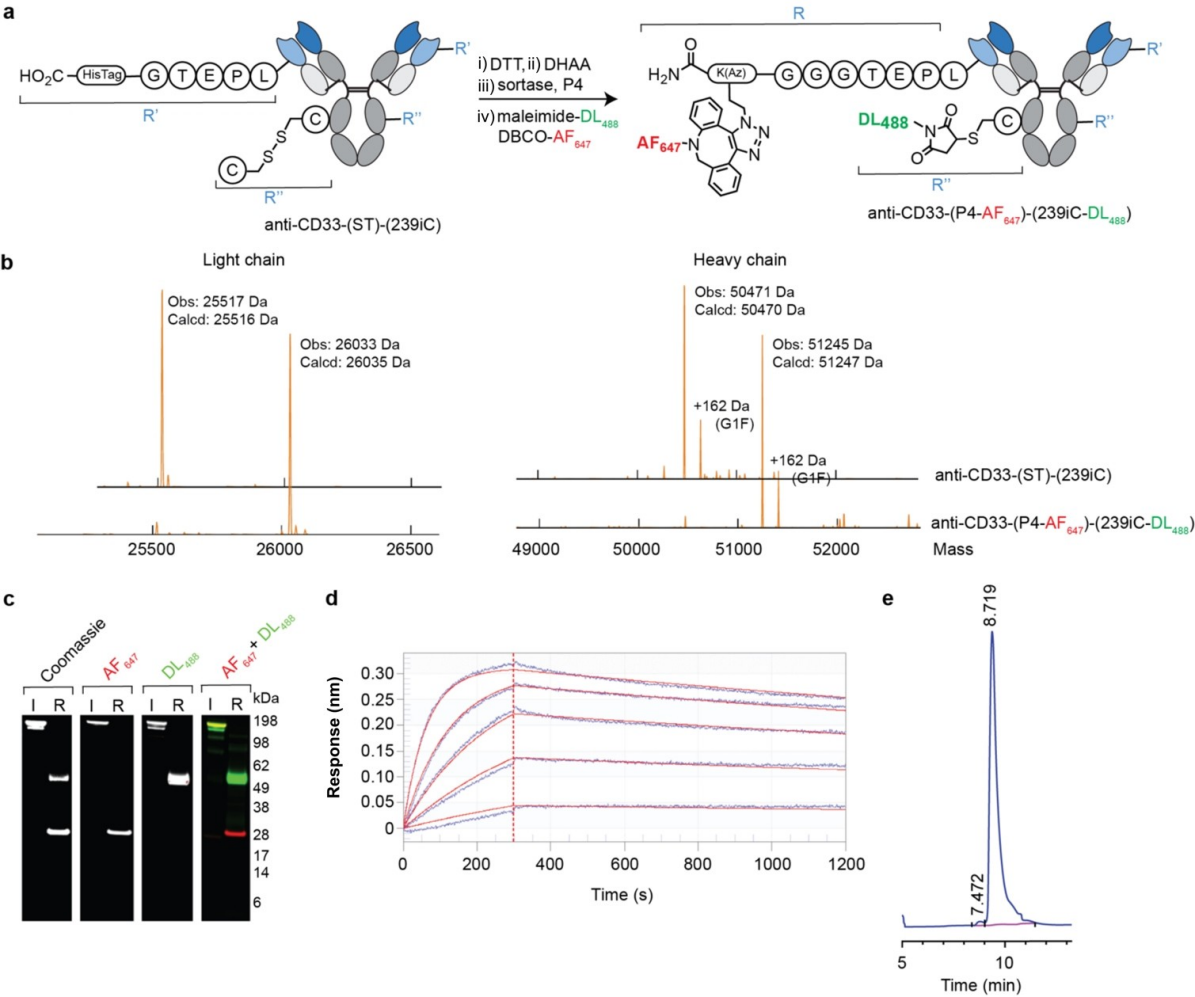
Preparation of a dual site‐specific modified antibody using maleimide and sortase approaches. **a**, Reaction scheme. **b**, Deconvoluted mass spectra obtained from LC–MS analysis following each step of anti‐CD33‐(P4‐AF647)‐(239iC‐DL488) synthesis. **c**, Images of SDS‐PAGE analysis of anti‐CD33‐(P4‐AF647)‐(239iC‐DL488) with AF647, AF488 and Coomassie filters (I=intact, R: reducing). **d**, Sensorgrams obtained following BLI analysis of anti‐CD33‐(P4‐AF647)‐(239iC‐DL488) (immobilized) with CD33 (50–3.125 nM, 2‐fold dilution series). **e**, UV chromatogram obtained from HP‐SEC analysis of anti‐CD33‐(P4‐AF647)‐(239iC‐DL488).

After establishing that position 239iC was both compatible with CPO modification and displayed the most favorable protein properties of the three positions investigated, the combination of an engineered cysteine at 239iC with the previously optimised sortase‐mediated conjugation methodology was explored (Figure 6a). A construct comprising a ST at the C‐terminus of the LC and an engineered cysteine at position 239iC in the HC, termed anti‐CD33‐(ST)‐(239iC) was expressed (normalised yield of 76 mg L^−1^) with minimal aggregation (<5 % aggregates, Figure S96i). LC–MS analysis (Figure 6b) revealed the isolated antibody had the expected mass, which was also verified qualitatively by SDS‐PAGE (Figure S96ii). Next, construct anti‐CD33‐(ST)‐(239iC) underwent the following reaction procedure: 1) DTT (10 equiv., 37 °C, 1 h); 2) DHAA (20 equiv., 25 °C, 1 h); 3) Sortase+P4 (0.05/50 equiv., 37 °C, 1 h); 4) DBCO‐AF647+maleimide‐DL488 (5/10 equiv, 25 °C, 1 h). Analysis by LC–MS confirmed progress at each step and that the expected dual‐site dual‐payload antibody construct, termed anti‐CD33‐(P4‐AF647)‐(239iC‐DL488), was produced with high homogeneity (>90 %, Figure 6b) and with the sites of modification as expected (LC: AF647, HC: DL488). The product was purified by SEC, which resulted in a final protein recovery of 79 % of starting material with less than 5 % aggregates in the isolated product (Figure 6e). SDS‐PAGE confirmed the site‐specificity of the two modifications with only the LC observable under a AF647 filter and only the HC observable under a AF488 filter (Figure 6c). The function of anti=CD33‐(P4‐AF647)‐(239iC‐DL488) was confirmed via a BLI binding assay, which demonstrated the modifications did not affect target binding affinity (*K*_D_: 0.67 nM, Figure 6d).

### Triple‐Functionalised Site‐Specific Antibody Conjugates

With two methods for generating dual‐site dual payload ADCs established, we next combined all three methods (CPO, maleimide and sortase mediated conjugations) to generate a triple‐functionalised site‐specific antibody conjugate. We designed an anti‐CD33 construct with three reactive sites: a cysteine residue proceeded by a FLAG tag at the N‐terminus of the LC; a ST at the C‐terminus of the LC; and an engineered cysteine at position 239iC in the HC. Following plasmid generation, expression and purification of this construct, termed anti‐CD33‐(FLAG‐Cys)‐(ST)‐(239iC), the following reaction protocol was performed: 1) enterokinase (16 h, 37 °C); 2) CPO‐DBCO+DTT (15/10 equiv., 25 °C, 4 h); 3) DHAA+azide‐AF555 (20/5 equiv., 25 °C, 1 h); 4) sortase+P4 (0.05/50 equiv., 37 °C, 1 h); and 5) maleimide‐DL488+DBCO‐AF647 (10/5 equiv., 25 °C, 1 h). The deconvoluted mass spectra show a single mass of the expected molecular weight, indicating the triple‐fluorophore product was generated with high homogeneity (>90 % by LC–MS, Figure S113). Akin to previous constructs, biophysical analysis confirmed the conjugation of the three fluorophores had not impacted the ability of the antibody to bind its target antigen (BLI: *K*_D_=1.49 nM, Figure S114, titrated FACS: EC_50_=5.91 nM, Figure S115).

### Quadruple‐Functionalised Site‐Specific Antibody Conjugates

**Figure 7:**
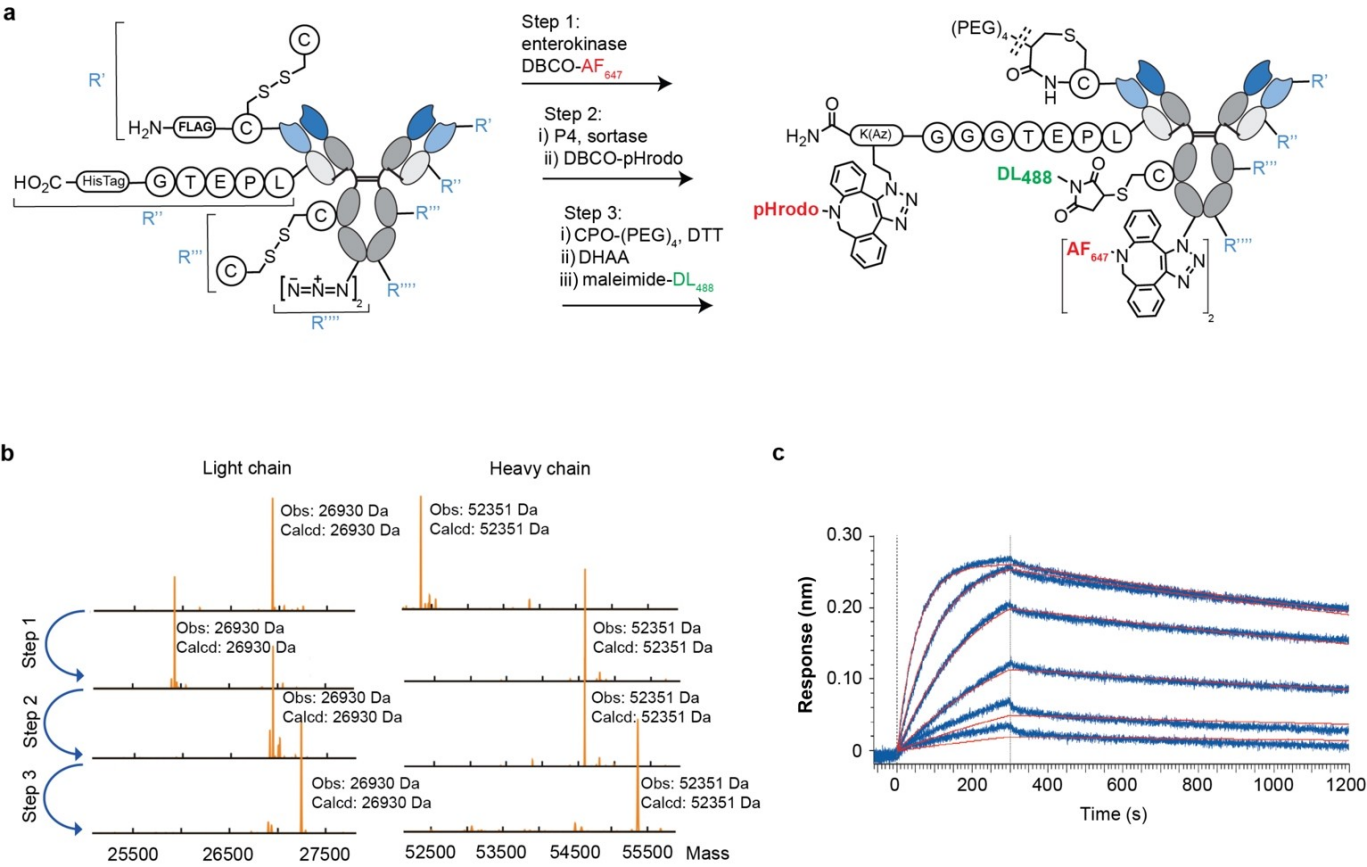
Preparation of a quadruple site‐specific modified antibody unding GALaXy, sortase, CPO and maleimide approaches. **a**, Reaction scheme. **b**, Deconvoluted mass spectra obtained from LC–MS analysis following each step of anti‐CD33‐(CPO‐PEG)‐(P4‐pHrodo)‐(239iC‐DL488)‐(GALx2 AF647) synthesis. **c**, Sensorgrams obtained following BLI analysis of anti‐CD33‐(CPO‐PEG)‐(P4‐pHrodo)‐(239iC‐DL488)‐(GALx2 AF647) (immobilized) with CD33 (50–3.125 nM, 2‐fold dilution series).

To further the current limits to preparing multi‐payload ADCs, we next attempted synthesis of a quadruple‐functionalised site‐specific antibody conjugate. To achieve this, we designed an antibody construct comprising: two GALaXy tags at the C terminus of each HC; a ST at the C terminus of the LC; a FLAG tag preceding a cysteine residue at the N terminus of the LC; and a cysteine inserted at position 239iC. This construct was expressed and subjected to a three‐step reaction procedure (Figure 7a). Analysis by LC–MS at each step revealed that the quadruple‐functionalised site‐specific antibody conjugate was produced with high homogeneity (>90 % by LCMS, Figure 7b). These reactions were carried out in a stepwise fashion with the click reaction (DBCO‐AF647) preceding maleimide conjugation. This was necessary because the latter requires a reduction step, which if performed first, would cause the azide moiety in GalNAz to be reduced to an amine which is non‐functional for subsequent conjugation reactions. The final construct contained the following functionalities: 2×PEG (LC N‐terminus); 2×AF488 (HC 239iC); 2×pHrodo (LC C‐terminus); and 4×AF647 (HC C‐terminus). Although achieved using hydrophilic payloads, successful isolation of an antibody bearing four unique payloads with high homogeneity represents an exciting step in the field of multi‐payload ADCs. This synthesis was further complimented with BLI analysis which confirmed the quadruple‐site quadruple‐payload antibody conjugate retained affinity for the CD33 antigen (*K*_D_ =0.84 nM, Figure 7c).

## Conclusions

We performed a comprehensive assessment of the capacity of several site‐specific protein modification strategies to be used in combination on a single IgG scaffold to form multi‐functionalised site‐specific antibody conjugates. Initially, six strategies that showed promise for combinatory use were assessed individually. The most robust methods were combined to generate homogeneous antibody conjugates comprising multiple unique modifications. The combination of CPO‐based N‐terminal cysteine modification with maleimide‐based modification of internal cysteine residues resulted in dual‐fluorophore antibody conjugates being generated with high homogeneity. Interestingly, the position in which the internal cysteine was engineered was found to significantly influence both the antibody expression yield and the production yield of the dual‐functionalised antibody, with the less exposed position of 239iC providing higher yields relative to other cysteine variants (A327C, T289C). These two conjugation strategies were then combined with optimised sortase and GALaXy‐mediated conjugation methods to generate an antibody construct with four unique modifications selectively installed at four distinct sites. Importantly, this product retained its antigen binding properties. This provides the first example of a homogeneous multi‐payload antibody conjugate with an order greater than two. Because the combination of different payloads into a single antibody represents the forefront of next‐generation of ADCs, our multi site‐specific antibody conjugation approach will become an useful tool to achieve ADCs with improved efficacy and capable to address cancer resistance.^[11]^ Future work in this area should aim to identify a suite of optimized payload combinations that, when conjugated to a single antibody construct, not only reduce the development of resistance but also exhibit an improved therapeutic index relative to traditional ADC regimes. Furthermore, we envision applications of site‐specific tetra labelling through combination of different payloads with imaging agents to allow monitoring biodistribution and/or intracellular localisation of ADCs (e.g. by using fluorescent and pH sensitive dyes). Other potential applications include the conjugation of additional functional or targeting domains such as peptides, degraders, VHHs or Fabs for use as therapeutic or screening tools.

## Supporting Information

Chemical, bioconjugation, molecular biology and protein expression methods. Analytical and biophysical characterization of conjugates.

## Conflict of Interests

T.V.M., M.A.P., M.G and J.G.K. are employees of AstraZeneca and have stock ownership and/or stock options or interests in the company.

1

## Supporting information

As a service to our authors and readers, this journal provides supporting information supplied by the authors. Such materials are peer reviewed and may be re‐organized for online delivery, but are not copy‐edited or typeset. Technical support issues arising from supporting information (other than missing files) should be addressed to the authors.

Supporting Information

## Data Availability

The data that support the findings of this study are available in the supplementary material of this article.
