# Supplementary material for: Site‐Specific Quadruple‐Functionalised Antibodies
Source: Angew Chem Int Ed Engl. 2024 Nov 16;64(5):e202417620. doi: 10.1002/anie.202417620 (PMC11773117; doi:10.1002/anie.202417620)

Angewandte Chemie
Eine Zeitschrift der Gesellschaft Deutscher Chemiker

Supporting Information

Site-Specific Quadruple-Functionalised Antibodies

*T. Journeaux, M. B. Geeson, T. V. Murray, M. A. Papworth, M. Gothard, J. G. Kettle, A. V. Vasco, G. J. L. Bernardes**

Supporting Information

S1 Chemical synthesis 5

S2 Molecular biology 14

S3 Protein expression and purification 15

S4 Tissue culture 17

S5 Characterisation and stability methods 18

S6 Bioconjugation methods 24

S7 Antibody conjugate characterisation 26

7.1 AntiCD33-(Wildtype) 26

7.2 CPO 28

7.2.1 AntiCD33-(TEV-Cys) 28

7.2.2 AntiCD33-(TEV-Cys) - Attempted TEV cleavage 30

7.2.3 AntiCD33-(FLAG-Cys) 32

7.2.4 AntiCD33-(FLAG-Cys) - Negative control 34

7.2.5 AntiCD33-(Cys) 35

7.2.6 AntiCD33-(CPO-Alkyne) 37

7.2.7 AntiCD33-(CPO-DBCO) 41

7.2.8 AntiCD33-(CPO-AF555) 43

7.2.9 GlySERIAS digestion 46

7.3 π-Clamp 49

7.3.1 AntiCD33-(πClampNTerm-LC) 50

7.3.2 AntiCD33-(πClampCTerm-HC) 52

7.3.3 AntiCD33-(P3NTerm-LC) - Attempted 54

7.3.4 AntiCD33-(P3CTerm-HC) 55

7.4 Sortase 57

7.4.1 AntiCD33-(ST) 58

7.4.2 Optimisation of sortase-mediated conjugation of P4 to antiCD33-(ST) 60

7.4.3 AntiCD33-(P4) 66

7.4.4 AntiCD33-(P4-AF647) 67

7.5 Transglutaminase 71

7.5.1 AntiCD33-(wildtype-deglycosylated) 72

7.5.2 AntiCD33-(2XAzidoLinker) 73

7.5.3 AntiCD33-(2XAzidoLinker-AF647) 75

7.6 GALaXy...76

7.6.1 AntiCD33-(GAL4) 77

7.6.2 AntiCD33-(GAL4-IR800) 78

7.7 Dual modification: CPO + maleimide (239iC) 79

7.7.1 AntiCD33-(FLAG-Cys)-(239iC) 80

7.7.2 AntiCD33-(Cys)-(239iC)...82

7.7.3 AntiCD33-(CPO-DBCO)-(239iC) 84

7.7.4 AntiCD33-(AF555AzDBCO-CPONTerm-LC)-(239iC-HC)..85

7.7.5 AntiCD33-(CPO-AF555)-(239iC-DL488) 86

7.8 Dual modification: CPO and maleimide (T289C) 90

7.8.1 AntiCD33-(FLAG-Cys)-(T289C) 90

7.8.2 AntiCD33-(Cys)-(T289C)..92

7.8.3 AntiCD33-(CPO-DBCO)-(T289C) 94

7.8.4 AntiCD33-(CPO-AF555)-(T289C) 95

7.8.5 AntiCD33-(CPO-AF555)-(T289C-DL488) 96

7.9 Dual modification: CPO and maleimide (A327C) 100

7.9.1 AntiCD33-(FLAG-Cys)-(A327C) 100

7.9.2 AntiCD33-(Cys)-(A327C) 102

7.9.3 AntiCD33-(CPO-DBCO)-(A327C)..104

7.9.4 AntiCD33-(CPO-AF555)-(A327C) 105

7.9.5 AntiCD33-(CPO-AF555)-(A327C-DL488)...106

7.10 Dual modification: Sortase and maleimide (239iC) 110

7.10.1 AntiCD33-(ST)-(239iC)-1 111

7.10.2 AntiCD33-(ST)-(239iC)-3 113

7.10.3 AntiCD33-(P4)-(239iC) 115

7.10.4 AntiCD33-(P4)-(239iC-DL488) 116

7.10.5 AntiCD33-(P4-AF647)-(239iC-DL488) 117

7.11 Triple modification: CPO, maleimide (239iC) and sortase 122

7.11.1 AntiCD33-(FLAG-Cys)-(ST)-(239iC) 123

7.11.2 AntiCD33-(Cys)-(ST)-(239iC) 125

7.11.3 AntiCD33-(CPO-DBCO)-(ST)-(239iC) 126

7.11.4 AntiCD33-(CPO-AF555)-(ST)-(239iC) 127

7.11.5 AntiCD33-(CPO-AF555)-(P4)-(239iC) 128

7.11.6 AntiCD33-(CPO-AF555)-(P4-AF647)-(239iC-DL488) 129

7.12 Quadruple modification: CPO, maleimide(239iC), sortase and GALaXy... 132

7.12.1 AntiCD33-(FLAG-Cys)-(ST)-(239iC)-(GAL4) 133

7.12.2 AntiCD33-(Cys)-(ST)-(239iC)-(GAL4-AF647) 134

7.12.3 AntiCD33-(Cys)-(P4)-(239iC)-(GAL4-AF647) 135

7.12.4 AntiCD33-(Cys)-(P4-pHrodo)-(239iC)-(GAL4-AF647) 136

7.12.5 AntiCD33-(CPO-PEG)-(P4-pHrodo)-(239iC-DL488)-(GAL4-AF647) 137

S8 NMR 140

Appendix 1 146

Appendix 2 152

S1 Chemical synthesis

Functionalised fluorescent probes were conjugated to various antibody constructs. The probes were purchased from the suppliers described below, however in some cases the structures or expected mass are not disclosed. To ascertain the expected mass increase upon conjugation of such probes to an antibody, LC-MS analysis of the commercial probes was performed.

Name	Supplier	Catalog Number	Reference	Mass (Da)
DyLight™ 488 Maleimide	Thermo Scientific™	46602	DL488Mal	777
Alexa Fluor™ 555 Azide, Triethylammonium Salt	Thermo Scientific™	A20012	AF555Az	740
AZDye™ 647 DBCO	ClickChemistryTools	1302	DBCODye647	1117
AFDye™ 647 DBCO	ClickChemistryTools	1302	DBCODye647	1131
pHrodo™ iFL Red	Invitrogen™	C20034	DBCOpHrodo	1612
IRDye 800CW DBCO	LI-COR	929-55000	IR800	1258

Perfluorophenyl hex-5-ynoate, (2)

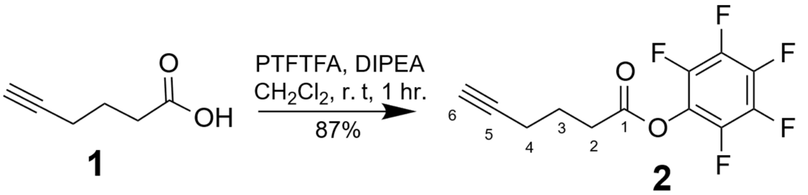

1 **2**

5-hexynoic acid **1** (0.465 g, 4.15 mmol, 1 equiv.) was dissolved in dry CH_2Cl_2 (15 mL). After cooling to 0°C, DIPEA (1.204 g, 9.338 mmol, 2.25 equiv.) and pentafluorophenyl trifluoroacetate (2.267 g, 8.097 mmol, 1.95 equiv.) were added dropwise. The subsequent solution was stirred at room temperature. After 2 hours, the reaction mixture was concentrated under vacuum and the crude product purified by CC (PE/Et_2O 98:2) to yield product **2** (1.00 g, 87%) as a colourless liquid.

TLC: (PE/Et_2O 4:1) R_f= 0.80.

^{1}H NMR: ($CDCl_3$, 400 MHz): δ 2.84 (t, J = 7.4 Hz, 2H, H-C(2)), 2.36 (td, J = 6.9, 2.7 Hz, 2H, H-C(4)), 2.05 – 1.94 (m, 3H, H-C(6), H-C(3)).

13C NMR: (CDCl3, 100 MHz): δ 169.15 (C(1)), 142.67–142.12, 141.01–140.48, 140.13–139.69, 139.45138.92, 138.53–137.94, 137.11–136.27, 125.44–124.70 (m, (C-arom)), 82.57 (C(5)), 69.90 (C(6)), 32.02 (C(2)), 23.46 (C(3)), 17.75 (C(4)).

^{19}F NMR: ($CDCl_3$, 376 MHz): δ -153.72 – -153.88 (m), -159.01 (t, J = 21.6 Hz), -163.24 – -163.44 (m).

ESI⁺-HRMS: calculated for C12H8F5O2 ([M+H]⁺) 279.0444, found 279.0435.

Perfluorophenyl 4-(3'-oxocycloprop-1'-en-1'-yl)butanoate, (3)

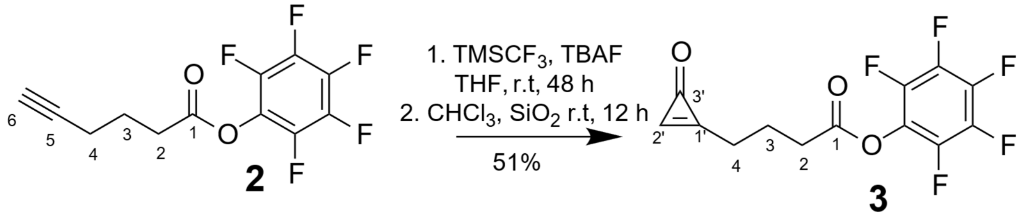

TBAF (0.58 g, 2.2 mmol) was added a pressure tube and dried under vaccum for 4 hours. Next, anhydrous THF (3.0 mL), trifluoromethyltrimethylsilane (300 µL, 2.0 mmol) and perfluorophenyl hex-5-ynoate **2** (278 mg, 1.0 mmol) were added. After 24 hrs of stirring at room temperature, further trifluoromethyltrimethylsilane (150 µL, 1.0 mmol) was added, and the reaction mixture was left stirring for another 24 h. The reaction mixture was then diluted with H_2O (20 mL) and extracted with Et_2O (3 x 20 mL). The combined organic layers with crude difluorocyclopropene were dried over $MgSO_4$, filtered, concentrated, adsorbed on silica in a solution of $CHCl_3$, and left overnight at room temperature to generate the corresponding cyclopropenone. The product was then concentrated under vacuum and purified by CC (PE/EtOAc 95:5 to 1:1 to 0:1), yielding product **3** (156 mg, 51%) as a brown solid.

TLC: (difluorocyclopropene intermediate) (PE/Et_2O 4:1) R_f= 0.45.

TLC: (**6**) (EtOAc) R_f= 0.50.

^{1}H NMR: ($CDCl_3$, 500 MHz): δ 8.53 (s, 1H, H-C(2')), 2.89 – 2.82 (m, 4H, H-C(2), H-C(4)), 2.22 (p, *J* = 7.3 Hz, 2H, H-C(3)).

13C NMR: (CDCl3, 125 MHz): δ 168.85 (C(1)), 168.59 (C(1')), 157.21 (C(3')), 149.25 (C(2')), 142.30141.82, 140.84–140.41, 140.16–139.86, 139.09–138.37, 137.07–136.71, 125.01–124.60 (m, (C-arom)), 32.25 (C(2)), 26.80 (C(4)), 20.97 (C(3)).

^{19}F NMR: ($CDCl_3$, 376 MHz): δ -152.03 – -153.11 (m), -157.47 (t, *J* = 21.7 Hz), -160.76 – -162.53 (m).

ESI⁺-HRMS: calculated for C13H7F5O3Na ([M+Na]⁺) 329.0208, found 329.0200

CPO-Alkyne, (4)

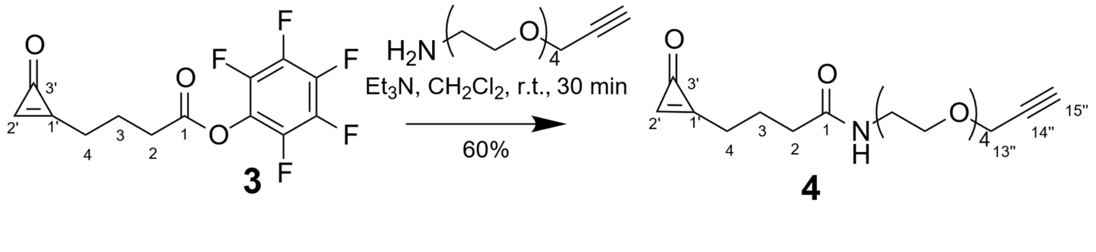

Perfluorophenyl 4-(3’-oxocycloprop-1’-en-1’-yl)butanoate **3** (10.0 mg, 32.7 µmol, 1 equiv.) was dissolved in CH_2Cl_2 (1 mL), in a vial equipped with a magnetic stirrer bar. After adding Et_3N (15 µL), NH_2-PEG$_4$-alkyne (9.0 mg, 39.0 µmol, 1.2 equiv.) was added in 3 mg portions until no starting material was observed via TLC. The crude reaction mixture was concentrated and purified by CC (CH_2Cl_2/MeOH 93:7 to 85:15) to yield product **4**, **CPO-Alkyne,** (6.9 mg, 60%) as a colourless oil.

TLC: (CH_2Cl_2/MeOH 9:1) R_f= 0.20.
^{1}H NMR: ($CDCl_3$, 400 MHz): δ 8.47 (s, 1H, H-C(2’)), 6.68 (br, 1H, NH), 4.19 (d, *J* = 2.4 Hz, 2H, H-C(13’’)), 3.73 – 3.59 (m, 12H, 6 x CH_2-PEG), 3.57 – 3.52 (m, 2H, CH_2-PEG), 3.44 (q, *J* = 5.2 Hz, 2H, C$\underline{H}_2$NH(PEG)), 2.76 (t, *J* = 7.1 Hz, 2H, H-C(4)), 2.44 (t, *J* = 2.3 Hz, 1H, H-C(15’’)), 2.35 (t, *J* = 7.1 Hz, 2H, H-C(2)), 2.06 (p, *J* = 7.1 Hz, 2H, H-C(3)).
^{13}C NMR: ($CDCl_3$, 100 MHz): δ 171.82 (C(1)), 169.66 (C(1’)), 158.03 (C(3’)), 148.48 (C(2’)), 79.61 (C(14’’)), 74.84 (C(15’’)), 70.65, 70.61, 70.44, 70.29, 69.92, 69.22 (6 x CH_2-PEG), 58.53 (C(13’’)), 39.36 (CH_2NH(PEG)), 34.89 (C(2)), 26.96 (C(4)), 21.71 (C(3)).
ESI$^+$-HRMS: calculated for C18H28O6N1 ($[M+H]^+$) 354.1911, found 354.1902.

CPO-PEG (5)

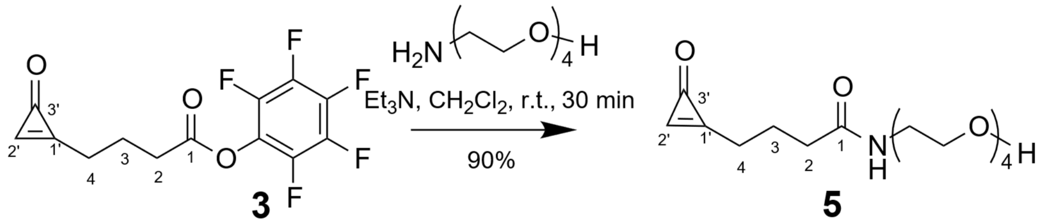

Perfluorophenyl 4-(3’-oxocycloprop-1’-en-1’-yl)butanoate **3** (10.0 mg, 32.7 µmol, 1 equiv.) was dissolved in CH_2Cl_2 (1 mL), in a vial equipped with a magnetic stirrer bar. After adding Et_3N (15 µL), NH_2-PEG$_4$-OH (9 mg, 46.6 µmol, 1.4 equiv.) was added in 3 mg portions until no starting material was observed via TLC. The crude reaction mixture was concentrated and purified by CC (CH_2Cl_2/MeOH 93:7 to 4:1) to yield product **5**, **CPO-PEG,** (9.2 mg, 90%) as a colourless oil.

TLC: (CH_2Cl_2/MeOH 93:7) R_f= 0.27.
^{1}H NMR: ($CDCl_3$, 400 MHz): δ 8.46 (s, 1H, H-C(2’)), 7.40 (br, 1H, NH), 3.75 – 3.70 (m, 4H, 2 x CH_2-PEG), 3.68 – 3.59 (m, 8H, 4 x CH_2-PEG), 3.54 – 3.51 (m, 2H, CH_2-PEG), 3.44 (q, *J* = 5.0 Hz, 2H, C$\underline{H}_2$NH(PEG)), 2.75 (t, *J* = 7.1 Hz, 2H, H-C(4)), 2.34 (t, *J* = 7.2 Hz, 2H, H-C(2)), 2.06 (p, *J* = 7.1 Hz, 2H, H-C(3)).

^{13}C NMR: ($CDCl_3$, 125 MHz): δ 171.92 (C(1)), 169.77 (C(1')), 158.04 (C(3')), 148.57 (C(2')), 72.68, 70.82, 70.57, 70.32, 70.12, 70.07, 61.65 (7 x CH_2-PEG), 39.30 (**C**H_2NH(PEG)), 34.81 (C(2)), 27.11 (C(4)), 21.74 (C(3)).

ESI$^+$-HRMS: calculated for C15H26O6N1 ($[M+H]^+$) 316.1755, found 316.1744.

CPO-PEG (6)

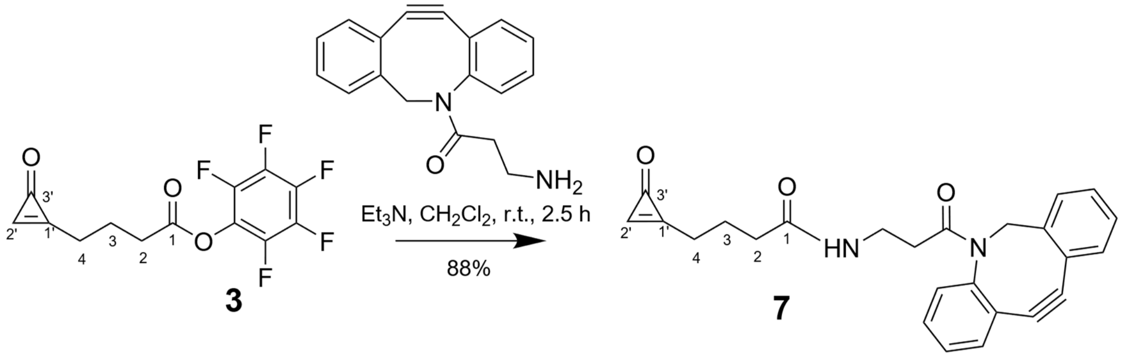

In a fumehood, DBCO-amine (14 mg, 0.051 mmol, 1.0 equiv) and CPO-PFP (23 mg, 0.076 mmol, 1.5 equiv) were weighed into a glass vial (2 mL) and dissolved in DCM (1.5 mL). To the reaction mixture was added triethylamine (10.8 mg, 15 µL, 0.102 mmol, 2 equiv) and the resulting mixture was stirred for 2.5 h at ambient temperature (23 °C). Silica gel (ca. 2 mL) was added to the reaction mixture and volatile material was removed from the mixture to give a fine powder. The material was purified by column chromatography (DCM/MeOH; 98:2→95:5) to provide CPO-DBCO as a yellow oil (18 mg, 0.045 mmol, 88%).

TLC: (DCM/MeOH 95:5) Rf= 0.38.

^{1}H NMR: (400 MHz, CDCl3) δ 8.44 (s, 1H), 7.75–7.66 (m, 1H), 7.51–7.25 (m, 8H), 6.28 (s, 1H), 5.16 (d, J = 14.0 Hz, 1H), 3.72 (d, J = 13.9 Hz, 1H), 3.44–3.19 (m, 2H), 2.68 (td, J = 7.1, 1.4 Hz, 2H), 2.48 (ddd, J = 16.7, 7.4, 3.9 Hz, 1H), 2.22–1.90 (m, 5H).

^{13}C NMR: (101 MHz, CDCl3) δ 172.26, 171.39, 169.57, 157.77, 151.08, 148.60, 148.12, 132.26, 129.16, 128.78, 128.63, 128.51, 128.03, 127.42, 125.69, 123.05, 122.62, 114.86, 107.91, 77.48, 77.16, 76.84, 55.68, 35.42, 34.97, 34.82, 26.95, 21.59.

HRMS: calculated for C25H23N2O3 ($[M+H]^+$) 399.1709, found 399.1732.

NH_2-VTLPSTCGAS-$CONH_2$ peptide, (P1)

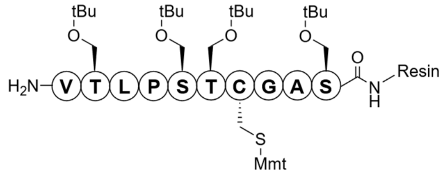

N-α-D-Fmoc protected amino acids were sourced from Bachem AG (Switzerland). Synthesis reagents and solvents were all obtained from NovaBioChem, Merck (UK) and used without further purification. Peptide sequences were prepared using automated solid phase peptide synthesis using the Prelude (PTI) synthesiser and standard Fmoc protocol with standard side chain protecting chemistries unless otherwise stated. P1 was synthesised as C-terminal carboxamides on Rink Amide MBHA resin (loading 0.36 mmol/g, 100-200 mesh) on a 0.15 mmol scale using the Prelude peptide synthesiser standard programme; double coupled amino acids (0.3 M in DMF, 2 x 1 hr) with DIPEA 35% *v/v* in NMP and HCTU 0.4M in DMF, deprotection using 20% piperidine in DMF, capping Ac_2O 8.7% *v/v* in DMF. All amino acids were coupled in the protected N-α-D-Fmoc form. The cysteine residue was coupled as Fmoc-Cys(Mmt)-OH. For characterisation, 2 mg of crude P1 was cleaved from the resin using a cleavage cocktail containing TFA (95%), triisopropylsilane (2.5%) and water (2.5%) for 5 hours at room temperature. The resin was removed by filtration and the cleavage solution removed in vacuuo. The peptide was precipitated by addition of diethyl ether, isolated by centrifuge at 3500 RPM, dried under a flow of dry nitrogen and subject to LC-MS analysis.

Ref.	Expected mass [M+H$^+$] (Da)	Observed mass (Da)
P1	935	935

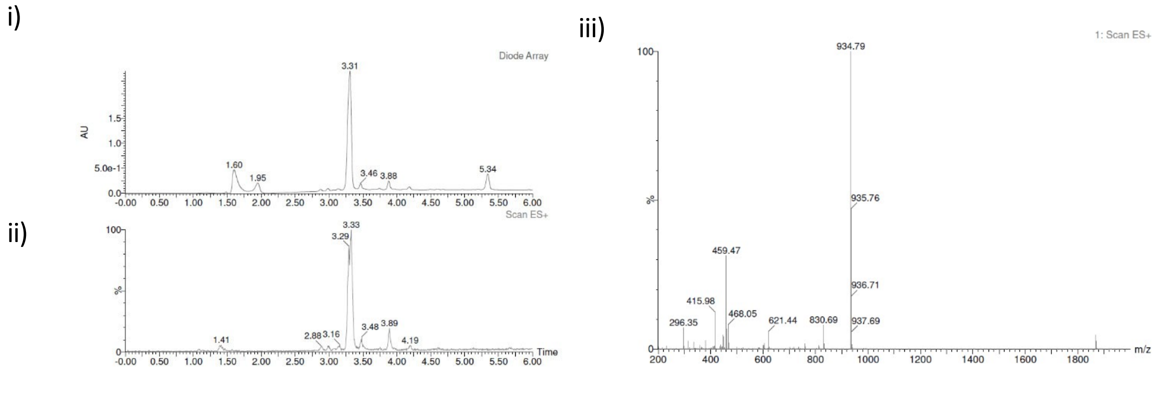

Figure S1. UV chromatogram (i), TIC (ii) and mass spectra (iii) obtained from LC-MS analysis of P1.

DBCO-PEG4-VTLPSTCGAS-CONH$_2$ peptide, (P2)

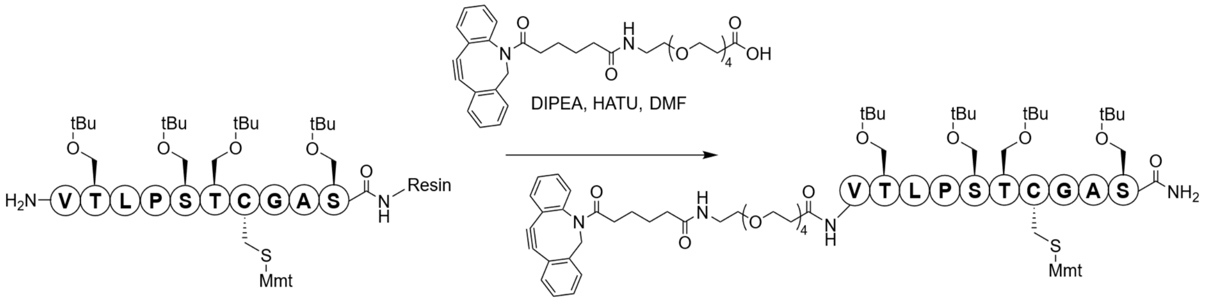

P2 was synthesised via the following manual coupling protocol: DBCO-NHCO-PEG$_4$-acid (SigmaAldrich, Catlog number: 759902) (2 equiv.), DIPEA (4 equiv.) and HATU (2 equiv.) were added to resin bound N-termini deprotected P1 in 10 mL of DMF and left shaking at room temperature for 1 hr. The reaction solution was then removed in vacuuo. For characterisation only, 1 mg of crude P2 was added to $(MeCN)_4CuBF_4$ (5 equiv., dry powder, SigmaAldrich, 677892) and a cleavage cocktail containing TFA (95%), triisopropylsilane (2.5%) and water (2.5%) for 5 hours at room temperature.[1] The resin was removed by filtration and the cleavage solution removed in vacuuo. The peptide was precipitated by addition of diethyl ether, isolated by centrifuge at 3500 RPM, dried under a flow of dry nitrogen and subject to LC-MS analysis.

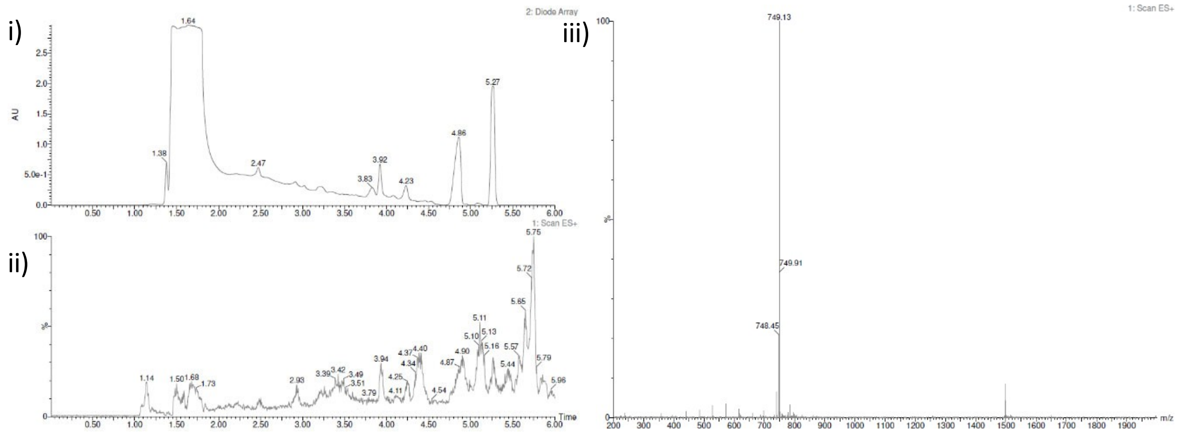

Ref.	Expected mass [M+H$^+$] (Da)	Observed mass (Da)
P2	1498	1498

Figure S2. UV chromatogram (i), TIC (ii) and mass spectra (iii) obtained from LC-MS analysis of P2.

DBCO-PEG4-VTLPSTC(PFA)GAS-CONH$_2$, (P3)

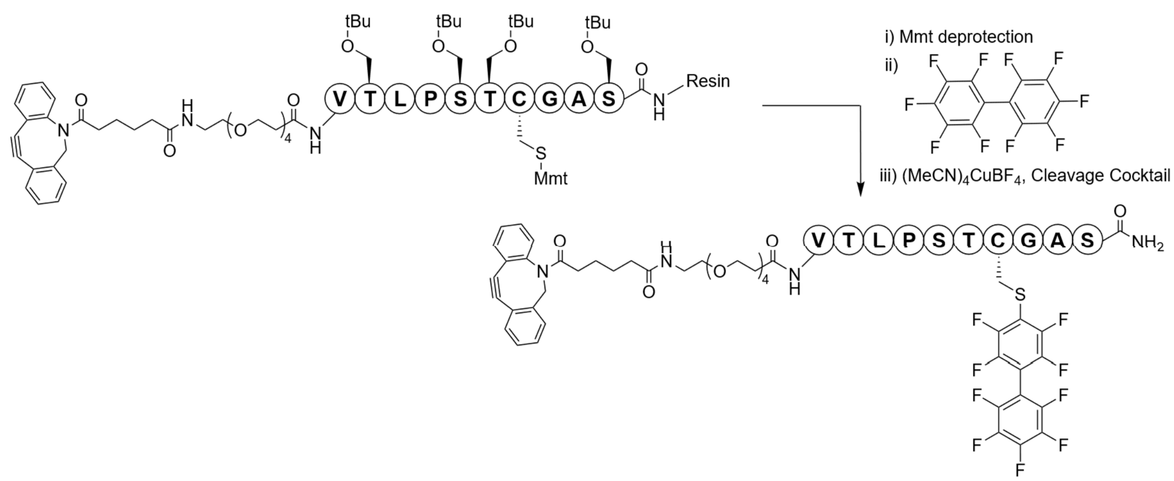

After washing with DCM and DMF, resin bound P2 was washed with 1% TFA (v/v) and 5% TIPS in DCM, which resulted in cleavage of the mmt protecting group from the cysteine residue. Washing was performed until the flow through appeared colourless, indicating complete mmt deprotection. Decafluorobiphenyl (2 equiv., SigmaAldrich, D227) dissolved in DMSO and DIPEA (4 equiv.) was then added to cys-deprotected resin bound P2 suspended in 250 mL of DMF. The reaction mixture was left stirring for 1 hr at room temperature. After washing with DCM and DMF, crude P3 was simultaneously cleaved from the resin and side-chain deprotected by treatment with $(MeCN)_4CuBF_4$ (5 equiv., dry powder) and a cleavage cocktail (2.5% water (v/v) and 1% TIPS (v/v) in neat TFA) for 4 hours at room temperature.[1] The resin was removed by filtration and the cleavage solution removed in vacuuo. The peptide was precipitated by addition of diethyl ether, isolated by centrifuge at 3500 RPM and dried under a flow of dry nitrogen to yield crude P3 as a white solid. Crude P3 was dissolved in 10 mL of water:ACN (90:10) and purified by RP-HPLC to yield P3 as a white solid (4.1 mg, 29% yield from P1), HRMS (ESI) m/z calc. for [M+2H]2+: 905.8601, found 905.6948.

i) ii) iii)

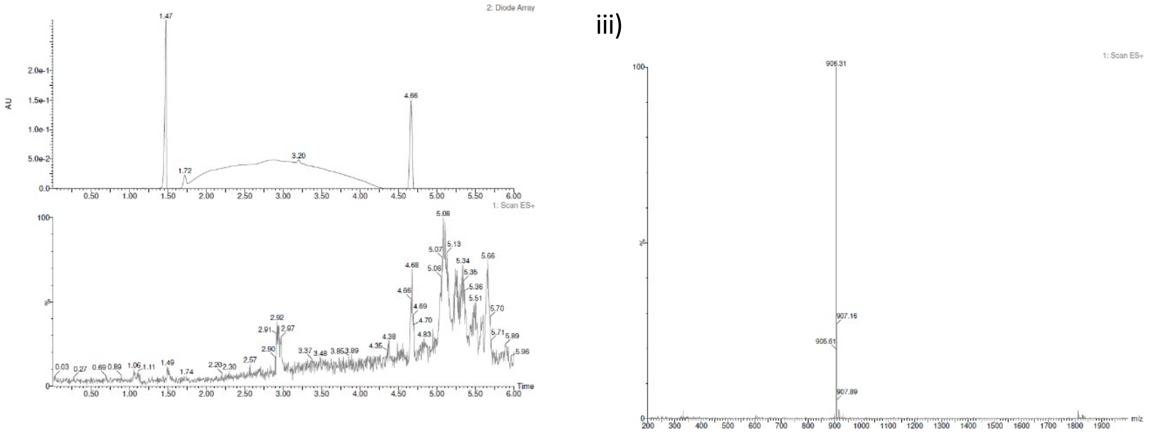

Ref.	Expected m/z [M+2H$^+$]	Observed m/z
P3	906	906

Figure S3. UV chromatogram (i), TIC (ii) and mass spectra (iii) obtained from LC-MS analysis of P3.

NH_2-GGGK(azido)-$CONH_2$ peptide, (P4)

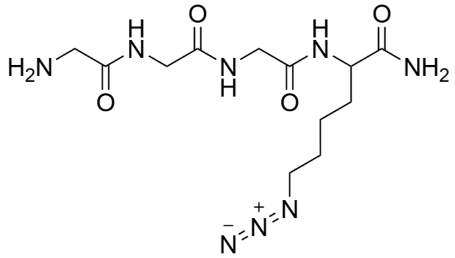

P4 was synthesised as C-terminal carboxamides on Rink Amide MBHA resin (loading 0.36 mmol/g, 100-200 mesh) on a 0.15 mmol scale using the Prelude peptide synthesiser standard programme; double coupled amino acids (0.3 M in DMF, 2 x 1 hr) with DIPEA 35% v/v in NMP and HCTU 0.4 M in DMF, deprotection using 20% piperidine in DMF, capping Ac_2O 8.7% v/v in DMF. All amino acids were coupled in the protected N-α-D-Fmoc form. After washing with DCM and DMF, Crude P4 was simultaneously cleaved from the resin and side-chain deprotected by treatment with 2.5% water (v/v) and 1% TIPS (v/v) in neat TFA for 4 hours at room temperature. The resin was removed by filtration and the cleavage solution removed in vacuuo. The peptide was precipitated by addition of diethyl ether, isolated by centrifuge at 3500 RPM and dried under a flow of dry nitrogen to yield crude P4 as a white solid. Crude P4 was dissolved in 10 mL of water:ACN (90:10) and purified by RP-HPLC to yield P4 as a white solid. P4 was subsequently analysed by LC-MS.

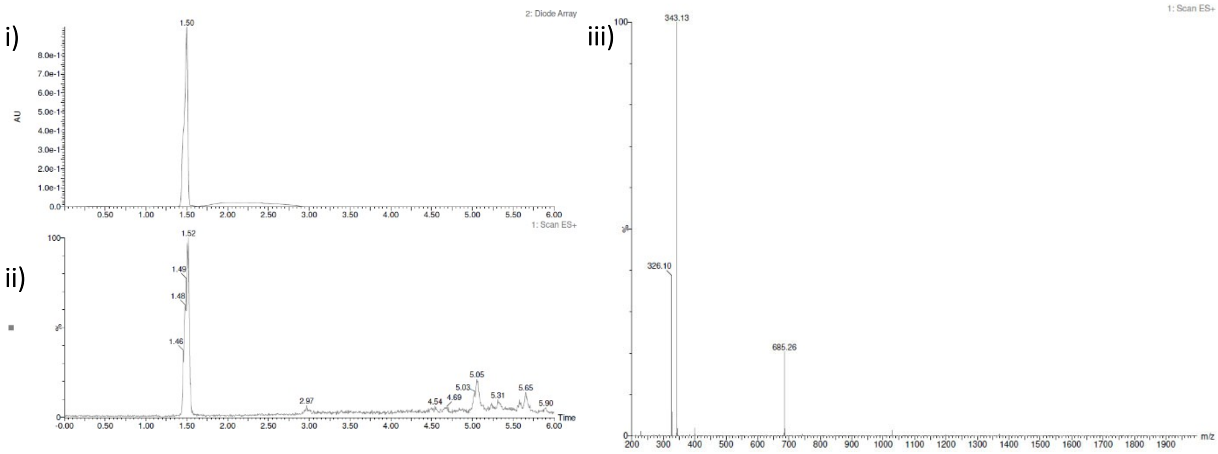

Ref.	Expected mass $[M+H^+]$ (Da)	Observed mass (Da)
P4	343	343

Figure S4. UV chromatogram (i), TIC (ii) and mass spectra (iii) obtained from LC-MS analysis of P4.

S2 Molecular biology

S2.1 Plasmid design

With the exception of GALaXy-containing constructs, pEU expression vectors, which encode EF-1α promoter, polyadenylation site, ampicillin resistance gene, bacterial origin of replication, and the origin of EBV replication (oriP), were used for expression of HC and LC proteins in EBNA1-producing mammalian cells. This was achieved by inserting codon optimised genes (GeneArt Thermo Fischer Scientific) encoding anti-CD33 heavy and light variable regions into separate pEU vectors (LC = pEU3.4 (encodes kappa LC constant region), HC = pEU1.4 (encodes IgG1 (triple mutant (TM)) HC constant region)).

For GALaXy-containing constructs, separate pBAMF expression vectors were used for cloning of HC and LC. These vectors are based on the pDest12.2 vector (Thermo Fisher) which encode the viral DNA replication (OriP) sequence and the viral Epstein-Barr nuclear antigen 1 (EBNA1) protein under the control of the simian virus 40 (SV40) promoter. The codon optimised genes (GeneArt Thermo Fischer Scientific) encoding anti-CD33 heavy and light variable regions were inserted into separate pBAMF vectors, which contain IgG1 (triple mutant (TM)) HC constant region and kappa LC constant region respectively.

Cloning of variable regions and constant region mutants was conducted using Gibson assembly processes, consisting of vector linearization, DNA purification, enzymatic assembly. Where appropriate, site-directed mutagenesis was also used. These processes were followed by E.coli transformation and plasmid DNA production.

S2.2 E. Coli transformation and production of plasmid clones

DNA samples (1 µL from Gibson assembly mix or SDM mix) were added to a freshly thawed aliquot (100 µL) of chemically competent cells (DH5 Alpha cells, Zymo Research, T3007) following manufactures protocol. Bacteria were plated on pre-heated 2xTYAG plates and left overnight at 37 °C. Single clones from the 2xTYAG plates were used to inoculate 5 mL of 2xTYAG media. The subsequent

cultures were then grown up overnight at 37 °C while shaking at 200 RPM. 500 µL of 50 % glycerol were added to 500 µL of the overnight stock and the resulting mixtures – glycerol stocks - were stored at -80 °C. The plasmid from the remaining culture was then harvested using a Qiagen Plasmid Plus Mini Kit following manufactures protocol and sequenced. Purified plasmids were sequenced by SourceBioScience using the custom forward and reverse sequencing primers obtained from SigmaAldrich. To prepare plasmid for DNA mammalian expression, individual clones were cultured by first inoculating 2TYAG (3 mL) with glycerol stock using a loop. Following growth for 8 hours at 37 °C, the 3 mL culture was used to inoculate 500 mL 2xTYAG and bacteria was cultured overnight. Bacteria were harvested by centrifugation (15 min, 8000 RPM) and plasmid DNA was extracted using Mega Plus DNA kit (Qiagen). Purity and concentration of the resulting DNA was assessed by nanodrop at A280/A260.

S3 Protein expression and purification

S3.1 Expression

With the exception of GALaXy-containing constructs, all proteins were expressed in CHO G22 as per the following protocol derived from Daramola et al:[1]

In a 15 mL falcon, a 5 mL of solution containing the required plasmid(s) (250 µg for each plasmid (obtained following Qiagen Plasmid Plus Mega Kit purification)) in 150 mM NaCl was prepared. 5 mL of linear polyethylenimine (PEI) Max (0.6 mg/mL, Polysciences Europe) in 150 mM NaCl was then added and the resulting PEI:DNA mixture was vortexed for 10 seconds and incubated at room temperature for 1 minute. The subsequent turbid PEI:DNA complex solution was added to 500 mL of CHO G22 cells in M20a medium in a 850 mL roller bottle containing 5 mL of Pen/Strep antibiotic. The cultures were incubated at 37 °C, 140 RPM with 5% CO_2 and 80% humidity. After at least 4 h, MedImmune-proprietary nutrient supplement was added to the cultures, and the cultures were moved to 34 °C, 140 RPM with 5% CO_2 and 80% humidity for 3 days. At the end of the 3rd day, further supplement was added to the cultures. The cultures were grown at 34 °C, 140 RPM with 5% CO_2 and 80% humidity for a further 4 days. On day 6, the cultures were centrifuged at 2200 RPM for 60 min at 4 °C. The supernatant was collected and filtered through a 0.22 µm filter and stored at 4 °C until purification.

GALaXy-containing constructs were expressed in HEK293F GALE KO cells as per the following protocol: In 15 mL falcon, a 10 mL of solution containing the required plasmid(s) (100 µg for each plasmid (obtained following Qiagen Plasmid Plus Mega Kit purification)) in Opti-MEM™ was prepared. In a

separate 15mL falcon, Fectin (300 µL, 293fectin™ Transfection Reagent, Thermo Fisher Scientific, 2347019) was mixed with 9.7 mL of Opti-MEM™. After incubation at room temperature for 5 minutes, the two solutions were mixed. After a further 20 minute incubation, the DNA-293fectin solution was added to GALE KO HEK293F cells (3x10^8 cells in 280 mL) cultured in FreeStyle™ 293 expression media (Thermo Fisher Scientific) in the presence of N-Azidoacetylgalactosamine-tetraacylated (final concentration 100 µM, 150 µL of 200 mM DMSO stock solution, SigmaAldrich, 900915) and Pen/Strep in a 850 mL roller bottle. Following transfection, cells were cultured at 37 °C, 140 RPM with 5% CO_2 and 80% humidity. Cells were fed by the addition of FreeStyle™ 293 expression media (300 mL, Thermo Fisher Scientific) and N-Azidoacetylgalactosamine-tetraacylated (300 µL of 200 mM DMSO stock solution) on day 3 of the incubation and harvested on day 6. On day 6, the cultures were centrifuged at 2200 RPM for 60 min at 4 °C. The supernatant was collected and filtered through a 0.22 µm filter and stored at 4 °C until purification.

S3.2 Purification following protein expression

The purification of antibody variants was performed using an ÄKTA Express system in combination with a HiTrap® MabSelect SuRe™ 5 mL column (GE Healthcare). After equilibrating the column and pump system with PBS, the sample was loaded onto the column, washed with PBS, and eluted with 0.1 M glycine (pH 2.7). Following elution the column was sequentially washed with H_2O, 0.5 M NaOH, H_2O and 20% ethanol. The column was then stored at 4 °C in 20% ethanol. Fractions of interest were combined, buffer exchanged to PBS via PD-10 columns (Cytiva) following manufactures protocol, followed by storage at -80 °C in 2 mL aliquots.

Where necessary, SEC purification was performed using an ÄKTA Express system in combination with a HiLoad™ 16/600 Superdex™ 200 pg column (Cytiva). After equilibrating the column and pump system with 2xPBS, the sample was manually loaded onto the column via a 5 mL loop, followed by elution with 2xPBS (1 mL/min). The ÄKTA Pure system with column attached was then sequentially washed with H_2O, 0.5 M NaOH, H_2O and 20% ethanol. The column was then stored at 4 °C in 20% ethanol. Fractions of interest were combined.

S4 Tissue culture

Cells were maintained in a humidified atmosphere of 5% CO_2 at 37 °C and cultured in the media stated below. Adherent cells were grown to approximately 80-90% confluency. Cells were detached using Trypsin (5 mL per T175 flask, Invitrogen 25200056) with 10 minute incubation at 37 °C. Growth media (20 mL per flask) was added to stop the reaction. Cell viability and density was measured using a Vi-Cell XR cell counter.

S4.1 CHO G22

CHO G22 cells (in house) were cultured in M20a medium (in house).

S4.2 HEK293F GALE KO

HEK293F GALE KO cells (in house) were cultured in FreeStyle™ 293 expression media (Thermo Fisher Scientific, 12338018).

S4.3 HEK 293 CD33+

HEK 293 CD33+ cells (HEK 293 Jump-In hCD33, generated in-house) were cultured in T175 flasks and routinely maintained in growth media outlined below.

HEK 293 Jump-In hCD33			
**Component**	**Volume (mL)**	**Source**	**Catalogue number**
DMEM (high glucose) with L-glutamine	500	Invitrogen	41966
Foetal bovine serum	50	Invitrogen	42F3393K
NEAA (x100 stock)	5	Invitrogen	11140
Geneticin (G418) (50 mg/mL stock)	10	Invitrogen	10131
Blasticidin (10 mg/mL stock)	0.250	Invitrogen	A11139-03

S4.4 HEK 293 CD33-

HEK 293 CD33- cells (HEK 293 Jump-In TREX, Invitrogen) were cultured in T175 flasks and routinely maintained in growth media outlined below.

HEK 293 Jump-In TREX			
**Component**	**Volume (mL)**	**Source**	**Catalogue number**
DMEM (high glucose) with L-glutamine	500	Invitrogen	41966
Foetal bovine serum	50	Invitrogen	42F3393K
NEAA (x100 stock)	5	Invitrogen	11140
Blasticidin (10 mg/mL stock)	0.250	Invitrogen	A11139-03
Hygromycin (50 mg/mL stock)	1	Invitrogen	10687-010

S5 Characterisation and stability methods

S5.1 HP-SEC

HP-SEC analysis was performed using an Agilent 1100 series in combination with a TSKgel G3000SWXL column (TOSOH, 7.8 mm I.D x 30 cm, 5 µM, 0008541) fitted with a TSKgel SWXL Guardcolumn (TOSOH, 6.0 mm I.D x 3 cm, 7 µM, 0008543). After equilibrating the column and pump system with HPLC buffer (0.1 M sodium phosphate dibasic anhydrous, 0.1 M sodium sulfate, pH 6.8) at 1 mL/min, standards (Biorad, Gel filtration standard, 151-1901) or samples (diluted to 1 mg/ml in PBS) were injected (70 µL). Standard injections were performed at the start and end of each analysis to confirm system suitability. An example standard chromatogram is given below. Following analysis, Agilent 1100 system with column attached was then sequentially washed with H_2O and 20% ethanol.

Standards Start

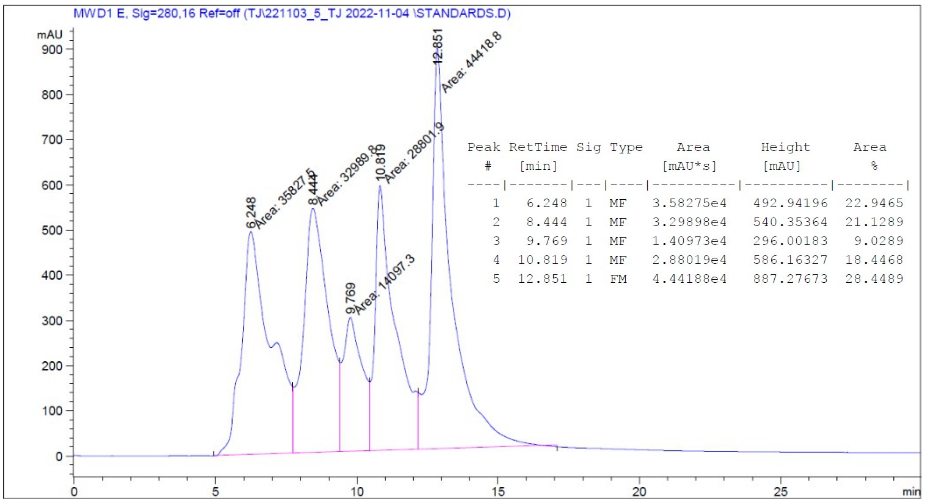

Standards End

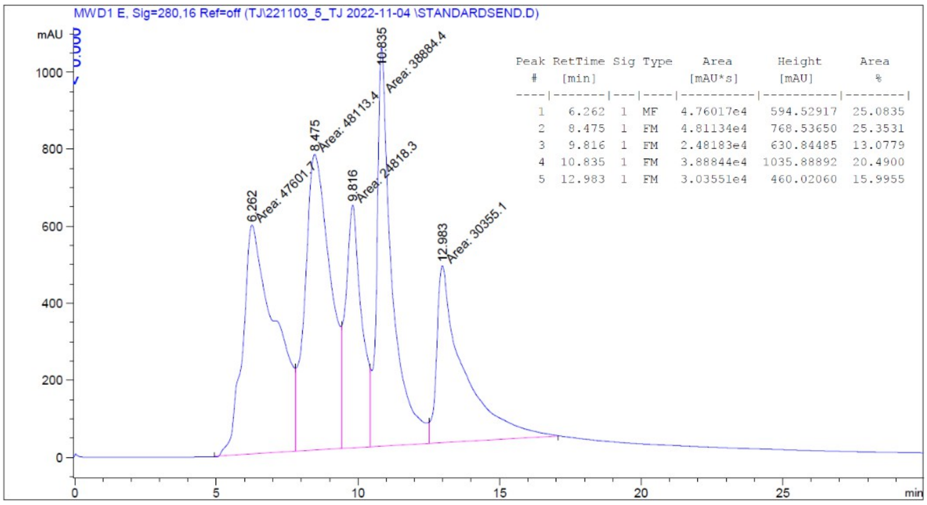

**Component**	**Molecular weight (Da)**
Thyroglobulin (bovine)	670,000
y-globulin (bovine)	158,000
Ovalbumin (chicken)	44000
Myoglobin (horse)	17000
Vitamin B12	1350

S5.2 Determination of protein/DNA concentration

Protein and DNA concentrations were measured using a plate reader (e.g. a Lunatic instrument from Unchained Labs) or nanodrop (Thermo) at either A280 (proteins) or A260 (DNA). For proteins, individual extinction coefficients were calculated from the primary sequence.

S5.3 Bio-Layer Interferometry

Antibody samples were analysed using a Basic Kinetic Experiment setup on an Octet® RED384 from ForteBio in combination with Anti-human IgG Fc Capture (AHC) sensors (ForteBio). Prior to measurement, the biosensor tips were rehydrated for at least 10 min in 200 µL of DPBS supplemented with 0.1% BSA and 0.02% Tween20 (BuffA). Tilted bottom 384-well plates were used with 50 µL solution per well, which was centrifuged at 1000 RPM for 2 mins before measurements were performed. Tips underwent the following steps at 25 °C with 500 Hz: Baseline (BuffA) (60 s), Loading (Antibody at 32 nM) (50 s), Baseline (BuffA) (60 s), Association (CD33 antigen (Acrobio, CD3-H5226) ranging from 25 nM to 1.56 nM) (300 s), Dissociation (BuffA) (900 s). Experimental runs were limited to 1 hour to minimise evaporation. Raw sensorgrams were processed in R (GNU project) by first subtracting the background from a reference sample (non-binding antibody loaded, maximum antigen concentration).

S5.4 Surface plasmon resonance - FcRn binding

Antibody samples were run using a Basic Kinetic Experiment setup on a Biocore T200 SPR Biosensor (cytiva 28-9750-01) in combination with Series S Sensor chip CM5 (cytiva, BR100668). Prior to measurement, FcRn (6 µg/µL, in house) diluted in Acetate 5.0 (cytiva, BR100351) was immobilised onto the chip using amine coupling kit (cytiva, BR-1000-50), NaOH (50 mM, Cytiva BR100358) and HBS-EP+ buffer (cytiva, BR100669) with a target level of 175. Following FcRn immobilisation, the immobilisation buffer was replaced with 500 mL of assay running buffer (50 mM NaPhos, 100 mM NaCl, 0.005% P20, pH 6.0) and a 2-fold dilution series from 1000 nM to 7.8 nM was prepared by adding 200 µL of previous sample dilution to 200 µL of pH 6.0 assay running buffer. Injection parameters for each sample were as follows: contact time (30 s), flow rate (30 µL/min) and dissociation time (60 s). Between each sample the chip was regenerated using PBS (pH 7.4) with injection parameters as follows: contact time (120 s) and flow rate (20 µL/min). Raw sensorgrams were processed in Biacore T200 Evaluation Software by first subtracting the background from a reference sample (FcRn, no

antibody) and a heterogeneous ligand model. All samples were run in duplicate. Rituxan Wildtype and YTE (in house) were used as positive controls.

S5.5 Surface plasmon resonance - FcγR binding

Antibody samples were analysed using a Basic Kinetic Experiment setup on a Biocore T200 SPR Biosensor (cytiva 28-9750-01) in combination with Series S Sensor chip CM5 (cytiva, BR100668). Prior to measurement, Anti-Histidine Antibody (AbD Serotek, MCA1396) (20 µg/mL, in house) diluted in Acetate 5.0 (cytiva, BR100351) was immobilised onto the chip using amine coupling kit (cytiva, BR-1000-50), NaOH (50 mM, Cytiva BR100358) and HBS-EP+ buffer (cytiva, BR100669) with a target level of 7000. FcγRIIIa (in house) was diluted to 2 µg/mL in HBS-EP+ buffer and a 2-fold dilution series from 500 to 7.8 nM was prepared by adding 200 µL of previous sample dilution to 200 µL HBS-EP+ buffer. For each sample, first FcγRIIIa was captured on the chip with the following parameters: contact time box (30 s), flow rate (10 µL/min). Sample was then injected with the following parameters: Contact time (60 s), dissociation time (60 s), flow rate 30 (µL/min). Between each sample the chip was regenerated using HCl (20 mM) with injection parameters as follows: contact time (35 s) and flow rate (30 µL/min). Raw sensorgrams were processed in Biacore T200 Evaluation Software by first subtracting the background from a reference sample (FcγR, no sample antibody) and a 1:1 binding model. Rituxan wildtype (in house) and Rituxan Triple Mutant (in house) were used as positive and negative controls respectively.

S5.6 Fluorescence-activated cell sorting

Cells were stained for viability using LIVE/DEAD™ Fixable Violet Dead Cell Stain Kit (Invitrogen, L34955) following manufacturers protocol. After seeding 2.5 x10^5 cells into each well of a V 96 well plate, the cells were first washed with 200 µL of FACS buffer (5% FBS GIBCO 10500064, 0.05% Sodium azide G Biosciences 786-299, 1% EDTA) and then incubated with 50 µL of FC block solution (Human IgG Fc Fragment (Rockland, 009-0103) diluted 1:150 in FACS buffer) for 20 mins at 4 °C and protected from light. The cell suspension was then centrifuged at 400 RPM for 5 mins and the supernatant removed. Following a further wash with 200 µL FACS buffer, the cells were treated with 50 µL of the antibody sample of interest at concentrations ranging from 0.05 µg/mL to 6.4 µg/mL in FACS buffer at 4 °C and protected from light. After 25 mins, the cells were washed with FACS buffer and then resuspended in 50 µL of the secondary antibody APC/Fire™ 750 anti-human IgG Fc antibody (Biolegend, Clone: M1310G05) at 2 ng/mL for 25 minutes at 4 °C. Finally, one additional washing step was performed prior to resuspension in 120 µL of FACS buffer. All experiments were conducted in duplicate.

The binding of the various antibodies was detected monitoring the fluorescence of the secondary antibody APC/FireTM 750 anti-human IgG Fc antibody using a BDLSRFortessa™ Flow Cytometer controlled by BD FACSDiva software (BD Biosciences). The first gate (P1) selected singlets, based on the forward scatter height (FSC-H) and the forward scatter area (FSC-A). The third gate (P3) selected the viable cells according to the FSC-A and the the fluorescence intensity of BV421 (channel to detect violet viability stain). Subsequently, the fluorescence intensity of APC-Cy7 (channel to detect APC/Fire) was measured. The data points were fitted to a log(agonist) *versus* response – variable slope (four parameter) equation (given below) using GraphPad Prism 9.

$$Y=b+ (a-b)/(1+10^{((LogEC50-X)*HillSlope)})$$

Where a and b are the upper and lower plateaux of the curves, X is the log(concentration of antibody), EC_{50} is the half maximal effective concentration and hillslope is the slope factor.

S5.7 SDS-PAGE

SDS-PAGE analysis was performed using NuPAGE 4-12% Bis-Tris gel (Invitrogen). Samples were prepared following manufactures instructions under reducing or non-reducing conditions. Samples

were loaded into the gel alongside a protein standard ladder (SeeBlue™ Plus2 Pre-stained, Invitrogen, LC5925). Electrophoresis was run with 1xNuPAGE™ MES SDS Running Buffer (ThermoFisher Scientific) at 200 V for 30 min. The subsequent gel was imaged using a ChemiDoc MP imaging system with appropriate filters applied in a sequential manner. The gel was then stained with Coomassie Blue (abcam) for 1 hour. After destaining in water for 2 hours, the gel was imaged using a ChemiDoc MP imaging system (Biorad) with Coomassie Blue filter applied.

S5.8 Stability study method

Protein solution (1 mg/mL, 190 µL, PBS) was prepared in a 0.5 mL Eppendorf. Glutathione solution (10 µL of a 20 mM stock solution, Sigma, G4251) was added at room temperature and the resulting mixture vortexed for 2 seconds. The protein solution was then shaken at 37 °C for 2 days with centrifugation performed every 24 hours. On day 2, an aliquot of the protein solution was removed and analysed by LC-MS in duplicate.

S6 Bioconjugation methods

General: All buffers were stored at room temperature and used within one month of preparation. All protein solutions (including enzymes) were stored at -80 °C. Prior to conjugation, protein solutions were thawed on ice with subsequent protein conjugates analysed and stored at 4 °C. All chemicals were stored per manufactures recommendations.

S6.1 TEV cleavage

Antibody solution (TEV Protease Reaction Buffer (NEB), 1 mg/mL) was incubated with of TEV protease (1 µL per 15 µg of antibody, 10,000 units/mL, NEB). The reaction was conducted with overnight incubation at 30 °C or 4 °C. The resulting protein solution was analysed by LC-MS.

S6.2 FLAG cleavage

Antibody solution (2 mg/mL in Tris (50 mM, pH 7.5), NaCl (150 mM), $CaCl_2$ (10 mM) buffer) was incubated overnight with enterokinase light chain (2.5 µL of 16,000 units/mL per 2 mg of protein, NEB) at 25 °C. The resulting protein solution was analysed by LC-MS.

S6.3 GlySERIAS cleavage

Antibody solution (20 µL, 1 mg/mL in TBS pH 7.6) was incubated with GlySERIAS (40 units, Genovis) at 37 °C for 1 hour. The resulting protein solution was analysed by LC-MS.

S6.4 Deglycosylation

Antibody solution (1 mL, 1.5 mg/mL in PBS) was incubated with PNGase F (15 µL, Gibco, A39245) overnight at 37 °C in a 1.5 mL Eppendorf. The resulting protein solution was analysed by LC-MS.

S6.5 CPO conjugation

Antibody solution (500-2 mL at 1 mg/mL in PBS) was incubated with DTT (10 equiv., 3.3-13.3 µL of 10 mM stock solution, SigmaAldrich, 10197777001) and CPO-Alkyne (15 equiv., 5-20 µL of 10 mM DMSO stock solution) at 25 °C for 4 hours. The resulting solution was then incubated with dehydroascorbic acid (SigmaAldrich, 261556, 20 equiv., 6.6-26.6 µL of 10 mM DMSO stock solution) at 25 °C for 1 hour.

S6.6 Sortase-mediated conjugation

Antibody solution (50-200 µL at 15 mg/mL in 50 mM Tris (pH 7.5), 150 mM NaCl and 10 mM $CaCl_2$) was incubated in an Eppendorf with sortase (0.05 equiv.) and P4 (50 equiv., 2.5-10 µL of 100 mM H_2O stock) at 37 °C for 1 hour.

S6.7 π-clamp conjugation

Antibody solution (40 µM in 100 mM phosphate buffer (pH 8.0), 1.25 M $(NH_4)_2SO_4$) was incubated in an Eppendorf with P3 (500 µM) and TCEP (10 mM) at 37 °C for 6 hours. The resulting solution was then purified using Amicon® Ultra 0.5 mL Centrifugal Filters (10K MWCO).

S6.8 MTGase-mediated conjugation

Antibody solution (10 µL, 17 mg/mL in PBS) was incubated with 11-Azido-3,6,9-trioxaundecan-1-amine (termed AzidoLinker) (80 equiv., 0.8 µL of 0.1M stock solution, SigmaAldrich, 17758) and microbial transglutaminase (1 unit, SigmaAldrich, SAE0159) overnight at 25 °C in a 0.5 mL Eppendorf.

S6.9 SPAAC conjugation

Antibody solution (100 µL-2 mL, 1 mg/mL in PBS) was incubated with SPAAC reagent (5 equiv., 0.33-6.66 µL of 10 mM DMSO stock) in a clean 0.5 mL Eppendorf at 25 °C for 1 h.

S6.10 Maleimide conjugation

Antibody solution (300-2000 µL, 1 mg/mL in PBS) was incubated with DL488-Mal (10 equiv., 3.3-22.2 µL of 5 mM DMSO stock) in a clean 0.5 mL Eppendorf at 25 °C for 1 h.

S6.11 Decysteinylation and rebridging

Antibody solution (750 µL, 4 mg/mL in PBS) was incubated with DTT (SigmaAldrich, 10197777001, 10 equiv., 20 µL of 10 mM stock solution) at 37 °C for 1 hour in a 1.5 mL Eppendorf. The reduced antibody solution was then mixed and incubated with dehydroascorbic acid (SigmaAldrich, 261556, 20 equiv., 40 µL of 10 mM DMSO stock solution) in a 1.5 mL Eppendorf at 25 °C for 45 minutes.

S7 Antibody conjugate characterisation

7.1 AntiCD33-(Wildtype)

AntiCD33-(Wildtype) was expressed and purified, following the protocols previously described, achieving a post-purification yield of 96 mg L^{-1}. LC-MS and biophysical analysis were conducted to assess the identity, integrity and functionality of the product.

i) Reduced

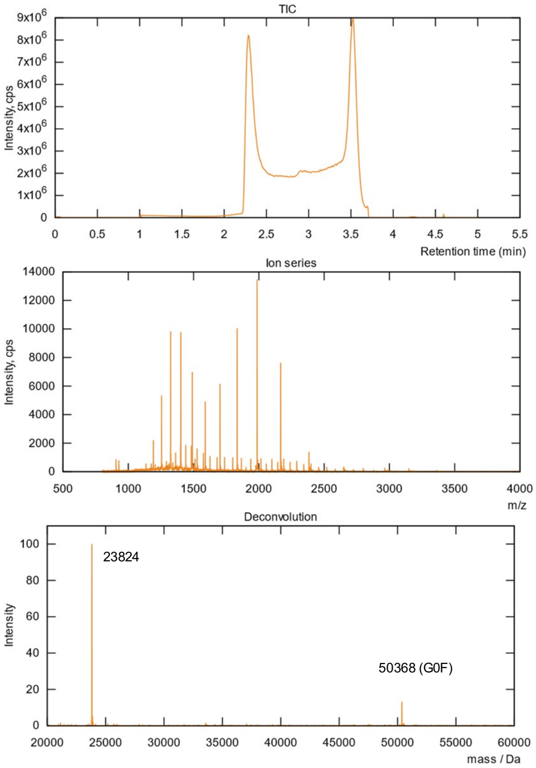

ii) Deglycosylated + Reduced

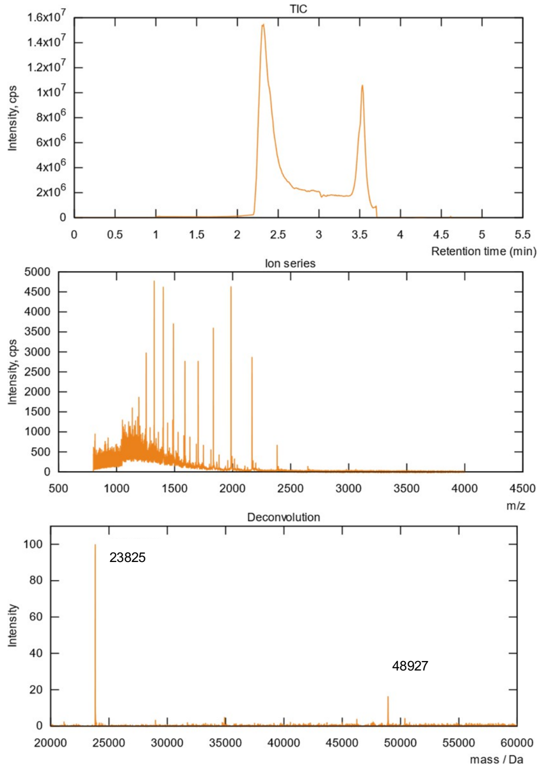

iii) Intact

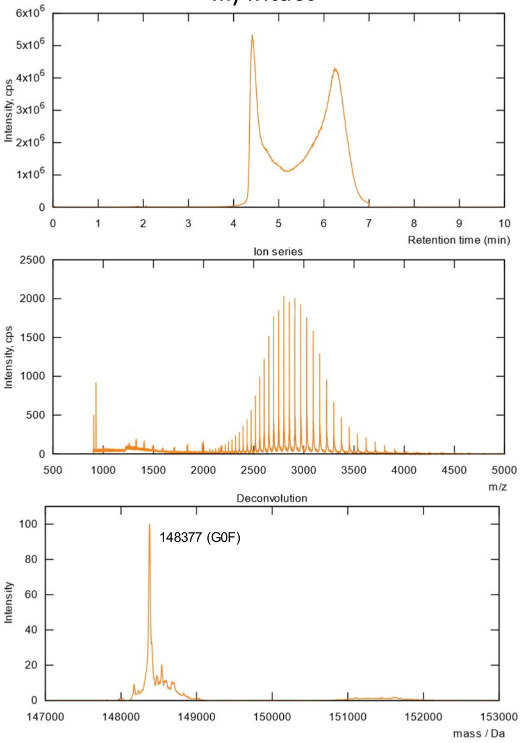

Ref.	Expected mass (Da)	Observed mass (Da)
i	LC: 23824<br>HC: 50367	LC: 23824<br>HC: 50368
ii	LC: 23824<br>HC: 48922	LC: 23825<br>HC: 48927
iii	148375	148377

Figure S5. UV chromatograms, ion series and deconvoluted mass spectra obtained from LC-MS analysis of antiCD33-(Wildtype) under i) reducing, ii) deglycosylated and reducing and iii) intact conditions.

i)

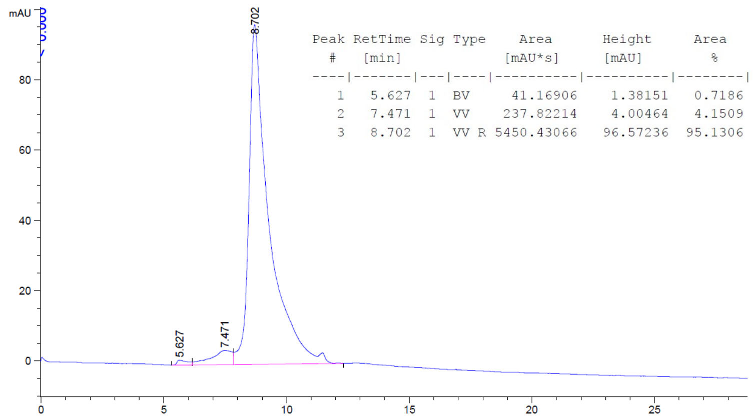

ii)

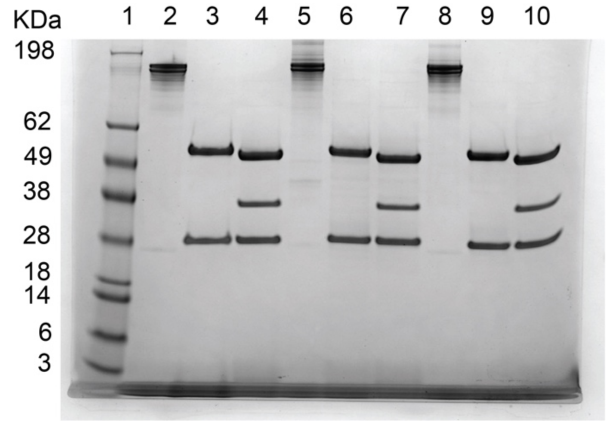

iii

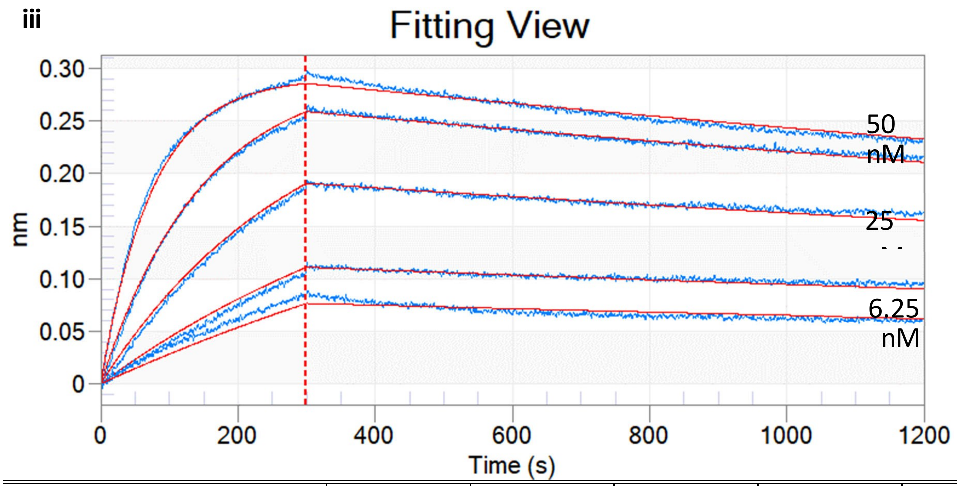

Sample	K_D (M)	K_D Fitting Error (M)	Kon (1/Ms)	Kon error (1/Ms)	Kdis (1/s)	Kdis error (1/s)
AntiCD33-(Wildtype)	8.68E-10	4.97E-12	2.63E+05	9.12E+02	2.28E-04	1.04E-06

Figure S6. Biophysical analysis of antiCD33-(Wildtype). i) UV chromatogram obtained from HP-SEC analysis, ii) Image of SDS-PAGE gel stained with Coomassie – lanes 2 to 4 (Lane 2 – NR: non-reducing, Lane 3 – R: reducing, Lane 4 –D+R: Deglycosylated and reducing). iii) Binding curves and corresponding kinetic parameters obtained from BLI analysis.

7.2 CPO

7.2.1 AntiCD33-(TEV-Cys)

AntiCD33-(TEV-Cys) was expressed and purified, following the protocols previously described, achieving a post-purification yield of 73 mg L^{-1} (normalization factor from antiCD33-(Wildtype) parallel expression: 1.04, normalised yield: 76 mg L^{-1}). LC-MS and biophysical analysis were conducted to assess the identity and integrity of the product.

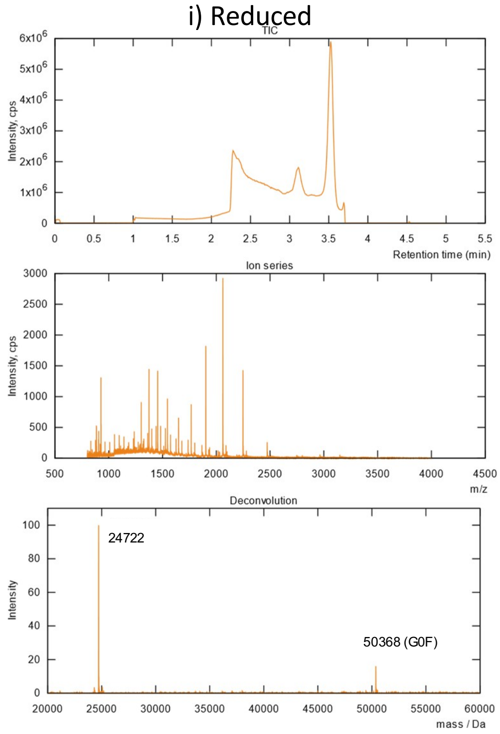

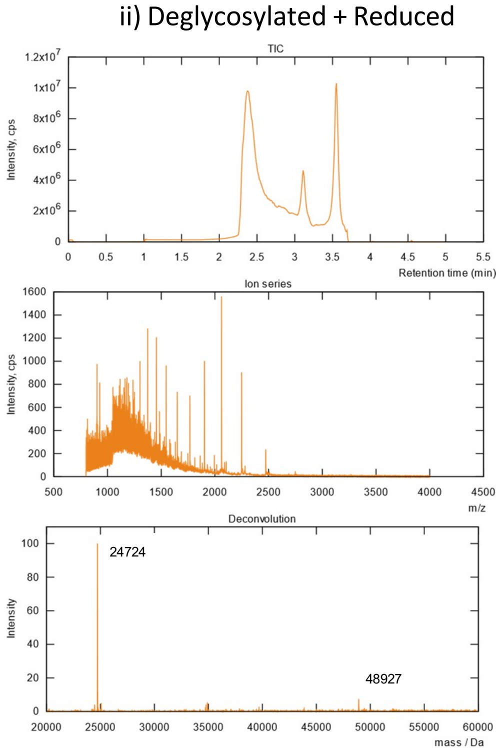

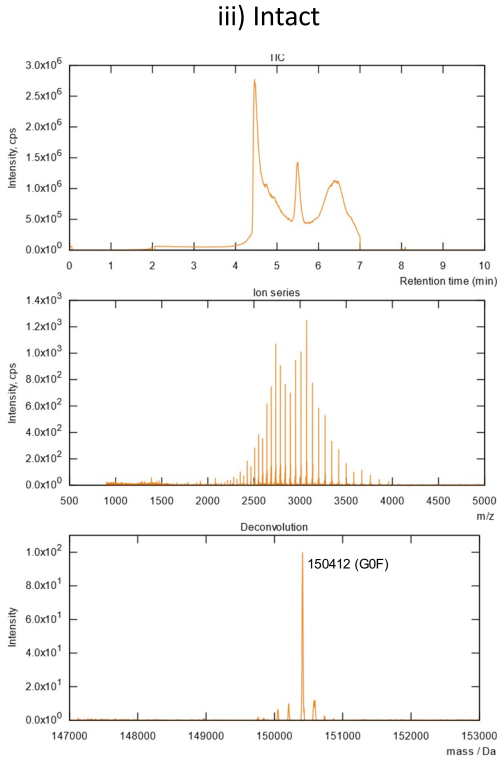

Ref.	Expected mass (Da)	Observed mass (Da)
i	LC: 24722<br>HC: 50367	LC: 24722<br>HC: 50368
ii	LC: 24722<br>HC: 48922	LC: 24724<br>HC: 48927
iii	150409	150412

Figure S7. UV chromatograms, ion series and deconvoluted mass spectra obtained from LC-MS analysis of antiCD33-(TEV-Cys) under reduced (i), deglycosylated and reduced (ii) and intact (iii) conditions.

i)

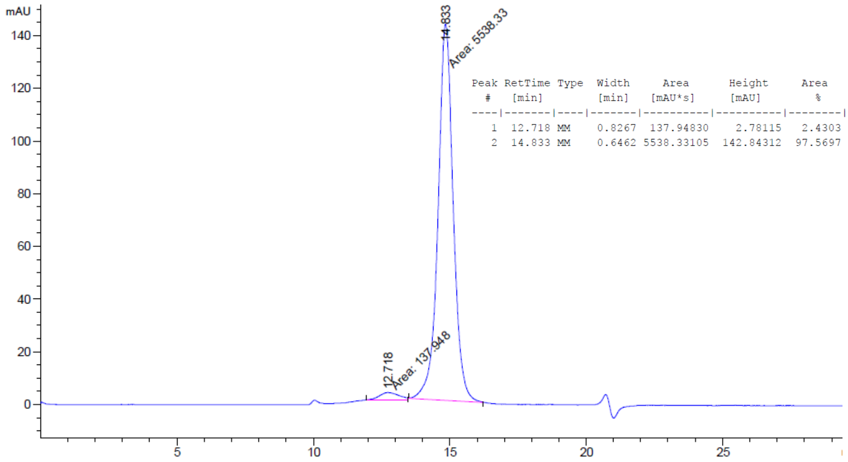

ii)

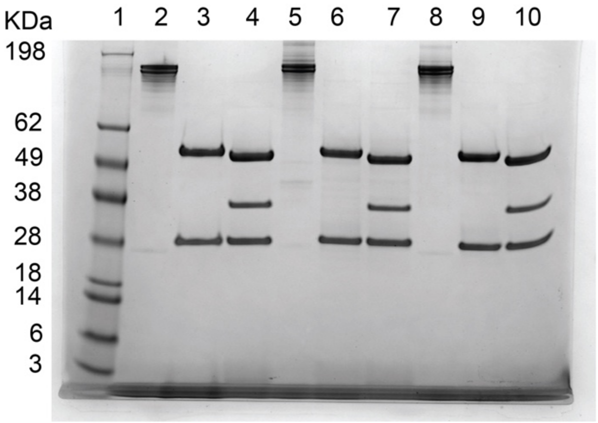

Figure S8. Biophysical analysis of antiCD33-(TEV-Cys). i) UV chromatogram obtained from HP- SEC analysis, ii) Image of SDS-PAGE gel stained with Coomassie – lanes 5 to 7 (Lane 5 – NR: non-reducing, Lane 6 – R: reducing, Lane 7 –D+R: Deglycosylated and reducing).

7.2.2 AntiCD33-(TEV-Cys) - Attempted TEV cleavage

TEV cleavage of antiCD33-(TEV-Cys) was attempted following method "TEV cleavage" described in S6.1. The results following LC-MS analysis are shown below.

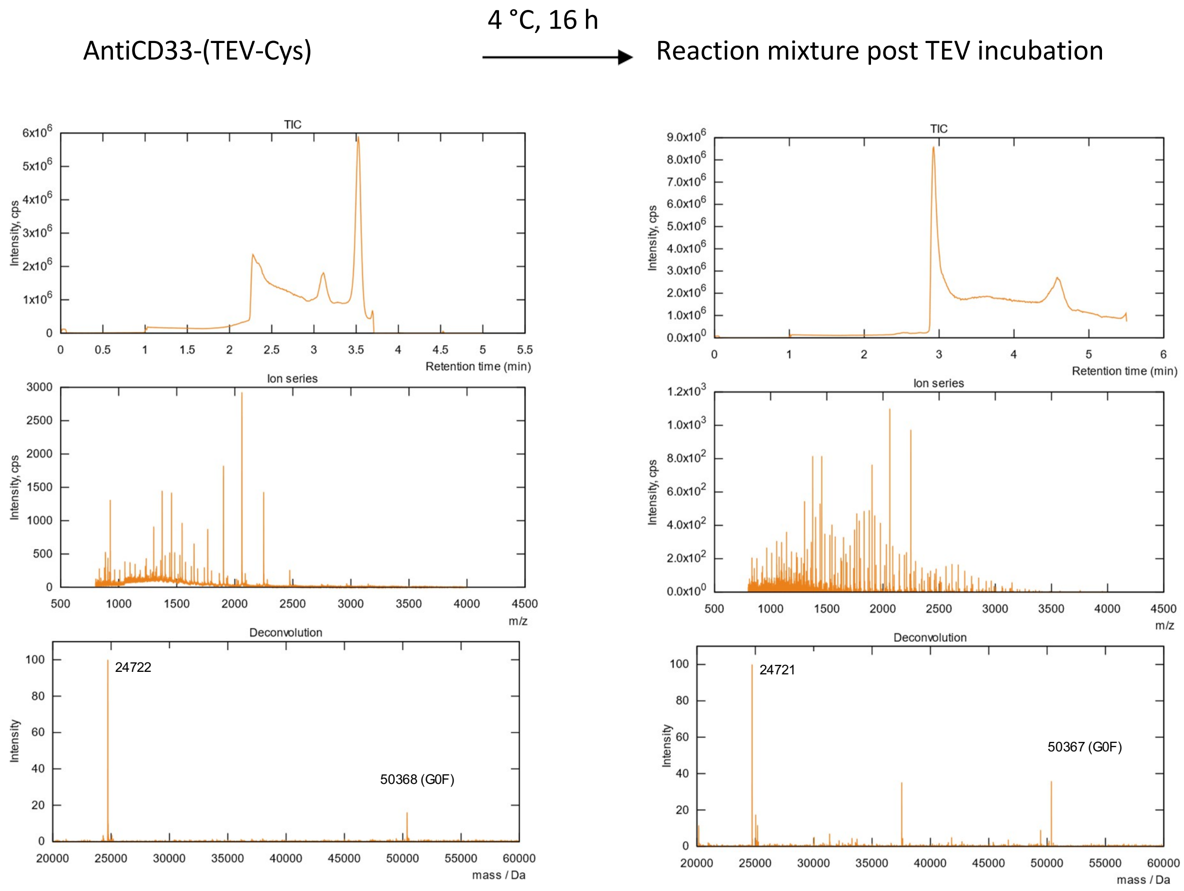

		Mass before reaction (Da)	Mass after reaction (Da)	Mass change (Da)
LC	Expected	24722	23927	-795
	Observed	24722	24721	-1
HC	Expected	50367	50367	0
	Observed	50368	50367	-1

Figure S9. UV chromatograms, ion series and deconvoluted mass spectra obtained from LC-MS analysis of antiCD33-(TEV-Cys) before and after TEV incubation at 4 °C.

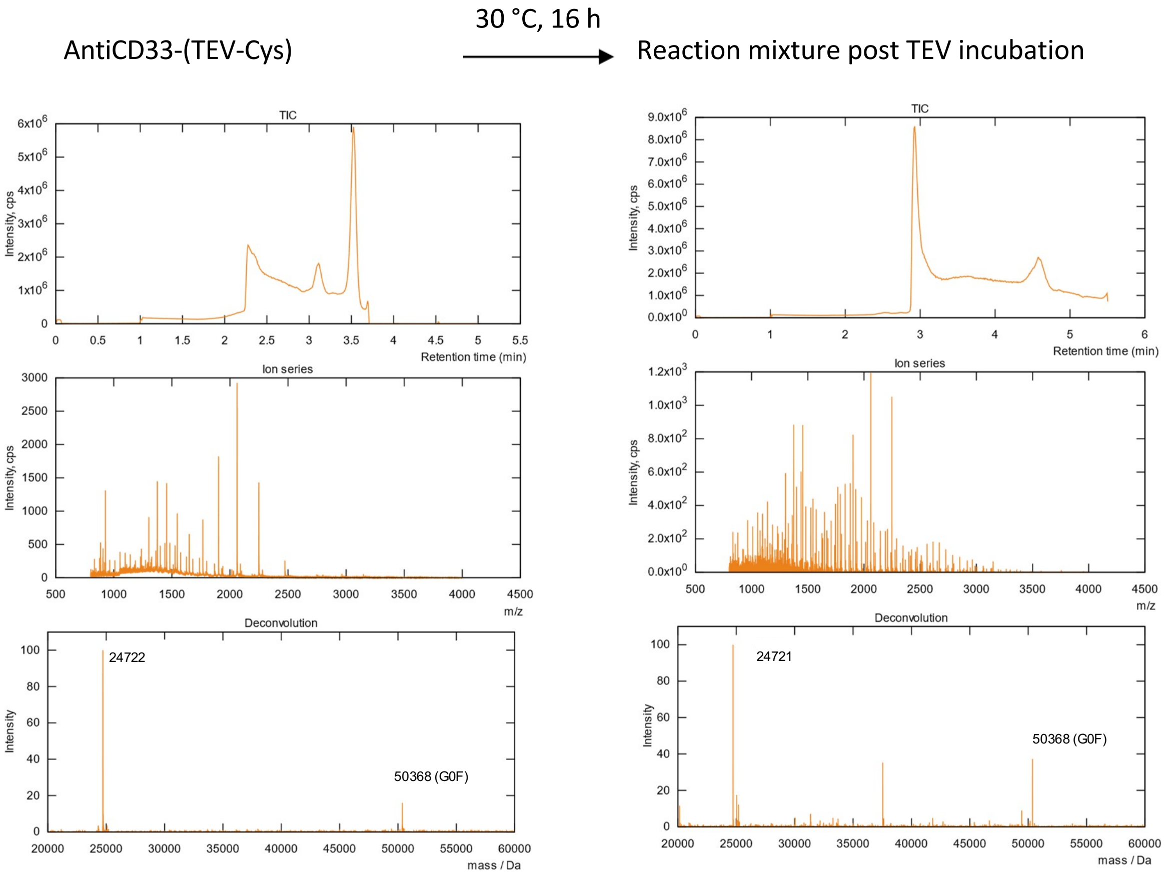

		Mass before reaction (Da)	Mass after reaction (Da)	Mass change (Da)
LC	Expected	24722	23927	-795
	Observed	24722	24721	-1
HC	Expected	50367	50367	0
	Observed	50368	50368	0

Figure S10. UV chromatograms, ion series and deconvoluted mass spectra obtained from LC-MS analysis of antiCD33-(TEV-Cys) before and after TEV incubation at 30 °C.

7.2.3 AntiCD33-(FLAG-Cys)

AntiCD33-(FLAG-Cys) was expressed and purified, following the protocol described in 6.2.4, achieving a post-purification yield of 30 mg L^{-1} (normalization factor from antiCD33-(Wildtype) parallel expression: 1.75, normalised yield: 53 mg L^{-1}). LC-MS and biophysical analysis were conducted to assess the identity and integrity of the product.

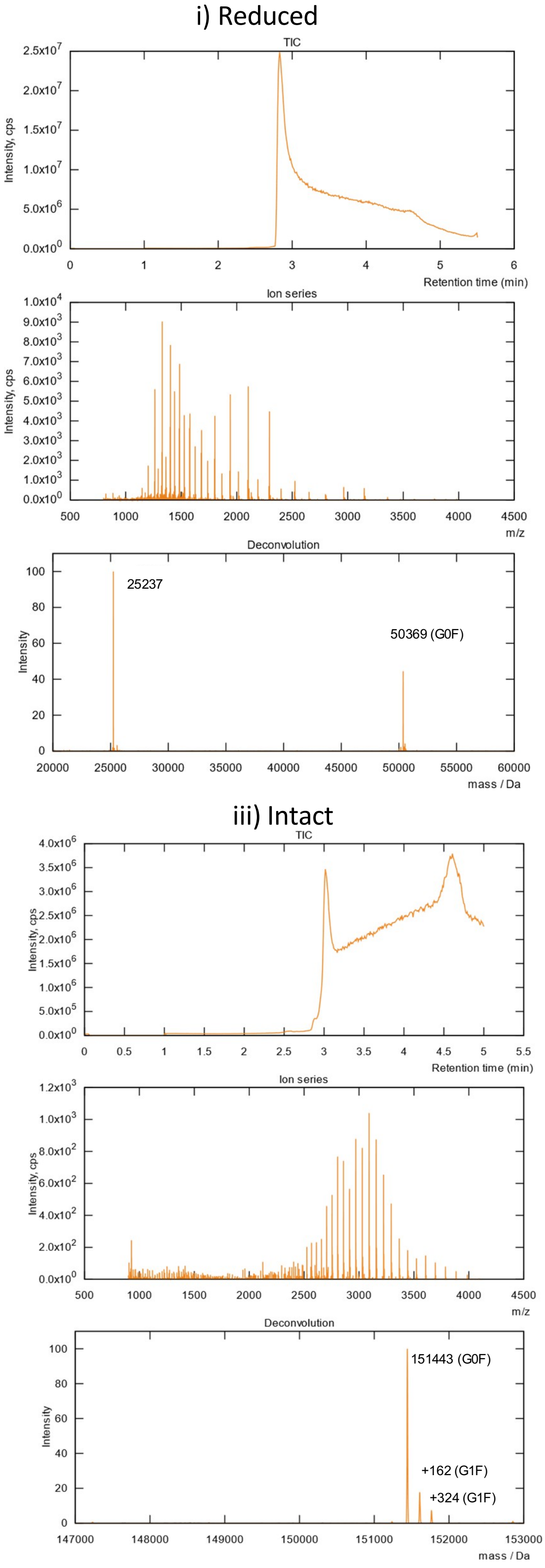

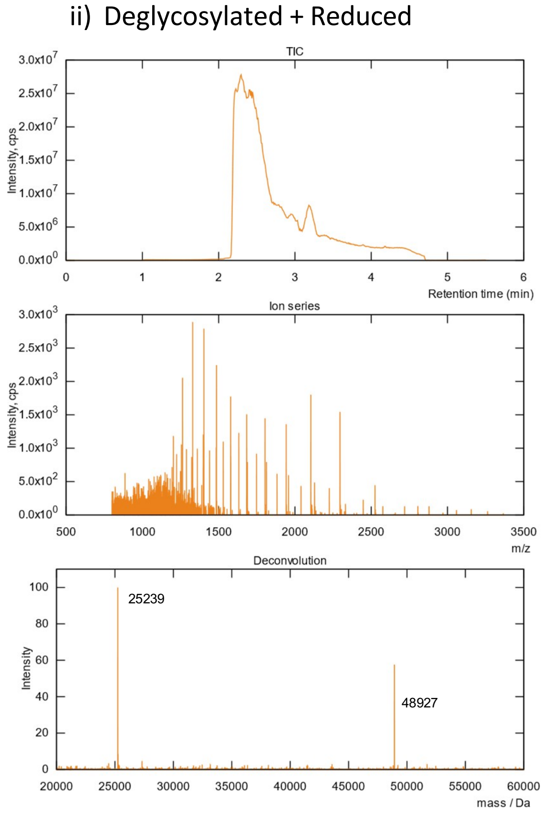

Ref.	Expected mass (Da)	Observed mass (Da)
i	LC: 25238<br>HC: 50367	LC: 25237<br>HC: 50369
ii	LC: 25238<br>HC: 48922	LC: 25239<br>HC: 48927
iii	151440	151443

Figure S11. UV chromatograms, ion series and deconvoluted mass spectra obtained from LC-MS analysis of antiCD33-(FLAG-Cys) under reducing (i), deglycosylated and reducing (ii) and intact (iii) conditions.

i) ii)

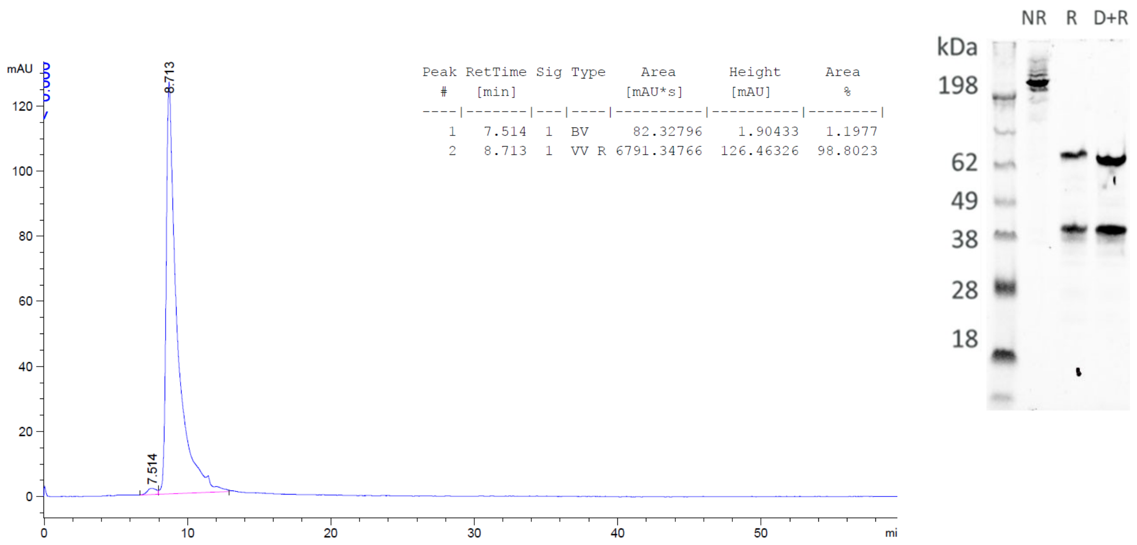

Figure S12. Biophysical analysis of antiCD33-(FLAG-Cys). i) UV chromatogram obtained from HP- SEC analysis, ii) Image of SDS-PAGE gel stained with Coomassie (NR: non-reducing, R: reducing, D+R: deglycosylated and reducing).

7.2.4 AntiCD33-(FLAG-Cys) - Negative control

Incubation of CPO-Alkyne with antiCD33-(FLAG-Cys) was performed following method "CPO conjugation" described in S6.5 using 2 mL of protein solution. No conjugation was observed.

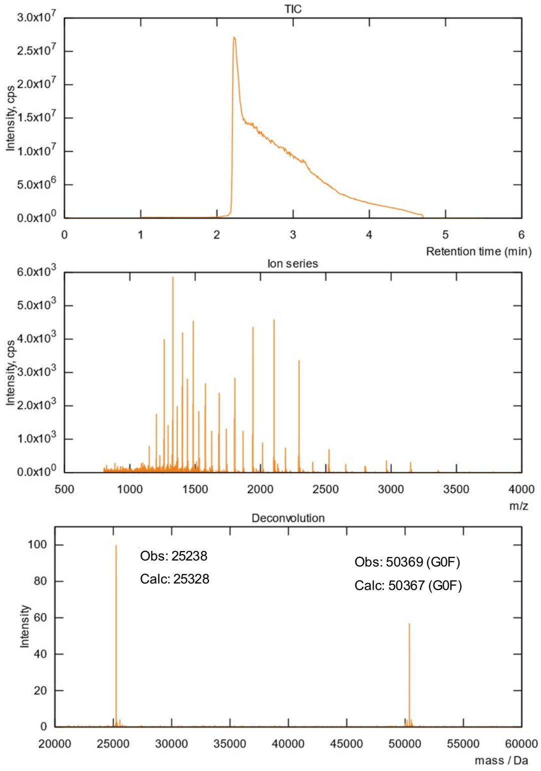

Figure S13. UV chromatograms, ion series and deconvoluted mass spectra obtained from LC-MS analysis of antiCD33-(FLAG-Cys) following incubation with CPO-Alkyne.

7.2.5 AntiCD33-(Cys)

The FLAG tag of AntiCD33-(FLAG-Cys) was removed following method "FLAG cleavage", described in S6.2. The subsequent reaction mixture was desalted using an Amicon® Ultra 0.5 mL Centrifugal Filter (10K MWCO). The purified protein, termed antiCD33-(Cys), was characterised by LC-MS, which confirmed successful cleavage of the FLAG tag and formation of an N-terminal cysteine.

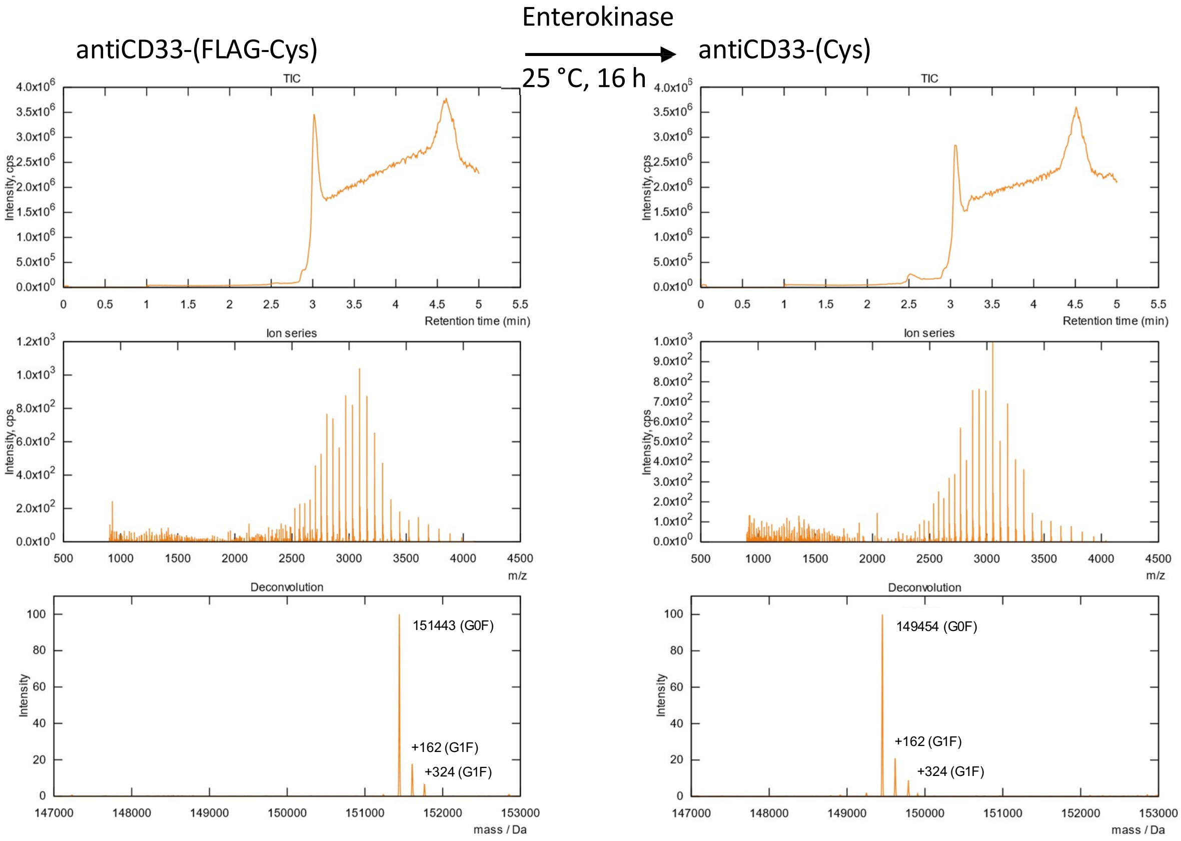

	Mass before Reaction (Da)	Mass after Reaction (Da)	Mass change (Da)
Expected	151440	149450	1990
Observed	151443	149454	1989

Figure S14. UV chromatograms, ion series and deconvoluted mass spectra obtained from LC-MS analysis of antiCD33-(FLAG-Cys) before and after modification.

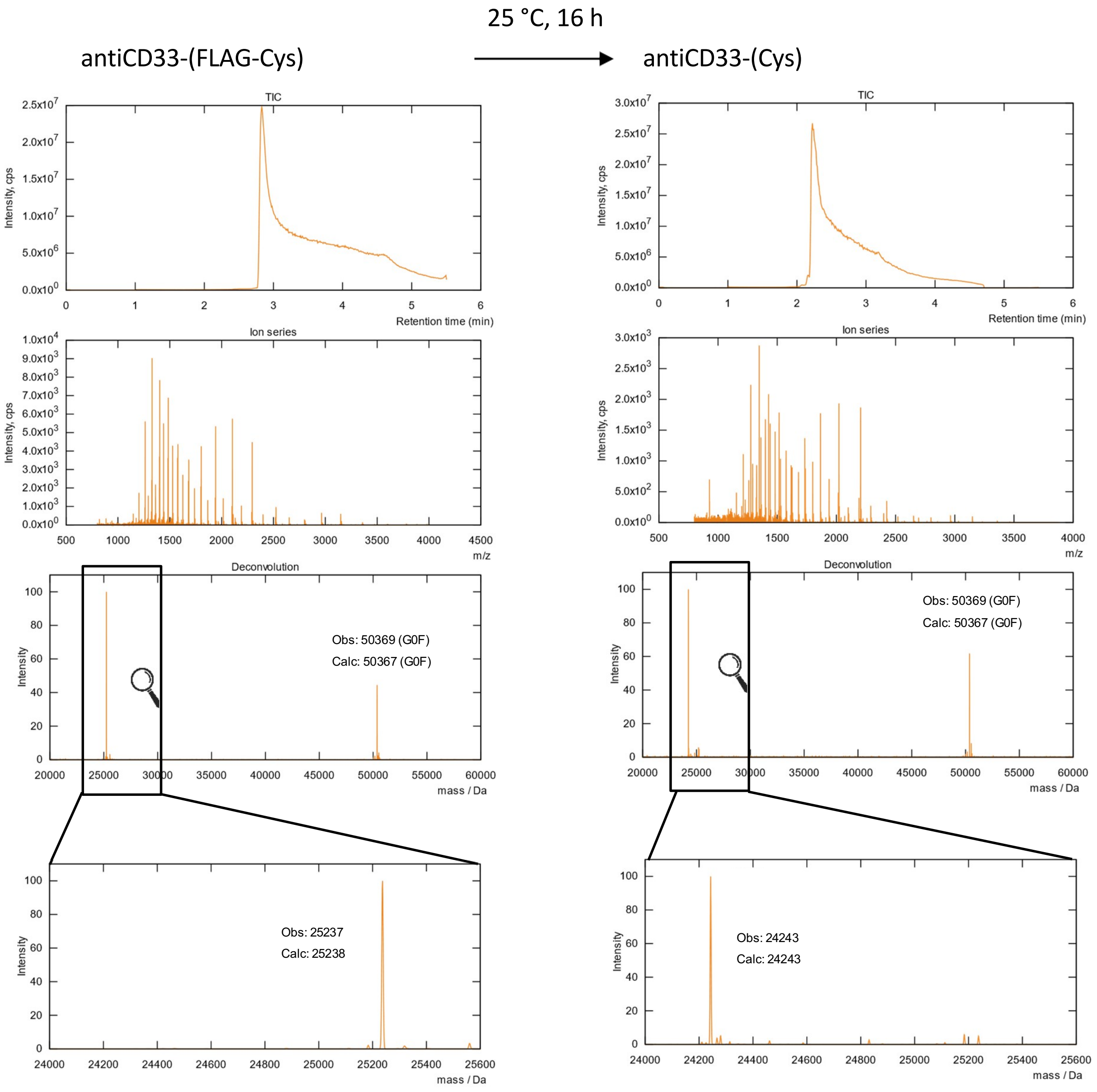

		Mass before Reaction (Da)	Mass after Reaction (Da)	Mass change (Da)
LC	Expected	25238	24243	-995
	Observed	25237	24243	-995
HC	Expected	50367	50367	0
	Observed	50369	50369	0

Figure S15. UV chromatograms, ion series and deconvoluted mass spectra obtained from LC-MS analysis of antiCD33-(FLAG-Cys) before and after modification.

7.2.6 AntiCD33-(CPO-Alkyne)

Conjugation of CPO-Alkyne to antiCD33-(Cys) was performed following method "CPO conjugation" described in S6.5 using 2 mL of protein solution. The reaction mixture was purified via SEC (94% protein recovery). LC-MS and biophysical analysis were conducted to assess the identity, integrity and functionality of the product, which was termed antiCD33-(CPO-Alkyne). The stability of this construct was assessed following method "stability study" described in 6.2.7.

antiCD33-(Cys) —(CPO-Alkyne, DTT; 25 °C, 4 h)→ antiCD33-(CPO-Alkyne)

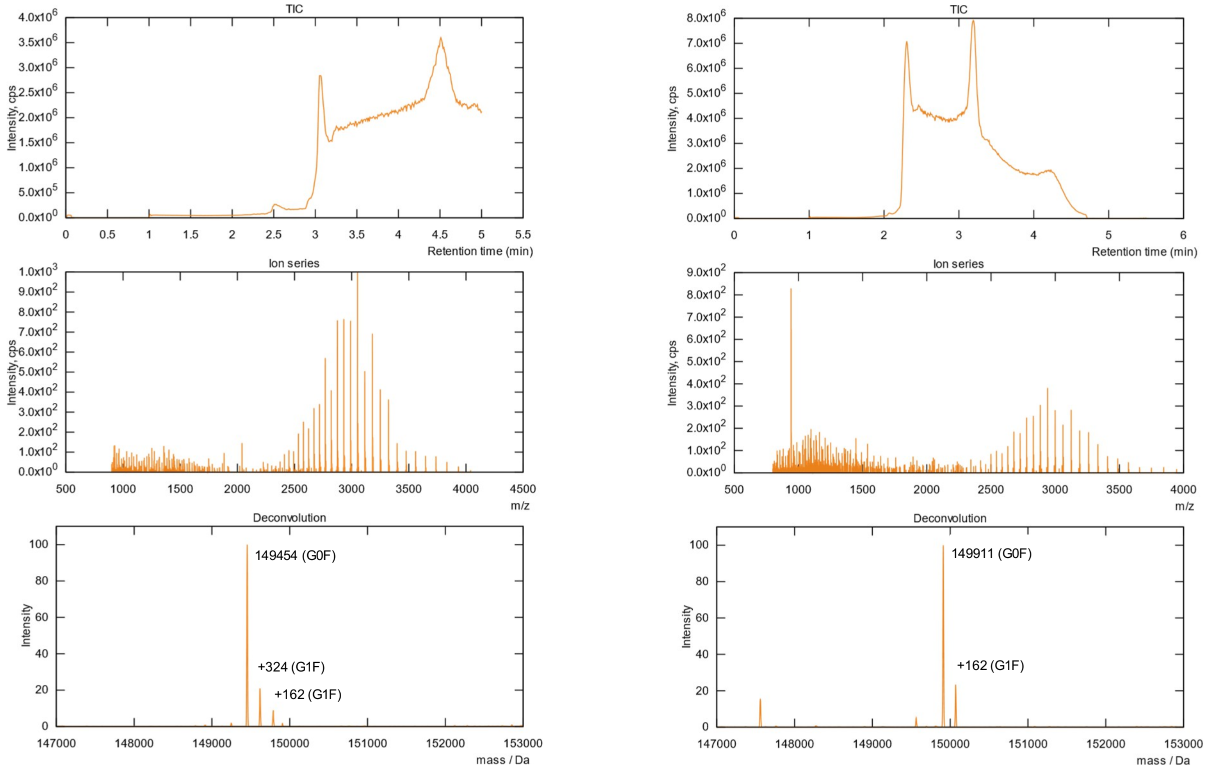

	Mass before Reaction (Da)	Mass after Reaction (Da)	Mass change (Da)
Expected	149450	149918	468
Observed	149454	149911	457

Figure S16. UV chromatograms, ion series and deconvoluted mass spectra obtained from LC-MS analysis of antiCD33-(Cys) before and after modification.

CPO-Alkyne

antiCD33-(Cys) → antiCD33-(CPO-Alkyne)

25 °C, 4 h

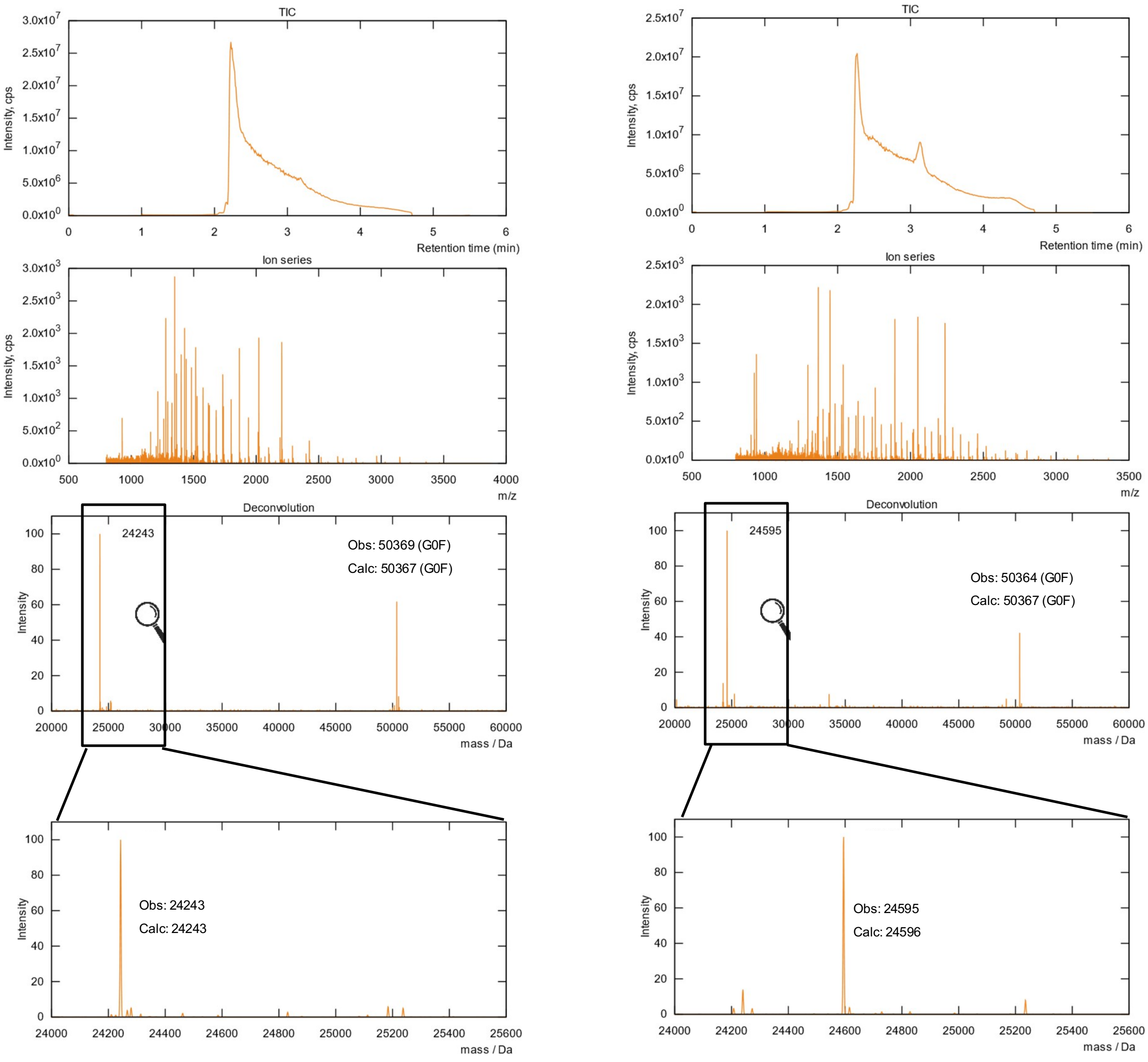

		Mass before reaction (Da)	Mass after reaction (Da)	Mass change (Da)
LC	Expected	24243	24596	353
	Observed	24243	24595	352
HC	Expected	50367	50367	0
	Observed	50369	50364	-5

Figure S17. UV chromatograms, ion series and deconvoluted mass spectra obtained from LC-MS analysis of antiCD33-(Cys) before and after modification.

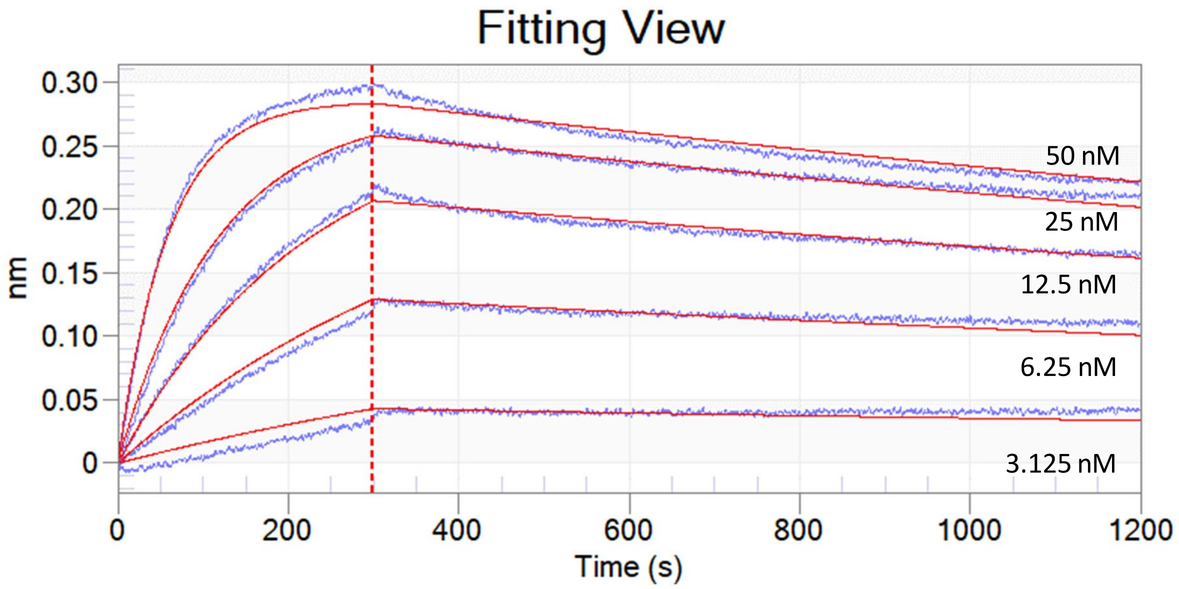

Ref.	K_D (M)	K_D Fitting Error (M)	K_{on} (1/Ms)	K_{on} error (1/Ms)	K_{dis} (1/s)	K_{dis} error (1/s)
antiCD33-(CPO-Alkyne)	8.22E-10	5.90E-12	3.33E+05	1.57E+03	2.74E-04	1.49E-06
antiCD33-(wildtype)	8.68E-10	4.97E-12	2.63E+05	9.12E+02	2.28E-04	1.04E-06

Figure S18. Biophysical analysis of antiCD33-(CPO-Alkyne). Binding curves and corresponding kinetic parameters obtained from BLI analysis.

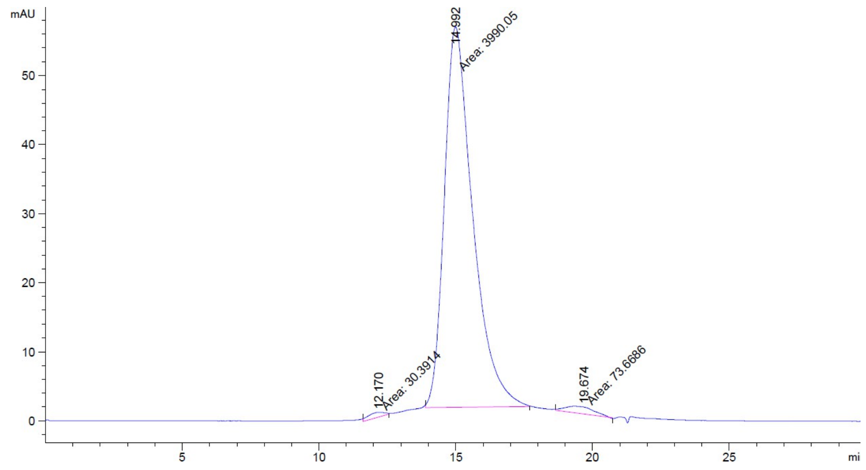

Figure S19. UV chromatogram from HP-SEC analysis of antiCD33-(CPO-Alkyne) following SEC purification.

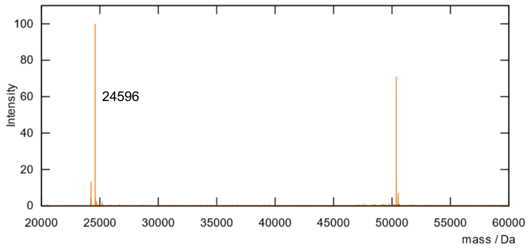

Initial
AntiCD33-(CPO-Alkyne)
LC: Calc.: 24596, Obs: 24596 (Relative intensity = 100%)
HC: Calc.: 50367, Obs: 50368
AntiCD33-(Cys)
LC: Calc.: 24243, Obs: 24242 (Relative intensity = 13%)
HC: Calc.: 50367, Obs: 50368

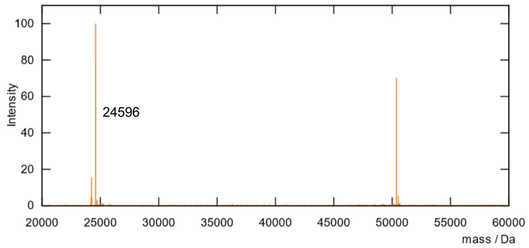

Day 2 Replicate 1
AntiCD33-(CPO-Alkyne)
LC: Calc.: 24596, Obs: 24596 (Relative intensity = 100%)
HC: Calc.: 50367, Obs: 50368
AntiCD33-(Cys)
LC: Calc.: 24243, Obs: 24242 (Relative intensity = 16%)
HC: Calc.: 50367, Obs: 50368

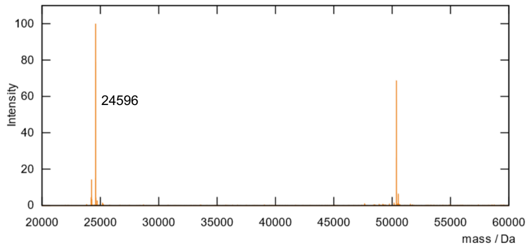

Day 2 Replicate 2
AntiCD33-(CPO-Alkyne)
LC: Calc.: 24596, Obs: 24596 (Relative intensity = 100%)
HC: Calc.: 50367, Obs: 50368
AntiCD33-(Cys)
LC: Calc.: 24243, Obs 24242 (Relative intensity = 14%)
HC: Calc.: 50367, Obs: 50368

Figure S20. Deconvoluted mass spectra of AntiCD33-(CPO-Alkyne) before and after glutathione incubation.

7.2.7 AntiCD33-(CPO-DBCO)

CPO-DBCO was conjugated to antiCD33-(Cys) following the “CPO conjugation” method described in S6.5 using 1 mL of protein solution. The resulting product, termed antiCD33-(CPO-DBCO), was desalted and analysed by LC-MS and BLI.

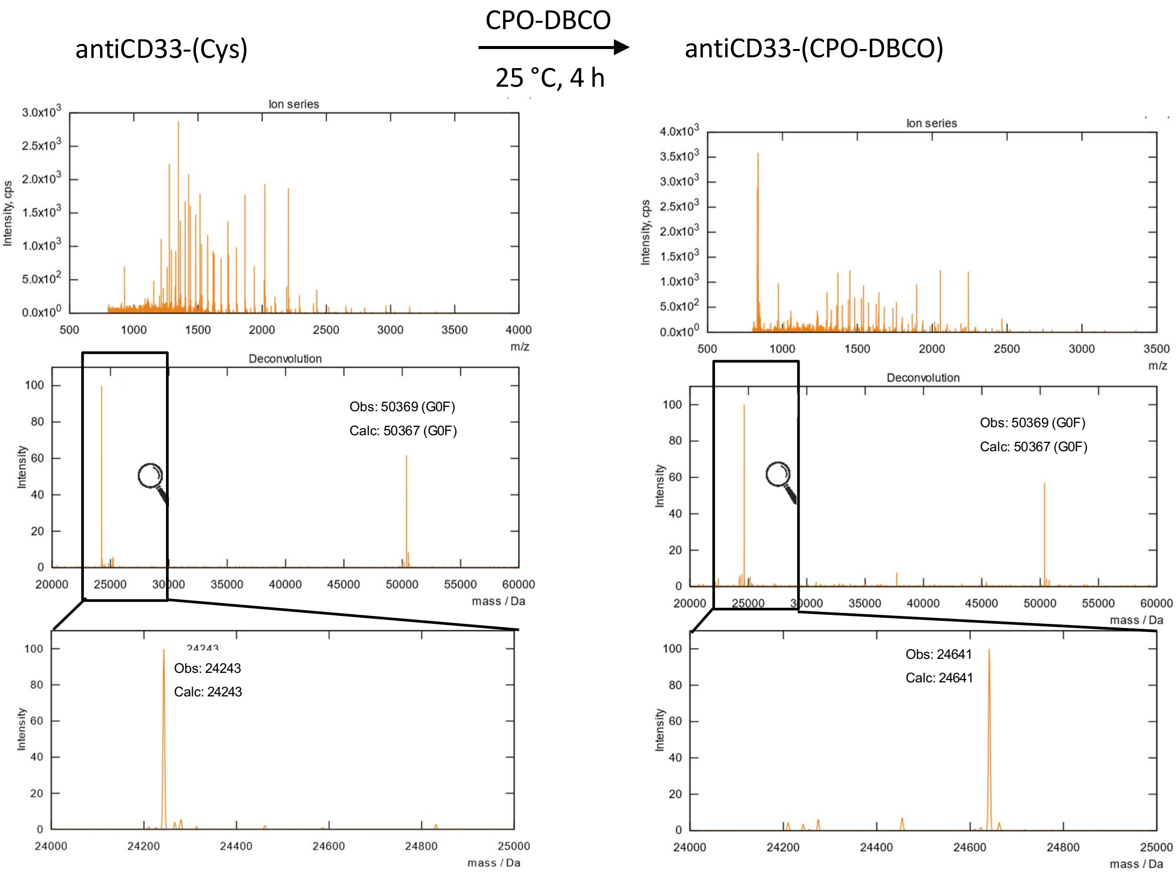

		Mass before Reaction (Da)	Mass after Reaction (Da)	Mass change (Da)
LC	Expected	24243	24641	398
	Observed	24243	24641	398
HC	Expected	50367	50367	0
	Observed	50369	50369	0

Figure S21. UV chromatograms, ion series and deconvoluted mass spectra obtained from LC-MS analysis of antiCD33-(Cys) before and after modification.

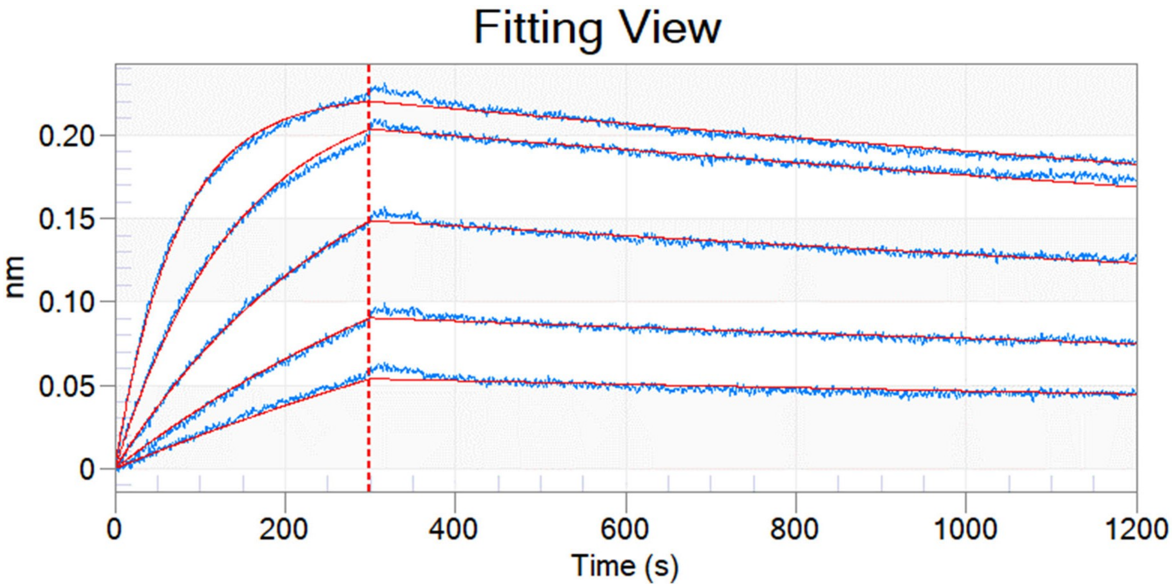

Ref.	K_D (M)	K_D Fitting Error (M)	K_{on} (1/Ms)	K_{on} error (1/Ms)	K_{dis} (1/s)	K_{dis} error (1/s)
antiCD33-(CPO-DBCO)	7.69E-10	3.85E-12	2.71E+05	7.74E+02	2.08E-04	8.56E-07
antiCD33-(wildtype)	8.68E-10	4.97E-12	2.63E+05	9.12E+02	2.28E-04	1.04E-06

Figure S22. Binding curves and corresponding kinetic parameters obtained from BLI analysis of antiCD33-(CPO-DBCO).

7.2.8 AntiCD33-(CPO-AF555)

AzAF555 was conjugated to AntiCD33-(CPO-DBCO) following the “SPAAC conjugation” method described in S6.9 using 1 mL of protein solution. LC-MS and biophysical analysis were conducted to assess the identity, integrity and functionality of the product, which was termed antiCD33-(CPO-AF555).

antiCD33-(CPO-DBCO) —(AzAF555; 25 °C, 1 h)→ antiCD33-(CPO-AF555)

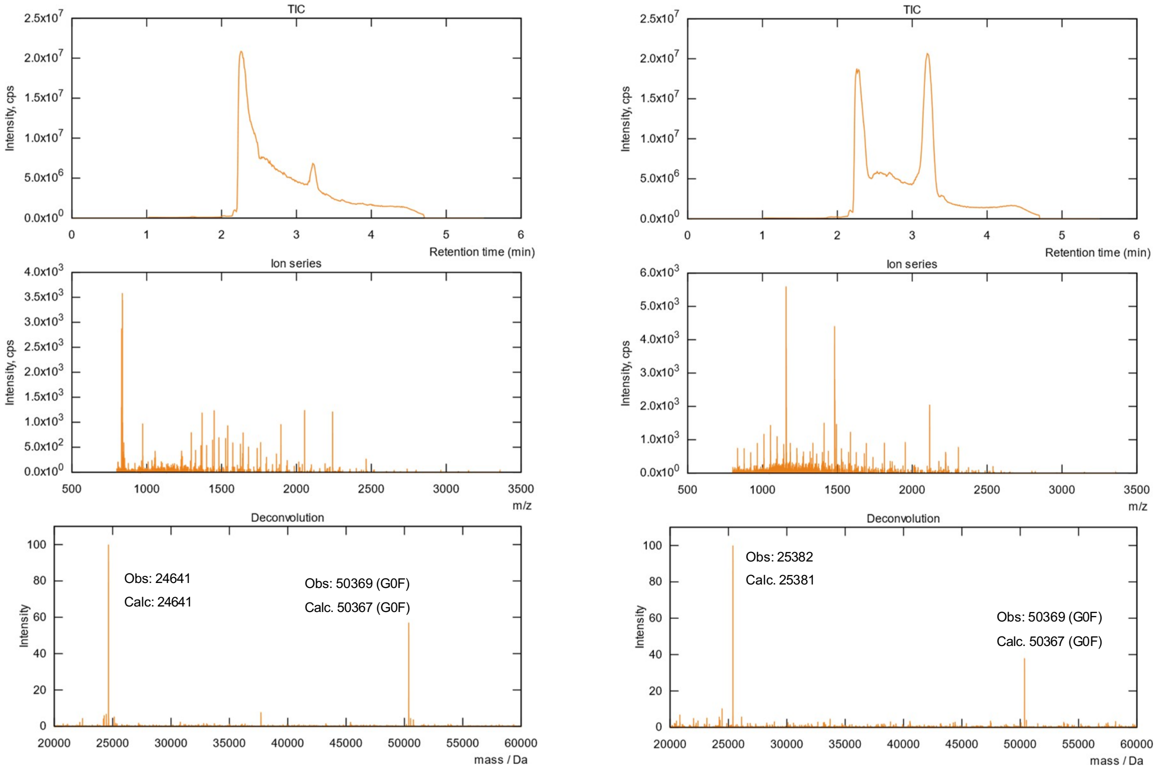

		Mass before Reaction (Da)	Mass after Reaction (Da)	Mass change (Da)
LC	Expected	24641	25381	+740
	Observed	24641	25382	+741
HC	Expected	50367	50367	0
	Observed	50369	50369	0

Figure S23. UV chromatograms, ion series and deconvoluted mass spectra obtained from LC-MS analysis of antiCD33-(CPO-DBCO) before and after modification.

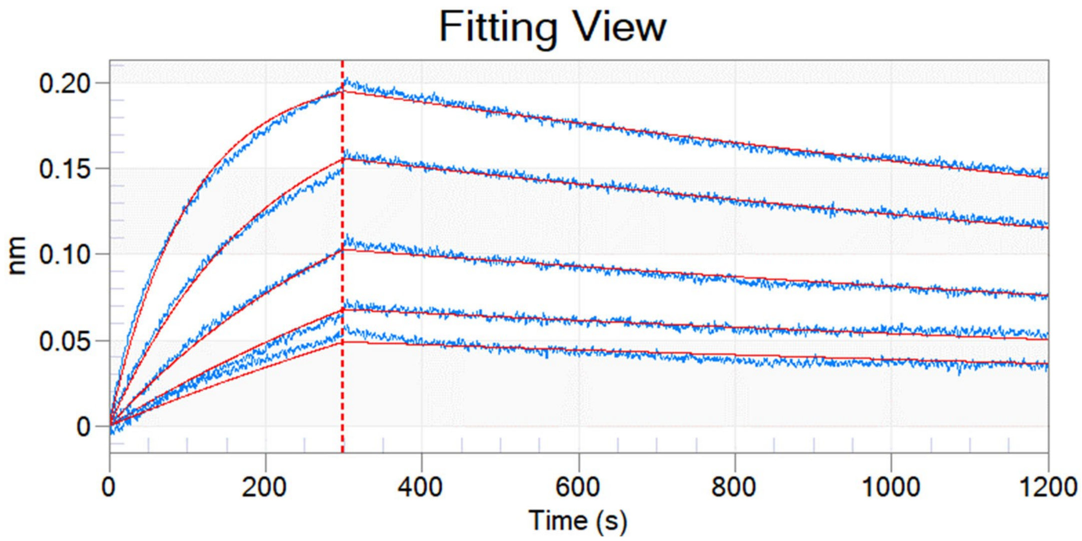

Ref.	K_D (M)	K_D Fitting Error (M)	Kon (1/Ms)	Kon error (1/Ms)	Kdis (1/s)	Kdis error (1/s)
antiCD33-(CPO-AF555)	1.77E-09	1.04E-11	1.89E+05	8.41E+02	3.34E-04	1.27E-06
antiCD33-(wildtype)	8.68E-10	4.97E-12	2.63E+05	9.12E+02	2.28E-04	1.04E-06

Figure S24. Binding curves and corresponding kinetic parameters obtained from BLI analysis of antiCD33-(CPO-AF555).

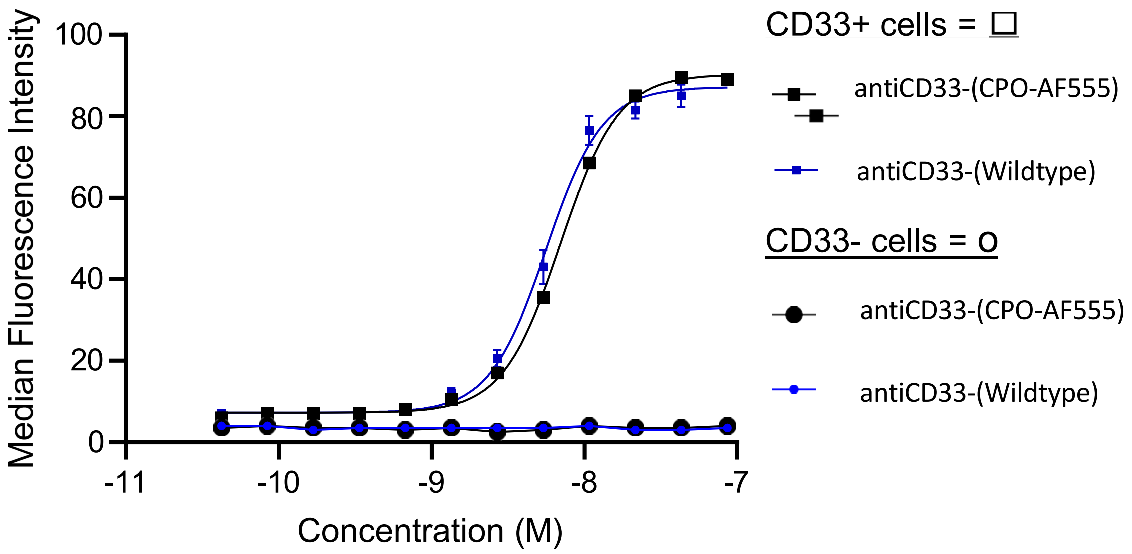

	EC_{50} (nM)	
	CD33-	CD33+
antiCD33-(Wildtype)	N/A	5.612 ± 0.361
antiCD33-(CPO-AF555)	N/A	6.985 ± 0.049

Figure S25. Titrated FACS binding curve of antiCD33-(CPO-AF555).

7.2.9 GlySERIAS digestion

AntiCD33-(wildtype), antiCD33-(Cys) and antiCD33-(CPO-DBCO) were exposed to “GlySERIAS digestion” method described in S6.3 in separate Eppendorfs. LC-MS spectra before and after GlySERIAS incubation are given for each the three constructs below.

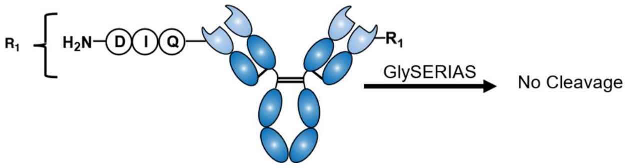

GlySERIAS

antiCD33-(Wildtype) → Reaction mixture post GlySERIAS incubation

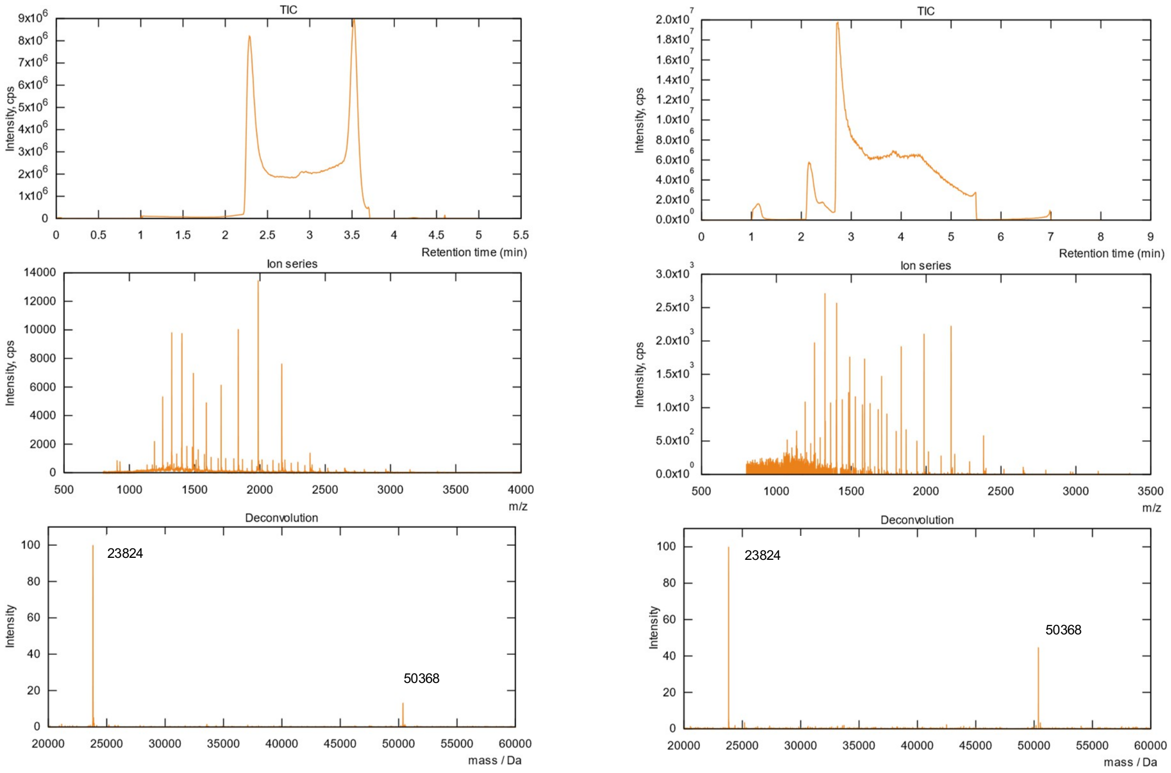

Figure S26. UV chromatograms, ion series and deconvoluted mass spectra obtained from LC-MS analysis of antiCD33-(Wildtype) before (left) and after (right) GlySERIAS incubation.

.

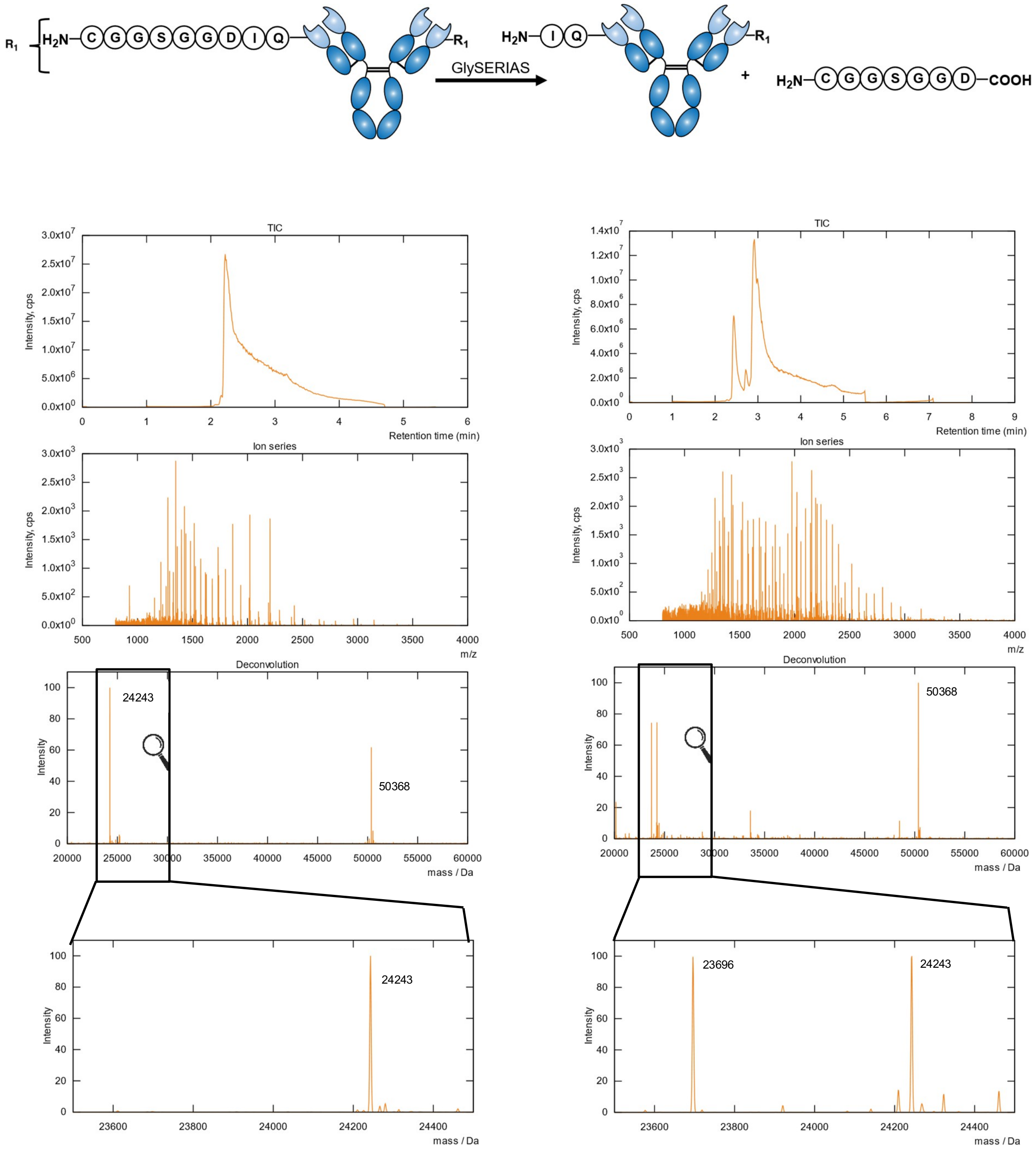

Figure S27. UV chromatograms, ion series and deconvoluted mass spectra obtained from LC-MS analysis of antiCD33-(Cys) before (left) and after (right) GlySERIAS incubation.

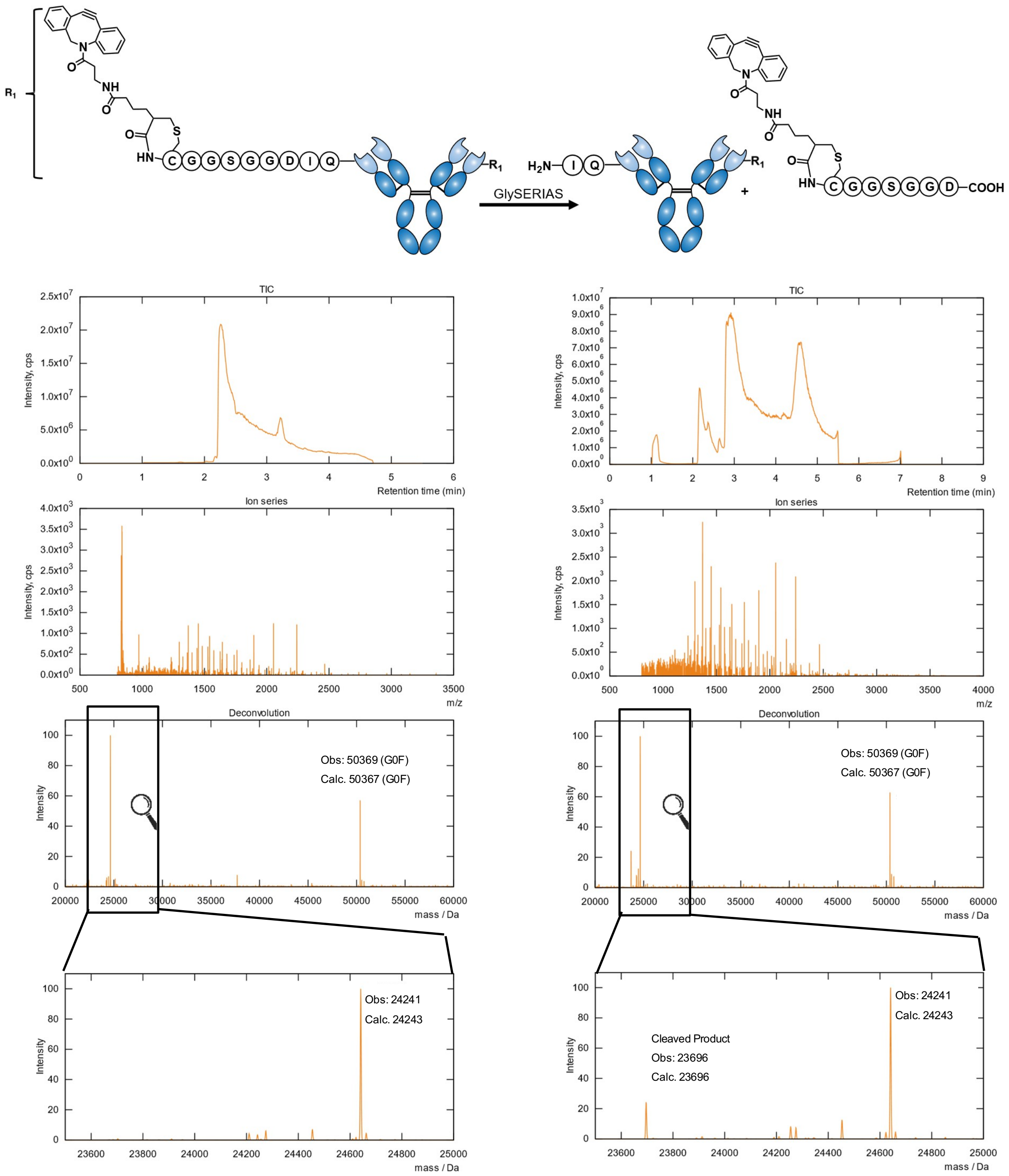

Figure S28. UV chromatograms, ion series and deconvoluted mass spectra obtained from LC-MS analysis of antiCD33-(CPO-DBCO) before (left) and after (right) GlySERIAS incubation.

7.3 π-Clamp

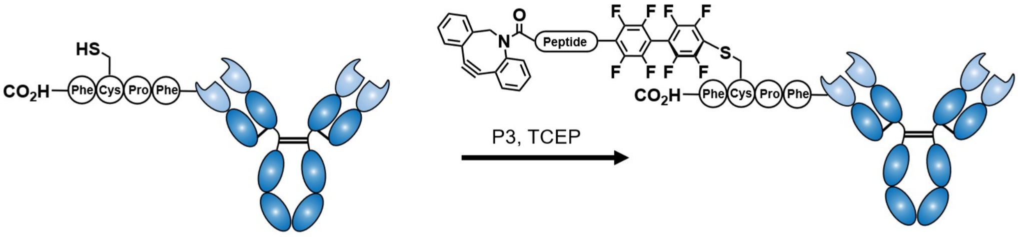

AntiCD33-(πClampNTerm-LC) AntiCD33-(P3NTerm-LC)

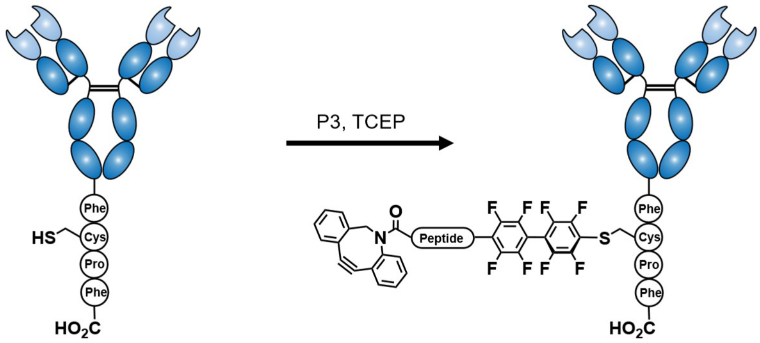

AntiCD33-(πClampCTerm-HC) AntiCD33-(P3CTerm-HC)

Figure S29. Schematic of the π-Clamp mediated modification protocol.

7.3.1 AntiCD33-(πClampNTerm-LC)

AntiCD33-(πClampNTerm-LC) was expressed and purified, following the protocol described in 6.2.4, achieving a post-purification yield of 21 mg L^{-1} (normalization factor from antiCD33-(Wildtype) parallel expression: 1.05, normalised yield: 22 mg L^{-1}). LC-MS and biophysical analysis were conducted to assess the identity and integrity of the product.

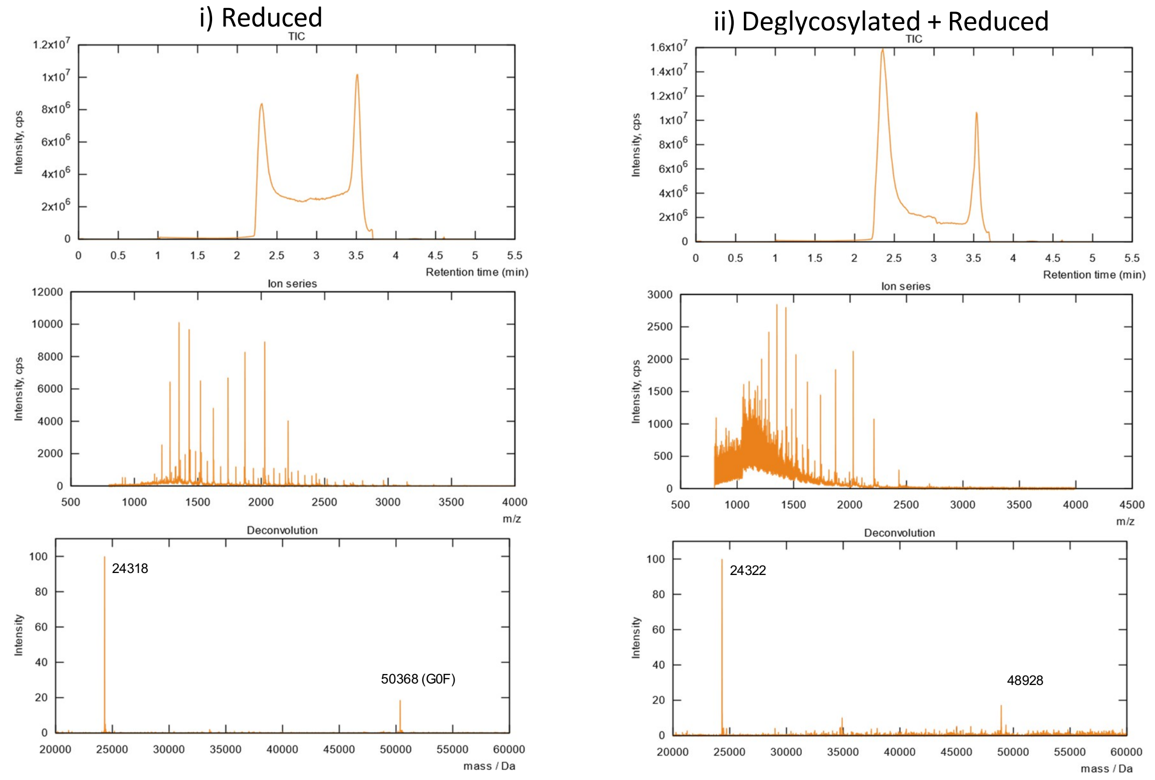

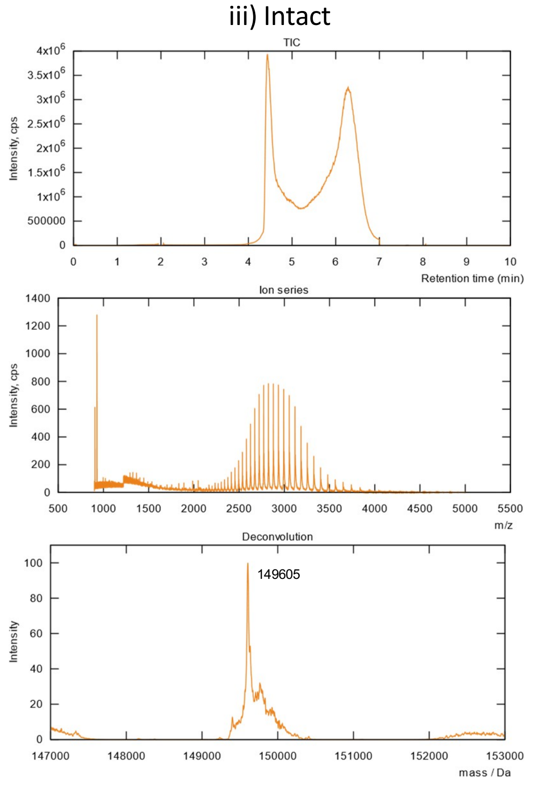

Ref.	Expected mass (Da)	Observed mass (Da)
A	LC: 24319<br>HC: 50367	LC: 24318<br>HC: 50368
B	LC: 24319<br>HC: 48922	LC: 24322<br>HC: 48928
C	149602	149605

Figure S30. UV chromatograms, ion series and deconvoluted mass spectra obtained from LC-MS analysis of antiCD33-(πClampNTerm-LC) under reduced (i), deglycosylated and reduced (ii) and intact (iii) conditions.

i) **ii)**

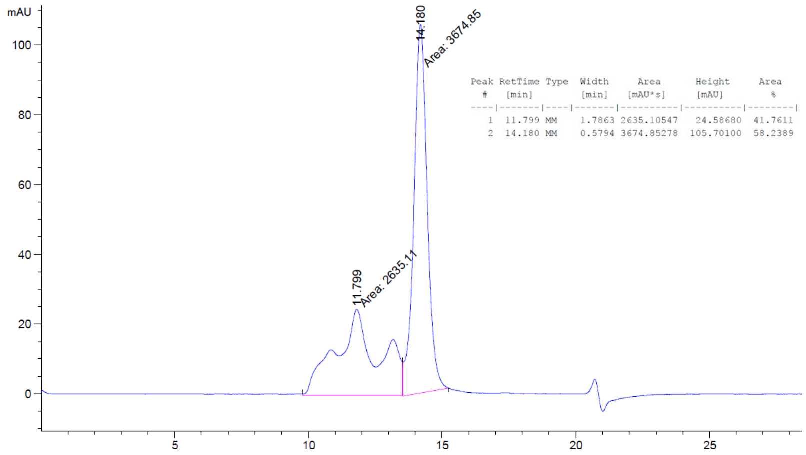

Peak #	RetTime [min]	Type	Width [min]	Area [mAU*s]	Height [mAU]	Area %
1	11.799	MM	1.7863	2635.10547	24.58680	41.7611
2	14.180	MM	0.5794	3674.85278	105.70100	58.2389

ii)

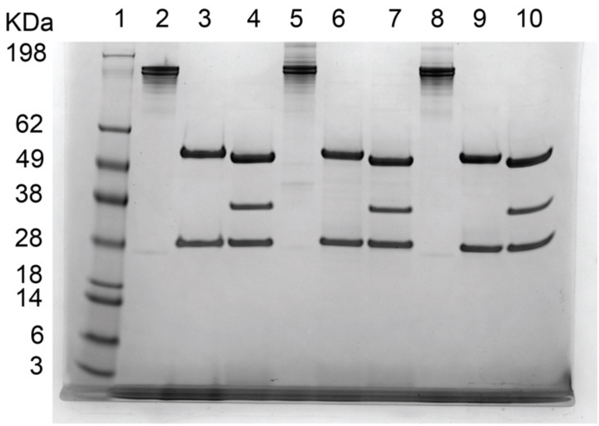

Figure S31. Biophysical analysis of antiCD33-(πClampNTerm-LC) (before SEC). i) UV chromatogram obtained from HP-SEC analysis, ii) Image of SDS-PAGE gel stained with Coomassie – lanes 8 to 10 (Lane 8 – NR: non-reducing, Lane 9 – R: reducing, Lane 10 –D+R: Deglycosylated and reducing).

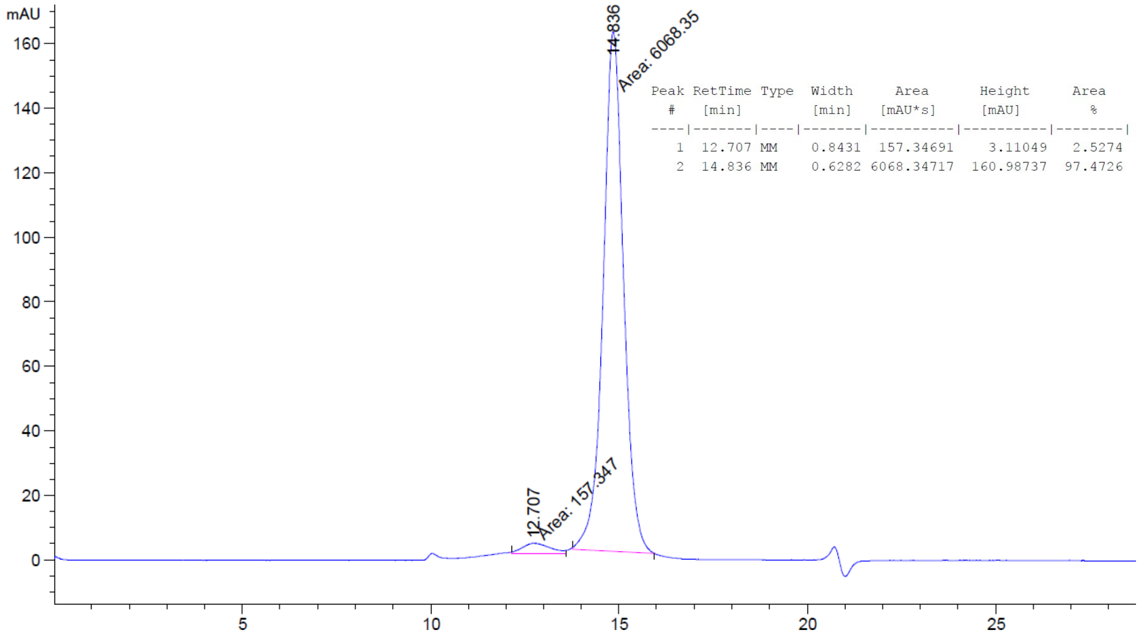

Figure S32. Biophysical analysis of antiCD33-(πClampNTerm-LC) (after SEC). i) UV chromatogram obtained from HP-SEC analysis.

7.3.2 AntiCD33-(πClampCTerm-HC)

AntiCD33-(πClampCTerm-HC) was expressed and purified, following the protocol described in 6.2.4, achieving a post-purification yield of 19 mg L^{-1} (normalization factor from antiCD33-(Wildtype) parallel expression: 0.89, normalised yield: 17 mg L^{-1}). LC-MS and biophysical analysis were conducted to assess the identity and integrity of the product.

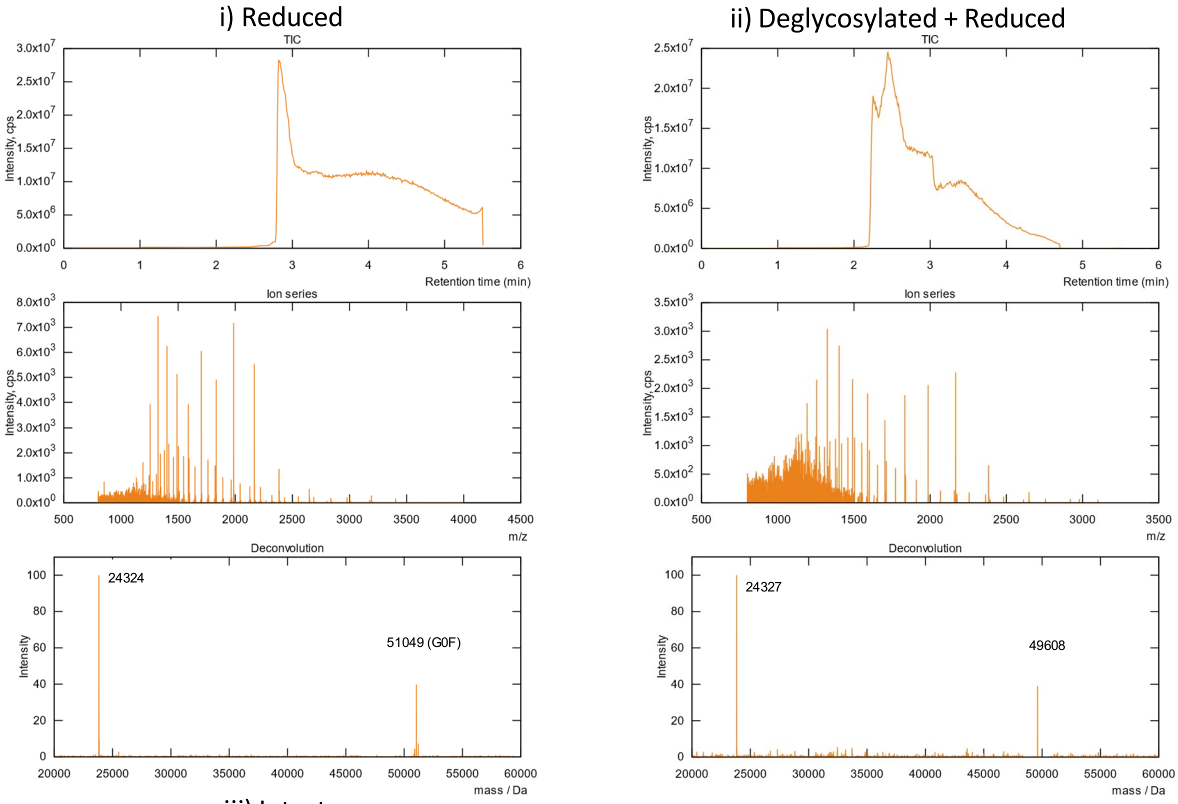

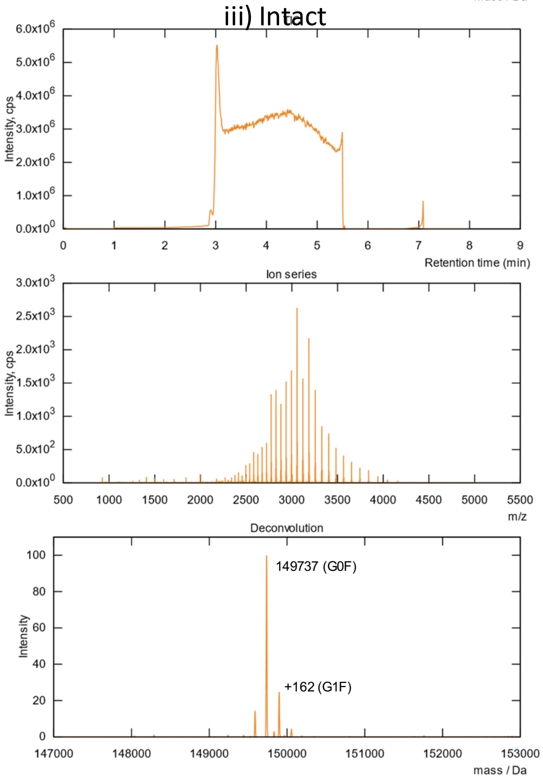

Ref.	Expected mass (Da)	Observed mass (Da)
A	LC: 23824<br>HC: 51047	LC: 24324<br>HC: 51049
B	LC: 23824<br>HC: 49602	LC: 24327<br>HC: 49608
C	149973	149737

Figure S33. UV chromatograms, ion series and deconvoluted mass spectra obtained from LC-MS analysis of antiCD33- (πClampCTerm-HC) under reduced (i), deglycosylated and reduced (ii) and intact (iii) conditions. Mass of intact suggests CTerm cysteines are disulfide bonded rather than cysteinylated.

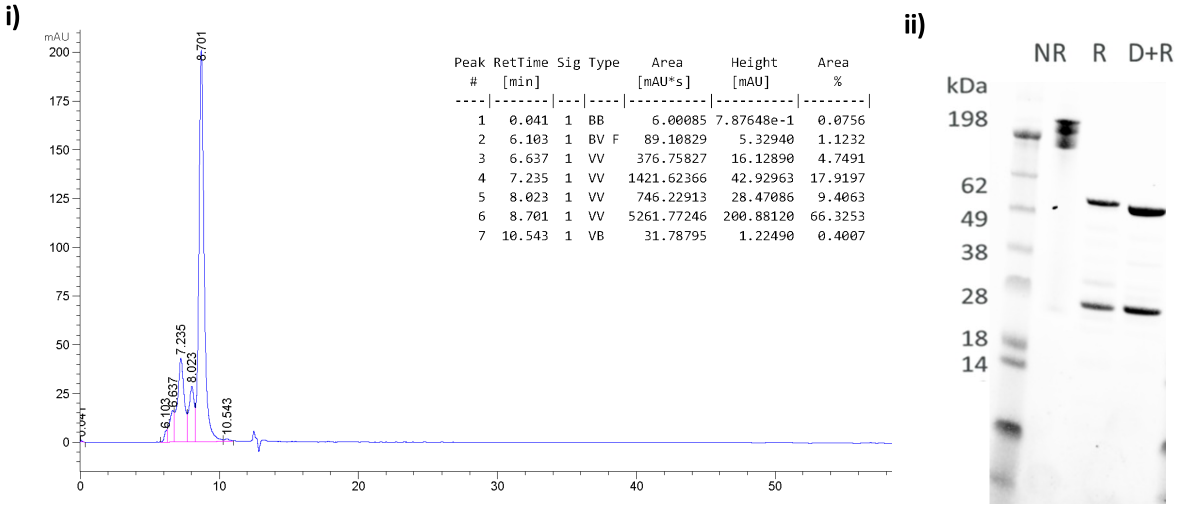

Peak #	RetTime [min]	Sig	Type	Area [mAU*s]	Height [mAU]	Area %
1	0.041	1	BB	6.00085	7.87648e-1	0.0756
2	6.103	1	BV F	89.10829	5.32940	1.1232
3	6.637	1	VV	376.75827	16.12890	4.7491
4	7.235	1	VV	1421.62366	42.92963	17.9197
5	8.023	1	VV	746.22913	28.47086	9.4063
6	8.701	1	VV	5261.77246	200.88120	66.3253
7	10.543	1	VB	31.78795	1.22490	0.4007

Figure S34. Biophysical analysis of antiCD33-(πClampNTerm-HC) (before SEC). i) UV chromatogram obtained from HP-SEC analysis, ii) Image of SDS-PAGE gel stained with Coomassie (NR: non-reducing, R: reducing, D+R: Deglycosylated and reducing).

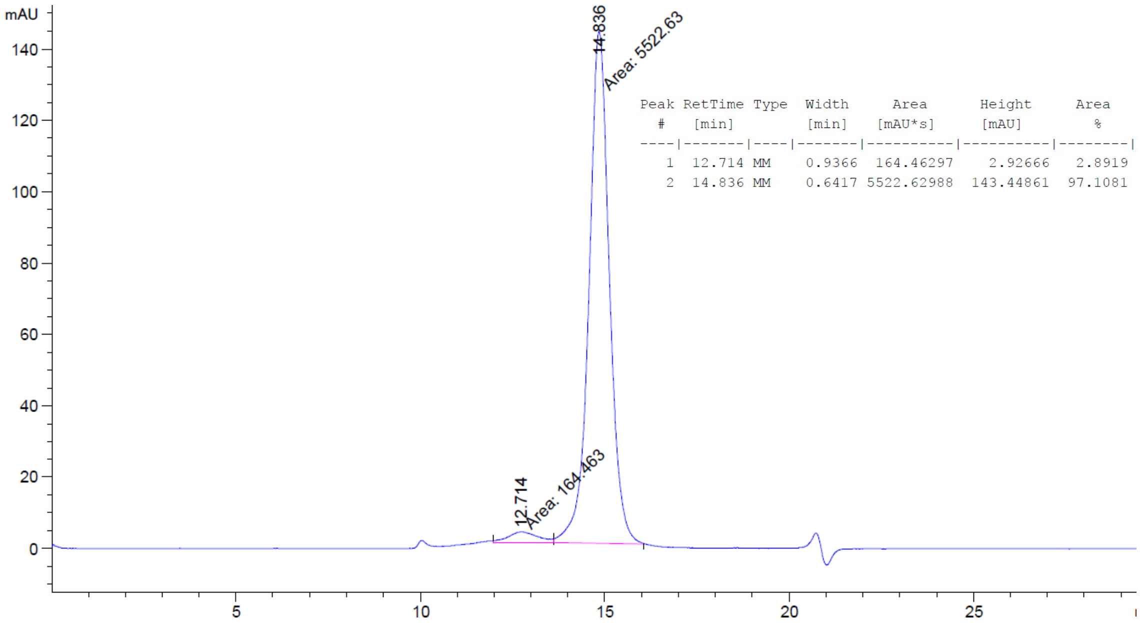

Peak #	RetTime [min]	Type	Width [min]	Area [mAU*s]	Height [mAU]	Area %
1	12.714	MM	0.9366	164.46297	2.92666	2.8919
2	14.836	MM	0.6417	5522.62988	143.44861	97.1081

Figure S35. Biophysical analysis of antiCD33-(πClampCTerm-HC) (after SEC). i) UV chromatogram obtained from HP-SEC analysis.

7.3.3 AntiCD33-(P3NTerm-LC) - Attempted

Conjugation of P3 to antiCD33-(πClampNTerm-LC) was attempted following method "π-clamp conjugation" described in S6.7. The resulting solution was then purified using Amicon® Ultra 0.5 mL Centrifugal Filters (10K MWCO).

antiCD33-(πClampNTerm-LC) —(P3; 37 °C, 6 h)→ No reaction

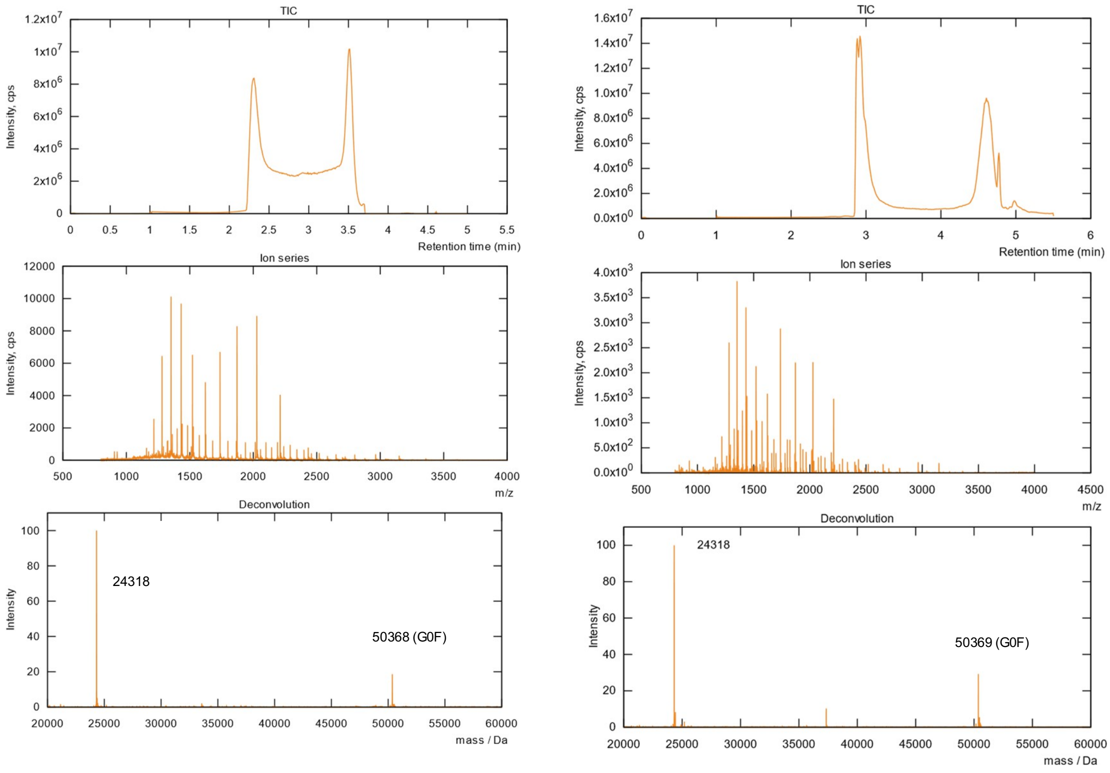

		Mass before reaction (Da)	Mass after reaction (Da)	Mass change (Da)
LC	Expected	24319	26110	+1791
	Observed	24318	24318	0
HC	Expected	50367	50367	0
	Observed	50368	50369	+1

Figure S36. UV chromatograms, ion series and deconvoluted mass spectra obtained from LC-MS analysis of antiCD33-(πClampNTerm-LC) before and after P3 incubation.

7.3.4 AntiCD33-(P3CTerm-HC)

Conjugation of P3 to antiCD33-(πClampCTerm-HC) was attempted following method "π-clamp conjugation" described in S6.7. The resulting solution was then purified using Amicon® Ultra 0.5 mL Centrifugal Filters (10K MWCO). The purified product, termed antiCD33-(P3CTerm-HC), was subjected to LC-MS analysis.

antiCD33-(πClampCTerm-HC) —(P3; 37 °C, 6 h)→ antiCD33-(P3CTerm-HC)

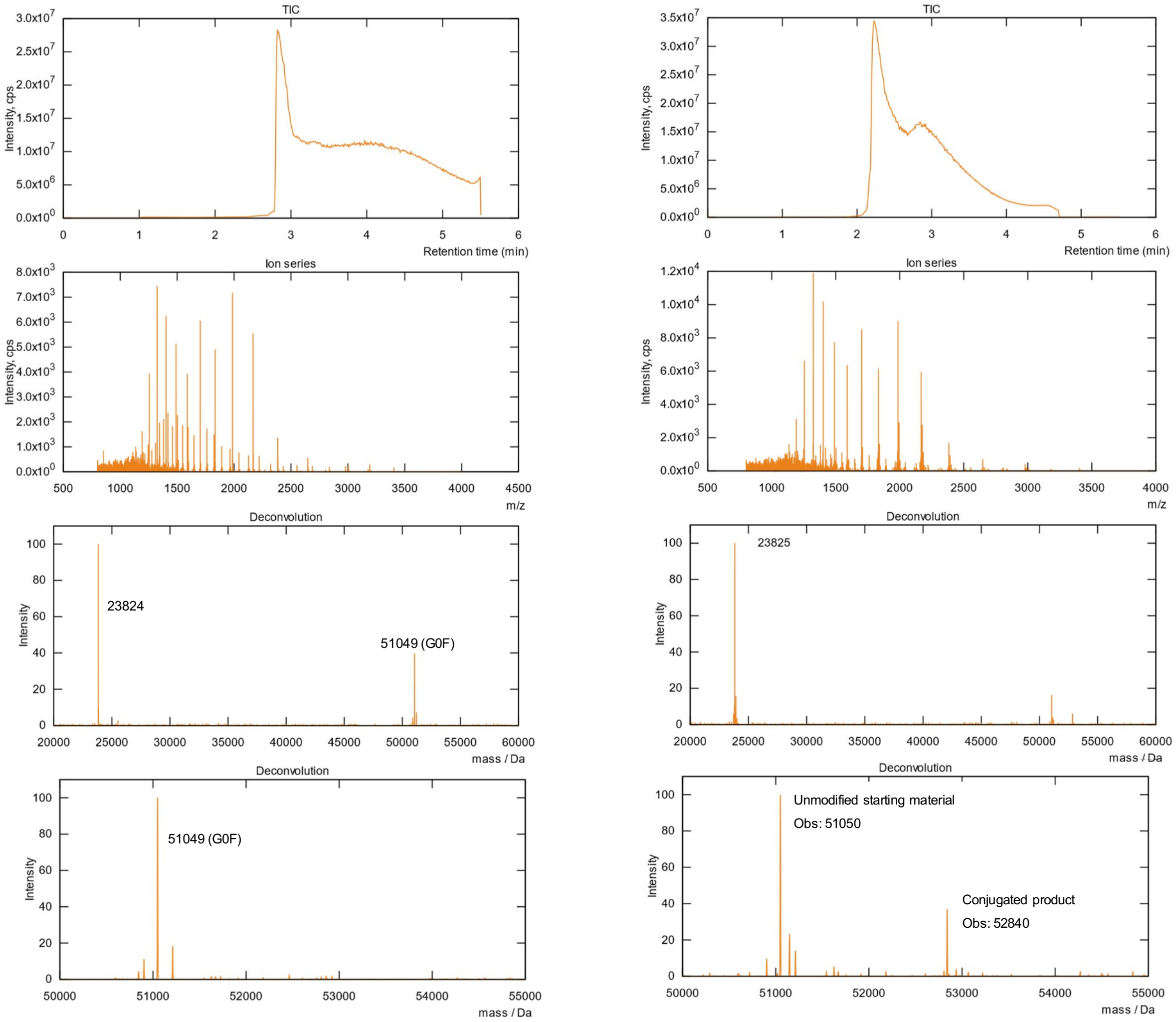

		Mass before reaction (Da)	Mass after reaction(Da)	Mass change (Da)
LC	Expected	23824	23824	0
	Observed	23824	23825	+1
HC	Expected	51047	52838	+1791
	Observed	51049	52840	+1791

Figure S37. UV chromatograms, ion series and deconvoluted mass spectra obtained from LC-MS analysis of antiCD33-(πClampCTerm-HC) before and after P3 incubation.

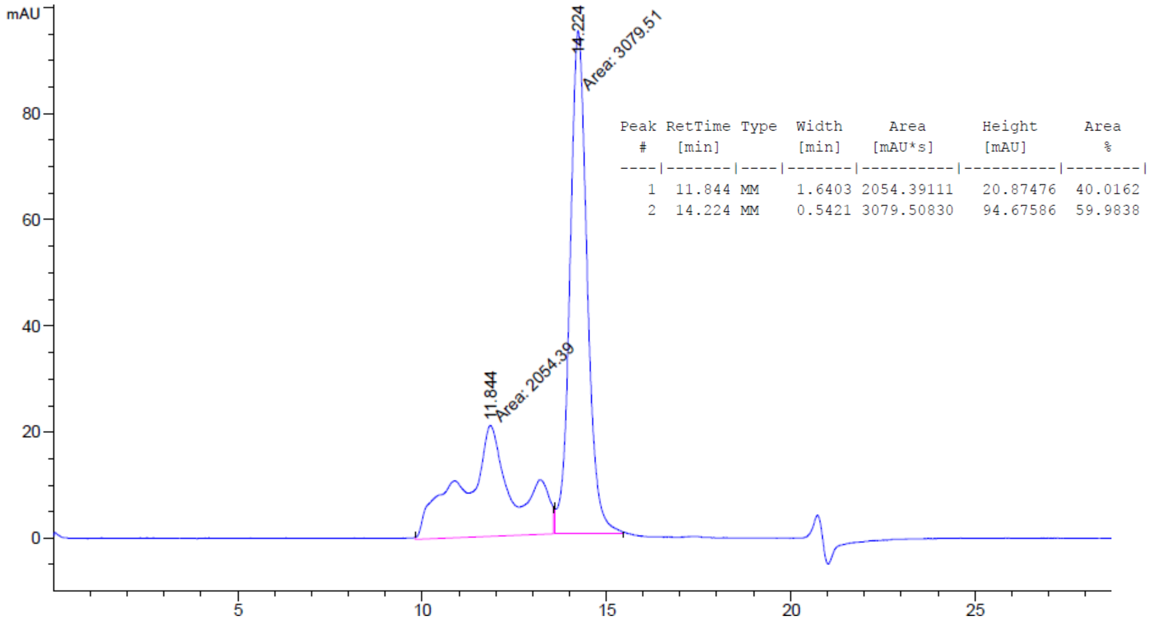

Figure S38. UV chromatogram obtained from HP-SEC analysis of antiCD33-(P3CTerm-HC).

7.4 Sortase

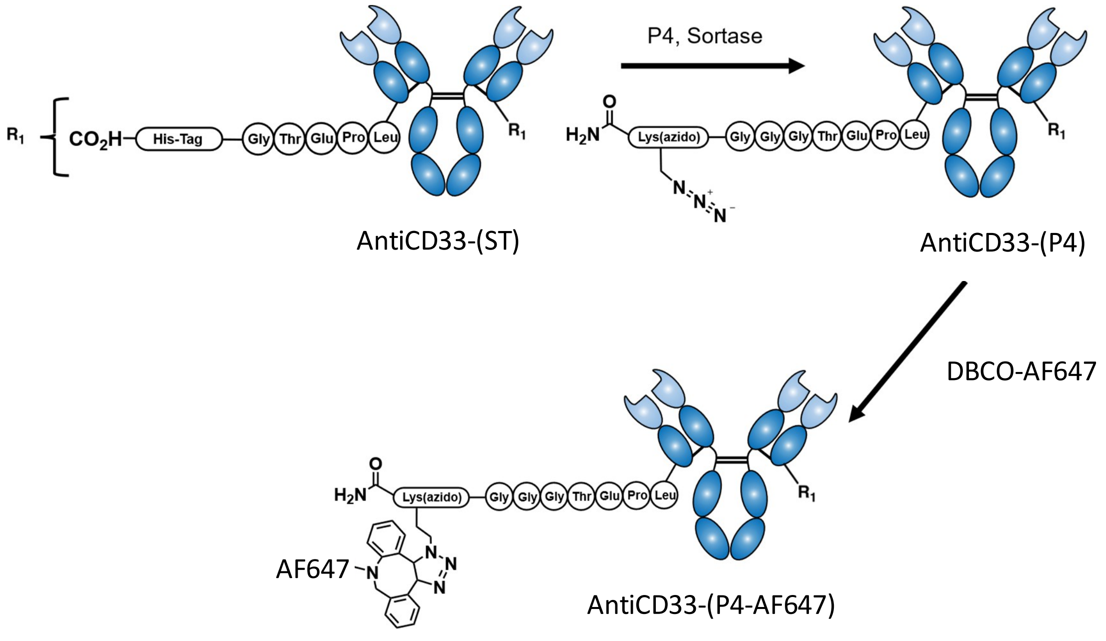

Figure S39. Schematic of the sortase-mediated modification protocol.

7.4.1 AntiCD33-(ST)

AntiCD33-(ST) was expressed and purified, following the protocol described in 6.2.4, achieving a post-purification yield of 29 mg L^{-1} (normalization factor from antiCD33-(Wildtype) parallel expression: 2.18, normalised yield: 63 mg L^{-1}). LC-MS and biophysical analysis were conducted to assess the identity and integrity of the product.

i) Reduced

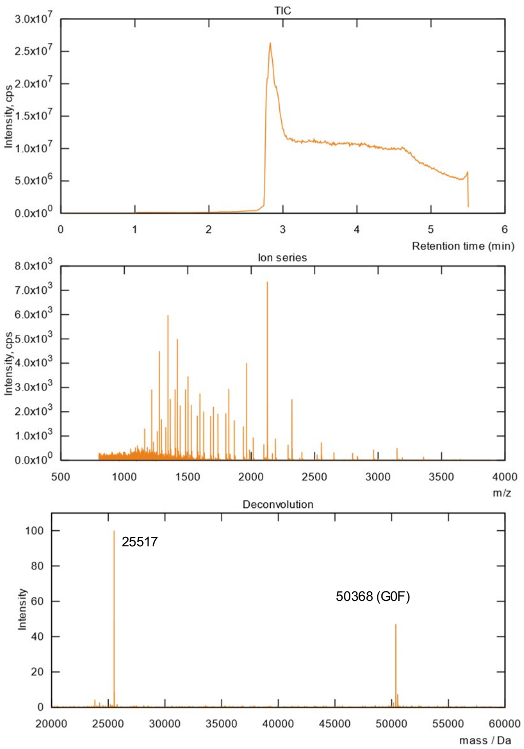

ii) Deglycosylated + Reduced

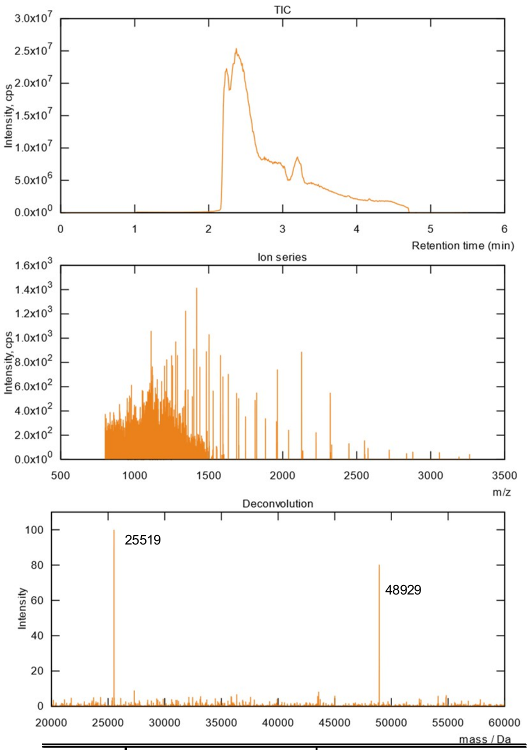

iii) Intact

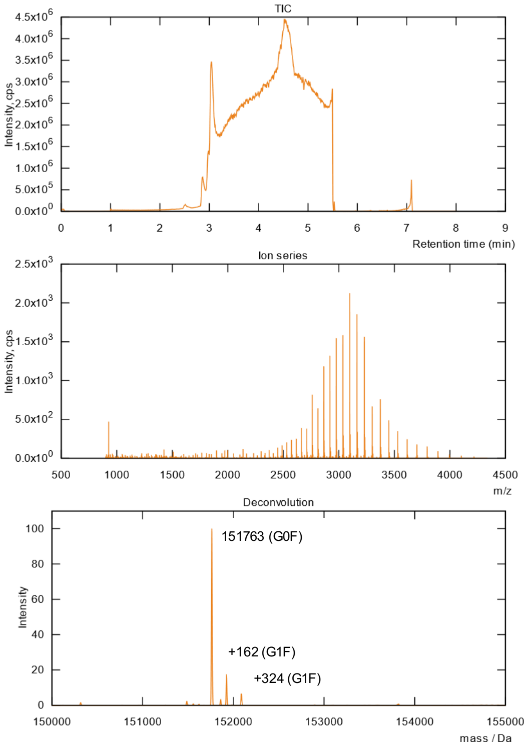

Ref.	Expected mass (Da)	Observed mass (Da)
i	LC: 25517<br>HC: 50367	LC: 25517<br>HC: 50368
ii	LC: 25517<br>HC: 48922	LC: 25519<br>HC: 48929
iii	151761	151763

Figure S40. UV chromatograms, ion series and deconvoluted mass spectra obtained from LC-MS analysis of antiCD33-(ST) under reduced (i), deglycosylated and reduced (ii) and intact (iii) conditions.

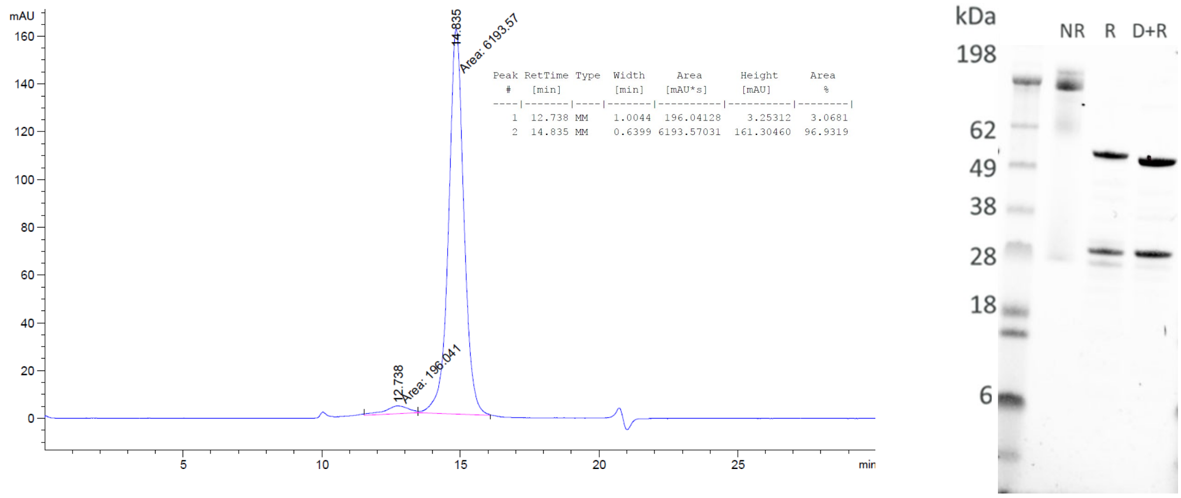

Figure S41. Biophysical analysis of antiCD33-(ST). i) UV chromatogram obtained from HP-SEC analysis, ii) Image of SDS-PAGE gel stained with Coomassie (NR: non-reducing, R: reducing, D+R: deglycosylated and reducing).

7.4.2 Optimisation of sortase-mediated conjugation of P4 to antiCD33-(ST)

AntiCD33-(ST) (50 mM Tris pH 7.5, 150 mM NaCl and 10 mM $CaCl_2$) was incubated with sortase and P4 in a 0.5 mL Eppendorf. With the total reaction volume fixed to 60 µL, 8 reactions were setup with varying concentrations of all three components, as described in Table 7.4.2-A. Each reaction was incubated at 37 °C, with aliquots taken after 2 h, 6 h and 24 h. Each aliquot was desalted using Amicon® Ultra 0.5 mL Centrifugal Filter (10K MWCO) before being subjected to LC-MS analysis. The expected masses of antiCD33-(ST) (starting material), antiCD33-(ST-cleaved) (sortase-cleaved intermediate), antiCD33-(P4) (desired product) and antiCD33-(reducedP4) (azide-reduced desired product) are given in Table 7.4.2-B. The conversion of antiCD33-(ST) to either antiCD33-(P4) or antiCD33-(reducedP4) for each reaction is given in Table 7.4.2-C.

Table 7.4.2-A: Summary of the reaction conditions assessed.

Protein concentration (µM)	Sortase Equiv.= 0.05		Sortase Equiv. = 0.5	
	Peptide equiv.		Peptide equiv.	
	5	50	5	50
5	R1	R2	R5	R6
100	R3	R4	R7	R8

Table 7.4.2-B: Summary of the expected masses for the starting material, intermediates and desired products of sortase-mediated conjugation.

Protein name	Substance	Symbol	Expected mass of light chain (Da)
antiCD33-(ST)	Starting material		25517
antiCD33-(ST-cleaved)	Sortase cleaved intermediate		24580
antiCD33-(P4)	Desired product		24903
antiCD33-(reducedP4)	Azide-reduced desired product		24877

Table 7.4.2-C: Summary of % conversions to desired product/azide-reduced desired product for reactions R1-R8.

	2 h	6 h	24 h
R1	33	35	22
R2	>90	>90	>90
R3	42	39	33
R4	>90	>90	>90
R5	27	21	21
R6	68	55	23
R7	0	0	0
R8	83	76	60

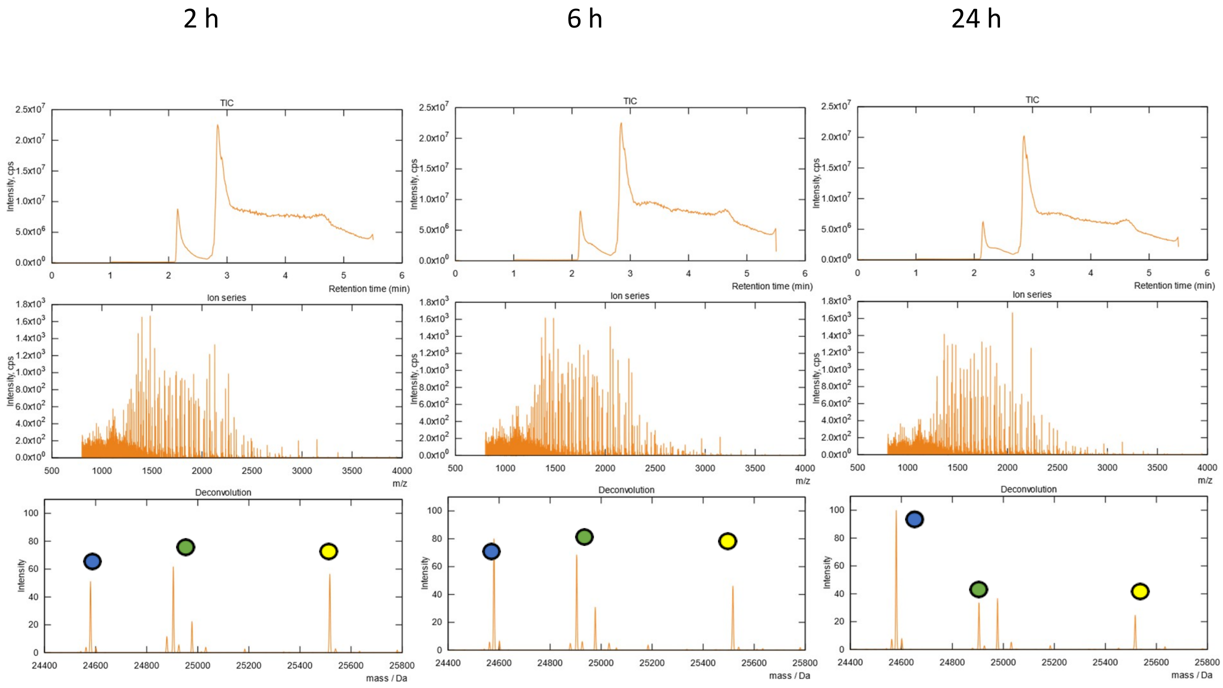

Figure S42. UV chromatograms, ion series and deconvoluted mass spectra obtained from LC-MS analysis of reaction mixture R1 after 2 h, 6 h and 24 h.

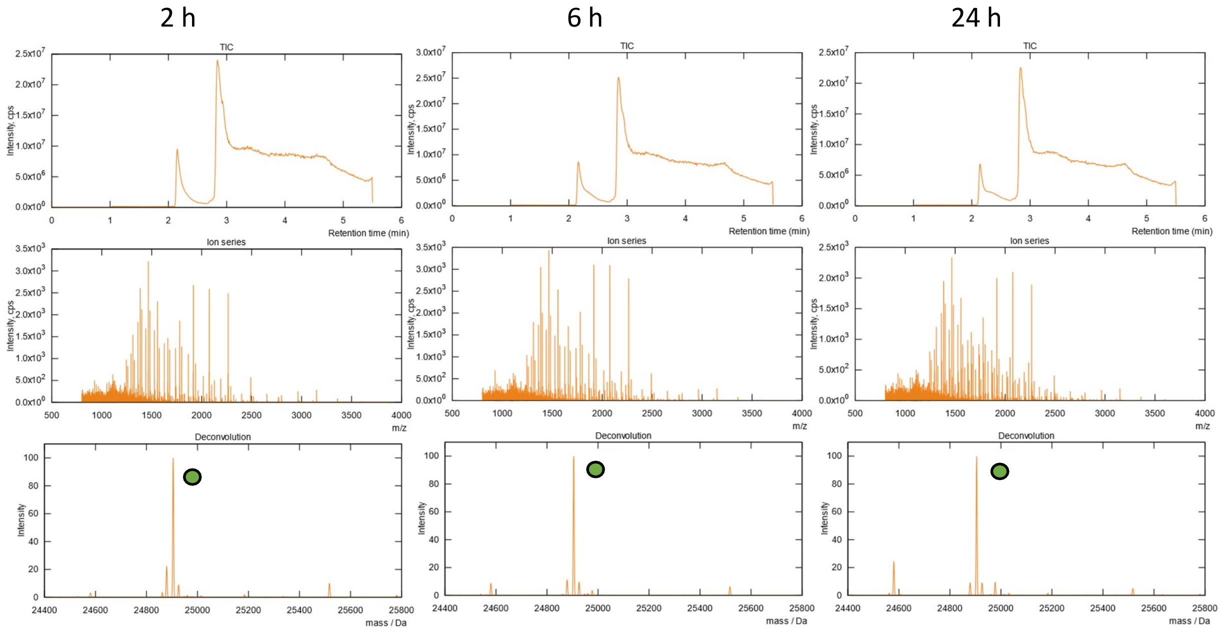

Figure S43. UV chromatograms, ion series and deconvoluted mass spectra obtained from LC-MS analysis of reaction mixture R2 after 2 h, 6 h and 24 h.

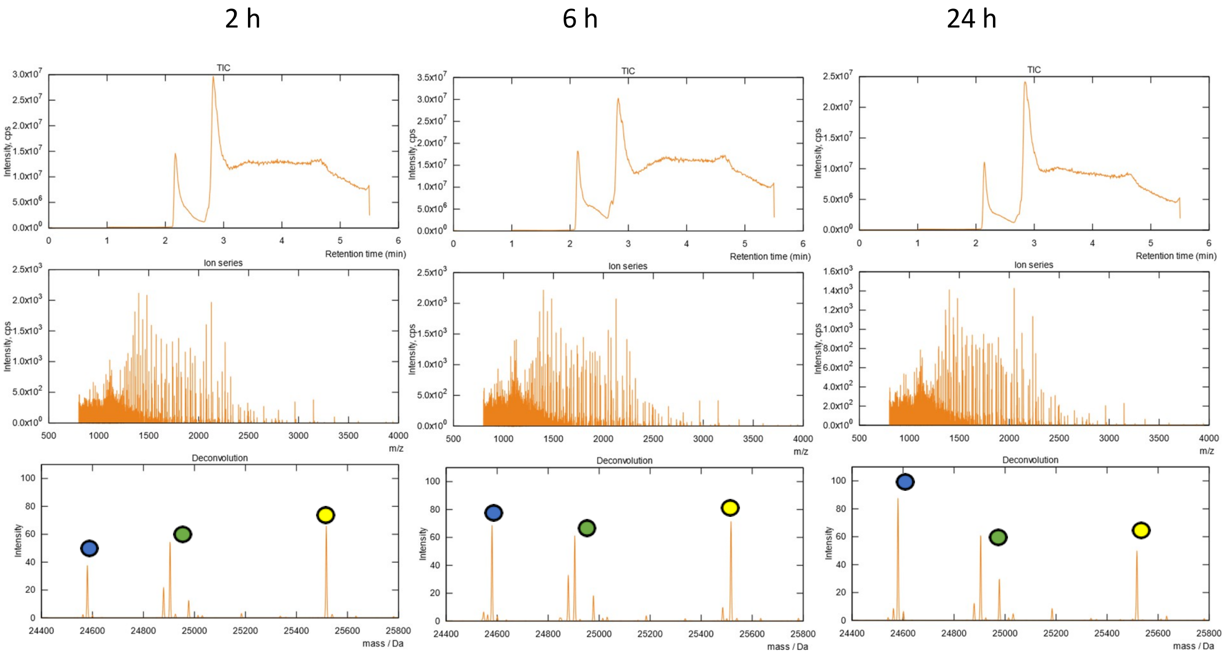

Figure S44. UV chromatograms, ion series and deconvoluted mass spectra obtained from LC-MS analysis of reaction mixture R3 after 2 h, 6 h and 24 h.

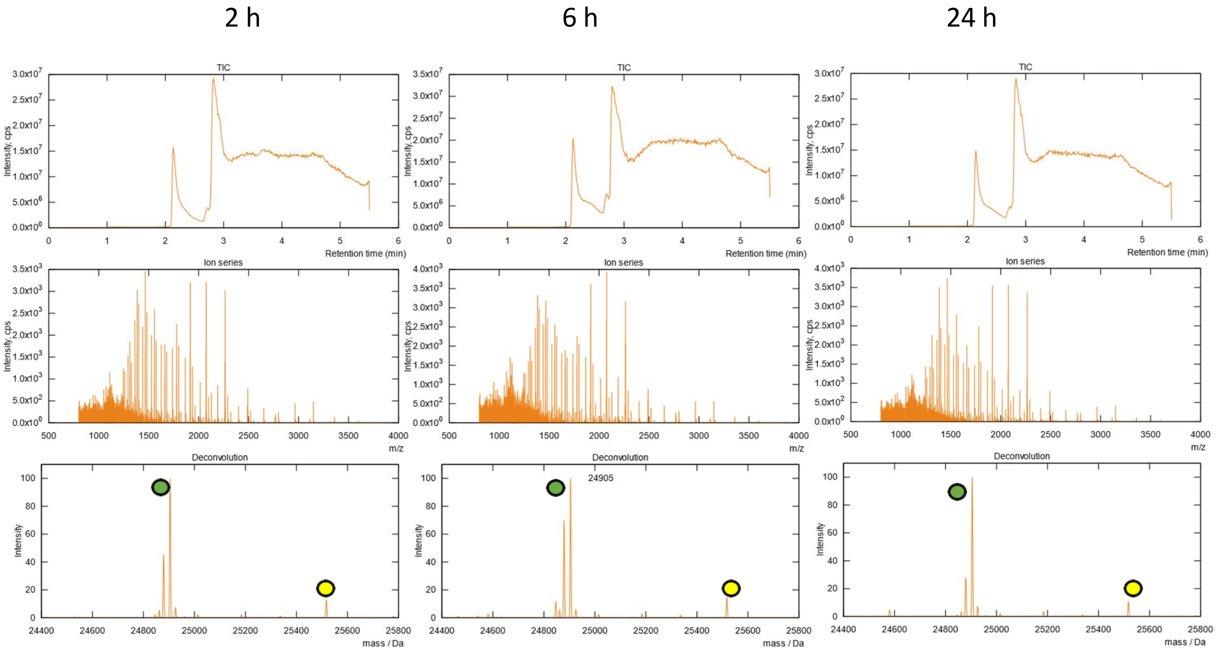

Figure S45. UV chromatograms, ion series and deconvoluted mass spectra obtained from LC-MS analysis of reaction mixture R4 after 2 h, 6 h and 24 h.

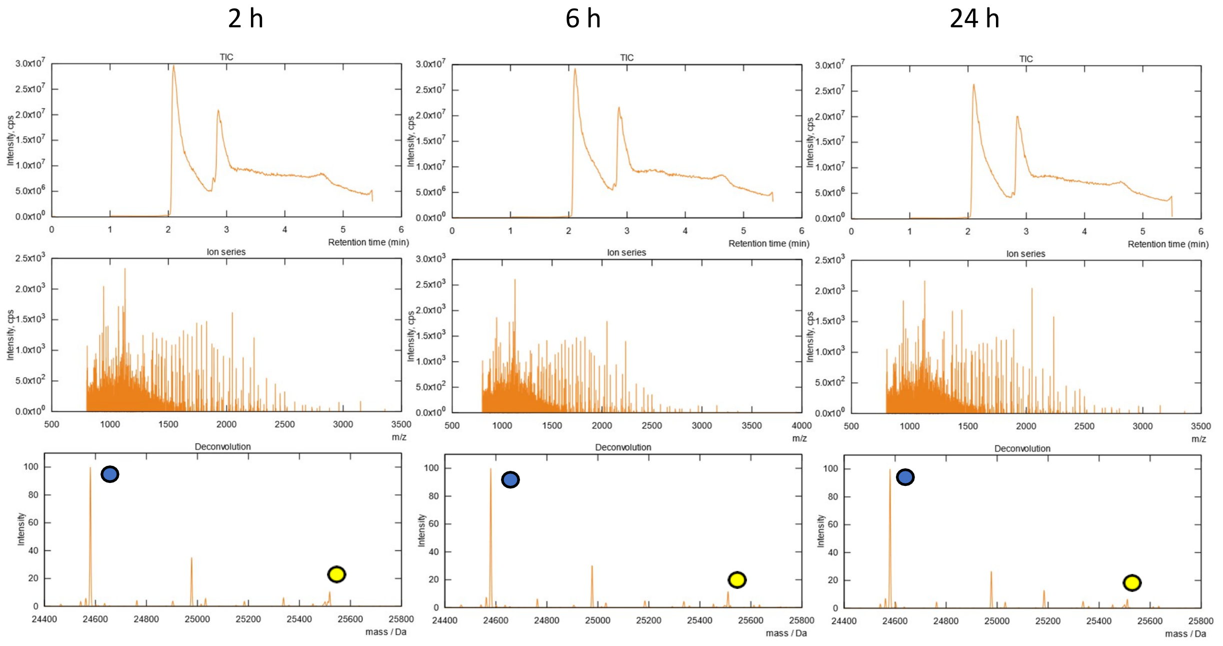

Figure S46. UV chromatograms, ion series and deconvoluted mass spectra obtained from LC-MS analysis of reaction mixture R5 after 2 h, 6 h and 24 h.

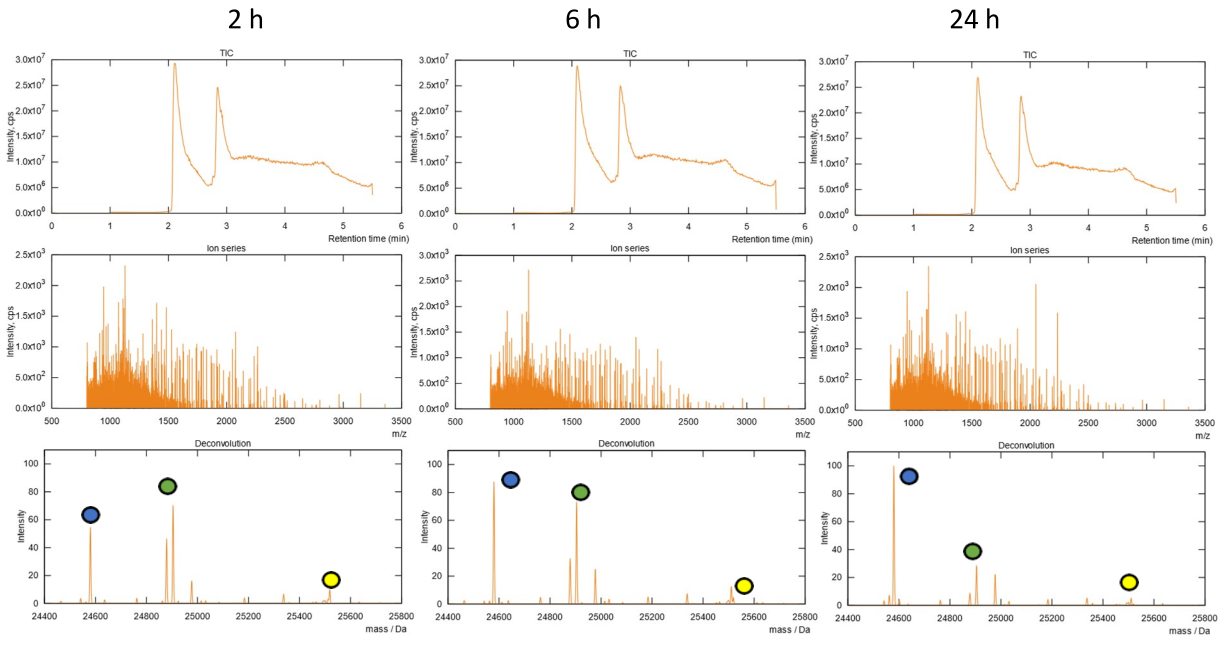

Figure S47. UV chromatograms, ion series and deconvoluted mass spectra obtained from LC-MS analysis of reaction mixture R6 after 2 h, 6 h and 24 h.

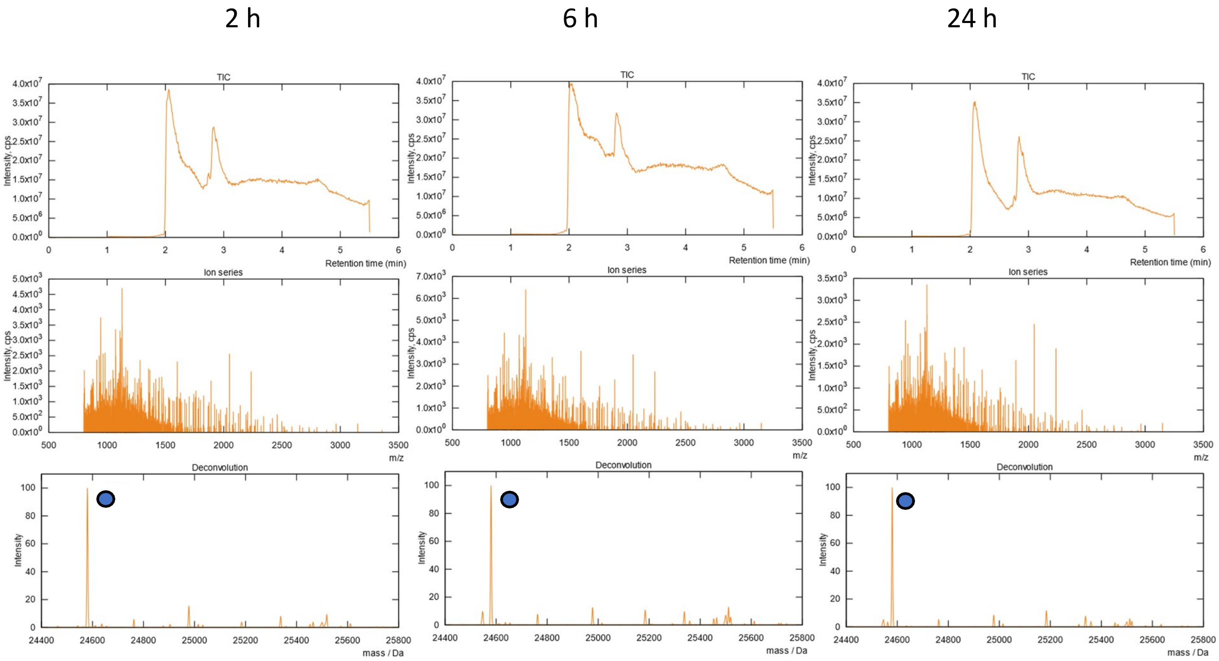

Figure S48. UV chromatograms, ion series and deconvoluted mass spectra obtained from LC-MS analysis of reaction mixture R7 after 2 h, 6 h and 24 h.

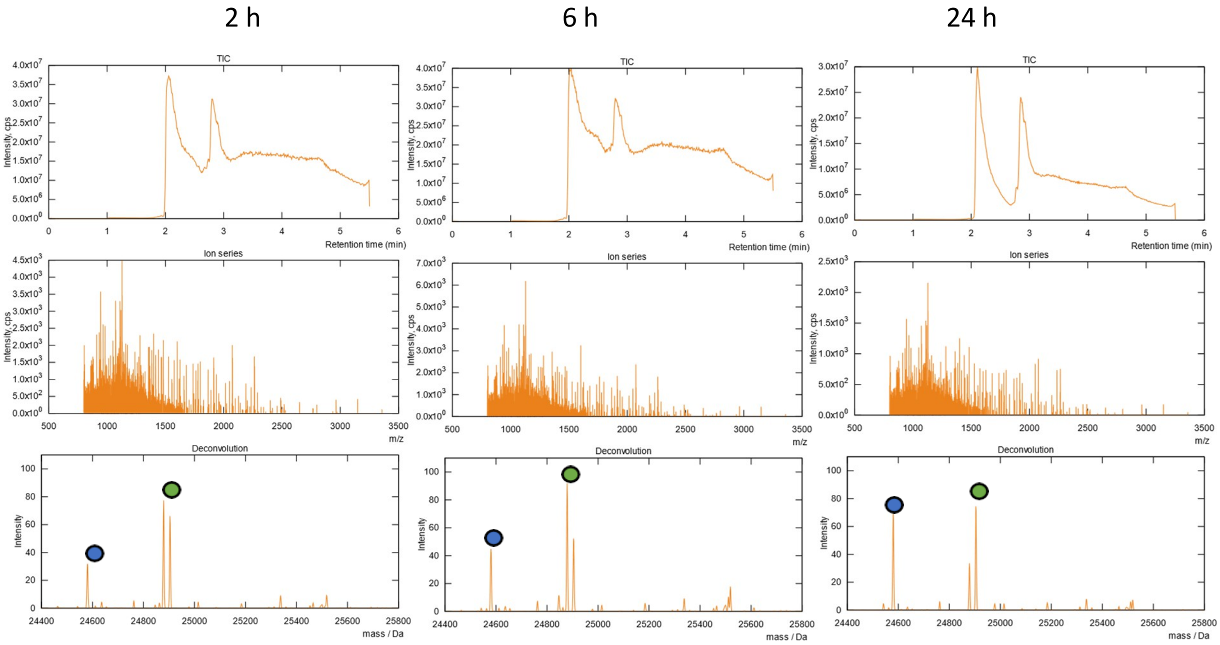

Figure S49. UV chromatograms, ion series and deconvoluted mass spectra obtained from LC-MS analysis of reaction mixture R8 after 2 h, 6 h and 24 h.

7.4.3 AntiCD33-(P4)

P4 was conjugated to antiCD33-(ST) following method "Sortase-mediated conjugation" described in S6.6 using 200 µL of protein solution . The resulting solution was then purified using Amicon® Ultra 0.5 mL Centrifugal Filters (10K MWCO). The purified product, termed antiCD33-(P4), was subjected to LC-MS analysis, which confirmed successful modification.

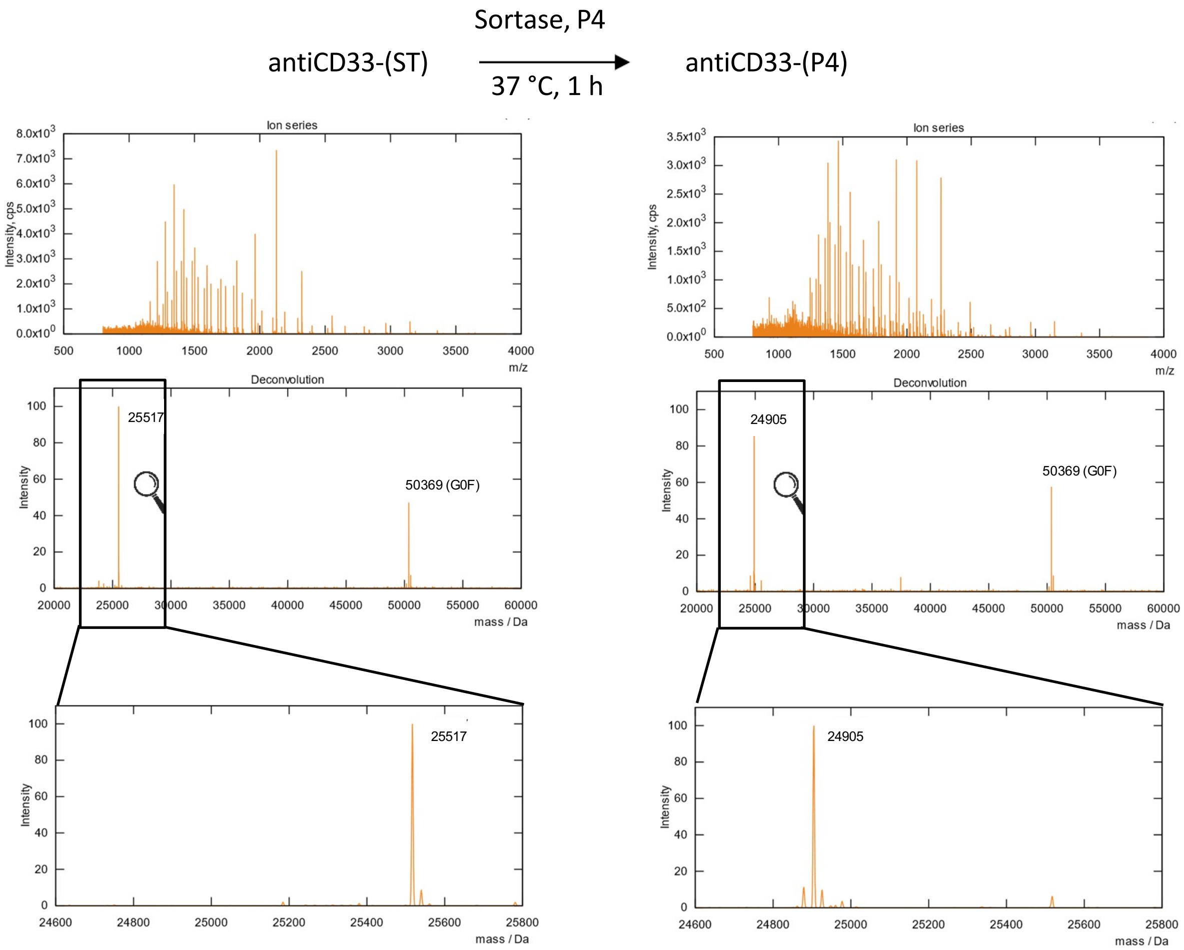

		Mass before reaction (Da)	Mass after reaction (Da)	Mass change (Da)
LC	Expected	25517	24903	-614
	Observed	25517	24905	-612
HC	Expected	50367	50367	0
	Observed	50369	50369	0

Figure S50. UV chromatograms, ion series and deconvoluted mass spectra obtained from LC-MS analysis of antiCD33-(ST) before and after modification.

7.4.4 AntiCD33-(P4-AF647)

DBCO-AF647 was conjugated to antiCD33-(P4) following method "SPAAC conjugation" described in S6.9 using 2 mL of protein solution. The resulting solution was purified via SEC. LC-MS and biophysical analysis were conducted to assess the identity, integrity and functionality of the product, which was termed antiCD33-(P4-AF647).

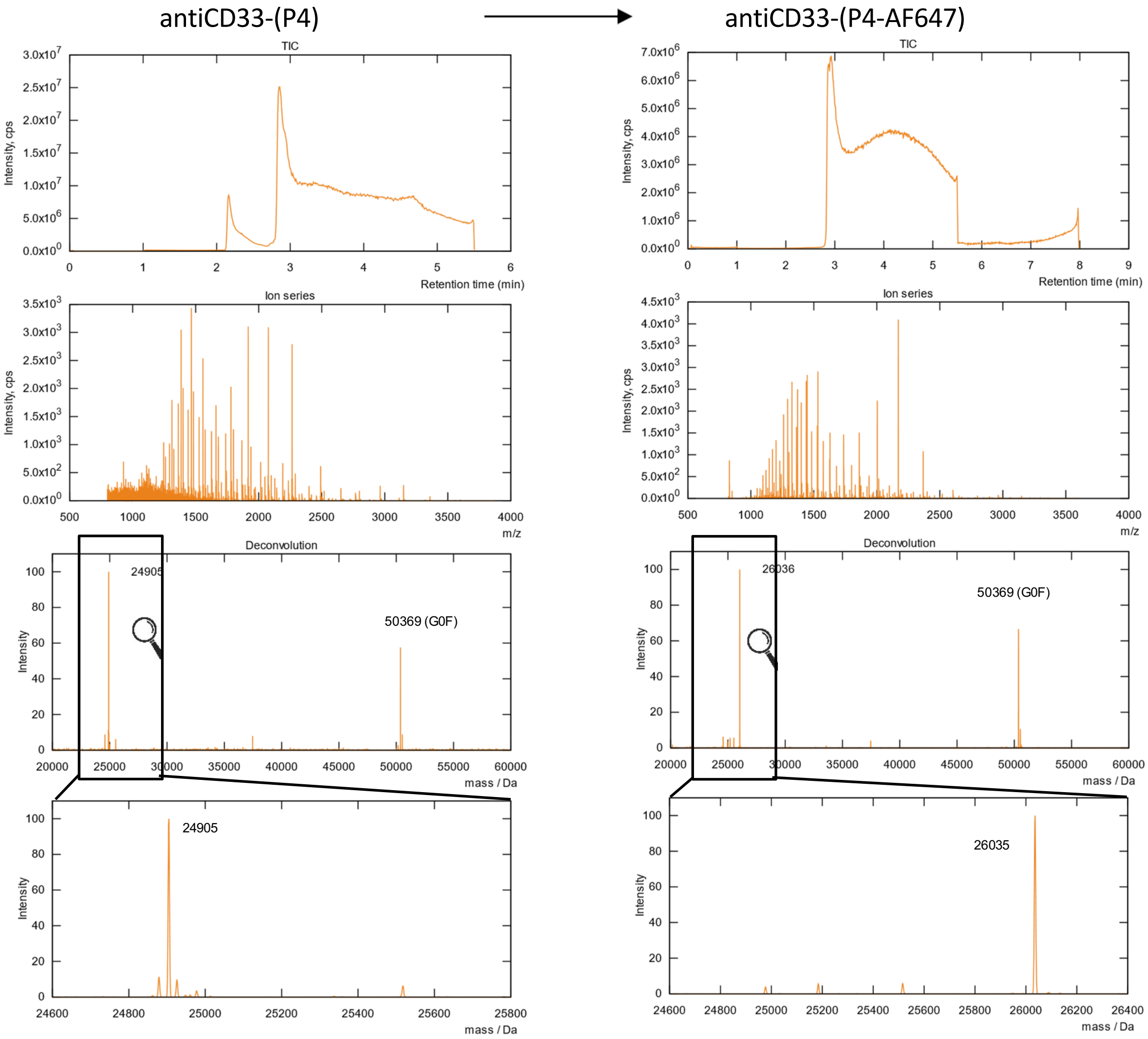

		Mass before reaction (Da)	Mass after reaction (Da)	Mass change (Da)
LC	Expected	25517	26034	+517
	Observed	25517	26035	+518
HC	Expected	50367	50367	0
	Observed	50369	50369	0

Figure S51. UV chromatograms, ion series and deconvoluted mass spectra obtained from LC-MS analysis of antiCD33-(P4) before and after modification.

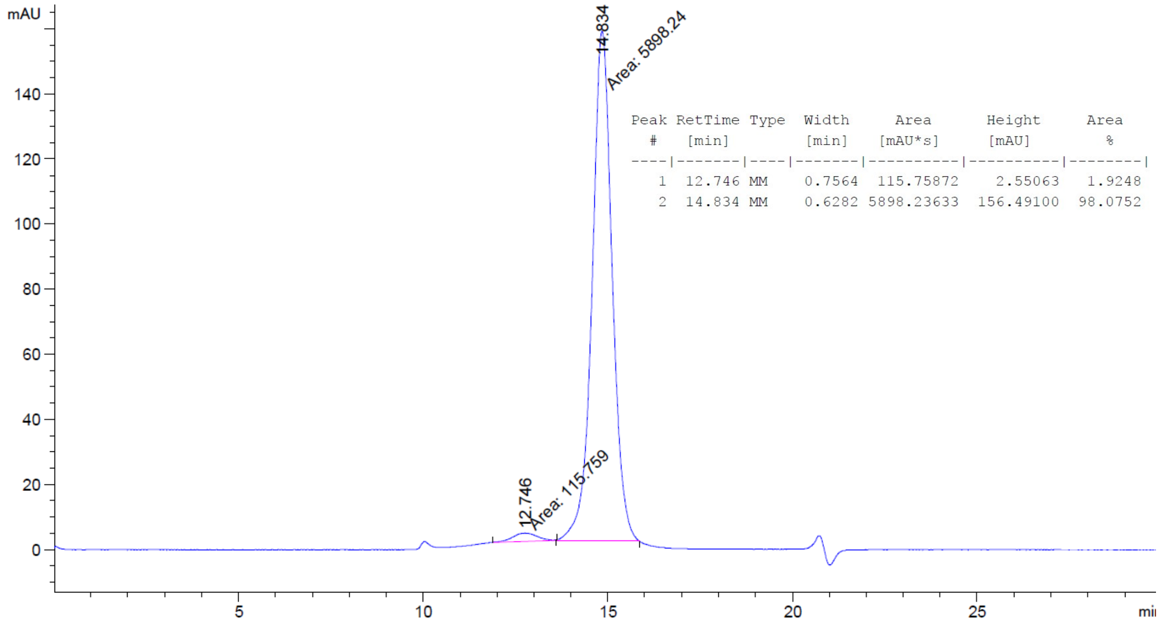

Figure S52. UV chromatogram obtained from HP- SEC analysis of antiCD33-(P4-AF647).

i)

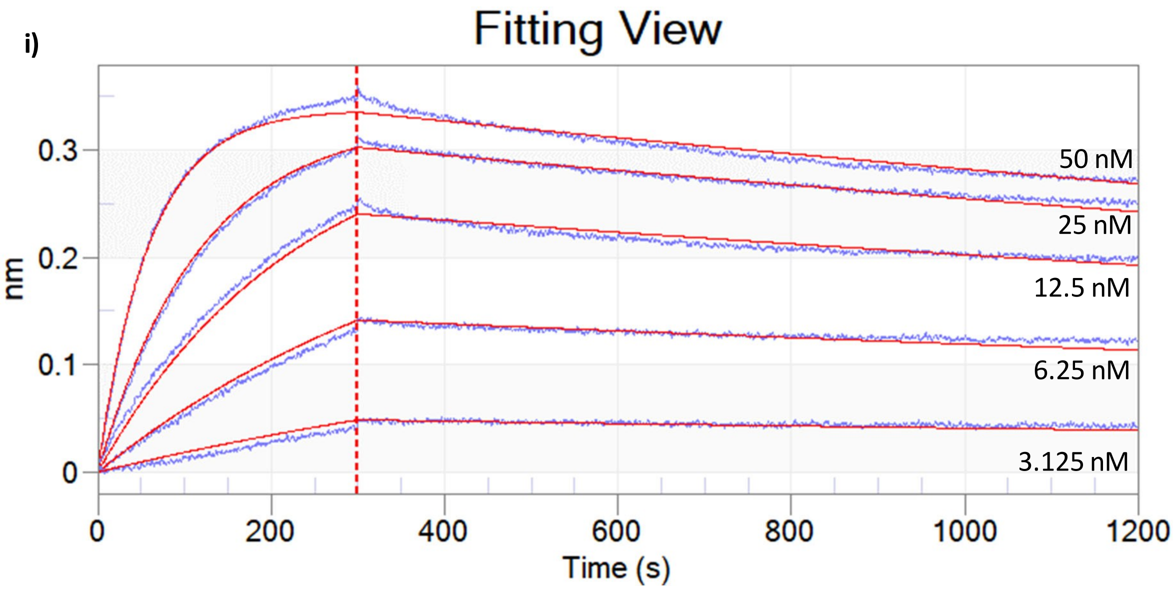

	K_D (M)	K_D Fitting Error (M)	K_{on} (1/Ms)	K_{on} error (1/Ms)	K_{dis} (1/s)	K_{dis} error (1/s)
antiCD33-(P4-AF647)	7.46E-10	4.25E-12	3.30E+05	1.15E+03	2.46E-04	1.11E-06
antiCD33-(Wildtype)	8.68E-10	4.97E-12	2.63E+05	9.12E+02	2.28E-04	1.04E-06

ii)

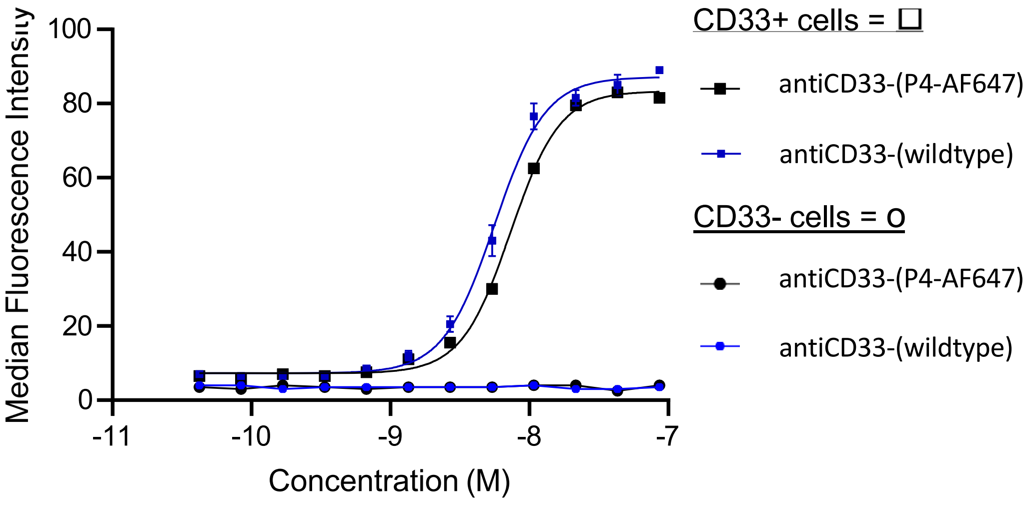

	EC50 (nM)	
	CD33-	CD33+
antiCD33-(Wildtype)	N/A	5.612 ± 0.361
antiCD33-(P4-AF647)	N/A	7.334 ± 0.113

Figure S53. Biophysical analysis of antiCD33-(P4-AF647). i) Binding curves and corresponding kinetic parameters obtained from BLI analysis. ii) Titrated FACS binding curve.

The stability of AntiCD33-(P4-AF647) was assessed following method “Stability study” described in 6.2.7. Resultant deconvolated mass spectra are shown below.

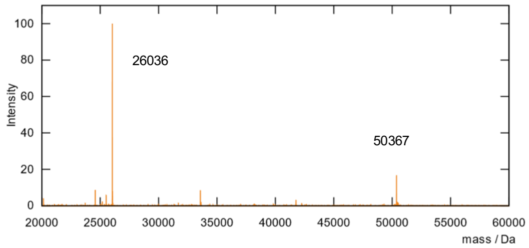

Initial AntiCD33-(P4-AF647)
LC: Calc.: 26034, Obs: 26036 (Relative intensity = 100%)
HC: Calc.: 50367, Obs: 50367
Other observed masses:
LC: 24579, 25516 (Relative intensity = 14%)

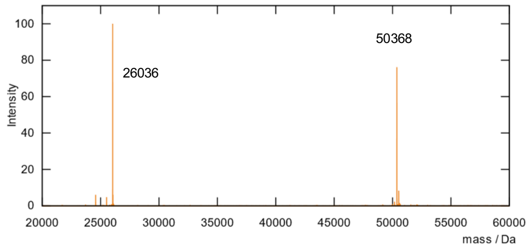

Day 2 Replicate 1 AntiCD33-(P4-AF647)
LC: Calc.: 26034, Obs: 26036 (Relative intensity = 100%)
HC: Calc.: 50367, Obs: 50368
Other observed masses:
LC: 24580, 25517 (Relative intensity = 10%)

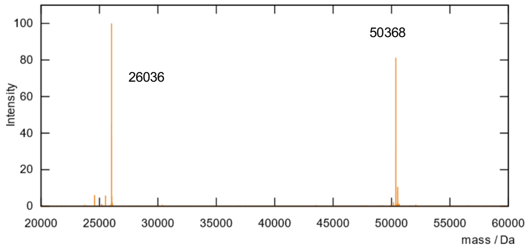

Day 2 Replicate 2 AntiCD33-(P4-AF647)
LC: Calc.: 26034, Obs: 26036 (Relative intensity = 100%)
HC: Calc.: 50367, Obs: 50368
Other observed masses:
LC: 24580, 25517 (Relative intensity = 12%)

Figure S54. Deconvoluted mass spectra of AntiCD33-(P4-AF647) before and after glutathione incubation.

7.5 Transglutaminase

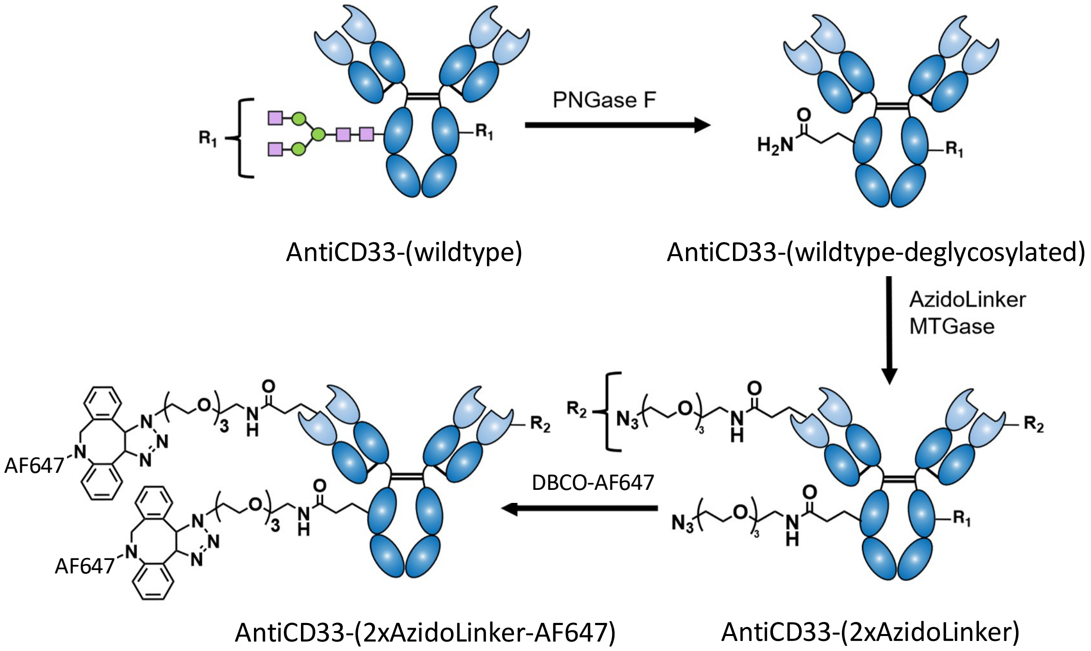

Figure S55. Schematic of the mTG-mediated modification protocol.

7.5.1 AntiCD33-(wildtype-deglycosylated)

AntiCD33-(Wildtype) antibody was deglyosylated following method “Deglycosylation” described in S6.4. The resulting solution was purified and concentrated to 17 mg/mL using an Amicon® Ultra 0.5 mL Centrifugal Filter (100K MWCO). The purified protein, termed antiCD33-(wildtype-deglycosylated), was then subjected to LC-MS analysis.

antiCD33-(Wildtype) —PNGase F, 37 °C, 20 h→ antiCD33-(wildtype-deglycosylated)

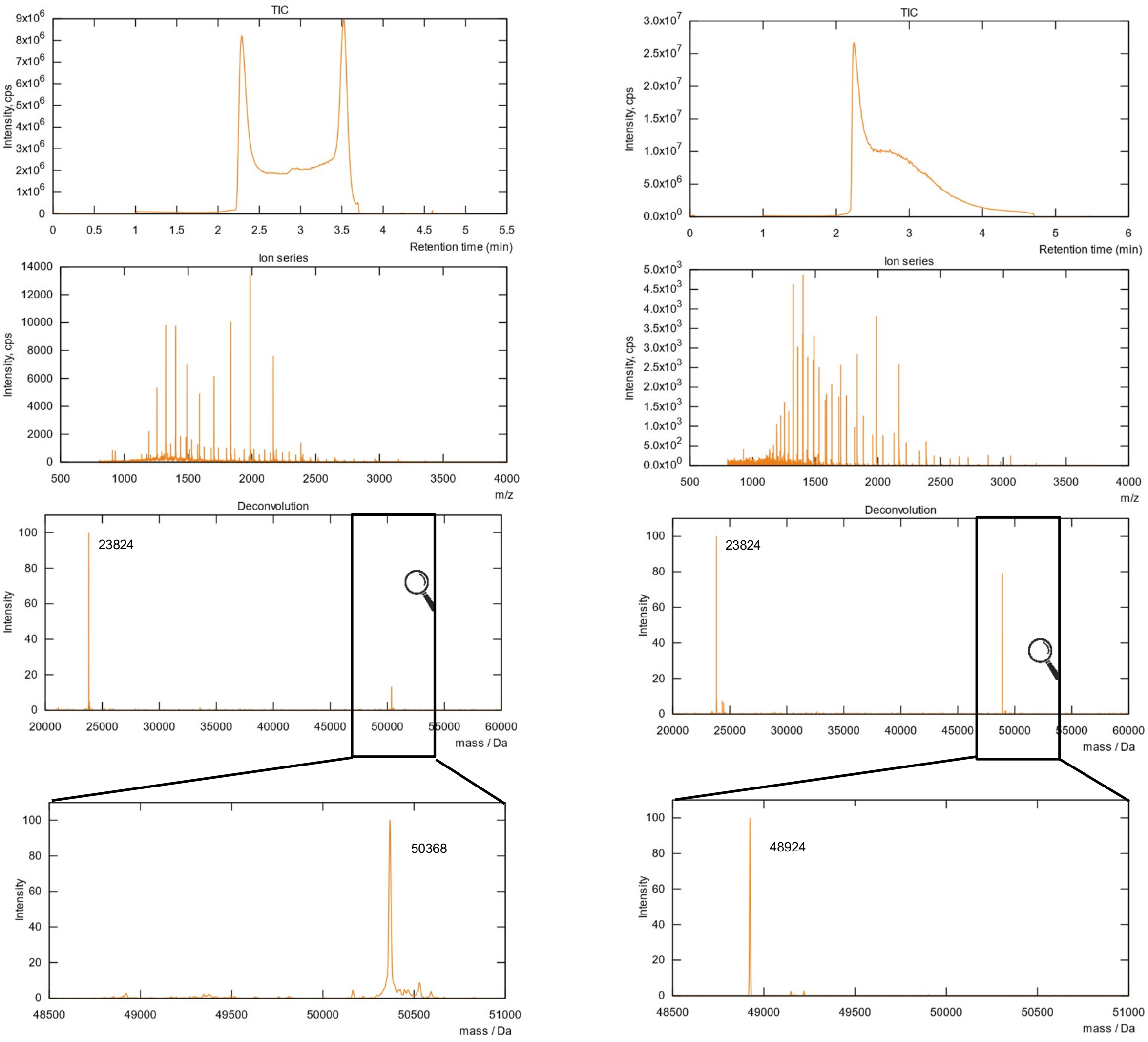

		Mass before reaction (Da)	Mass after reaction (Da)	Mass change (Da)
LC	Expected	23824	23824	0
	Observed	23824	23824	0
HC	Expected	50367	48922	-1445
	Observed	50368	48924	-1444

Figure S56. UV chromatograms, ion series and deconvoluted mass spectra obtained from LC-MS analysis of antiCD33-(Wildtype) before and after modification.

7.5.2 AntiCD33-(2XAzidoLinker)

Conjugation of AzidoLinker to antiCD33-(wildtype-deglycosylated) was performed following method "MTGase-mediated conjugation" described in 6.4.2.8. The resulting protein, named antiCD33-(2XAzidoLinker), was then purified using an Amicon® Ultra 0.5 mL Centrifugal Filter (100K MWCO) and subjected to reduced LC-MS and LC-MS-MS analysis.

antiCD33-(deglycosylated) → antiCD33-(2XAzidoLinker)

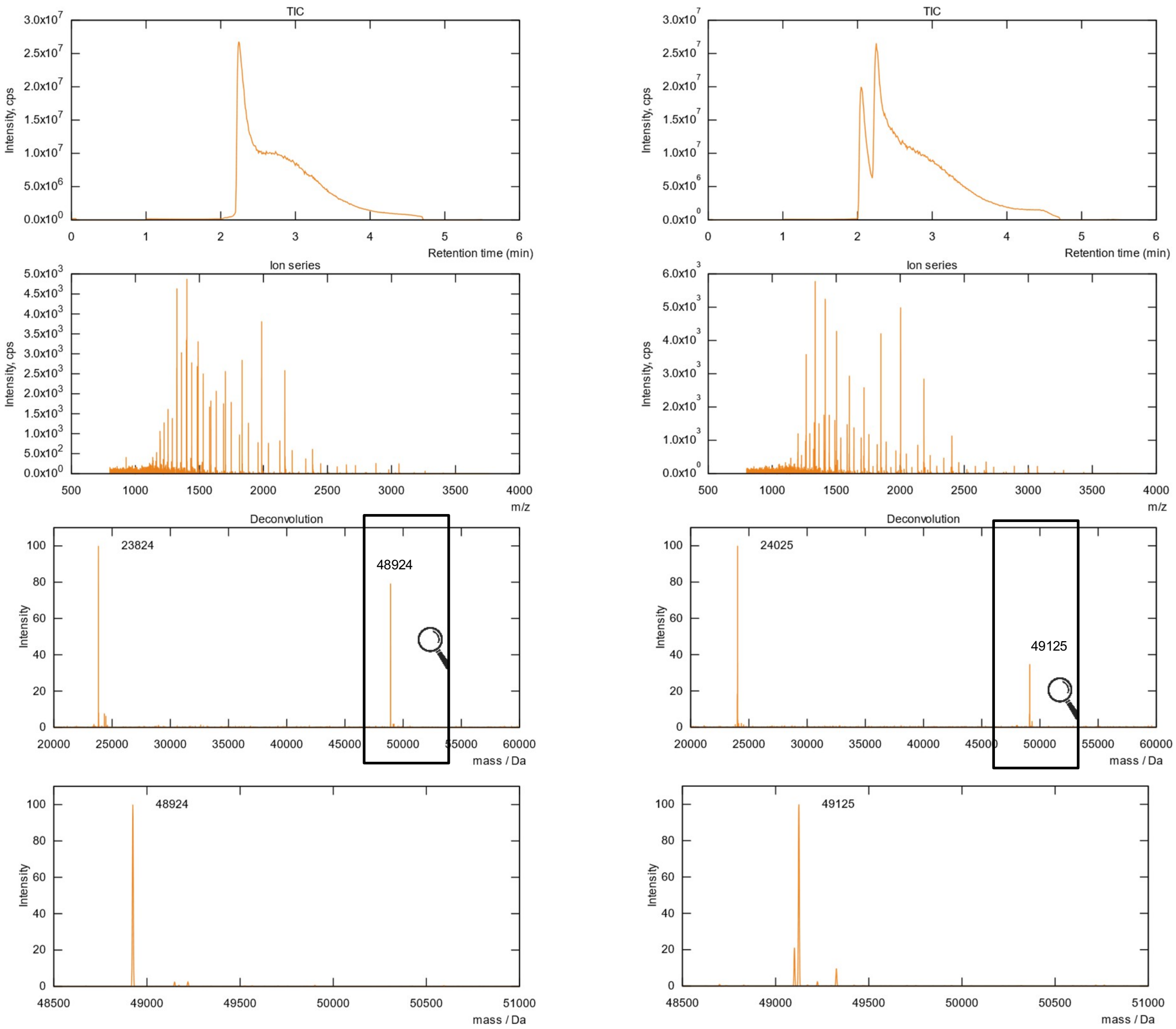

		Mass before reaction (Da)	Mass after reaction (Da)	Mass change (Da)
LC	Expected	23824	23824	+0
	Observed	23824	24025	+201
HC	Expected	48922	49123	+201
	Observed	48924	49125	+201

Figure S57. UV chromatograms, ion series and deconvoluted mass spectra obtained from LC-MS analysis of antiCD33-(wildtype-deglycosylated) before and after modification.

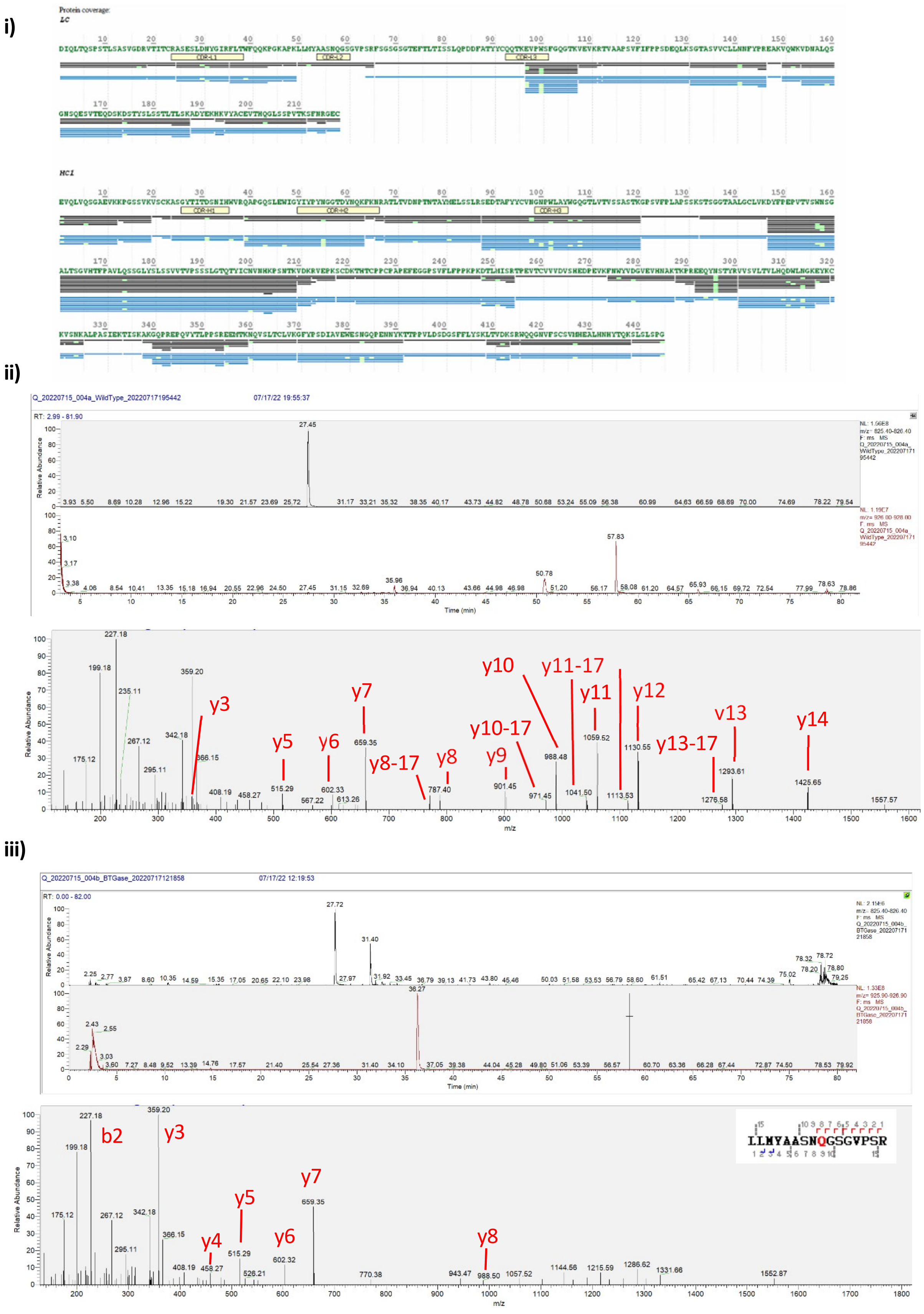

Figure S58. i) Protein coverage of antiCD33-(wildtype)(black) and antiCD33-(2XAzidoLinker)(Blue). ii) MS1/MS2 of peptide LLMYAASNQGSGVPSR from antiCD33-(wildtype). iii) MS1/MS2 of peptide LLMYAASN**Q**(azidolinker)GSGVPSR from antiCD33-(2XAzidoLinker).

7.5.3 AntiCD33-(2XAzidoLinker-AF647)

Conjugation of DBCO-AZDye647 to AntiCD33-(2XAzidoLinker) was performed following method "SPAAC conjugation" described in S6.9 using 100 µL of protein solution. The resulting solution was desalted using an Amicon® Ultra 0.5 mL Centrifugal Filter (100K MWCO). LC-MS and biophysical analysis were conducted to assess the identity, integrity and functionality of the product, which was termed antiCD33-(2XAzidoLinker-AF647).

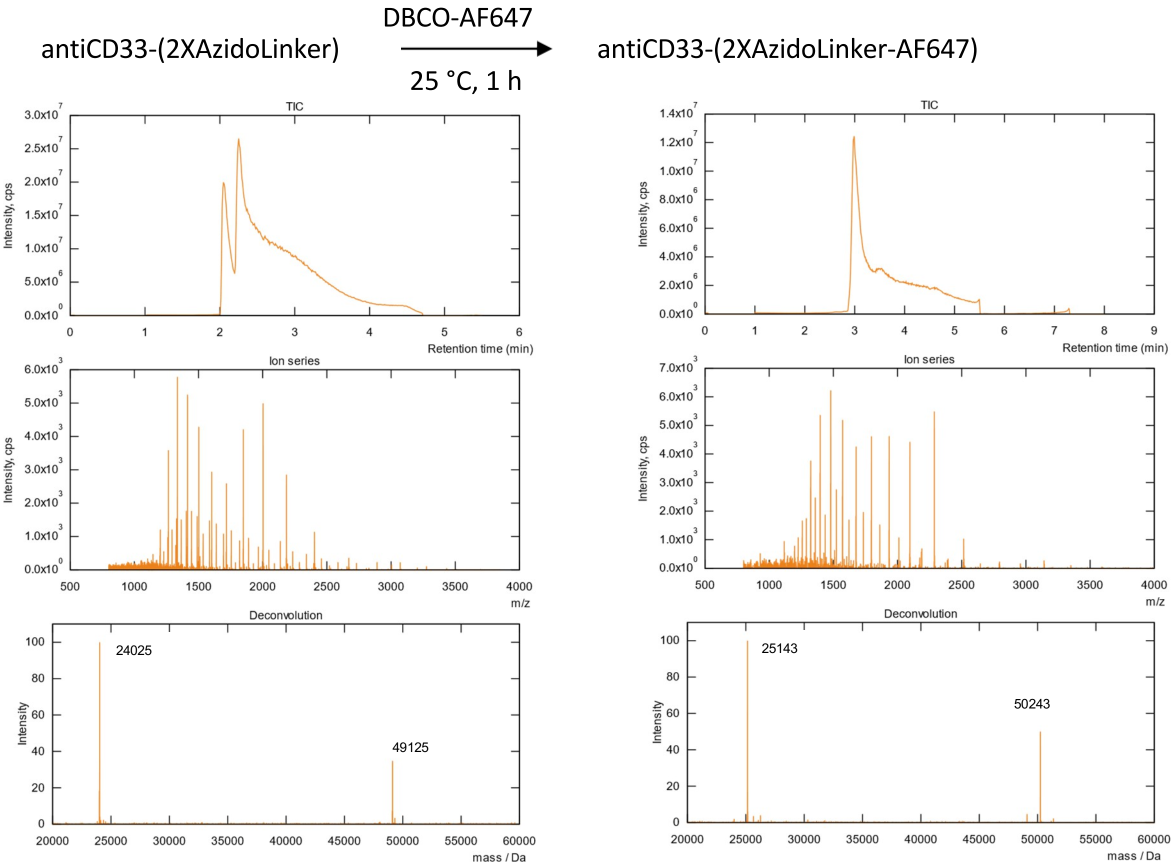

		Mass before reaction (Da)	Mass after reaction (Da)	Mass change (Da)
LC	Expected	23824	23824	+0
	Observed	24025	25143	+1118
HC	Expected	49123	50240	+1117
	Observed	49125	50243	+1118

Figure S59. UV chromatograms, ion series and deconvoluted mass spectra obtained from LC-MS analysis of antiCD33-(2XAzidoLinker) before and after modification.

7.6 GALaXy

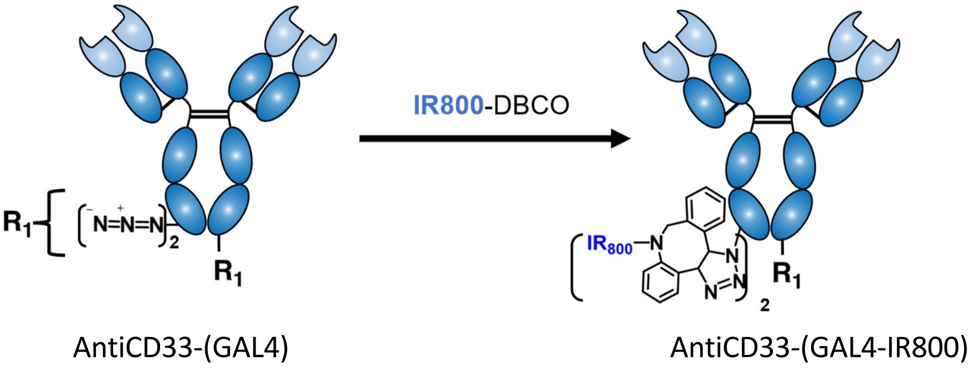

Figure S60. Schematic of the GALaXy-based modification protocol.

7.6.1 AntiCD33-(GAL4)

AntiCD33-(GAL4) was expressed and purified, following the protocol described in 6.2.4, achieving a post-purification yield of 24 mg L^{-1} (normalization factor from antiCD33-(Wildtype) parallel expression: 1.02, normalised yield: 93 mg L^{-1}). LC-MS and biophysical analysis were conducted to assess the identity and integrity of the product.

i)

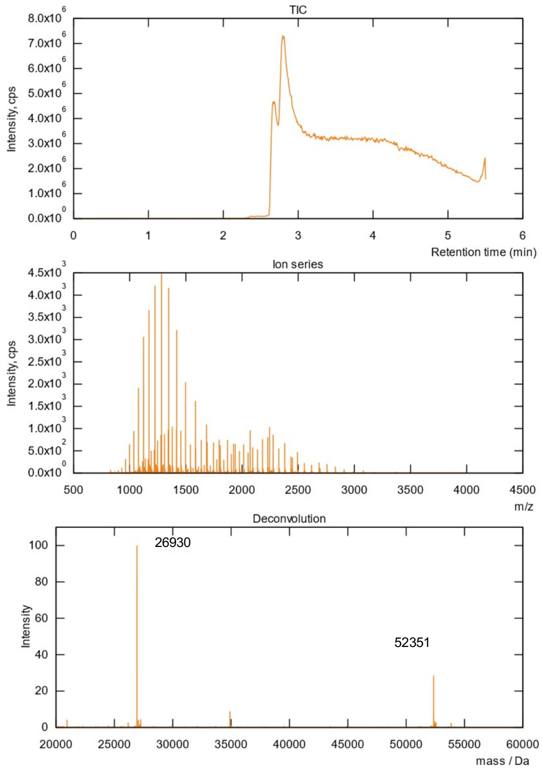

Expected mass (Da)	Observed mass (Da)
LC: 26930<br>HC: 52351	LC: 26930<br>HC: 52351

ii)

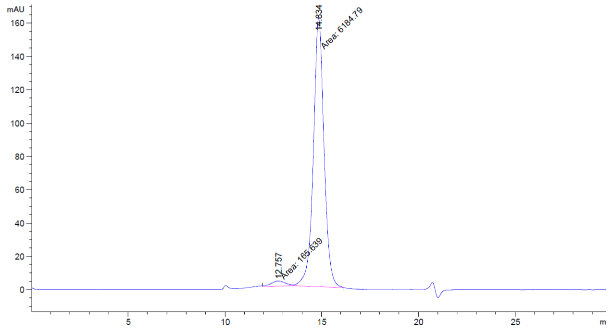

Peak #	RetTime [min]	Type	Width [min]	Area [mAU*s]	Height [mAU]	Area %
1	12.757	MM	0.8817	165.63892	3.13102	2.6083
2	14.834	MM	0.6398	6184.78857	161.10677	97.3917

Figure S61. i) UV chromatograms, ion series and deconvoluted mass spectra obtained from LC-MS analysis of antiCD33-(GAL4) under reduced conditions. ii) UV chromatogram obtained from HP-SEC analysis of antiCD33-(GAL4).

7.6.2 AntiCD33-(GAL4-IR800)

Conjugation of IR800-DBCO to antiCD33-(GAL4) was performed following method "SPAAC" conjugation described in S6.9 using 200 µL protein solution. The resulting solution was desalted using an Amicon® Ultra 0.5 mL Centrifugal Filter (100K MWCO).

antiCD33-(GAL4) —(IR800-DBCO; 25 °C, 1 h)→ antiCD33-(GAL4-IR800)

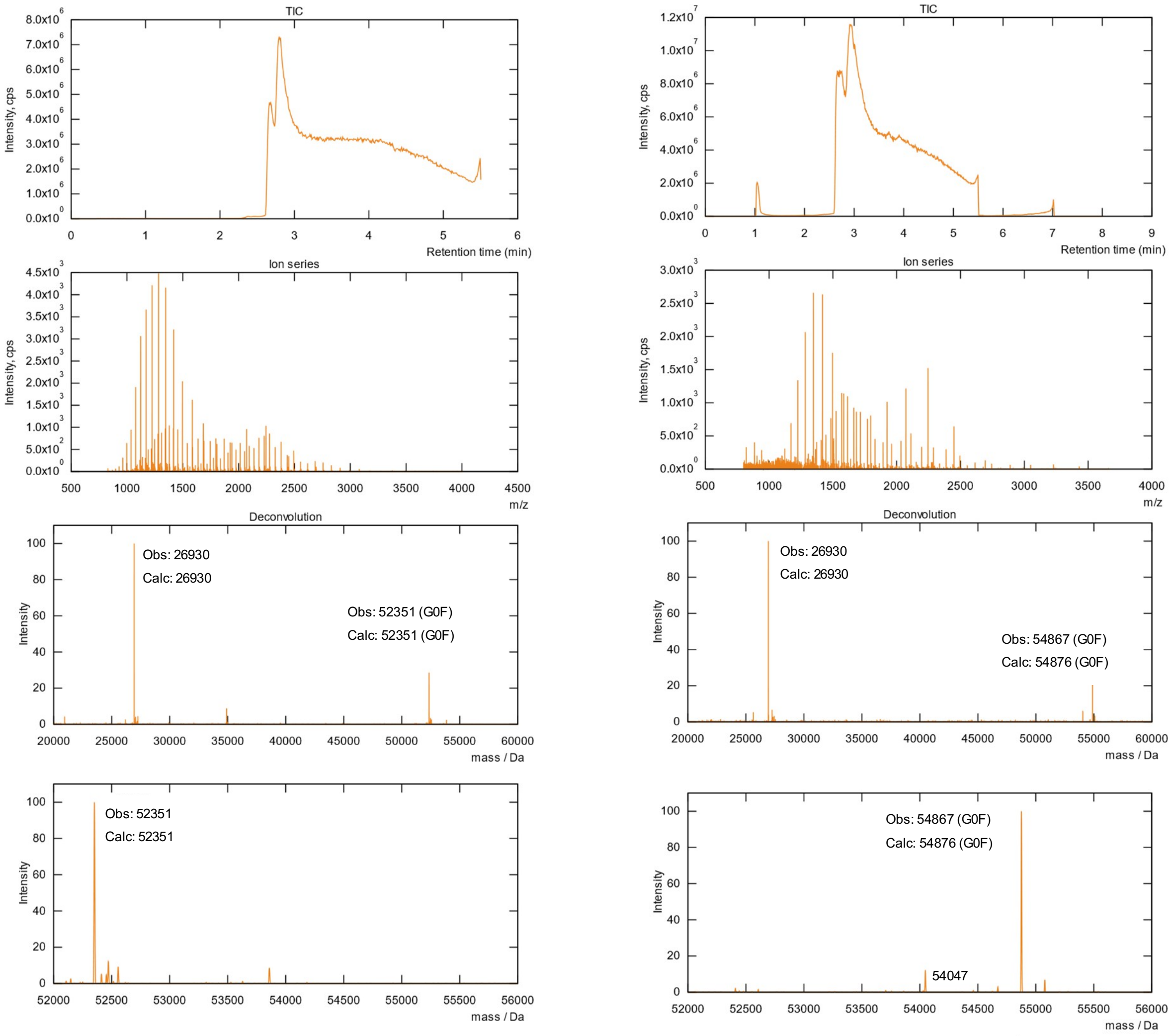

		Mass before reaction (Da)	Mass after reaction (Da)	Mass change (Da)
LC	Expected	26930	26930	0
	Observed	26930	26930	0
HC	Expected	52351	54867	+2516
	Observed	52351	54876	+2525

Figure S62. i) UV chromatograms, ion series and deconvoluted mass spectra obtained from LC-MS analysis of antiCD33-(GAL4) before and after modification.

7.7 Dual modification: CPO + maleimide (239iC)

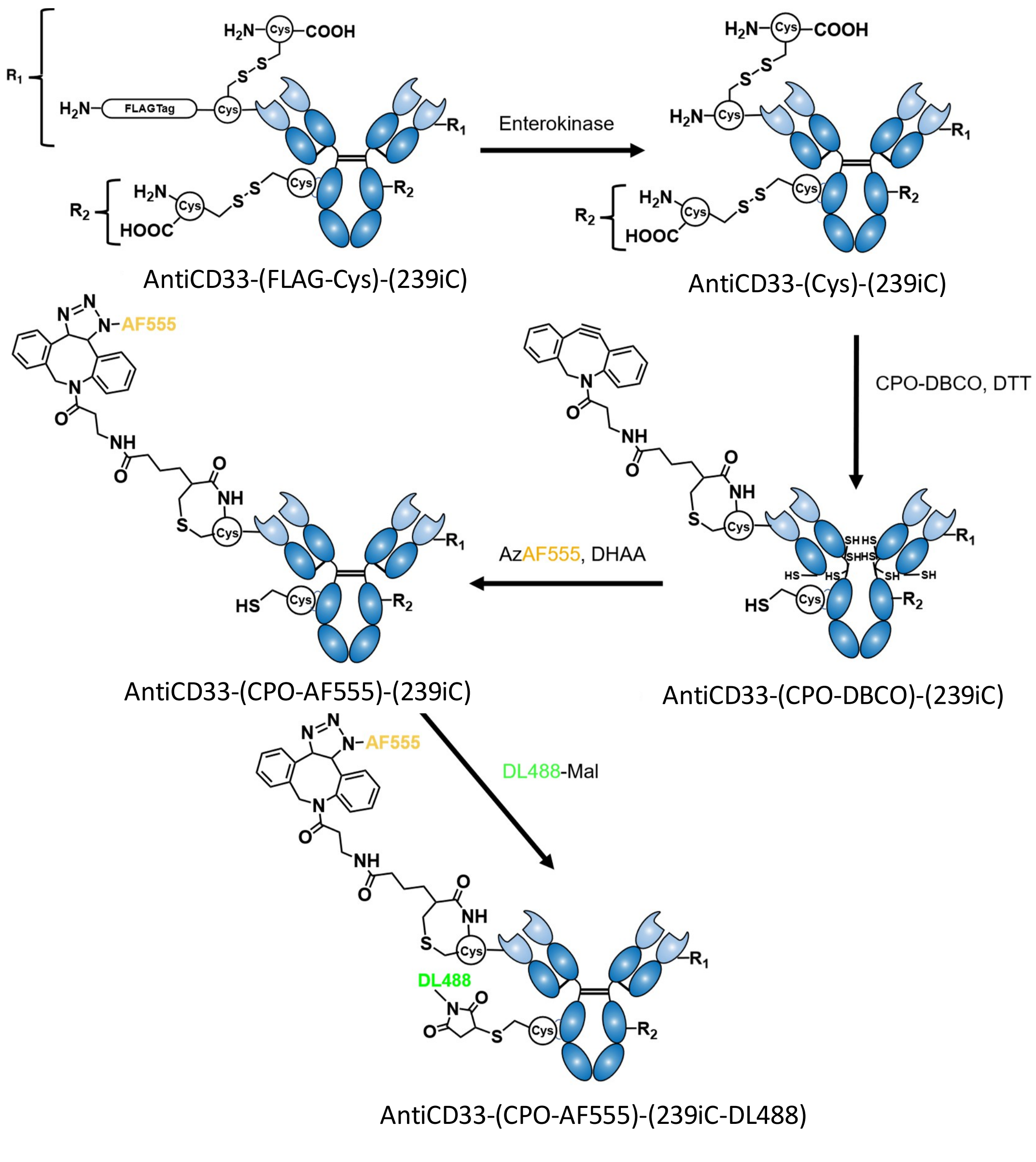

Figure S63. Schematic of the CPO and maleimide (239iC) based modification protocol.

7.7.1 AntiCD33-(FLAG-Cys)-(239iC)

AntiCD33-(FLAG-Cys)-(239iC) was expressed and purified, following the protocol described in 6.2.4, achieving a post-purification yield of 45 mg L^{-1} (Normalised to WT = 50 mg L^{-1}). LC-MS and biophysical analysis were conducted to assess the identity and integrity of the product.

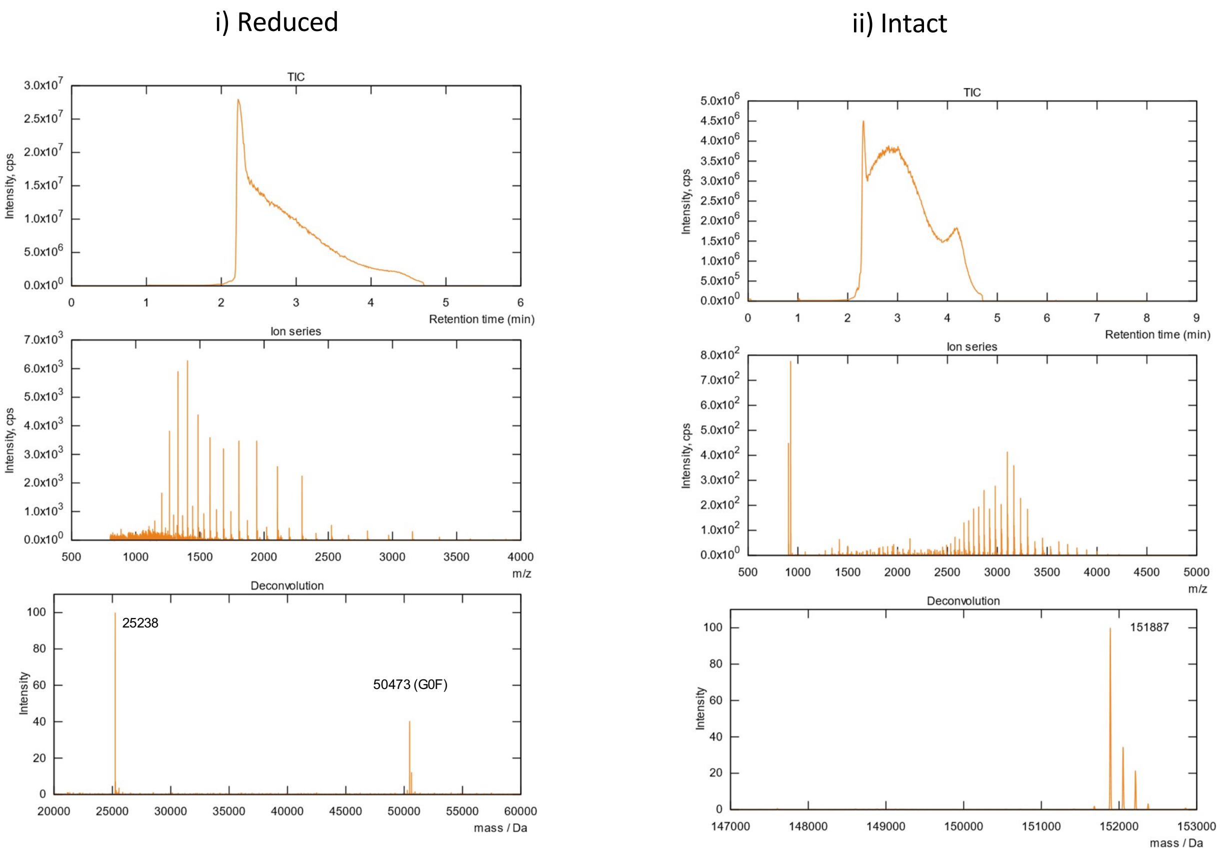

Ref.	Expected mass (Da)	Observed mass (Da)
i	LC: 25238<br>HC: 50470	LC: 25238<br>HC: 50473
ii	151884	151887

Figure S64. UV chromatograms, ion series and deconvoluted mass spectra obtained from LC-MS analysis of antiCD33-(FLAG-Cys)-(239iC) under reduced (i) and intact (ii) conditions.

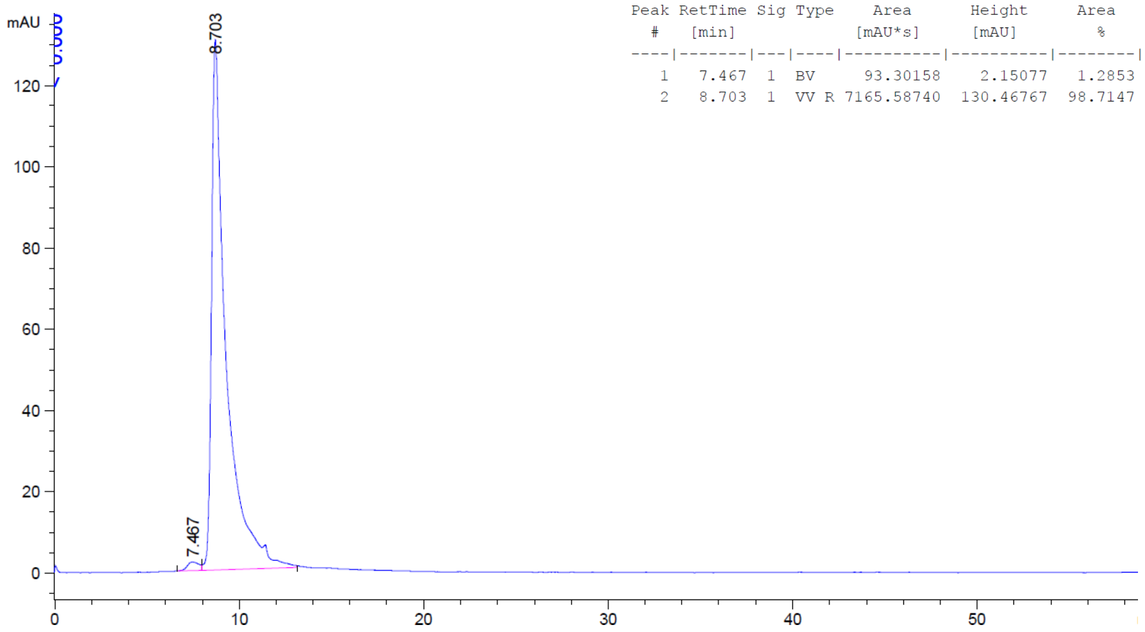

Peak #	RetTime [min]	Sig	Type	Area [mAU*s]	Height [mAU]	Area %
1	7.467	1	BV	93.30158	2.15077	1.2853
2	8.703	1	VV R	7165.58740	130.46767	98.7147

Figure S65. UV chromatogram obtained from HP-SEC analysis of antiCD33-(FLAG-Cys)-(239iC).

7.7.2 AntiCD33-(Cys)-(239iC)

The FLAG tag of AntiCD33-(FLAG-Cys)-(239iC) was removed following method “FLAG cleavage”, described in S6.2. The subsequent reaction mixture was desalted using an Amicon® Ultra 0.5 mL Centrifugal Filter (10K MWCO). The purified protein, termed antiCD33-(Cys)-(239iC), was then characterised by LC-MS, which confirmed successful cleavage of the FLAG tag and formation of an N-terminal cysteine.

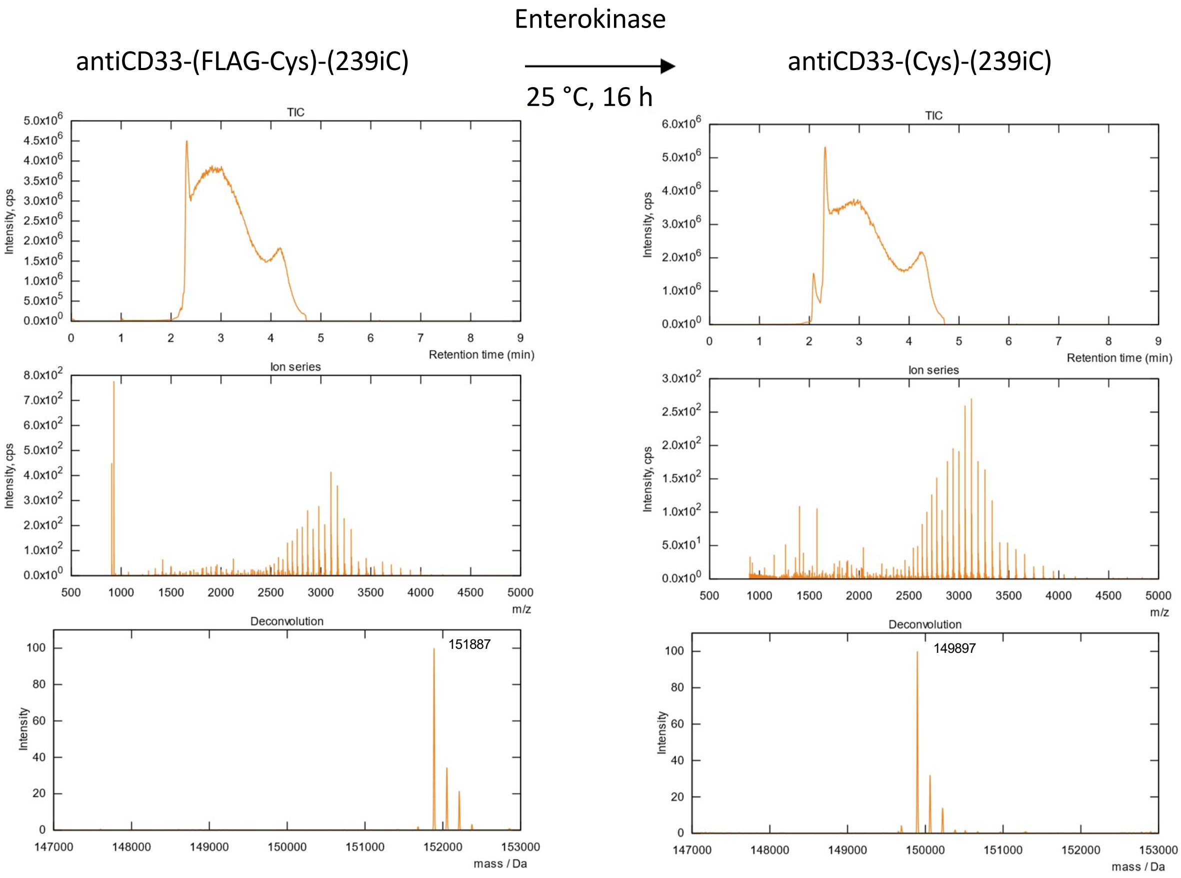

	Mass before Cleavage (Da)	Mass after Cleavage (Da)	Mass change (Da)
Expected	151884	149894	-1990
Observed	151887	149897	-1990

Figure S66. UV chromatograms, ion series and deconvoluted mass spectra obtained from LC-MS analysis of antiCD33-(FLAG-Cys)-(239iC) before and after cleavage.

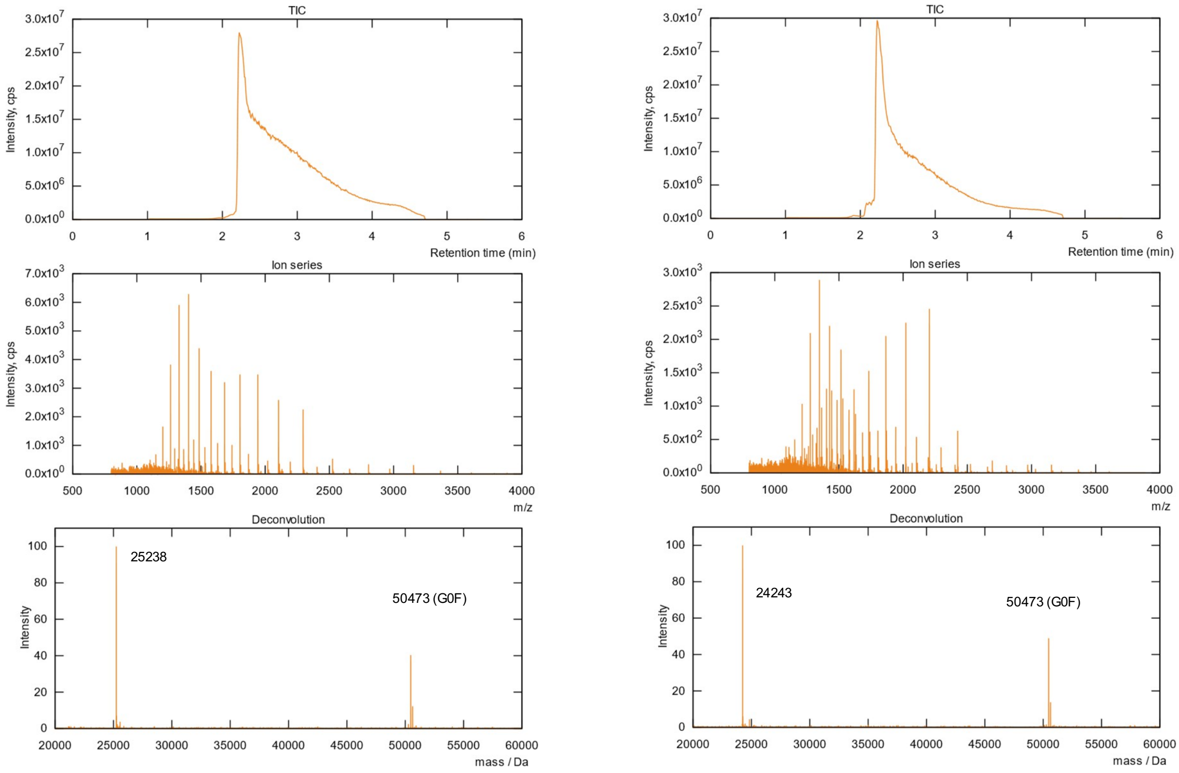

		Mass before cleavage (Da)	Mass after cleavage (Da)	Mass change (Da)
LC	Expected	25238	24243	-995
	Observed	25238	24243	-995
HC	Expected	50470	50470	0
	Observed	50473	50473	0

Figure S67. UV chromatograms, ion series and deconvoluted mass spectra obtained from LC-MS analysis of antiCD33-(FLAG-Cys)-(239iC) before and after cleavage.

7.7.3 AntiCD33-(CPO-DBCO)-(239iC)

Conjugation of CPO-DBCO to antiCD33-(Cys)-(239iC) was performed following method "CPO conjugation" described in S6.5 using 2 mL of protein solution. The subsequent reaction mixture was purified using an Amicon® Ultra 0.5 mL Centrifugal Filter (10K MWCO). The purified protein, termed antiCD33-(CPO-DBCO)-(239iC), was characterised by LC-MS, which confirmed successful CPO-DBCO modification.

antiCD33-(Cys)-(239iC) —CPO-DBCO, 25 °C, 4 h→ antiCD33-(CPO-DBCO)-(239iC)

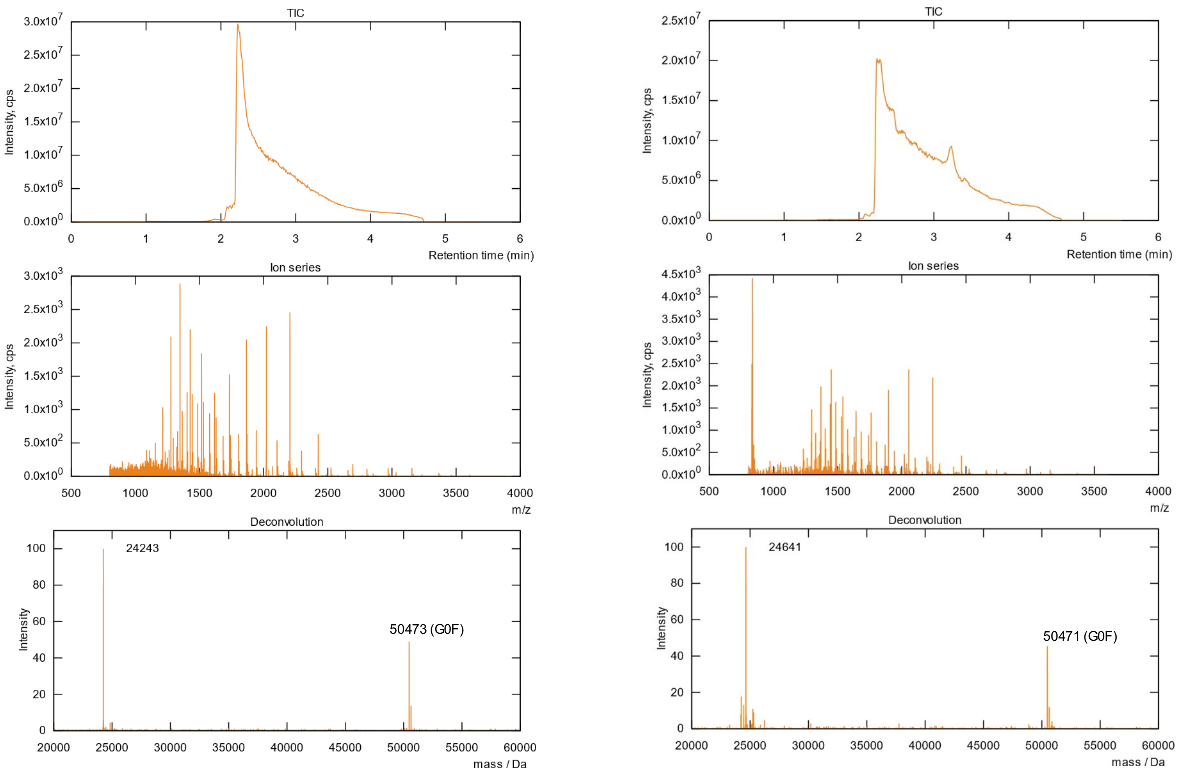

		Mass before reaction (Da)	Mass after reaction (Da)	Mass change (Da)
LC	Expected	24243	24641	398
	Observed	24243	24641	398
HC	Expected	50470	50470	0
	Observed	50473	50471	-2

Figure S68. UV chromatograms, ion series and deconvoluted mass spectra obtained from LC-MS analysis of antiCD33-(Cys)-(239iC) before and after modification.

7.7.4 AntiCD33-(AF555AzDBCO-CPONTerm-LC)-(239iC-HC)

Conjugation of AzAF555 to AntiCD33-(CPO-DBCO)-(239iC) was performed following method “SPAAC conjugation” described in S6.9 using 2 mL of protein solution. The resulting solution was purified by SEC. The purified protein, termed antiCD33-(CPO-AF555)-(239iC) was characterised by LC-MS, which confirmed successful conjugation of AzAF555.

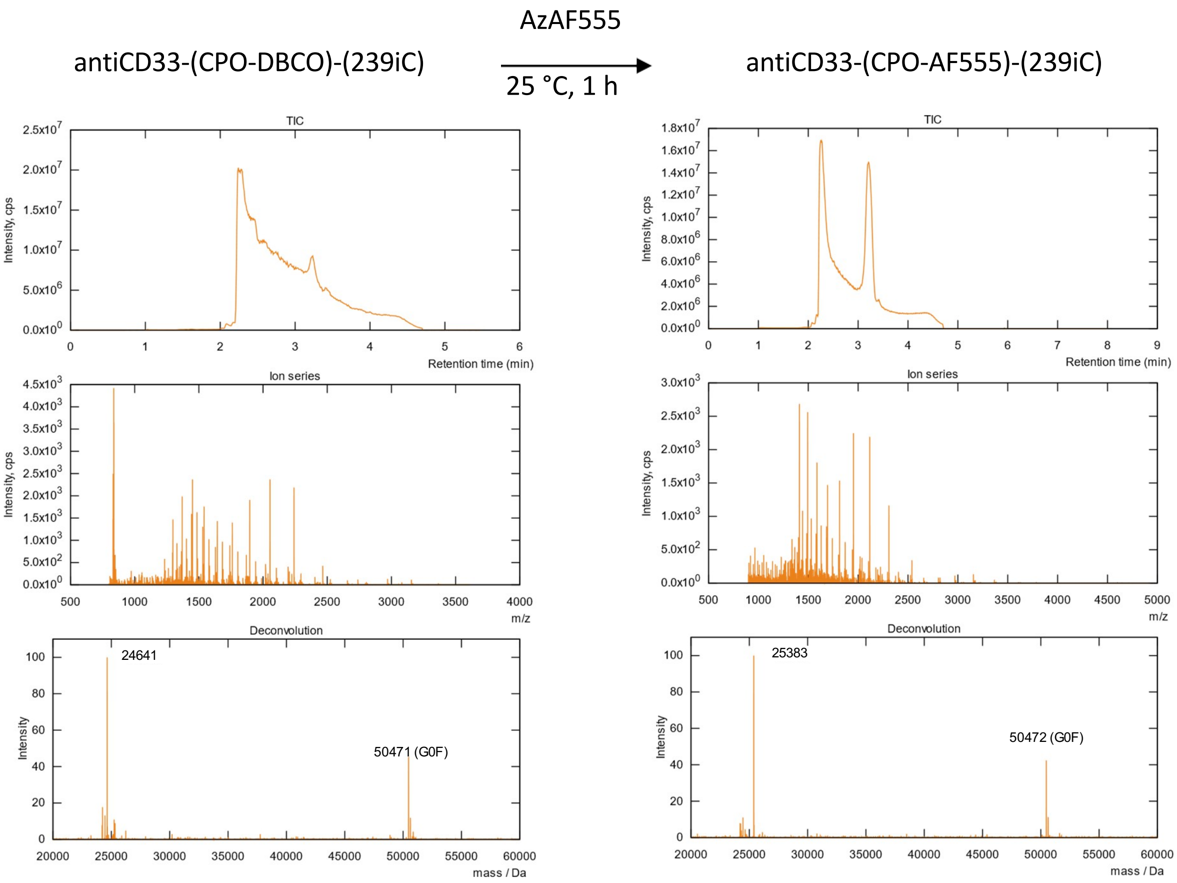

		Mass before reaction (Da)	Mass after reaction (Da)	Mass change (Da)
LC	Expected	24641	25381	+740
	Observed	24641	25383	+742
HC	Expected	50470	50470	0
	Observed	50471	50472	+1

Figure S69. UV chromatograms, ion series and deconvoluted mass spectra obtained from LC-MS analysis of antiCD33-(CPO-DBCO)-(239iC-HC) before and after modification.

7.7.5 AntiCD33-(CPO-AF555)-(239iC-DL488)

Conjugation of DL488-Mal to AntiCD33-(CPO-AF555)-(239iC) was performed following method “Maleimide conjugation” described in S6.10 using 300 µL of protein solution. The resulting solution was desalted using an Amicon® Ultra 0.5 mL Centrifugal Filter (100K MWCO). LC-MS and biophysical analysis were conducted to assess the identity, integrity and functionality of the product, which was termed antiCD33-(CPO-AF555)-(239iC-DL488).

antiCD33-(CPO-AF555)-(239iC) —DL488-Mal, 25 °C, 1 h→ antiCD33-(CPO-AF555)-(239iC-DL488)

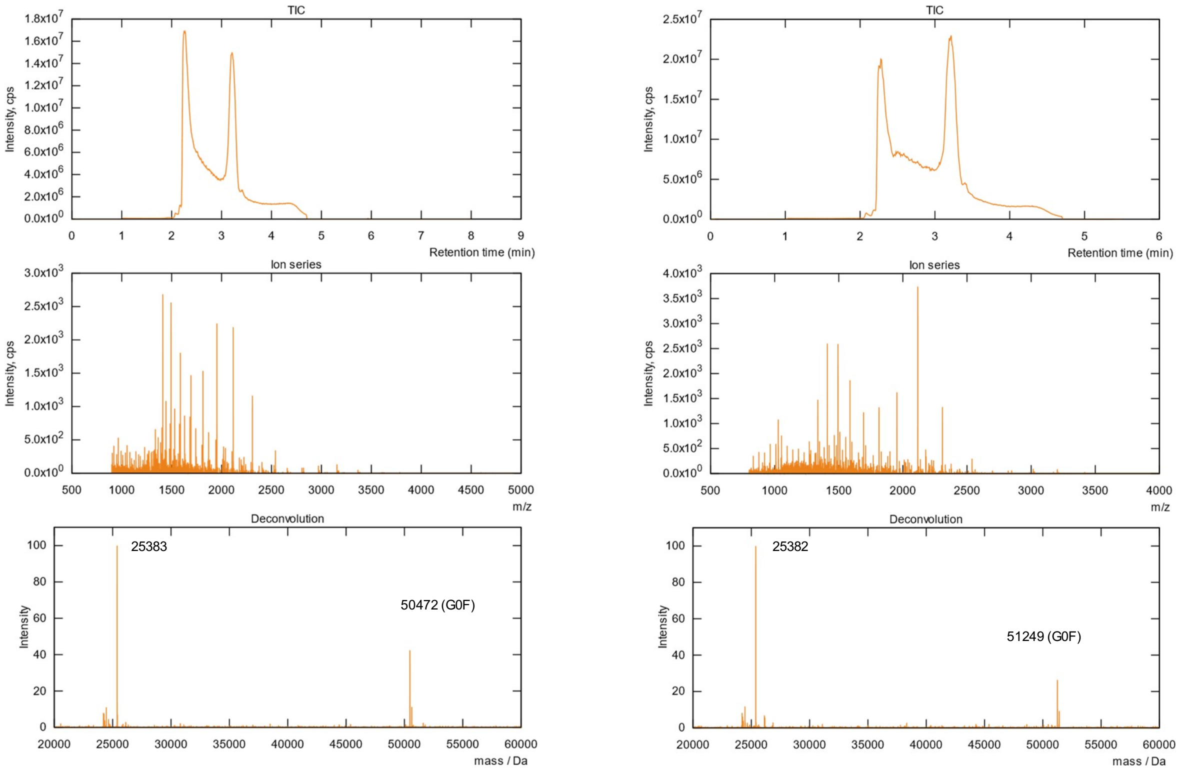

		Mass before reaction (Da)	Mass after reaction (Da)	Mass change (Da)
LC	Expected	25381	25381	0
	Observed	25383	25382	-1
HC	Expected	50470	51247	+777
	Observed	50472	51249	+777

Figure S70. UV chromatograms, ion series and deconvoluted mass spectra obtained from LC-MS analysis of antiCD33-(CPO-AF555)-(239iC) before and after modification.

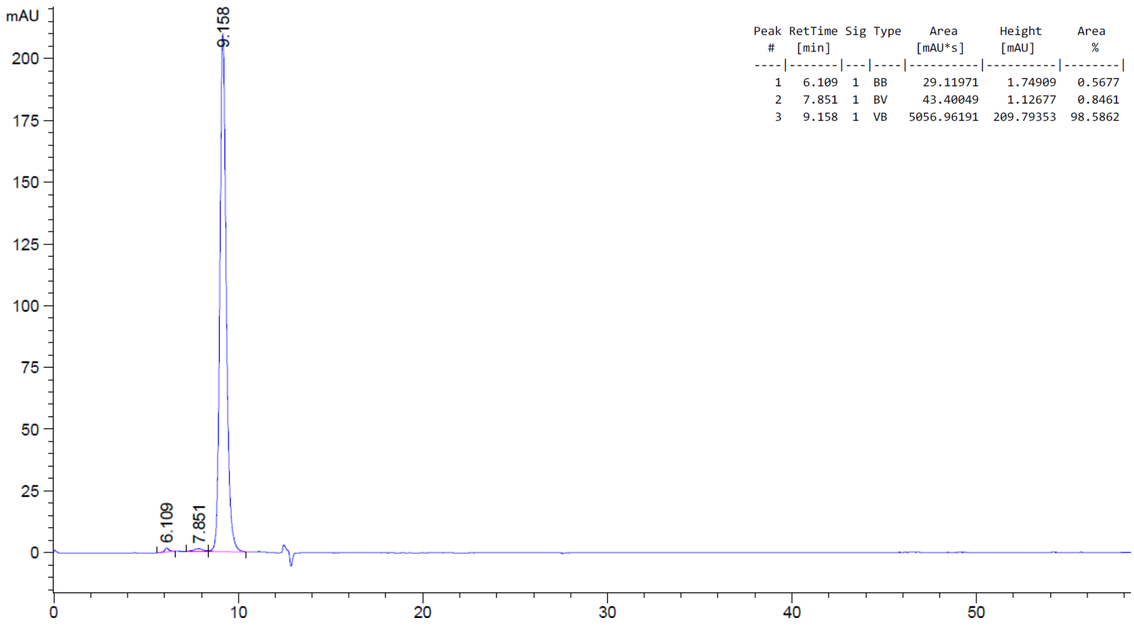

Peak #	RetTime [min]	Sig	Type	Area [mAU*s]	Height [mAU]	Area %
1	6.109	1	BB	29.11971	1.74909	0.5677
2	7.851	1	BV	43.40049	1.12677	0.8461
3	9.158	1	VB	5056.96191	209.79353	98.5862

Figure S71. UV chromatogram obtained from HP-SEC analysis.

i)

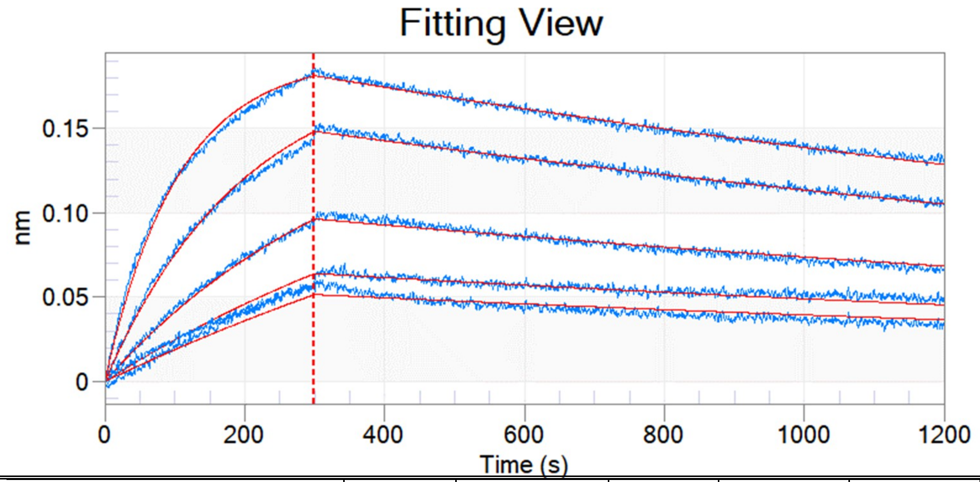

	K_D (M)	K_D Fitting Error (M)	K_{on} (1/Ms)	K_{on} error (1/Ms)	K_{dis} (1/s)	K_{dis} error (1/s)
antiCD33-(CPO-AF555)-(239iC-DL488)	2.07E-09	1.33E-11	1.85E+05	9.54E+02	3.82E-04	1.47E-06
antiCD33-(Wildtype)	8.68E-10	4.97E-12	2.63E+05	9.12E+02	2.28E-04	1.04E-06

ii)

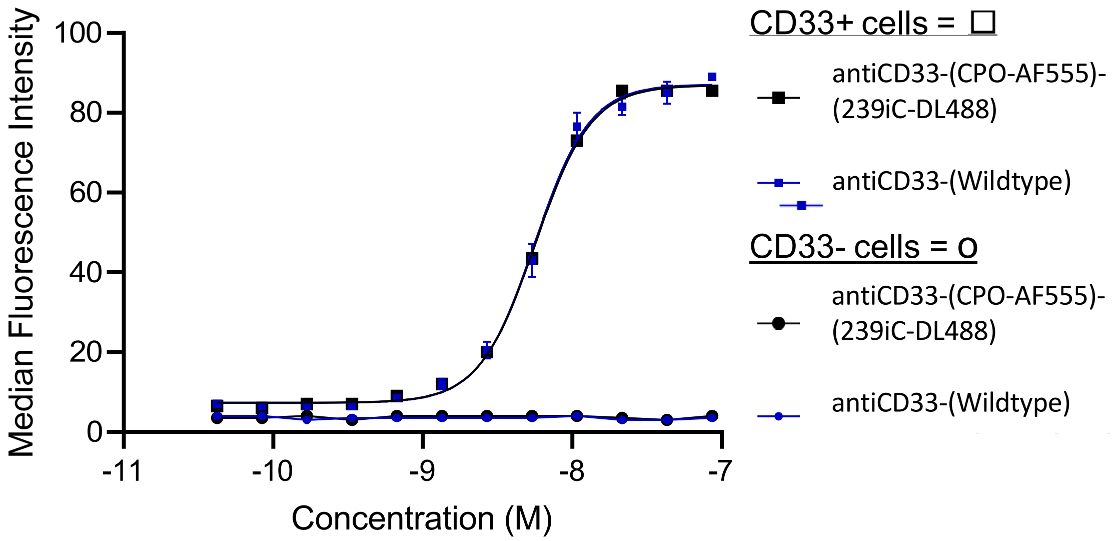

	EC_{50} (nM)	
	CD33-	CD33+
antiCD33-(Wildtype)	N/A	5.612 ± 0.361
antiCD33-(CPO-AF555)-(239iC-DL488)	N/A	5.673 ± 0.243

Figure S72. Biophysical analysis of antiCD33-(CPO-AF555)-(239iC-DL488). i) Binding curves and corresponding kinetic parameters obtained from BLI analysis. ii) Titrated FACS binding curve.

i)

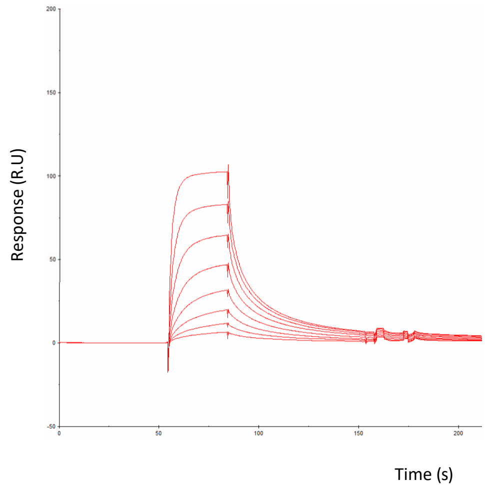

ii)

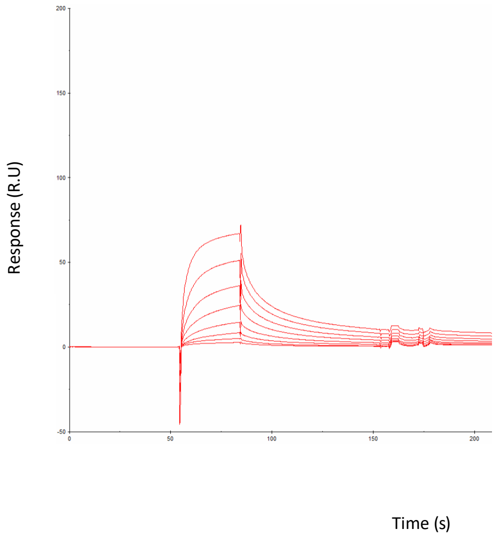

iii)

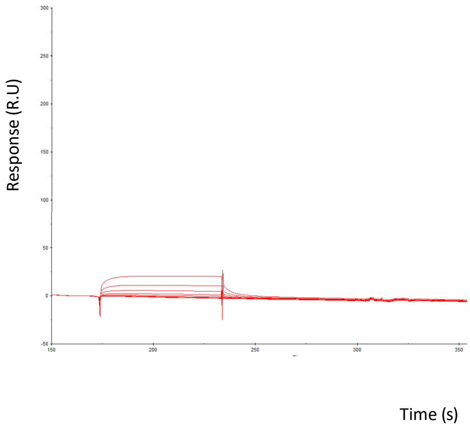

iv)

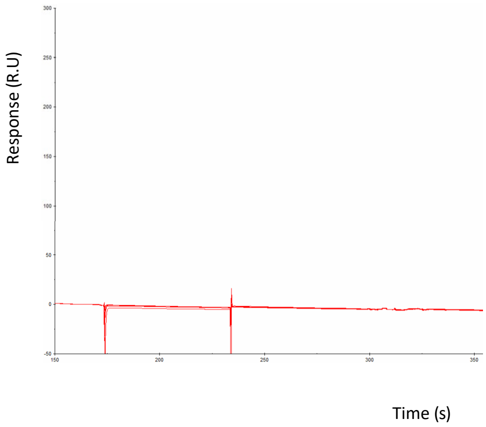

	**FcRn**		**FcγR**	
	**K_{D2} (nM)**	**STD (nM)**	**K_D (nM)**	**STD (nM)**
antiCD33-(Wildtype)	46	4.3	No binding	N/A
antiCD33-(CPO-AF555)-(239iC-DL488)	102	13.0	No binding	N/A

Figure S73. i) FcRn binding curve of antiCD33-(wildtype) ii) FcRn binding curve of antiCD33-(CPO- AF555)-(239iC-DL488) iii) FcγR binding curve of antiCD33-(wildtype) iv) FcγR binding curve of antiCD33- (CPO-AF555)-(239iC-DL488)

7.8 Dual modification: CPO and maleimide (T289C)

7.8.1 AntiCD33-(FLAG-Cys)-(T289C)

AntiCD33-(FLAG-Cys)-(T289C) was expressed and purified, following the protocol described in 6.2.4, achieving a post-purification yield of 26 mg L^{-1} (Normalised to WT = 29 mg L^{-1}). LC-MS and biophysical analysis were conducted to assess the identity and integrity of the product.

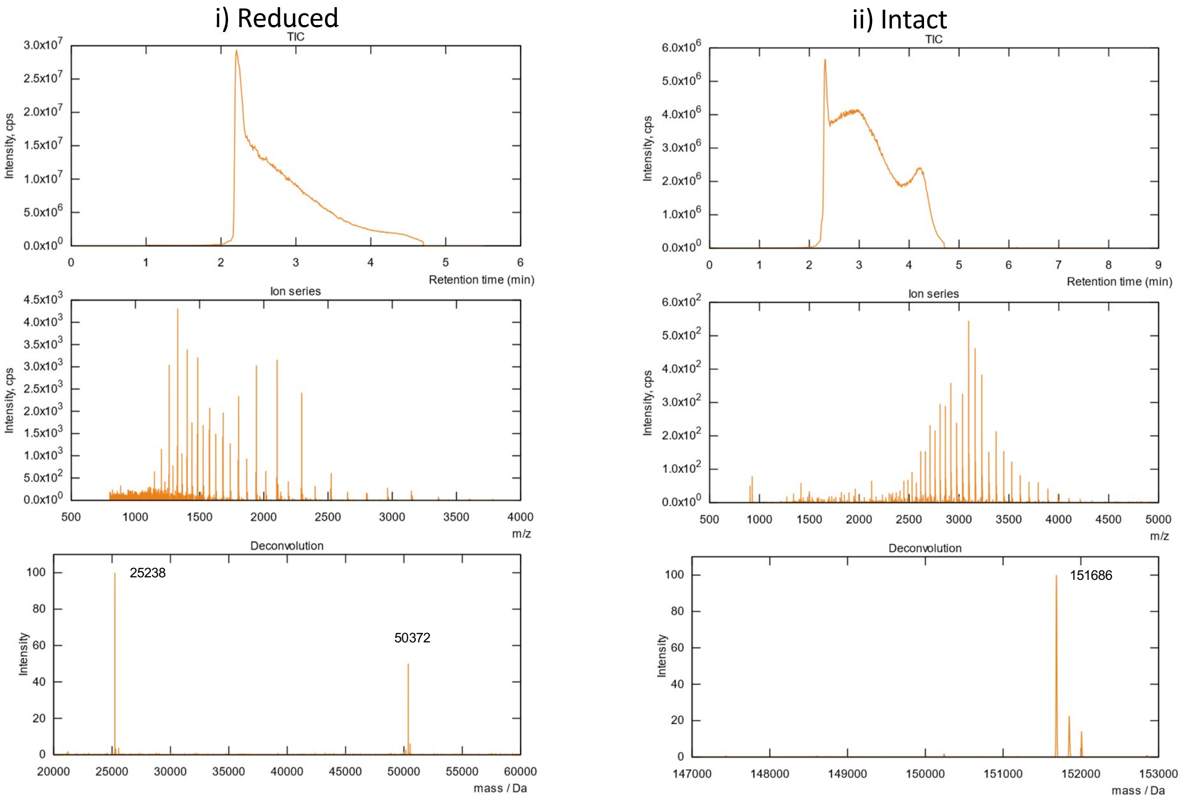

Ref.	Expected mass (Da)	Observed mass (Da)
i	LC: 25238<br>HC: 50369	LC: 25238<br>HC: 50372
ii	151682	151686

Figure S74. UV chromatograms, ion series and deconvoluted mass spectra obtained from LC-MS analysis of antiCD33-(FLAG-Cys)-(T289C) under reduced (i) and intact (ii) conditions.

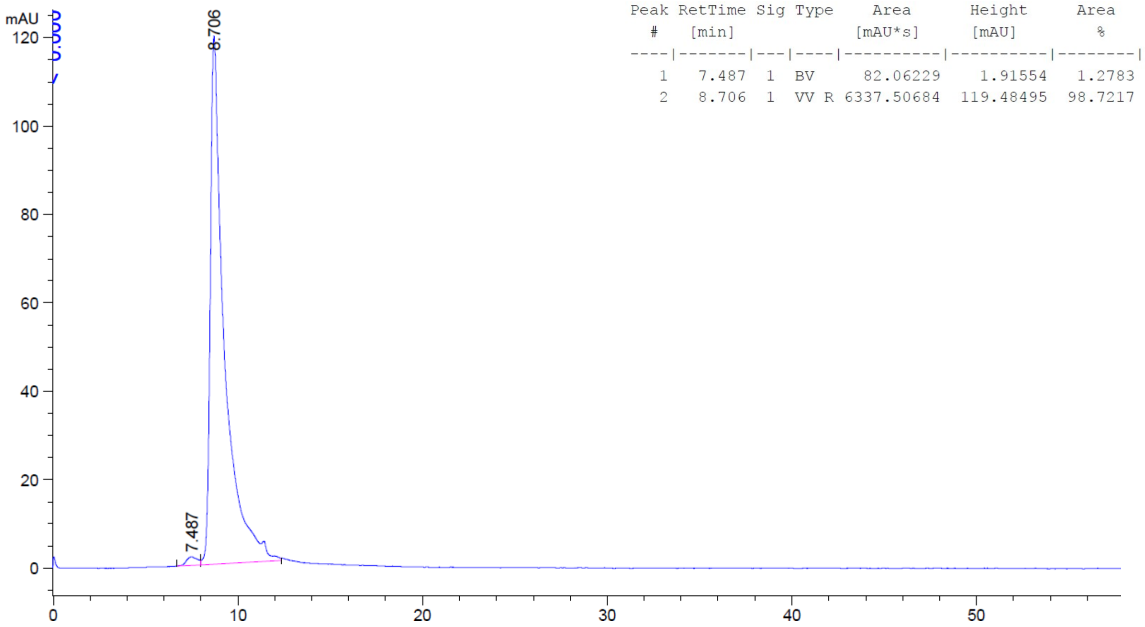

Peak #	RetTime [min]	Sig	Type	Area [mAU*s]	Height [mAU]	Area %
1	7.487	1	BV	82.06229	1.91554	1.2783
2	8.706	1	VV R	6337.50684	119.48495	98.7217

Figure S75. UV chromatogram obtained from HP-SEC analysis of antiCD33-(FLAG-Cys)-(T289C).

7.8.2 AntiCD33-(Cys)-(T289C)

The FLAG tag of AntiCD33-(FLAG-Cys)-(T289C) was removed following method “FLAG cleavage”, described in S6.2. The subsequent reaction mixture was desalted using an Amicon® Ultra 0.5 mL Centrifugal Filter (10K MWCO). The purified protein, termed antiCD33-(Cys)-(T289C), was then characterised by LC-MS, which confirmed successful cleavage of the FLAG tag and formation of an N-terminal cysteine.

antiCD33-(FLAG-Cys)-(T289C) —Enterokinase, 25 °C, 16 h→ antiCD33-(Cys)-(T289C)

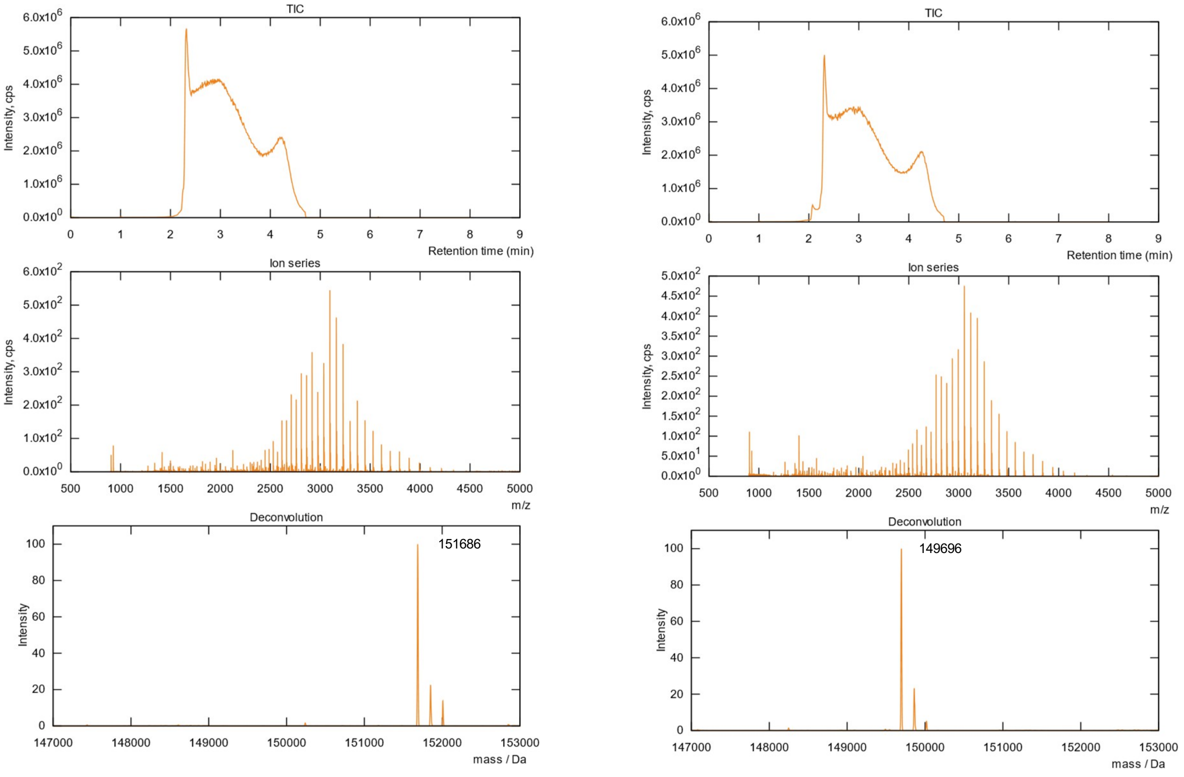

	Mass before Cleavage (Da)	Mass after Cleavage (Da)	Mass change (Da)
Expected	151682	149692	-1990
Observed	151686	149696	-1990

Figure S76. UV chromatograms, ion series and deconvoluted mass spectra obtained from LC-MS analysis of antiCD33-(FLAG-Cys)-(T289C) before and after cleavage.

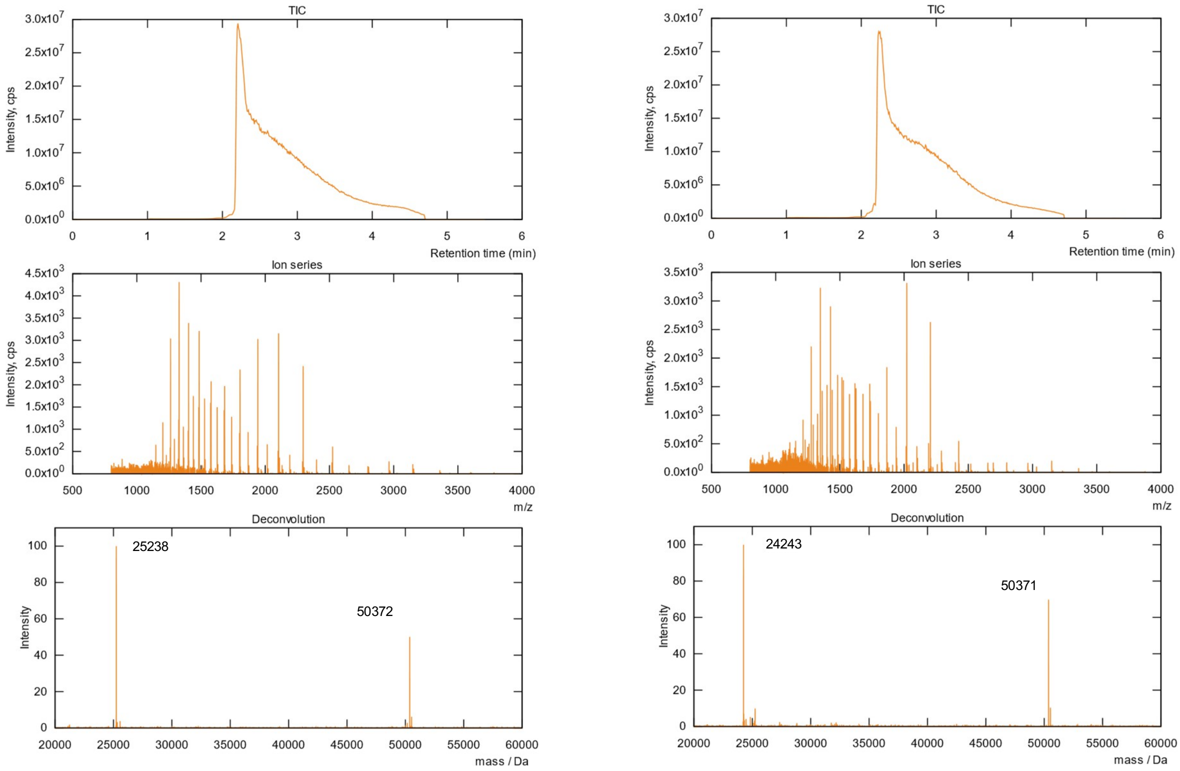

		Mass before cleavage (Da)	Mass after cleavage (Da)	Mass change (Da)
LC	Expected	25238	24243	-995
	Observed	25238	24243	-995
HC	Expected	50369	50369	0
	Observed	50372	50371	-1

Figure S77. UV chromatograms, ion series and deconvoluted mass spectra obtained from LC-MS analysis of antiCD33-(FLAG-Cys)-(T289C) before and after cleavage.

7.8.3 AntiCD33-(CPO-DBCO)-(T289C)

Conjugation of CPO-DBCO to antiCD33-(Cys)-(T289C) was performed following method “CPO conjugation” described in S6.5 using 2 mL of protein solution. The subsequent reaction mixture was purified using an Amicon® Ultra 0.5 mL Centrifugal Filter (10K MWCO). The purified protein, termed antiCD33-(CPO-DBCO)-(T289C), was characterised by LC-MS, which confirmed successful CPO-DBCO modification.

antiCD33-(Cys)-(T289C) —CPO-DBCO, 25 °C, 4 h→ antiCD33-(CPO-DBCO)-(T289C)

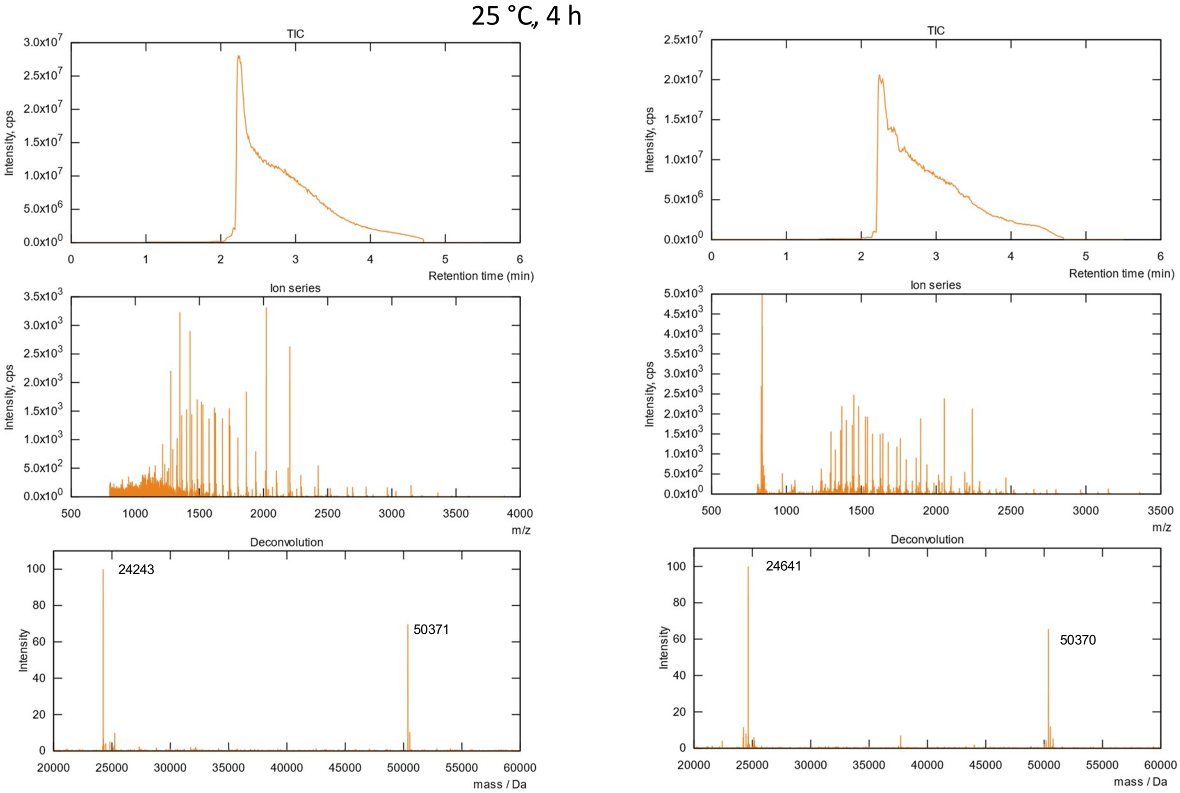

		Mass before reaction (Da)	Mass after reaction (Da)	Mass change (Da)
LC	Expected	24243	24641	398
	Observed	24243	24641	398
HC	Expected	50369	50369	0
	Observed	50371	50370	-1

Figure S78. UV chromatograms, ion series and deconvoluted mass spectra obtained from LC-MS analysis of antiCD33-(Cys)-(T289C) before and after modification.

7.8.4 AntiCD33-(CPO-AF555)-(T289C)

Conjugation of AzAF555 to AntiCD33-(CPO-DBCO)-(T289C) was performed following method “SPAAC conjugation” described in S6.9 using 2 mL of protein solution. The resulting solution was purified by SEC. The purified protein, termed antiCD33-(CPO-AF555)-(T289C) was characterised by LC-MS, which confirmed successful conjugation of AzAF555.

antiCD33-(CPO-DBCO)-(T289C) —AzAF555, 25 °C, 1 h→ antiCD33-(CPO-AF555)-(T289C)

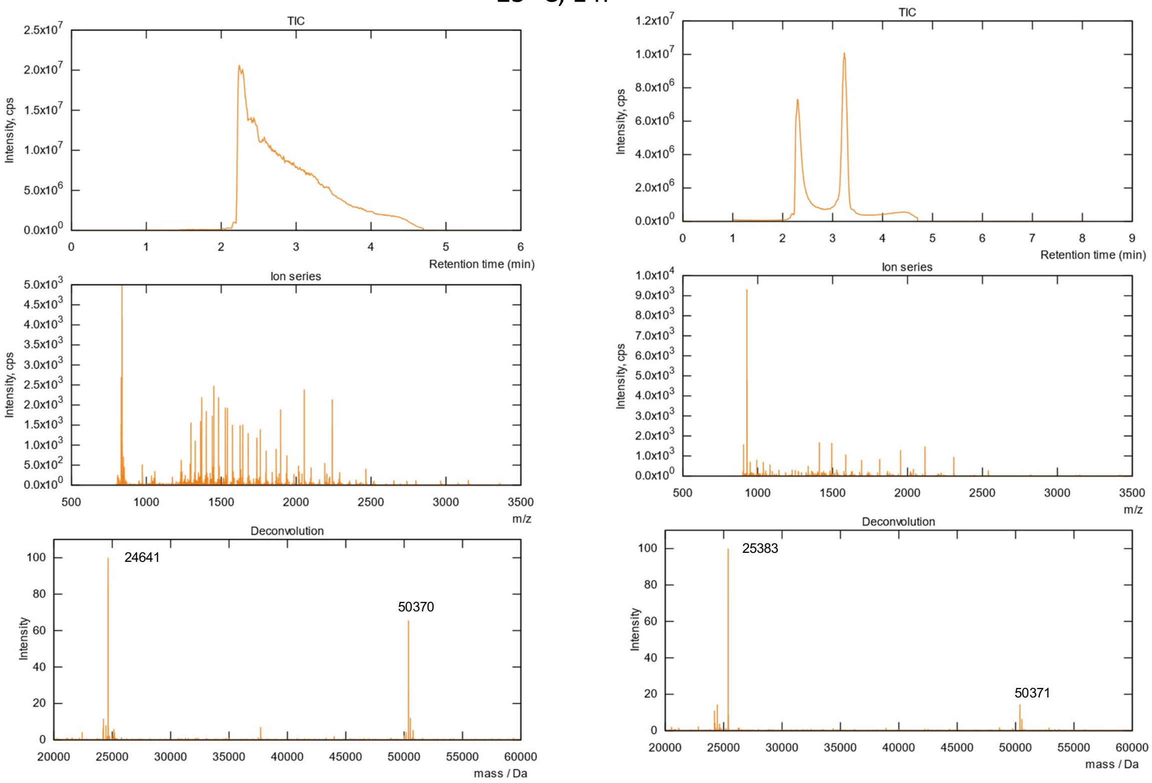

		Mass before reaction (Da)	Mass after reaction (Da)	Mass change (Da)
LC	Expected	24641	25381	+740
	Observed	24641	25383	+742
HC	Expected	50369	50369	0
	Observed	50370	50371	+1

Figure S79. UV chromatograms, ion series and deconvoluted mass spectra obtained from LC-MS analysis of antiCD33-(CPO-DBCO)-(T289C) before and after modification.

7.8.5 AntiCD33-(CPO-AF555)-(T289C-DL488)

Conjugation of DL488-Mal to AntiCD33-(CPO-AF555)-(T289C-DL488) was performed following method “Maleimide conjugation” described in S6.10 using 300 µL of protein solution. The resulting solution was desalted using an Amicon® Ultra 0.5 mL Centrifugal Filter (100K MWCO). LC-MS and biophysical analysis were conducted to assess the identity, integrity and functionality of the product, which was termed antiCD33-(CPO-AF555)-(T289C-DL488).

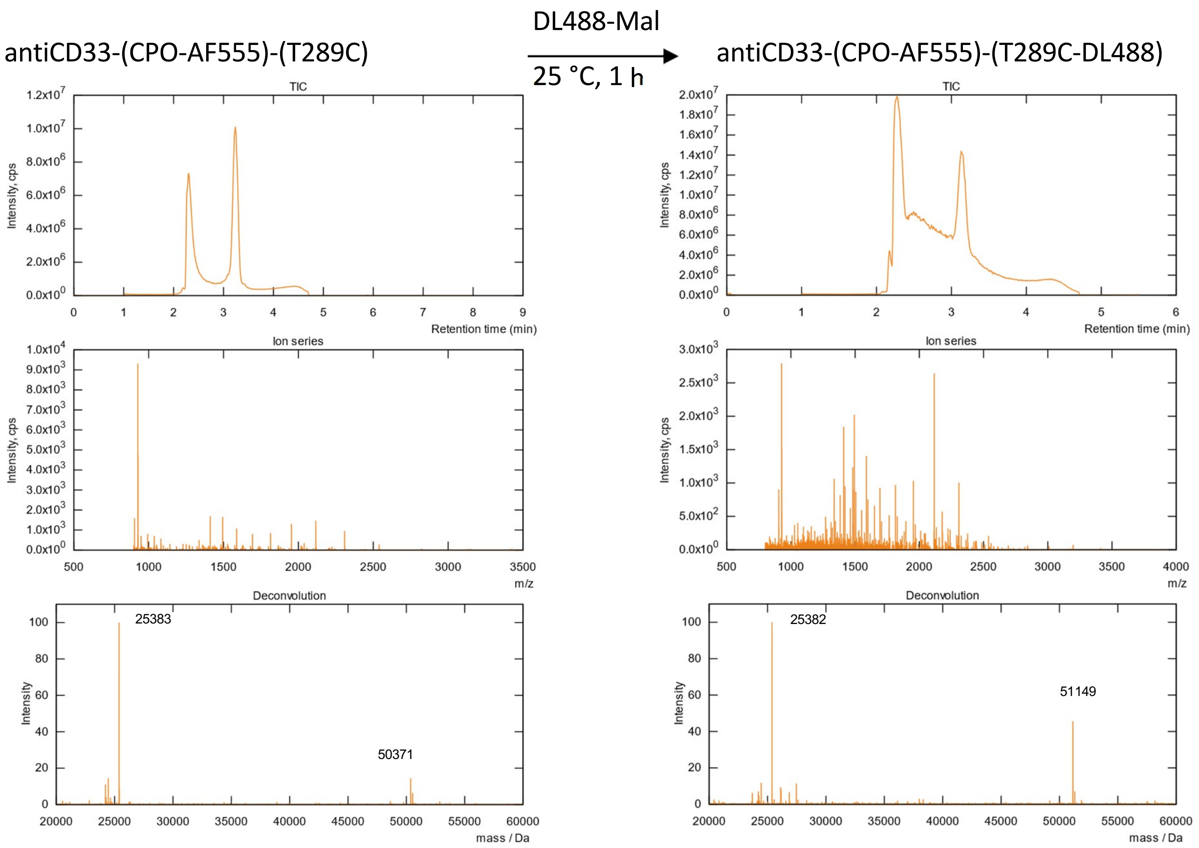

		Mass before reaction (Da)	Mass after reaction (Da)	Mass change (Da)
LC	Expected	25381	25381	0
	Observed	25383	25382	-1
HC	Expected	50369	51146	+777
	Observed	50371	51149	+778

Figure S80. UV chromatograms, ion series and deconvoluted mass spectra obtained from LC-MS analysis of antiCD33-(CPO-AF555)-(T289C) before and after modification.

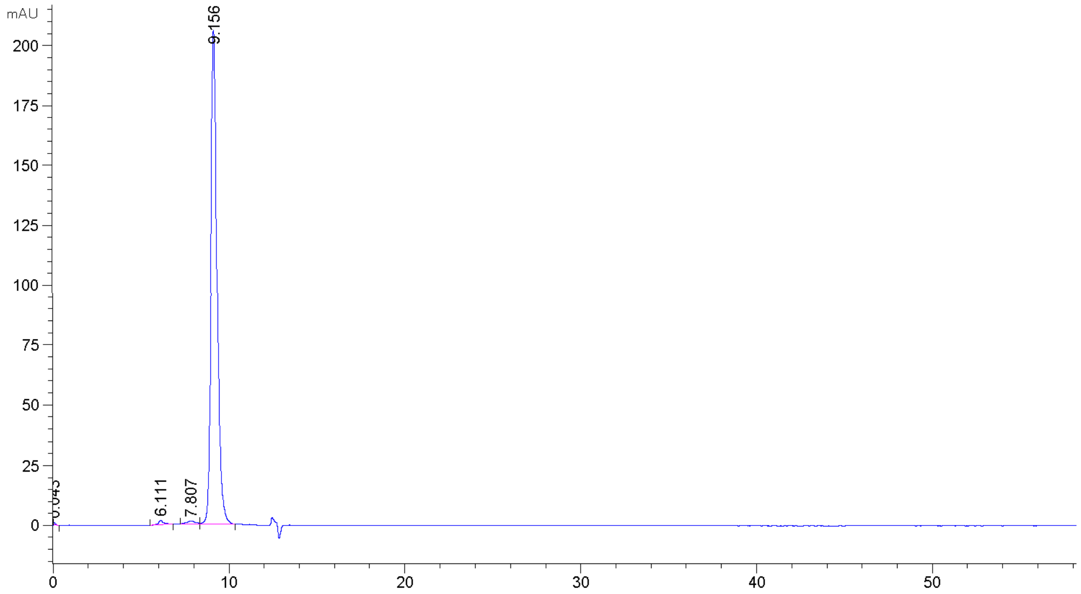

Figure S81. UV chromatogram obtained from HP-SEC analysis.

i)

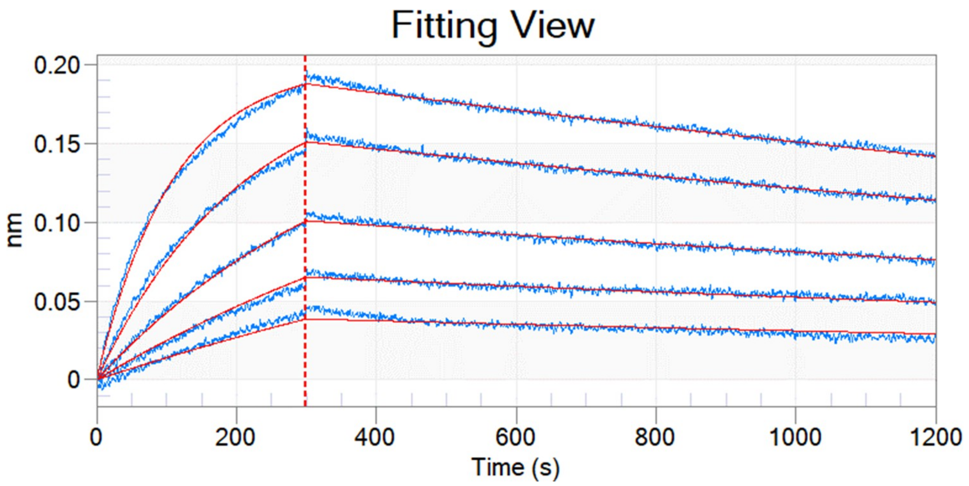

Ref.	K_D (M)	K_D Fitting Error (M)	Kon (1/Ms)	Kon error (1/Ms)	Kdis (1/s)	Kdis error (1/s)
antiCD33-(CPO-AF555)-(T289C-DL488)	1.75E-09	9.87E-12	1.78E+05	7.51E+02	3.13E-04	1.16E-06
antiCD33-(Wildtype)	8.68E-10	4.97E-12	2.63E+05	9.12E+02	2.28E-04	1.04E-06

ii)

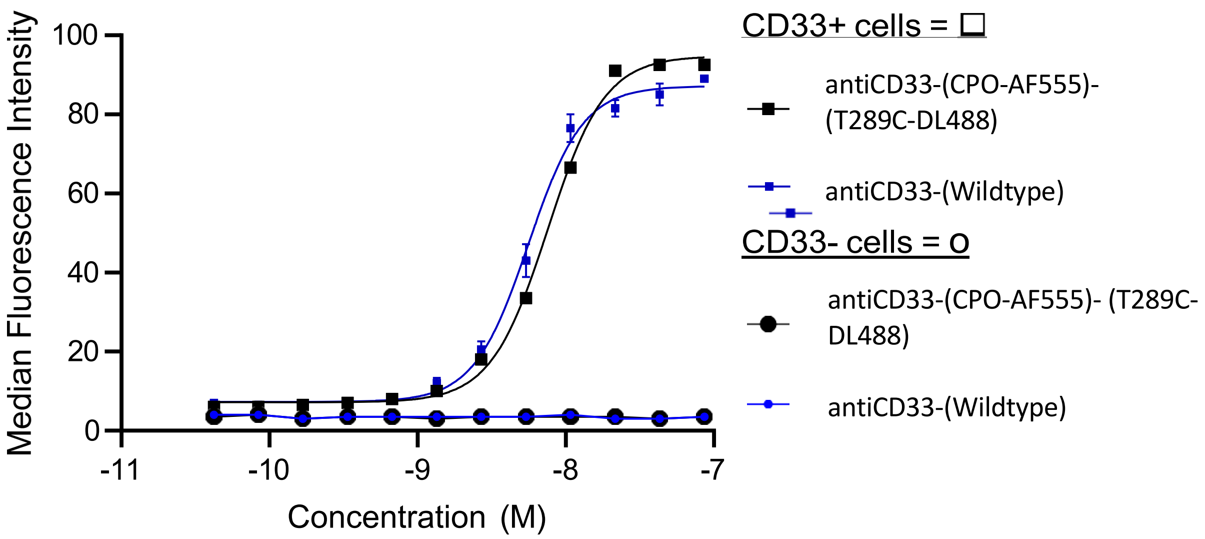

	EC50 (nM)	
	CD33-	CD33+
antiCD33-(Wildtype)	N/A	5.612 ± 0.361
antiCD33-(CPO-AF555)-(T289C-DL488)	N/A	7.565 ± 0.158

Figure S82. Biophysical analysis of antiCD33-(CPO-AF555)-(T289C-DL488). i) Binding curves and corresponding kinetic parameters obtained from BLI analysis. ii) Titrated FACS binding curve.

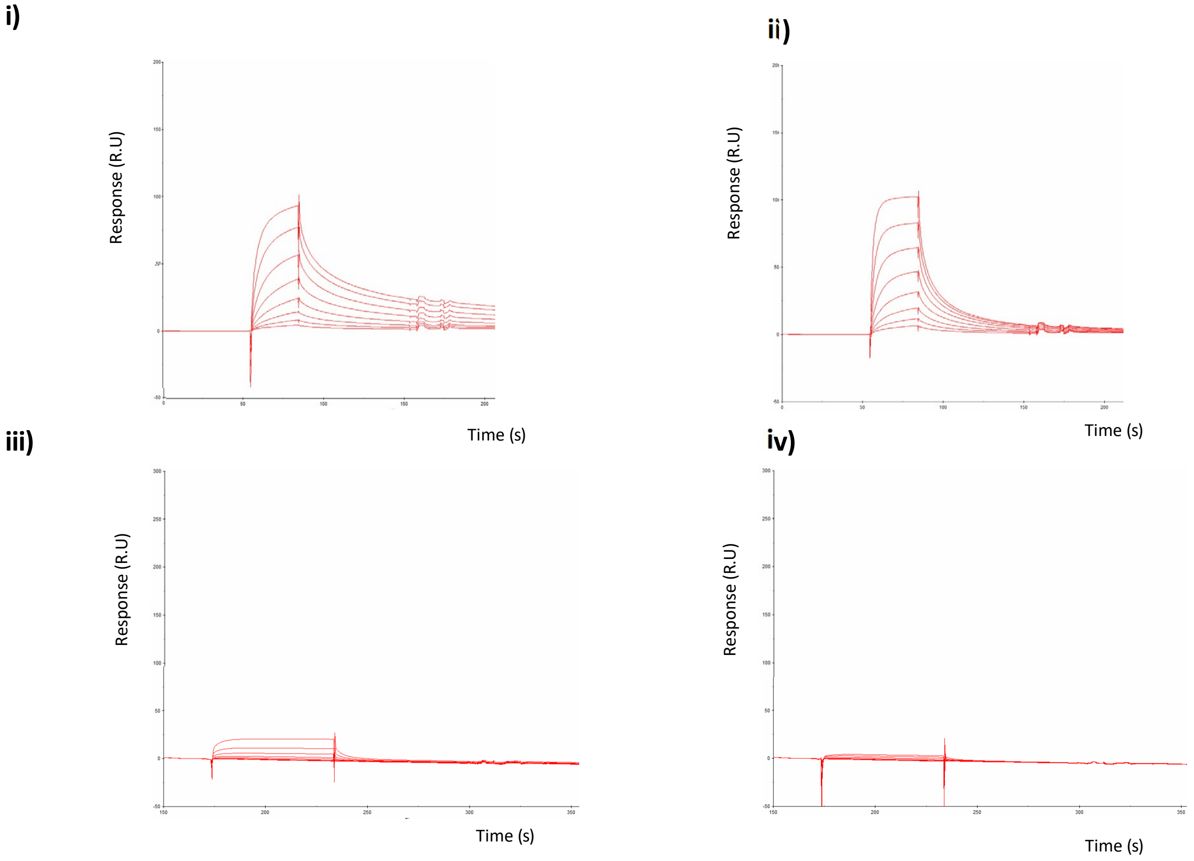

	FcRn		FcγR	
	K_{D2} (nM)	STD (nM)	K_D (nM)	STD (nM)
antiCD33-(wildtype)	46	4.3	No binding	N/A
antiCD33-(CPO-AF555)-(T289C-DL488)	62	8.7	No binding	N/A

Figure S83. i) FcRn binding curve of antiCD33-(wildtype) ii) FcRn binding curve of antiCD33-(CPO- AF555)-(T289C-DL488) iii) FcγR binding curve of antiCD33-(wildtype) iv) FcγR binding curve of antiCD33-(CPO-AF555)-(T289C-DL488).

7.9 Dual modification: CPO and maleimide (A327C)

7.9.1 AntiCD33-(FLAG-Cys)-(A327C)

AntiCD33-(FLAG-Cys)-(A327C) was expressed and purified, following the protocol described in 6.2.4, achieving a post-purification yield of 36 mg L^{-1} (Normalised to WT = 46 mg L^{-1}). LC-MS and biophysical analysis were conducted to assess the identity and integrity of the product.

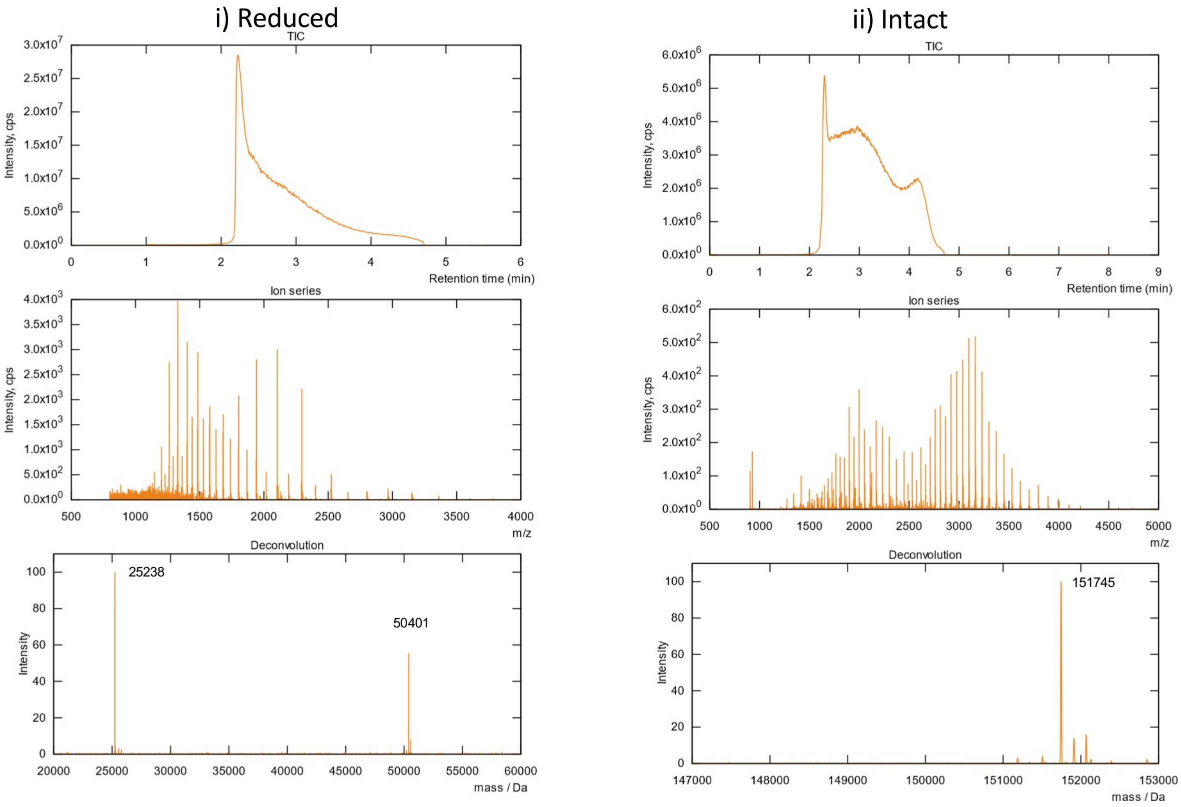

Ref.	Expected mass (Da)	Observed mass (Da)
i	LC: 25238<br>HC: 50399	LC: 25238<br>HC: 50401
ii	151742	151745

Figure S84. UV chromatograms, ion series and deconvoluted mass spectra obtained from LC-MS analysis of antiCD33-(FLAG-Cys)-(A327C) under reduced (i) and intact (ii) conditions.

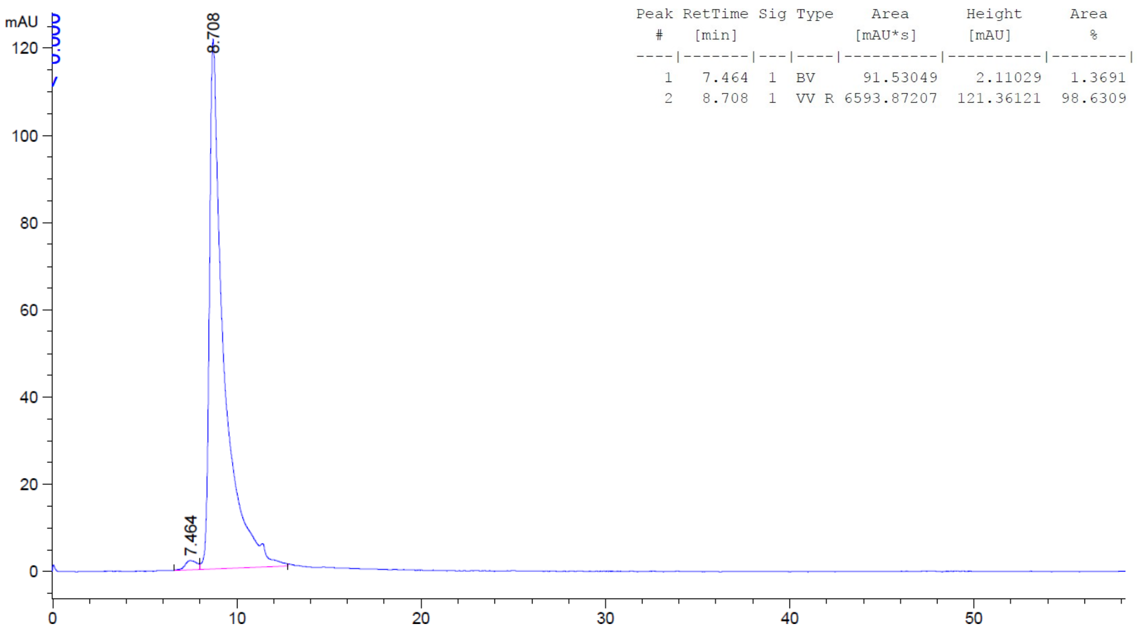

Figure S85. UV chromatogram obtained from HP-SEC analysis of antiCD33-(FLAG-Cys)-(A327C).

7.9.2 AntiCD33-(Cys)-(A327C)

The FLAG tag of AntiCD33-(FLAG-Cys)-(A327C) was removed following method “FLAG cleavage”, described in S6.2. The subsequent reaction mixture was desalted using an Amicon® Ultra 0.5 mL Centrifugal Filter (10K MWCO). The purified protein, termed antiCD33-(Cys)-(A327C), was then characterised by LC-MS, which confirmed successful cleavage of the FLAG tag and formation of an N-terminal cysteine.

antiCD33-(FLAG-Cys)-(A327C) —(Enterokinase; 25 °C, 16 h)→ antiCD33-(Cys)-(A327C)

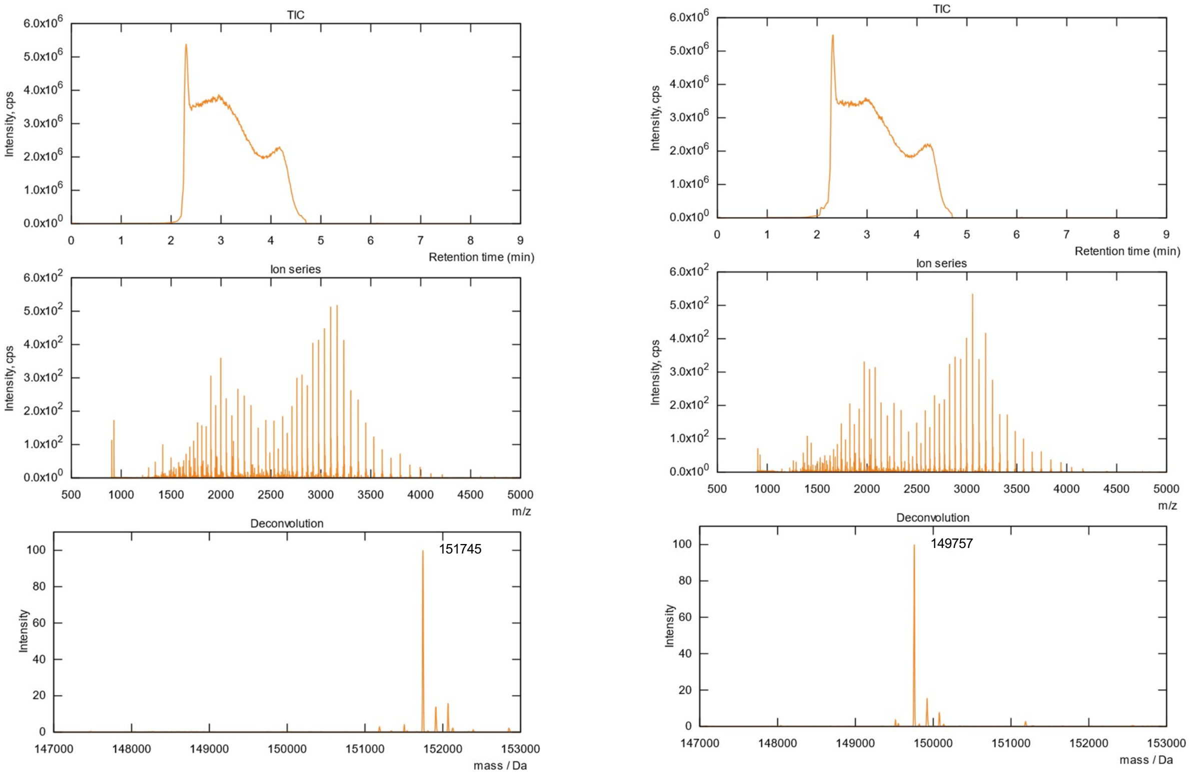

	Mass before Cleavage (Da)	Mass after Cleavage (Da)	Mass change (Da)
Expected	151742	149752	-1990
Observed	151745	149757	-1988

Figure S86. UV chromatograms, ion series and deconvoluted mass spectra obtained from LC-MS analysis of antiCD33-(FLAG-Cys)-(A327C) before and after cleavage.

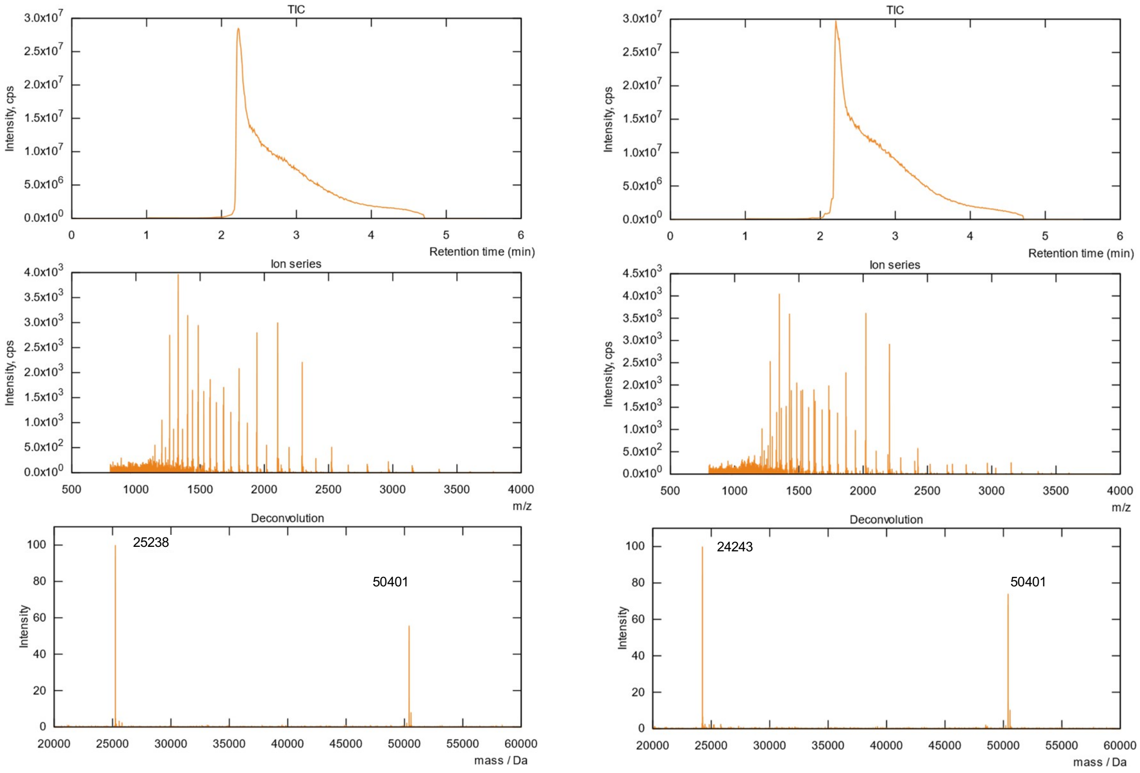

		Mass before cleavage (Da)	Mass after cleavage (Da)	Mass change (Da)
LC	Expected	25238	24243	-995
	Observed	25238	24243	-995
HC	Expected	50399	50399	0
	Observed	50401	50401	0

Figure S87. UV chromatograms, ion series and deconvoluted mass spectra obtained from LC-MS analysis of antiCD33-(FLAG-Cys)-(A327C) before and after cleavage.

7.9.3 AntiCD33-(CPO-DBCO)-(A327C)

Conjugation of CPO-DBCO to antiCD33-(Cys)-(A327C-HC) was performed following method "CPO conjugation" described in S6.5 using 2 mL of protein solution. The subsequent reaction mixture was purified using an Amicon® Ultra 0.5 mL Centrifugal Filter (10K MWCO). The purified protein, termed antiCD33-(CPO-DBCO)-(A327C), was characterised by LC-MS, which confirmed successful CPO-DBCO modification.

antiCD33-(Cys)-(A327C-HC) —(CPO-DBCO; 25 °C, 4 h)→ antiCD33-(CPO-DBCO)-(A327C)

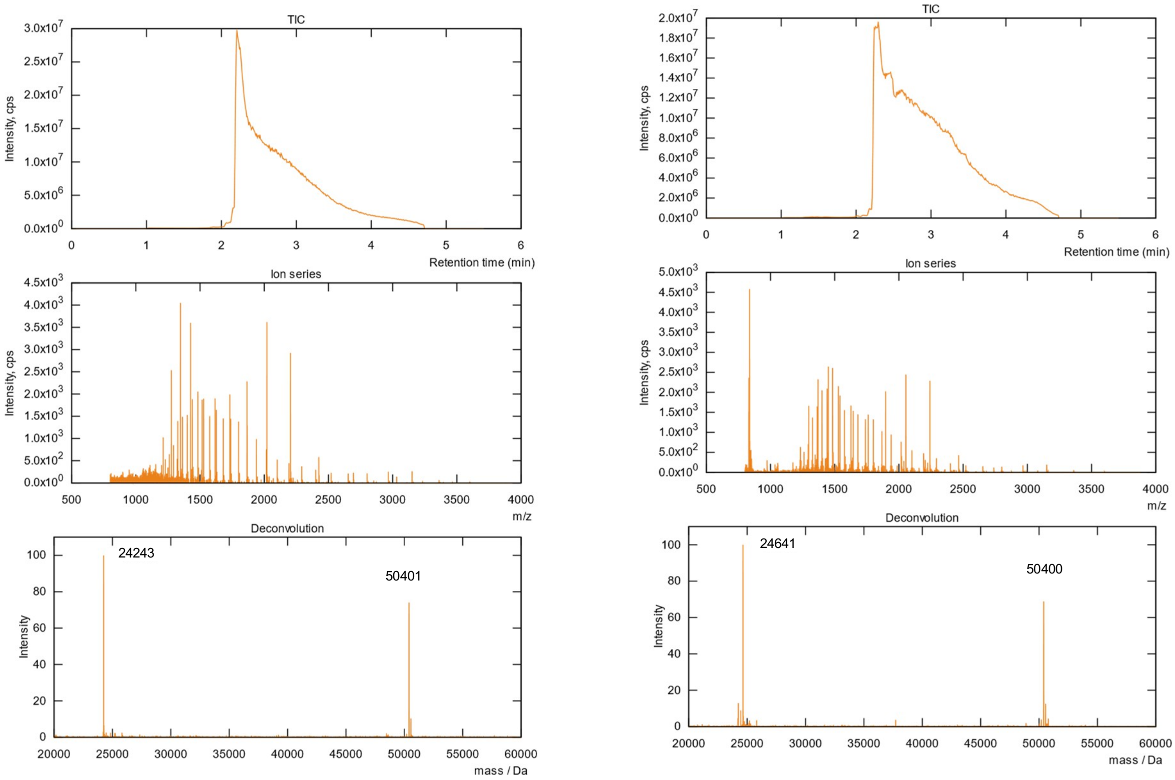

		Mass before reaction (Da)	Mass after reaction (Da)	Mass change (Da)
LC	Expected	24243	24641	398
	Observed	24243	24641	398
HC	Expected	50399	50399	0
	Observed	50401	50400	-1

Figure S88. UV chromatograms, ion series and deconvoluted mass spectra obtained from LC-MS analysis of antiCD33-(Cys)-(A327C) before and after modification.

7.9.4 AntiCD33-(CPO-AF555)-(A327C)

Conjugation of AzAF555 to AntiCD33-(CPO-DBCO)-(A327C) was performed following method “SPAAC conjugation” described in S6.9 using 2 mL of protein solution. The resulting solution was purified by SEC. The purified protein, termed antiCD33-(CPO-AF555)-(A327C) was characterised by LC-MS, which confirmed successful conjugation of AzAF555.

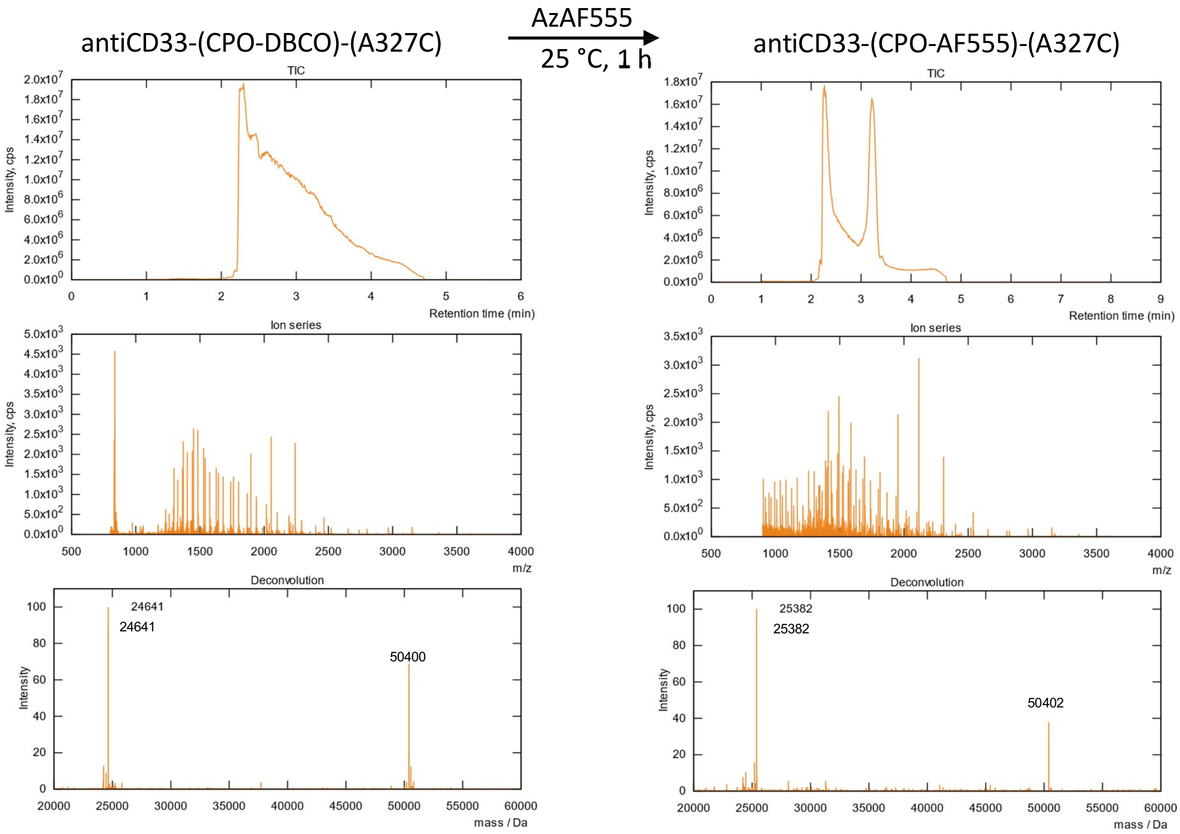

		Mass before reaction (Da)	Mass after reaction (Da)	Mass change (Da)
LC	Expected	24641	25381	+740
	Observed	24641	25382	+741
HC	Expected	50399	50399	0
	Observed	50400	50402	+2

Figure S89. UV chromatograms, ion series and deconvoluted mass spectra obtained from LC-MS analysis of antiCD33-(CPO-DBCO)-(A327C) before and after modification.

7.9.5 AntiCD33-(CPO-AF555)-(A327C-DL488)

Conjugation of DL488-Mal to AntiCD33-(CPO-AF555)-(A327C) was performed following method “Maleimide conjugation” described in S6.10 using 300 µL of protein solution. The resulting solution was desalted using an Amicon® Ultra 0.5 mL Centrifugal Filter (100K MWCO). LC-MS and biophysical analysis were conducted to assess the identity, integrity and functionality of the purified protein, termed antiCD33-(CPO-AF555)-(A327C-DL488).

antiCD33-(CPO-AF555)-(A327C) —(DL488-Mal; 25 °C, 1 h)→ antiCD33-(CPO-AF555)-(A327C-DL488)

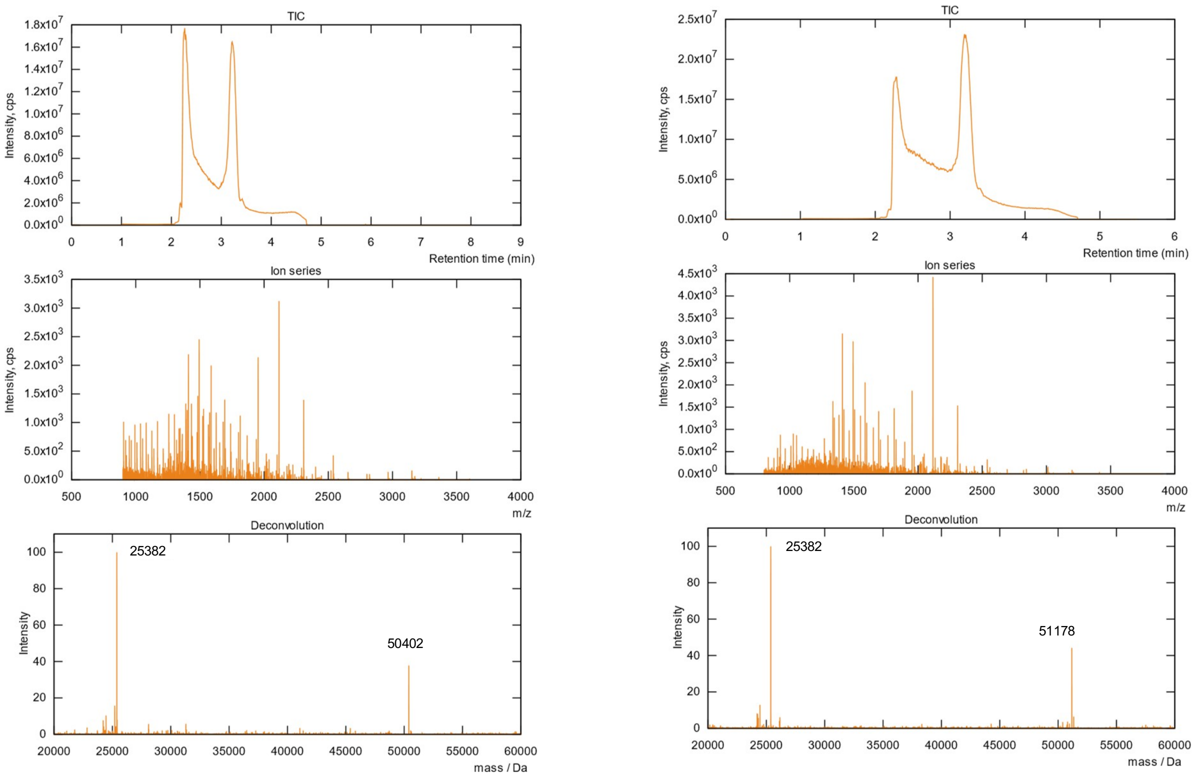

		Mass before reaction (Da)	Mass after reaction (Da)	Mass change (Da)
LC	Expected	25381	25381	0
	Observed	25382	25382	0
HC	Expected	50399	51176	+777
	Observed	50402	51178	+776

Figure S90. UV chromatograms, ion series and deconvoluted mass spectra obtained from LC-MS analysis of antiCD33-(CPO-AF555)-(A327C) before and after modification.

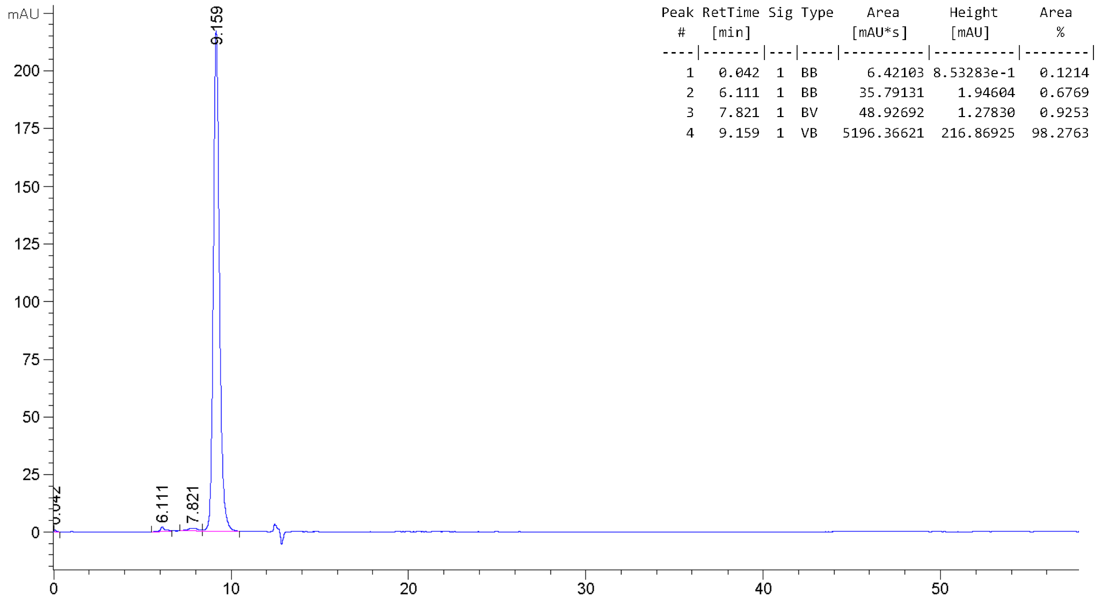

Peak #	RetTime [min]	Sig	Type	Area [mAU*s]	Height [mAU]	Area %
1	0.042	1	BB	6.42103	8.53283e-1	0.1214
2	6.111	1	BB	35.79131	1.94604	0.6769
3	7.821	1	BV	48.92692	1.27830	0.9253
4	9.159	1	VB	5196.36621	216.86925	98.2763

Figure S91. UV chromatogram obtained from HP-SEC analysis.

i)

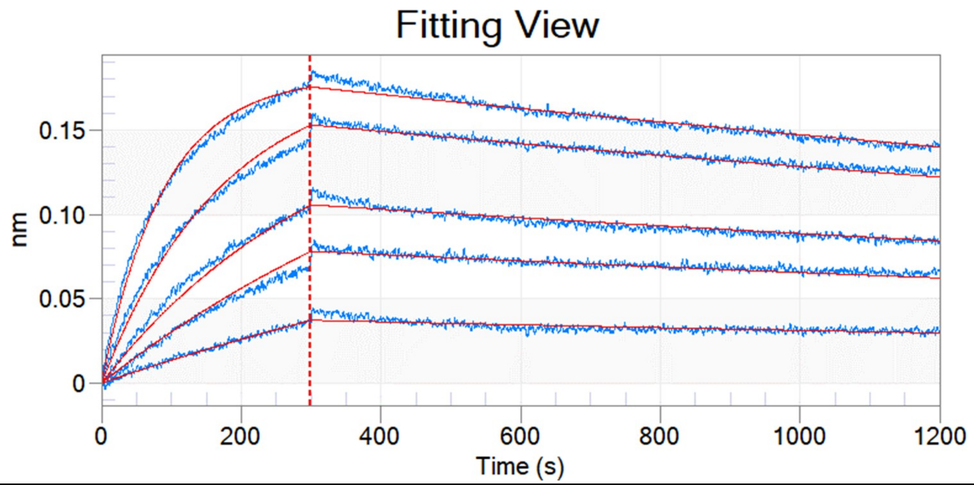

Ref.	K_D (M)	K_D Fitting Error (M)	K_{on} (1/Ms)	K_{on} error (1/Ms)	K_{dis} (1/s)	K_{dis} error (1/s)
antiCD33-(CPO-AF555)-(A327C-DL488)	1.14E-09	8.09E-12	2.20E+05	1.03E+03	2.51E-04	1.34E-06
antiCD33-(wildtype)	8.68E-10	4.97E-12	2.63E+05	9.12E+02	2.28E-04	1.04E-06

ii)

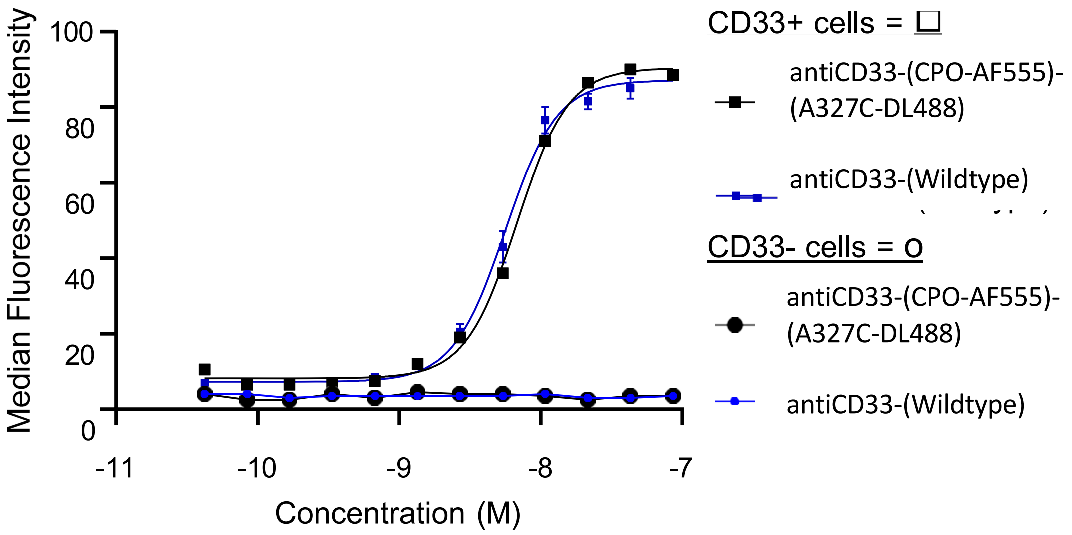

	EC_{50} (nM)	
	CD33-	CD33+
antiCD33-(Wildtype)	N/A	5.612 ± 0.361
antiCD33-(CPO-AF555)-(A327C-DL488)	N/A	6.793 ± 0.021

Figure S92. Biophysical analysis of antiCD33-(CPO-AF555)-(A327C-DL488). i) Binding curves and corresponding kinetic parameters obtained from BLI analysis. ii) Titrated FACS binding curve.

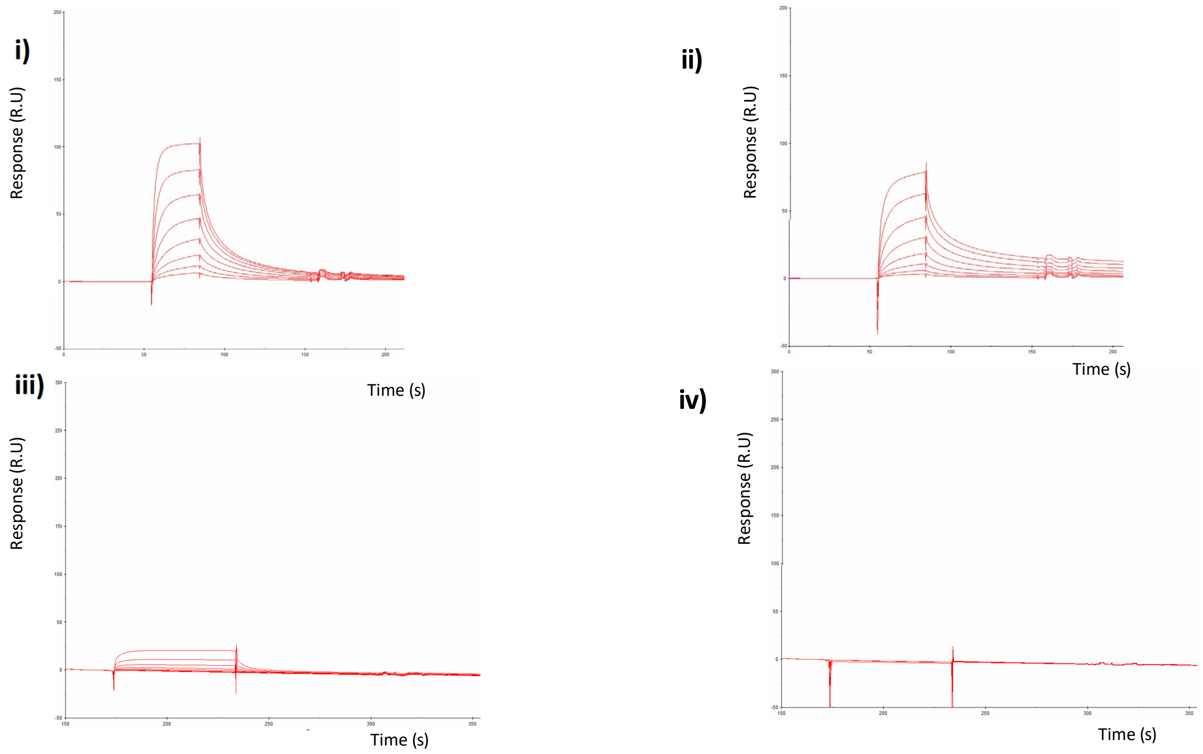

	FcRn		FcγR	
	K_{D2} (nM)	STD (nM)	K_D (nM)	STD (nM)
antiCD33-(wildtype)	46	4.3	No binding	N/A
antiCD33-(CPO-AF555)-(A327C-DL488)	77	6.8	No binding	N/A

Figure S93. i) FcRn binding curve of antiCD33-(wildtype) ii) FcRn binding curve of antiCD33-(CPO- AF555)-(A327C-DL488) iii) FcγR binding curve of antiCD33-(wildtype) iv) FcγR binding curve of antiCD33-(CPO-AF555)-(A327C-DL488).

7.10 Dual modification: Sortase and maleimide (239iC)

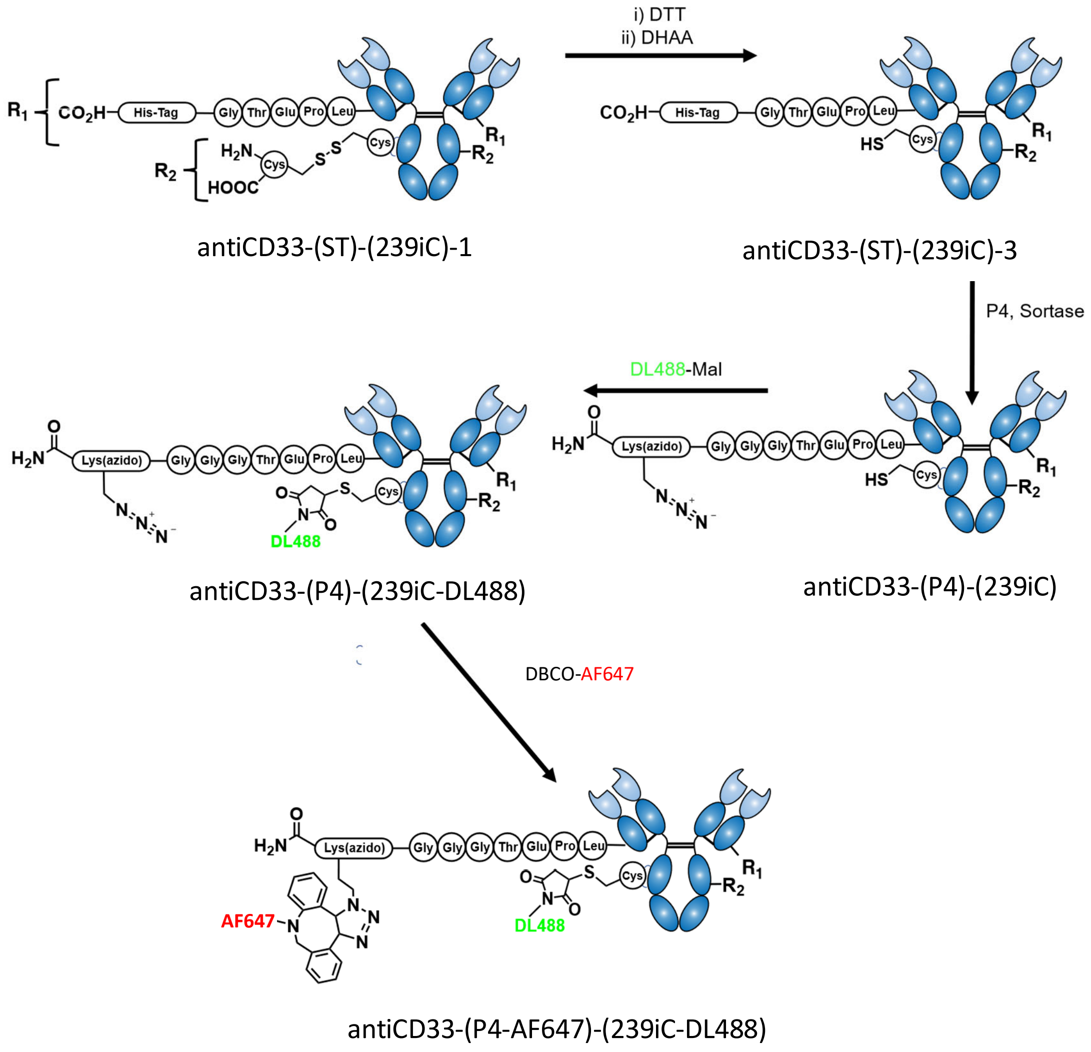

Figure S94. Schematic of the sortase and maleimide (239iC) based modification protocol.

7.10.1 AntiCD33-(ST)-(239iC)-1

AntiCD33-(ST)-(239iC)-1 was expressed and purified, following the protocol described in 6.2.4, achieving a post-purification yield of 61 mg L^{-1} [1] (normalization factor from antiCD33-(Wildtype) parallel expression: 1.25, normalised yield: 76 mg L^{-1}). LC-MS and biophysical analysis were conducted to assess the identity and integrity of the product.

i) Reduced

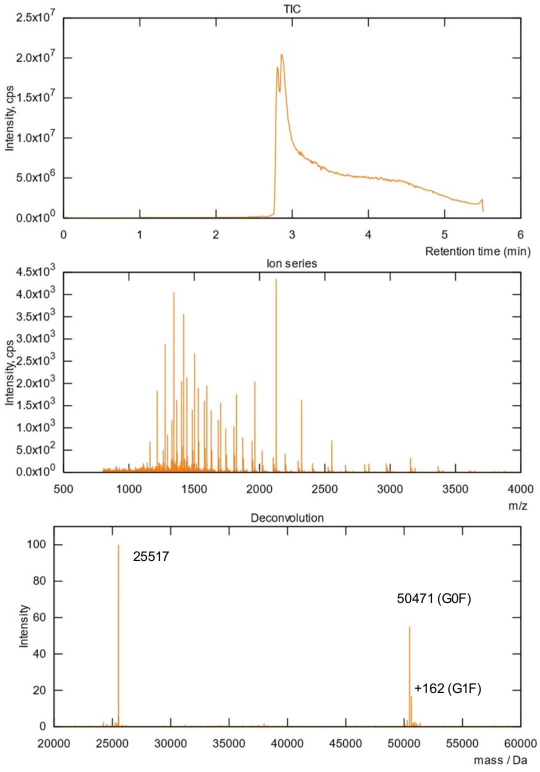

ii) Deglycosylated + Reduced

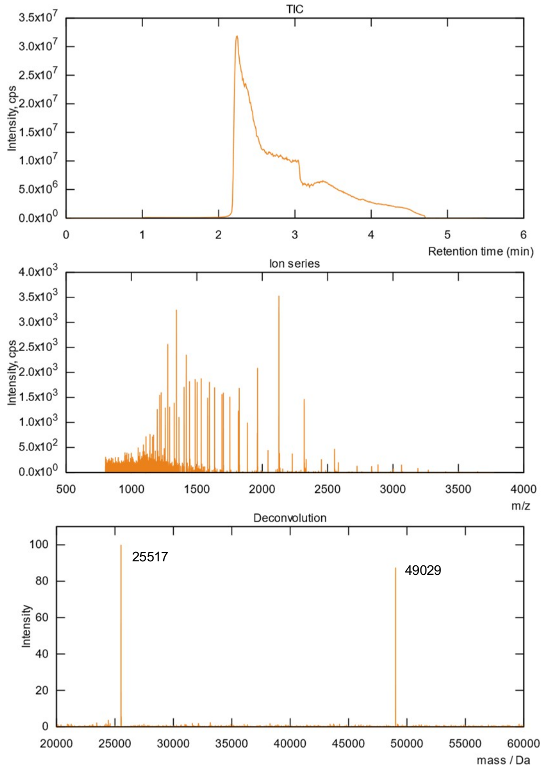

iii) Intact

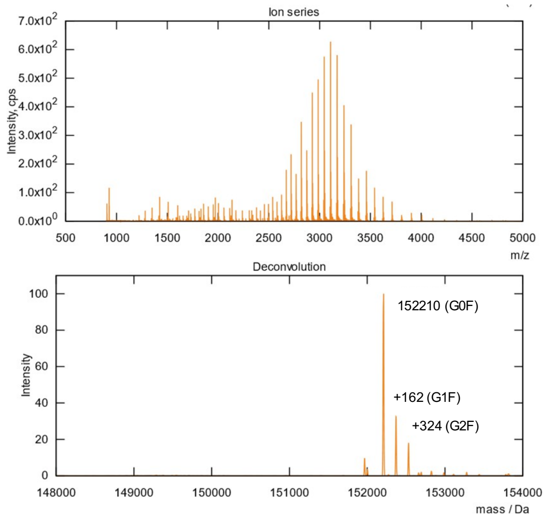

Ref.	Expected mass (Da)	Observed mass (Da)
i	LC: 25516<br>HC: 50470	LC: 25517<br>HC: 50471
ii	LC: 25516<br>HC: 49025	LC: 25517<br>HC: 49029
iii	152205	152210

Figure S95. UV chromatograms, ion series and deconvoluted mass spectra obtained from LC-MS analysis of antiCD33-(ST)- (239iC)-1 under reducing (i), deglycosylated and reducing (ii) and intact (iii) conditions.

i)

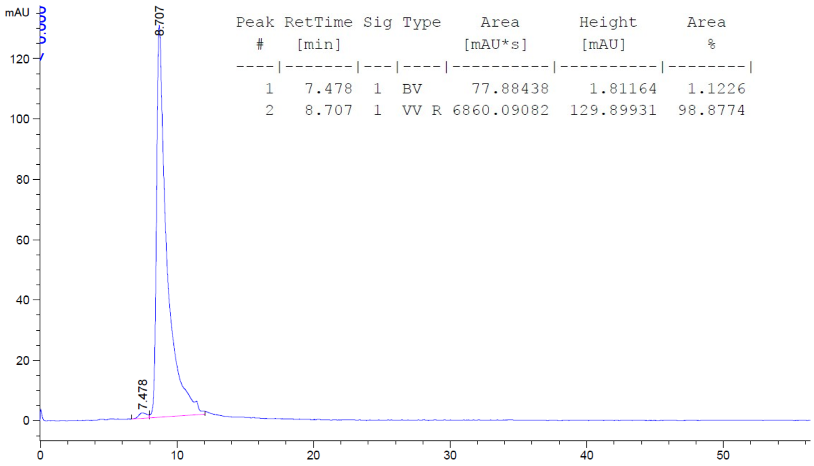

ii)

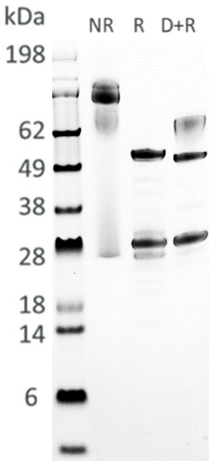

Figure S96. Biophysical analysis of antiCD33-(ST)-(239iC)-1. i) UV chromatogram obtained from HP-SEC analysis, ii) Image of SDS-PAGE gel stained with Coomassie (NR: non-reducing, R: reducing, D+R: Deglycosylated and reducing).

7.10.2 AntiCD33-(ST)-(239iC)-3

AntiCD33-(ST)-(239iC)-1 was decysteinylated and rebridged following method "Decysteinylation and rebridging" described in 6.4.2.11. The subsequent reaction mixture was desalted using an Amicon® Ultra 0.5 mL Centrifugal Filter (10K MWCO). The purified protein, termed antiCD33-(ST)-(239iC)-3, was analysed by LC-MS. To ensure rebridging had been successful, an aliquot (25 µL) of antiCD33-(ST)-(239iC)-3 was placed into a 0.5 mL Eppendorf containing methyl-PEG$_{24}$-maleimide (SigmaAldrich, 22713, 10 equiv.). Following incubation at 25 °C for 1 hour, the resulting protein solution, termed antiCD33-(ST)-(239iC)-4, was desalted using an Amicon® Ultra 0.5 mL Centrifugal Filter (10K MWCO) and analysed by LC-MS.

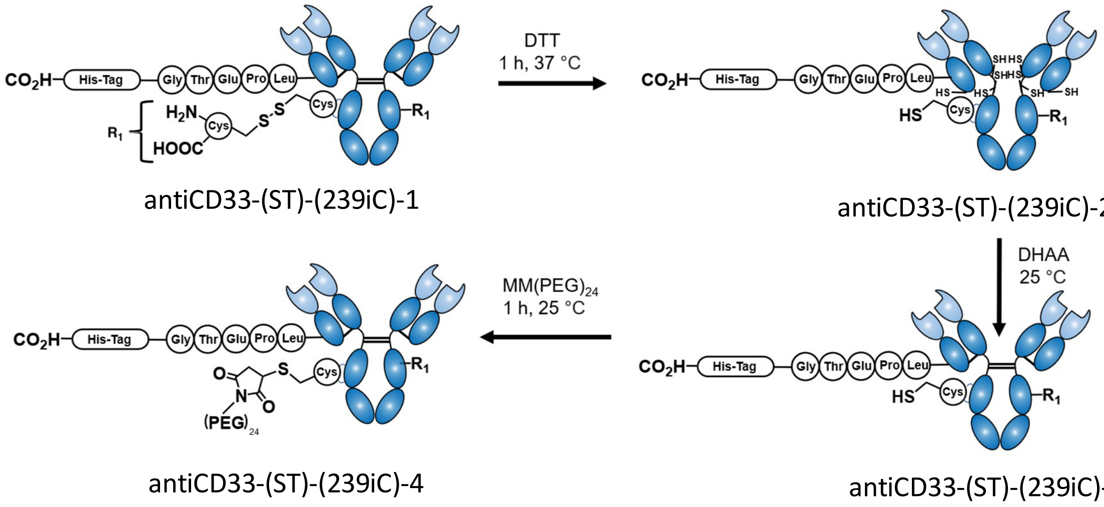

Figure S97. Summary of the reaction protocol for decapping and rebridging antiCD33-(ST)- (239iC)-1

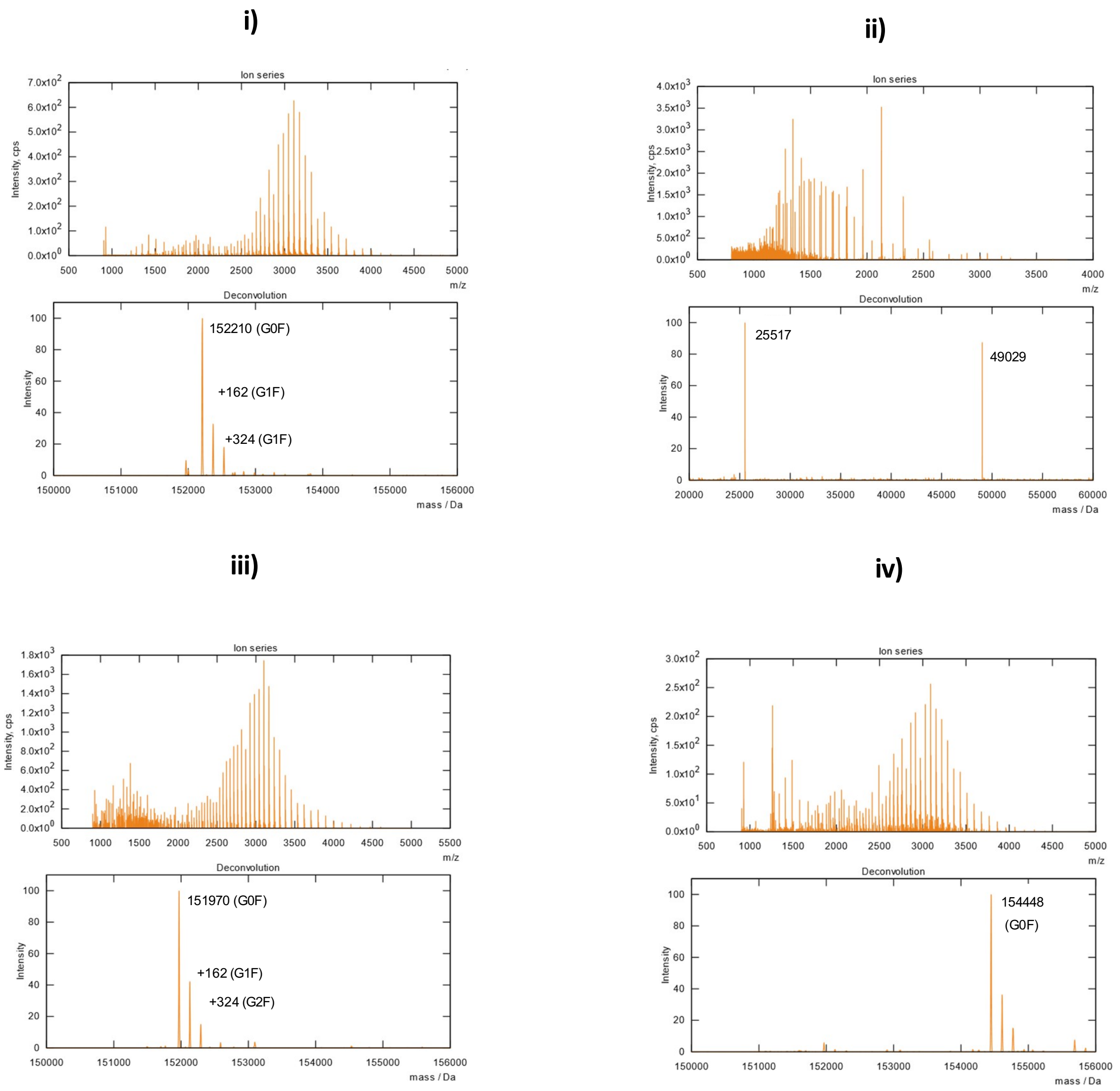

	Expected mass (Da)	Observed mass (Da)
**i**	152205	152210
**ii**	LC: 25516<br>HC: 50470	LC: 25517<br>HC: 50471
**iii**	151967	151970
**iv**	154445	154448

Figure S98. Ion series and deconvoluted mass spectra obtained from LC-MS analysis of i) antiCD33-(ST)-(239iC)-1, ii) antiCD33-(ST)-(239iC)-2, iii) antiCD33-(ST)-(239iC)-3, iv) antiCD33-(ST)- (239iC)-4.

7.10.3 AntiCD33-(P4)-(239iC)

Conjugation of P4 to antiCD33-(ST)-(239iC)-3 was performed following method “Sortase-mediated conjugation” described in S6.6 using 200 µL of protein solution.. The resulting solution was then purified using Amicon® Ultra 0.5 mL Centrifugal Filters (10K MWCO). The purified product, termed antiCD33-(P4)-(239iC), was subjected to LC-MS analysis, which confirmed successful modification.

antiCD33-(ST)-(239iC)-3 → (P4, Sortase; 37 °C, 1 h) antiCD33-(P4)-(239iC)

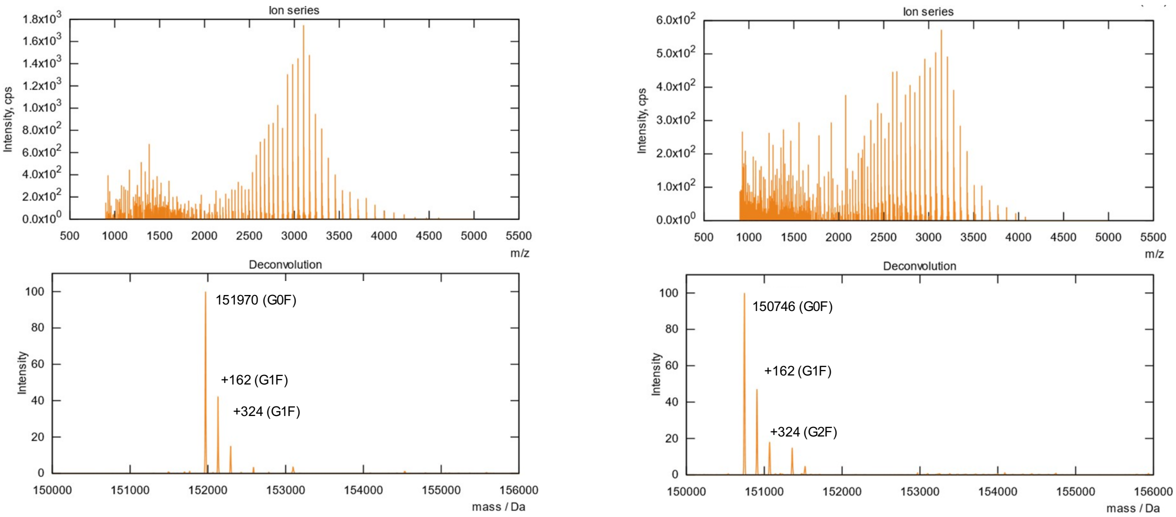

	Mass before reaction (Da)	Mass after reaction (Da)	Mass change (Da)
Expected	151967	150741	-1226
Observed	151970	150746	-1224

Figure S99. UV chromatograms, ion series and deconvoluted mass spectra obtained from LC-MS analysis of antiCD33-(ST)-(239iC)-3 before and after modification.

7.10.4 AntiCD33-(P4)-(239iC-DL488)

Conjugation of DL488-Mal to AntiCD33-(P4)-(239iC) was performed following method "Maleimide conjugation" described in S6.10 using 2 mL of protein solution. The resulting solution was desalted using an Amicon® Ultra 0.5 mL Centrifugal Filter (100K MWCO). The purified protein, termed antiCD33-(P4)-(239iC-DL488) was characterised by LC-MS, which confirmed successful conjugation of DL488-Mal.

antiCD33-(P4)-(239iC) —(DL488-Mal; 25 °C, 1 h)→ antiCD33-(P4)-(239iC-DL488)

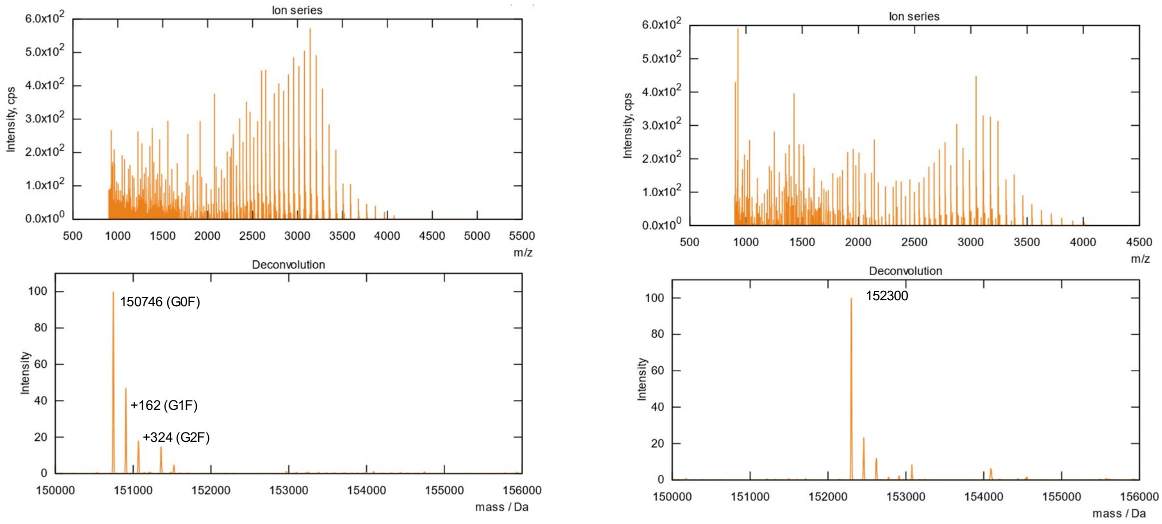

	Mass before reaction (Da)	Mass after reaction (Da)	Mass change (Da)
Expected	150742	152296	+1554
Observed	150746	152300	+1554

Figure S100. Ion series and deconvoluted mass spectra obtained from LC-MS analysis of antiCD33- (P4)-(239iC) before and after modification.

7.10.5 AntiCD33-(P4-AF647)-(239iC-DL488)

Conjugation of AZDye647 to antiCD33-(P4)-(239iC-DL488) was performed following method "SPAAC conjugation" described in S6.9 using 2 mL of protein solution. An aliquot of the resulting solution was desalted using an Amicon® Ultra 0.5 mL Centrifugal Filter (100K MWCO) and analysed by LC-MS. This confirmed conversion to the expected product. The remaining protein solution was then purified by SEC.

antiCD33-(P4)-(239iC-DL488) —(AF647-DBCO; 25 °C, 1 h)→ antiCD33-(P4-AF647)-(239iC-DL488)

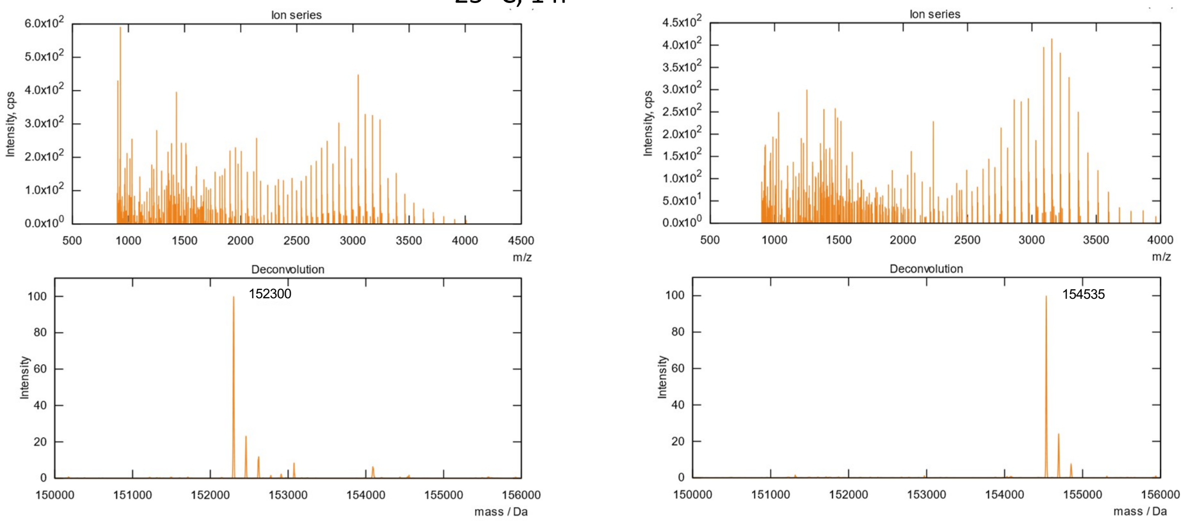

	Mass before reaction (Da)	Mass after reaction (Da)	Mass change (Da)
Expected	152296	154530	+2234
Observed	152300	154535	+2235

Figure S101. Ion series and deconvoluted mass spectra obtained from LC-MS analysis of antiCD33- (P4)-(239iC-DL488) before and after modification.

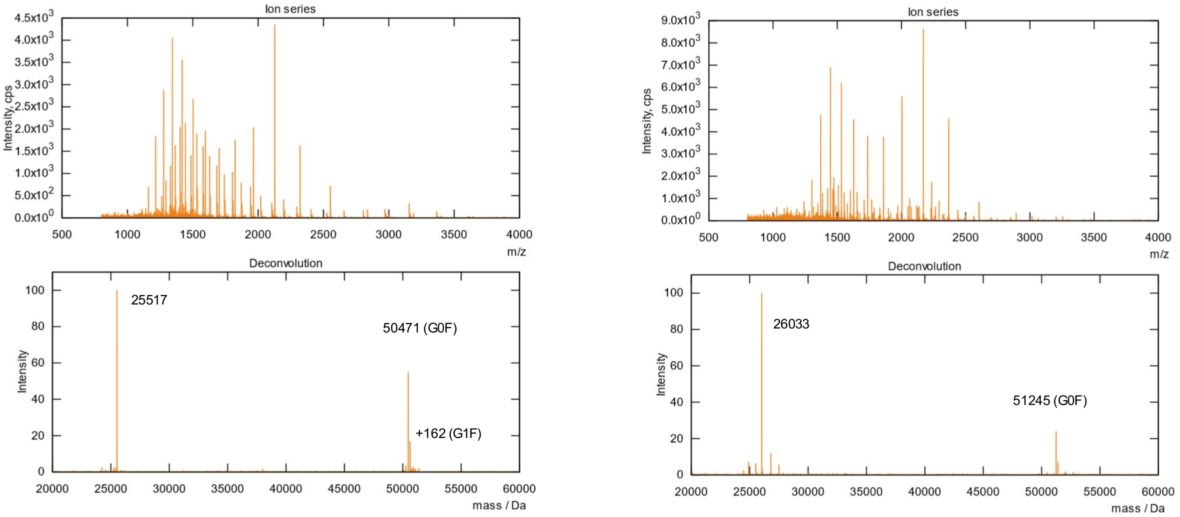

		Mass before reaction (Da)	Mass after reaction (Da)	Mass change (Da)
LC	Expected	25517	26035	518
	Observed	25517	26033	516
HC	Expected	50470	51247	777
	Observed	50471	51245	774

Figure S102. Ion series and deconvoluted mass spectra obtained from LC-MS analysis of antiCD33- (P4)-(239iC-DL488) before and after modification.

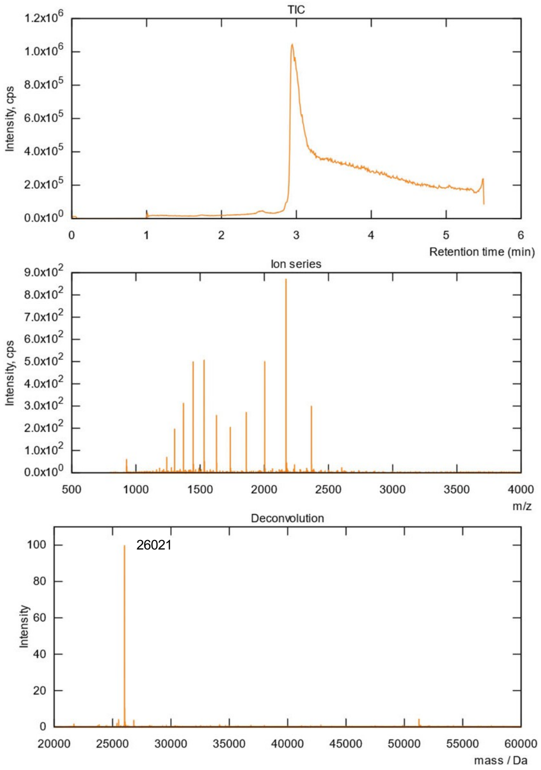

Figure S103. Ion series and deconvoluted mass spectra obtained from LC-MS analysis of antiCD33- (P4-AF647)-(239iC-DL488) after SEC purification.

Peak #	RetTime [min]	Sig	Type	Area [mAU*s]	Height [mAU]	Area %
1	7.472	1	BV	79.67460	1.81234	1.0830
2	8.719	1	VV R	7276.90625	137.61610	98.9170

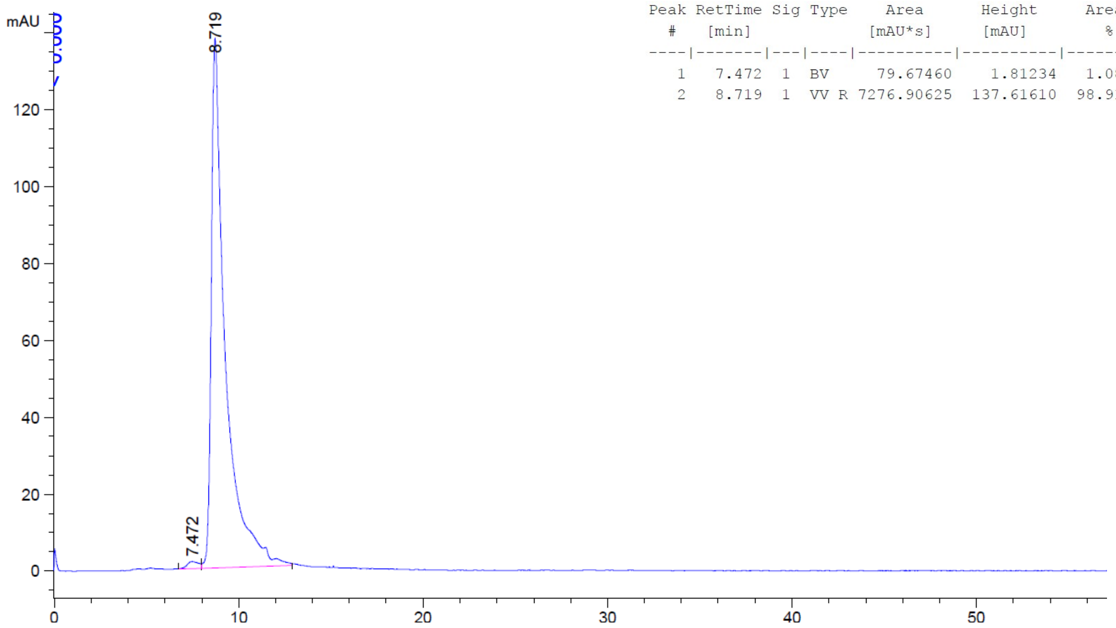

Figure S104. UV chromatogram of antiCD33-(P4-AF647)-(239iC-DL488) obtained from HP-SEC analysis.

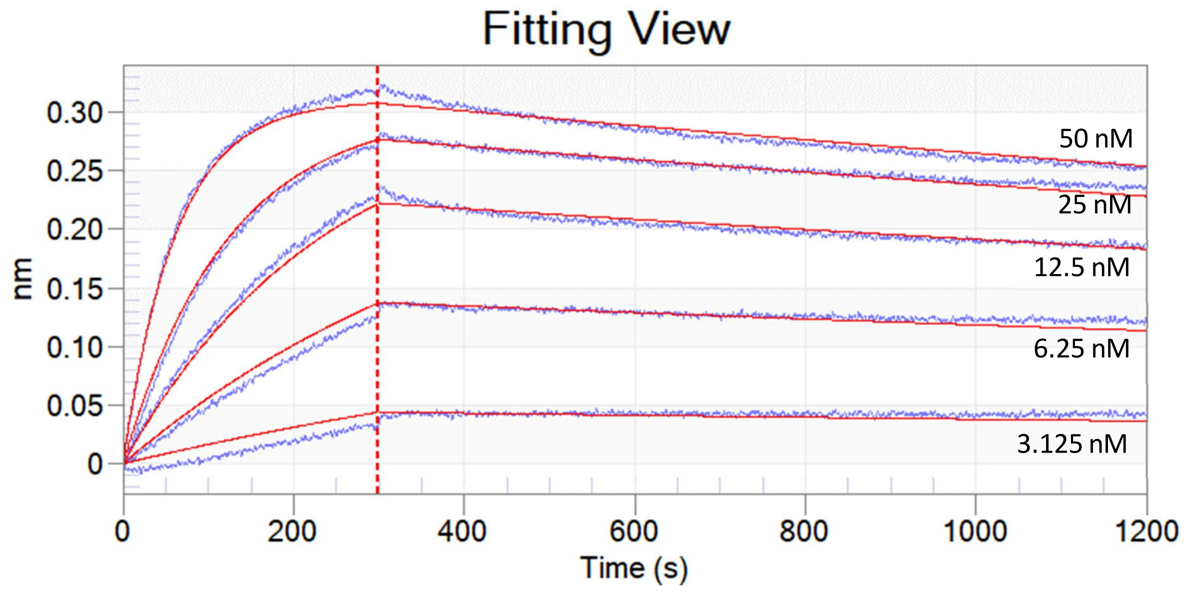

Sample Ref.	K_D (M)	K_D Fitting Error (M)	K_{on} (1/Ms)	K_{on} error (1/Ms)	K_{dis} (1/s)	K_{dis} error (1/s)
antiCD33-(P4-AF647)-(239iC-DL488)	6.69E-10	5.00E-12	3.19E+05	1.35E+03	2.13E-04	1.31E-06
antiCD33-(wildtype)	8.68E-10	4.97E-12	2.63E+05	9.12E+02	2.28E-04	1.04E-06

Figure S105. Binding curves and corresponding kinetic parameters obtained from BLI analysis of antiCD33-(P4-AF647)-(239iC-DL488).

7.11 Triple modification: CPO, maleimide (239iC) and sortase

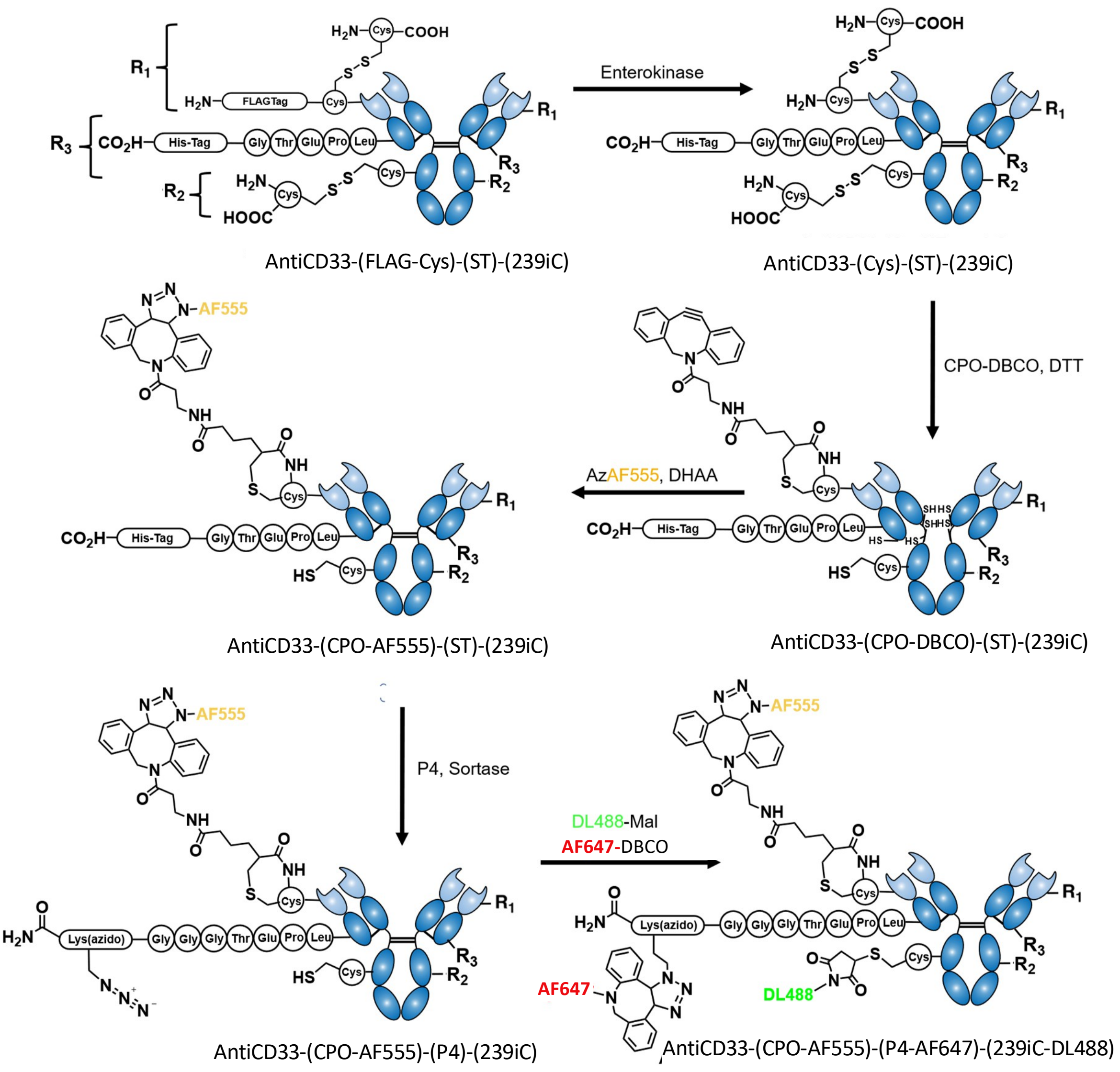

Figure S106. Schematic of the CPO, maleimide (239iC) and sortase based modification protocol.

7.11.1 AntiCD33-(FLAG-Cys)-(ST)-(239iC)

AntiCD33-(FLAG-Cys)-(ST)-(239iC) was expressed and purified, following the protocol described in 6.2.4, achieving a post-purification yield of 38 mg L^{-1} (Normalised to wildtype = 42 mg L^{-1}). LC-MS and biophysical analysis were conducted to assess the identity and integrity of the product.

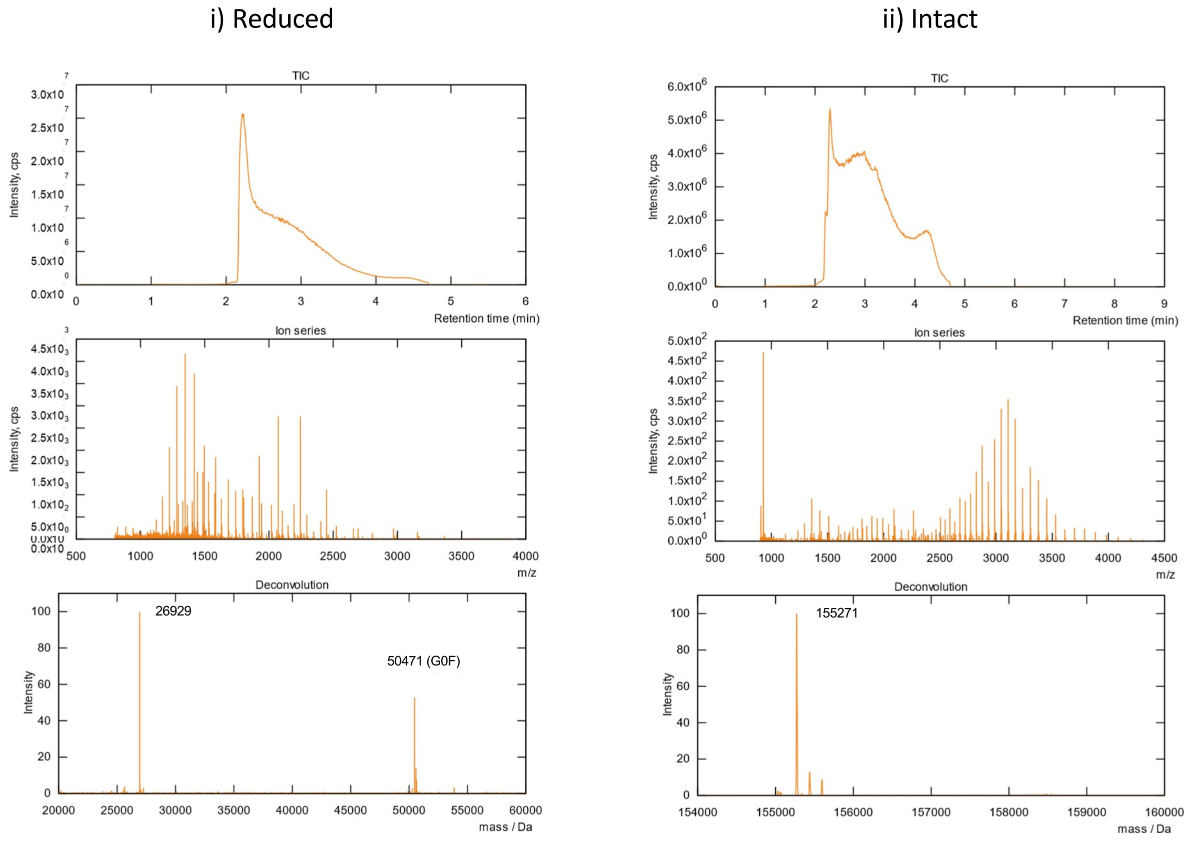

Ref.	Expected mass (Da)	Observed mass (Da)
i	LC: 26930<br>HC: 50470	LC: 26929<br>HC: 50471
ii	155269	155271

Figure S107. UV chromatograms, ion series and deconvoluted mass spectra obtained from LC-MS analysis of antiCD33-(FLAG-Cys)-(ST)-(239iC) under (i) reduced and (ii) intact conditions.

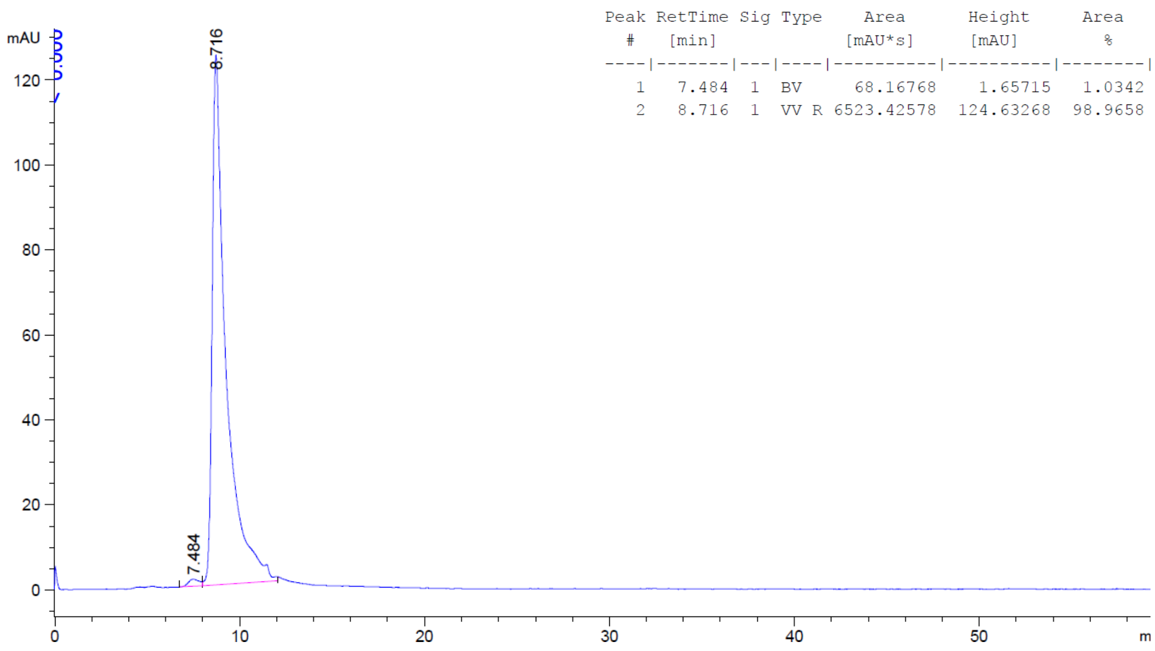

Figure S108. UV chromatogram obtained from HP-SEC analysis of antiCD33-(FLAG-Cys)-(ST)- (239iC).

7.11.2 AntiCD33-(Cys)-(ST)-(239iC)

The FLAG tag of AntiCD33-(FLAG-Cys)-(ST)-(239iC) was removed following method "FLAG cleavage", described in S6.2. The subsequent reaction mixture was desalted using an Amicon® Ultra 0.5 mL Centrifugal Filter (10K MWCO). The purified protein, termed antiCD33-(Cys)-(ST)-(239iC), was then characterised by LC-MS, which confirmed successful cleavage of the FLAG tag.

antiCD33-(FLAG-Cys)-(ST)-(239iC) —(Enterokinase; 25 °C, 16 h)→ antiCD33-(Cys)-(ST)-(239iC)

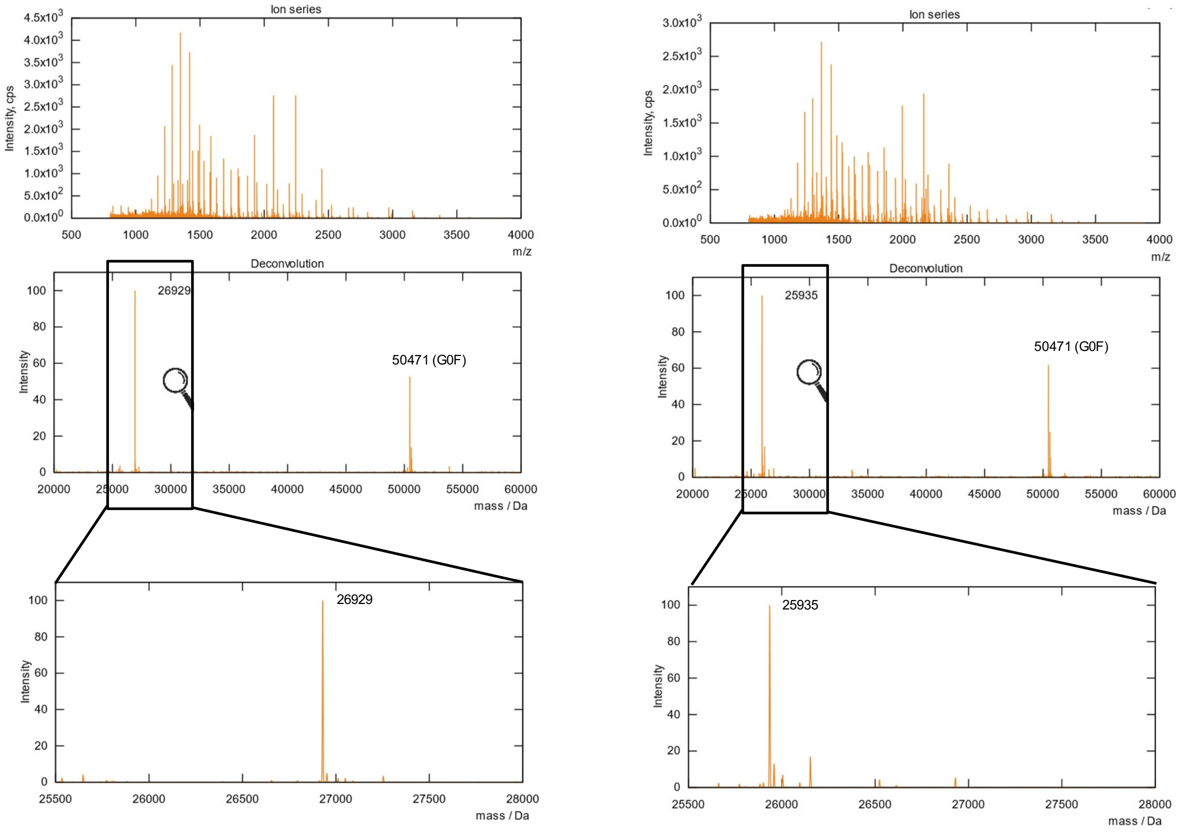

		Mass before cleavage (Da)	Mass after cleavage (Da)	Mass change (Da)
LC	Expected	26930	25935	-995
	Observed	26929	25935	-994
HC	Expected	50470	50470	0
	Observed	50471	50471	0

Figure S109. UV chromatograms, ion series and deconvoluted mass spectra obtained from LC-MS analysis of antiCD33-(FLAG-Cys)-(ST)-(239iC) before and after cleavage.

7.11.3 AntiCD33-(CPO-DBCO)-(ST)-(239iC)

Conjugation of CPO-DBCO to antiCD33-(Cys)-(ST)-(239iC) was performed following method "CPO conjugation" described in S6.5 using 500 µL of protein solution. The subsequent reaction mixture was purified using an Amicon® Ultra 0.5 mL Centrifugal Filter (10K MWCO). The purified protein, termed antiCD33-(DBCO-CPO)-(ST)-(239iC), was characterised by LC-MS, which confirmed successful CPO-DBCO conjugation.

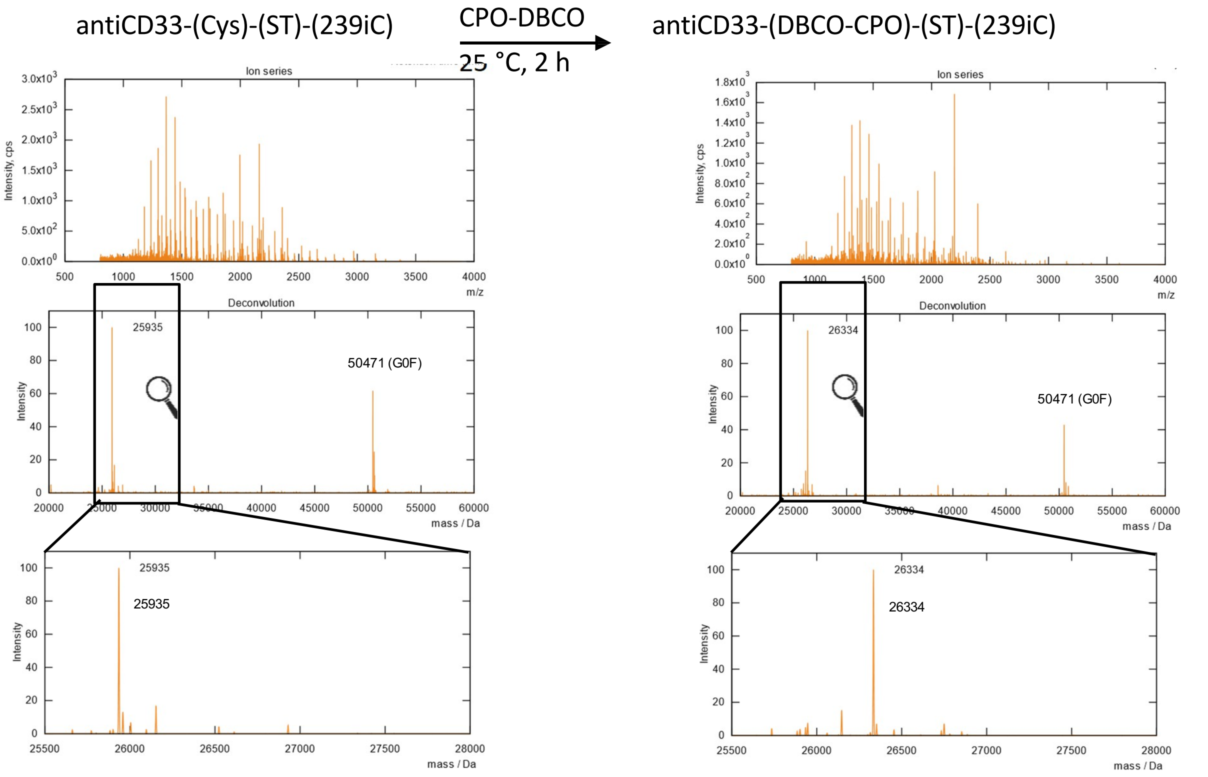

		Mass before reaction (Da)	Mass after reaction (Da)	Mass change (Da)
LC	Expected	25935	26333	+398
	Observed	25935	26334	+399
HC	Expected	50470	50470	0
	Observed	50471	50471	0

Figure S110. UV chromatograms, ion series and deconvoluted mass spectra obtained from LC-MS analysis of antiCD33-(Cys)-(ST)-(239iC) before and after modification.

7.11.4 AntiCD33-(CPO-AF555)-(ST)-(239iC)

Conjugation of AzAF555 to antiCD33-(CPO-DBCO)-(ST)-(239iC) was performed following method "SPAAC conjugation" described in S6.9 using 500 µL of protein solution. The resulting solution was desalted using an Amicon® Ultra 0.5 mL Centrifugal Filter (100K MWCO). The purified protein, termed antiCD33-(CPO-AF555)-(ST)-(239iC) was characterised by LC-MS, which confirmed successful conjugation of AzAF555.

antiCD33-(CPO-DBCO)-(ST)-(239iC) —[AzAF555; 25 °C, 1 h]→ antiCD33-(CPO-AF555)-(ST)-(239iC)

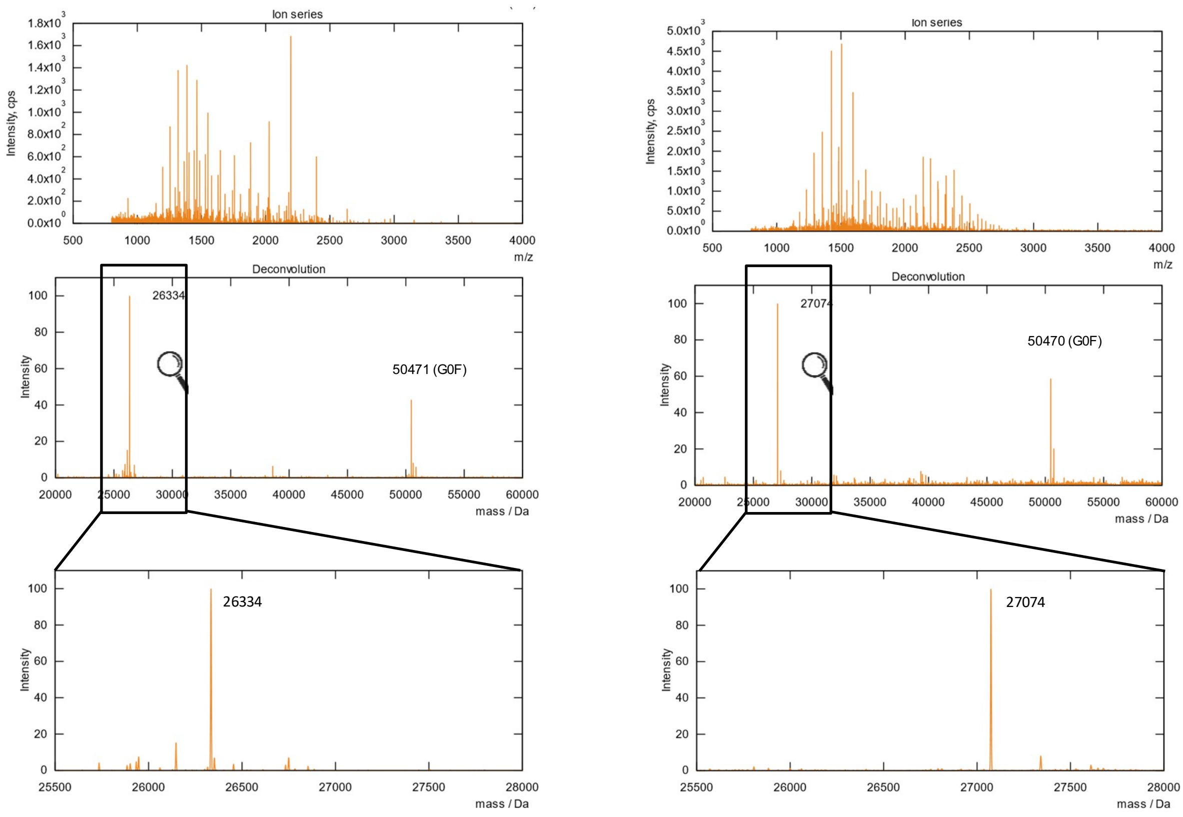

		Mass before reaction (Da)	Mass after reaction (Da)	Mass change (Da)
LC	Expected	26333	27073	+740
	Observed	26334	27074	+740
HC	Expected	50470	50470	0
	Observed	50471	50470	-1

Figure S111. UV chromatograms, ion series and deconvoluted mass spectra obtained from LC-MS analysis of antiCD33-(CPO-DBCO)-(ST)-(239iC) before and after modification.

7.11.5 AntiCD33-(CPO-AF555)-(P4)-(239iC)

Conjugation of P4 to antiCD33-(CPO-AF555)-(ST)-(239iC) was performed following method "Sortase-mediated conjugation" described in S6.6 using 50 µL of protein solution.. The resulting solution was then purified using Amicon® Ultra 0.5 mL Centrifugal Filters (10K MWCO). The purified product, termed antiCD33-(CPO-AF555)-(P4)-(239iC), was subjected to LC-MS analysis, which confirmed successful modification.

antiCD33-(CPO-AF555)-(ST)-(239iC) —(P4, Sortase; 37 °C, 1 h)→ antiCD33-(CPO-AF555)-(P4)-(239iC)

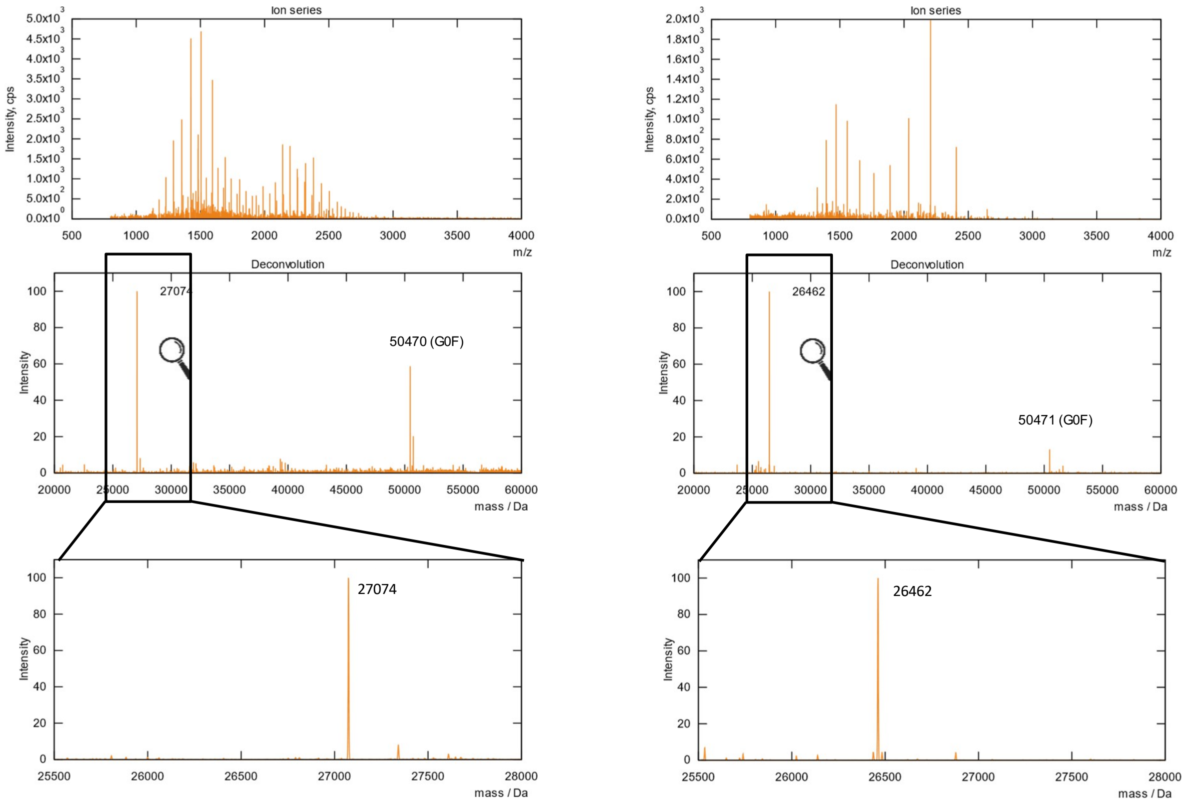

		Mass before reaction (Da)	Mass after reaction (Da)	Mass change (Da)
LC	Expected	27073	26459	-614
	Observed	27074	26462	-612
HC	Expected	50470	50470	0
	Observed	50470	50471	+1

Figure S112. Ion series and deconvoluted mass spectra obtained from LC-MS analysis of antiCD33- (CPO-AF555)-(ST)-(239iC) before and after modification.

7.11.6 AntiCD33-(CPO-AF555)-(P4-AF647)-(239iC-DL488)

Conjugation of AF647 to antiCD33-(CPO-AF555)-(P4)-(239iC) was performed following method "SPAAC conjugation" described in S6.9 using 300 µL of protein solution. Conjugation of DL-488 maleimide was then performed on the resulting solution following method "Maleimide conjugation" described in 6.4.2.10 using 300 µL of protein solution.. The resulting solution was desalted using an Amicon® Ultra 0.5 mL Centrifugal Filter (100K MWCO). The purified protein, termed antiCD33-(CPO- AF555)-(P4-AF647)-(239iC-DL488) was characterised by LC-MS, which confirmed successful conjugation.

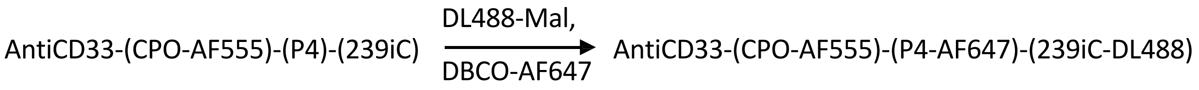

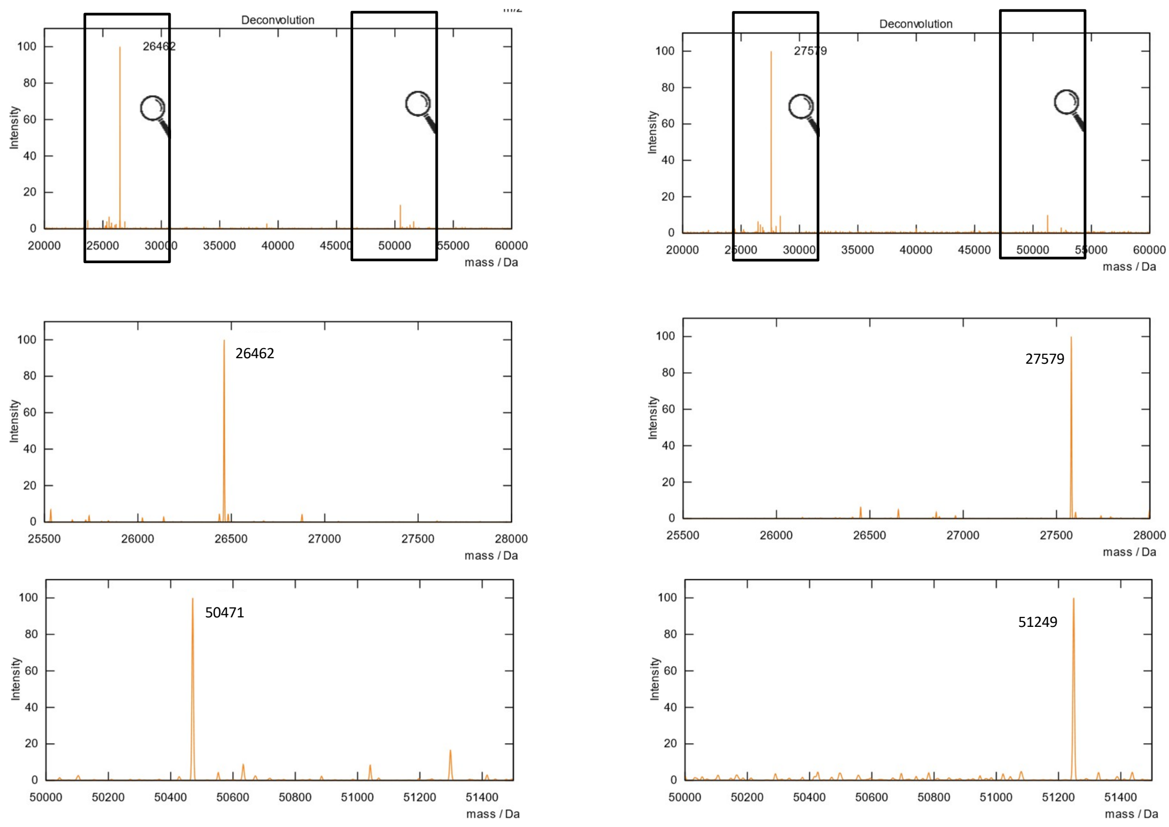

		Mass before reaction (Da)	Mass after reaction (Da)	Mass change (Da)
LC	Expected	26459	27576	+1117
	Observed	26462	27579	+1117
HC	Expected	50470	51247	+777
	Observed	50471	51249	+778

Figure S113. Ion series and deconvoluted mass spectra obtained from LC-MS analysis of antiCD33- (CPO-AF555)-(P4)-(239iC) before and after modification.

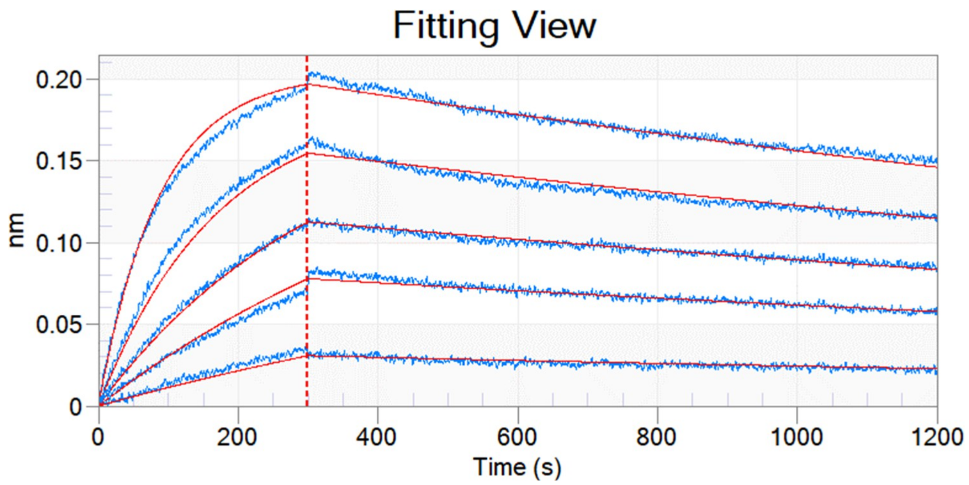

Ref.	K_D (M)	K_D Fitting Error (M)	K_{on} (1/Ms)	K_{on} error (1/Ms)	K_{dis} (1/s)	K_{dis} error (1/s)
AntiCD33-(wildtype)	8.68E-10	4.97E-12	2.63E+05	9.12E+02	2.28E-04	1.04E-06
AntiCD33-(CPO-AF555)-(P4-AF647)-(239iC-DL488)	1.49E-09	9.65E-12	2.23E+05	1.06E+03	3.32E-04	1.45E-06

Figure S114. Binding curves and corresponding kinetic parameters obtained from BLI analysis of AntiCD33-(CPO-AF555)-(P4-AF647)-(239iC-DL488).

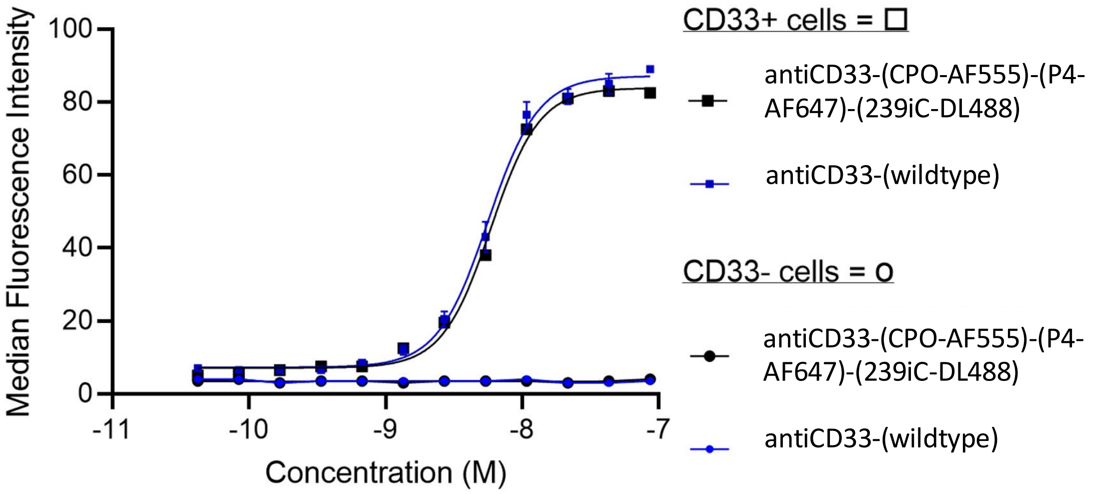

	EC_{50}	
	CD33-	CD33+
antiCD33-(wildtype)	N/A	5.612 ± 0.361
antiCD33-(CPO-AF555)-(P4-AF647)-(239iC-DL488)	N/A	5.912 ± 0.365

Figure S115. Titrated FACS binding curve from antiCD33-(CPO-AF555)-(P4-AF647)-(239iC-DL488).

7.12 Quadruple modification: CPO, maleimide(239iC), sortase and GALaXy

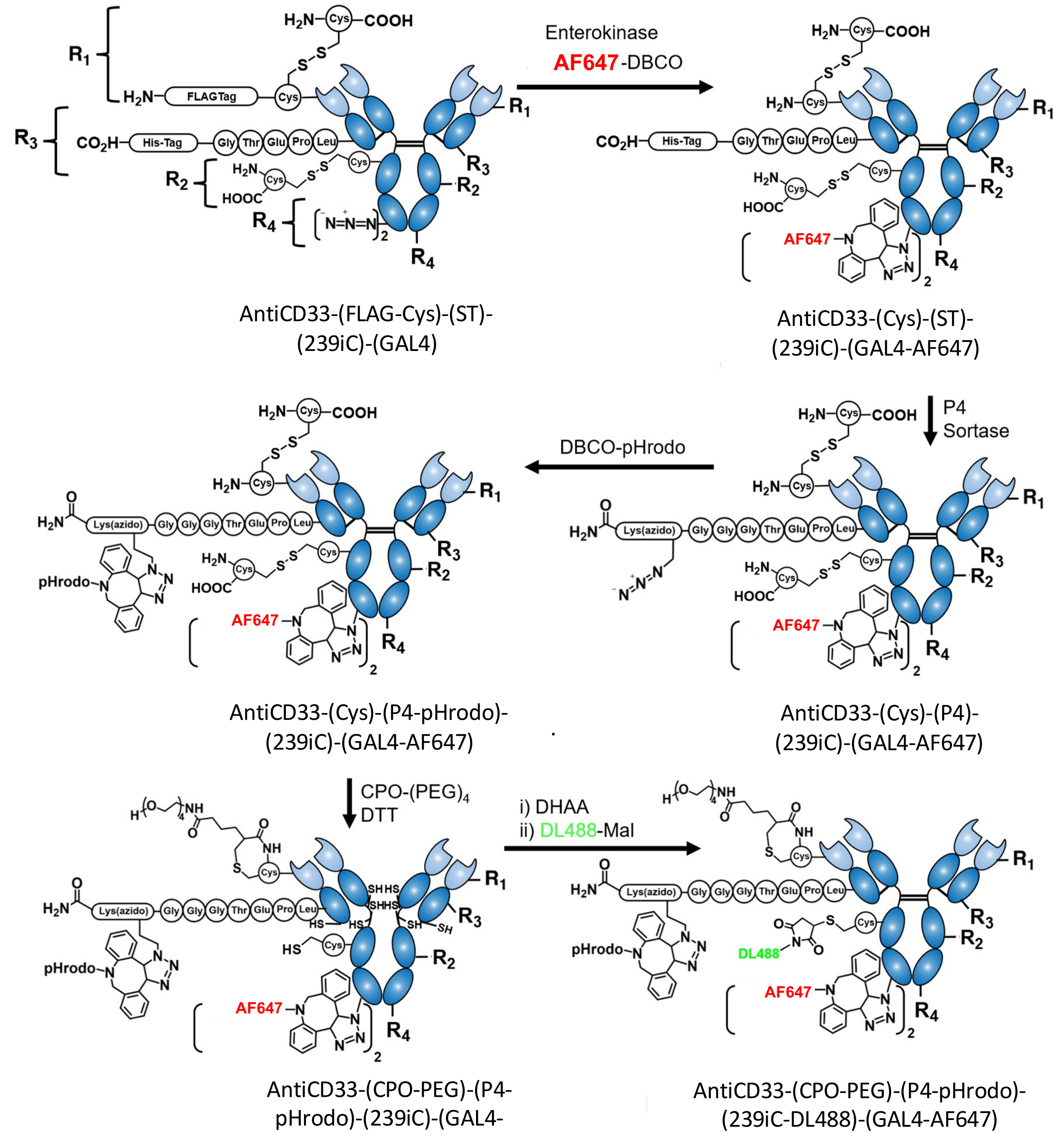

Figure S116. Schematic of the CPO, maleimide(239iC), sortase and GALaXy based protocol.

7.12.1 AntiCD33-(FLAG-Cys)-(ST)-(239iC)-(GAL4)

AntiCD33-(FLAG-Cys)-(ST)-(239iC)-(GAL4) was expressed and purified, following the protocol described in 6.2.4, achieving a post-purification yield of 12 mg L^{-1}. LC-MS and biophysical analysis were conducted to assess the identity of the product.

A) Reducing B) Intact

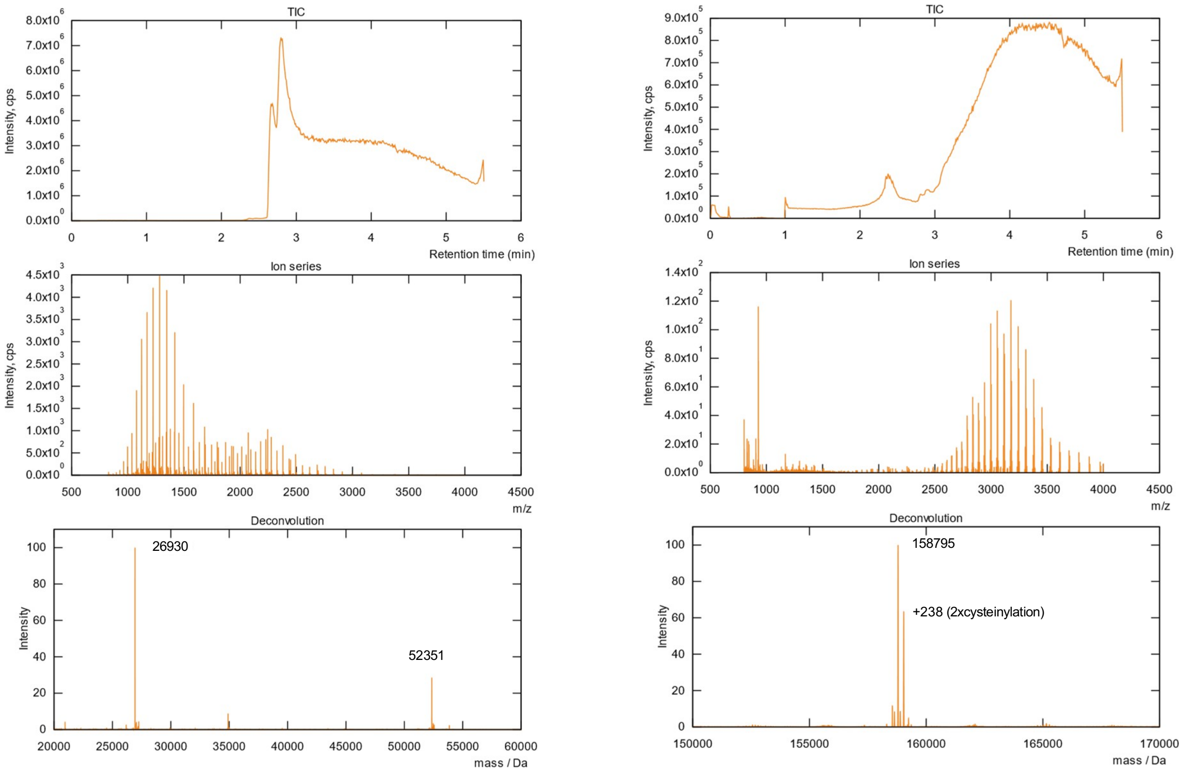

Ref.	Expected mass (Da)	Observed mass (Da)
i	LC: 26930<br>HC: 52351	LC: 26930<br>HC: 52351
ii	158792	158795

Figure S117. UV chromatograms, ion series and deconvoluted mass spectra obtained from LC-MS analysis of antiCD33-(FLAG-Cys)-(ST)-(239iC)-(GAL4) under (i) reduced and (ii) intact conditions.

7.12.2 AntiCD33-(Cys)-(ST)-(239iC)-(GAL4-AF647)

The FLAG tag of AntiCD33-(FLAG-Cys)-(ST)-(239iC)-(GAL4) was removed following method "FLAG cleavage", described in S6.2. The described method was adapted to include DBCO-AF647 (10 equiv.). The subsequent reaction mixture was desalted using an Amicon® Ultra 0.5 mL Centrifugal Filter (10K MWCO). The purified protein, termed antiCD33-(Cys)-(ST)-(239iC)-(GAL4-AF647), was then characterised by LC-MS, which confirmed successful cleavage of the FLAG tag and conjugation of DBCO-AF647.

AntiCD33-(FLAG-Cys)-(ST)-(239iC)-(GAL4) ⟶ AntiCD33-(Cys)-(ST)-(239iC)-(GAL4-AF647)

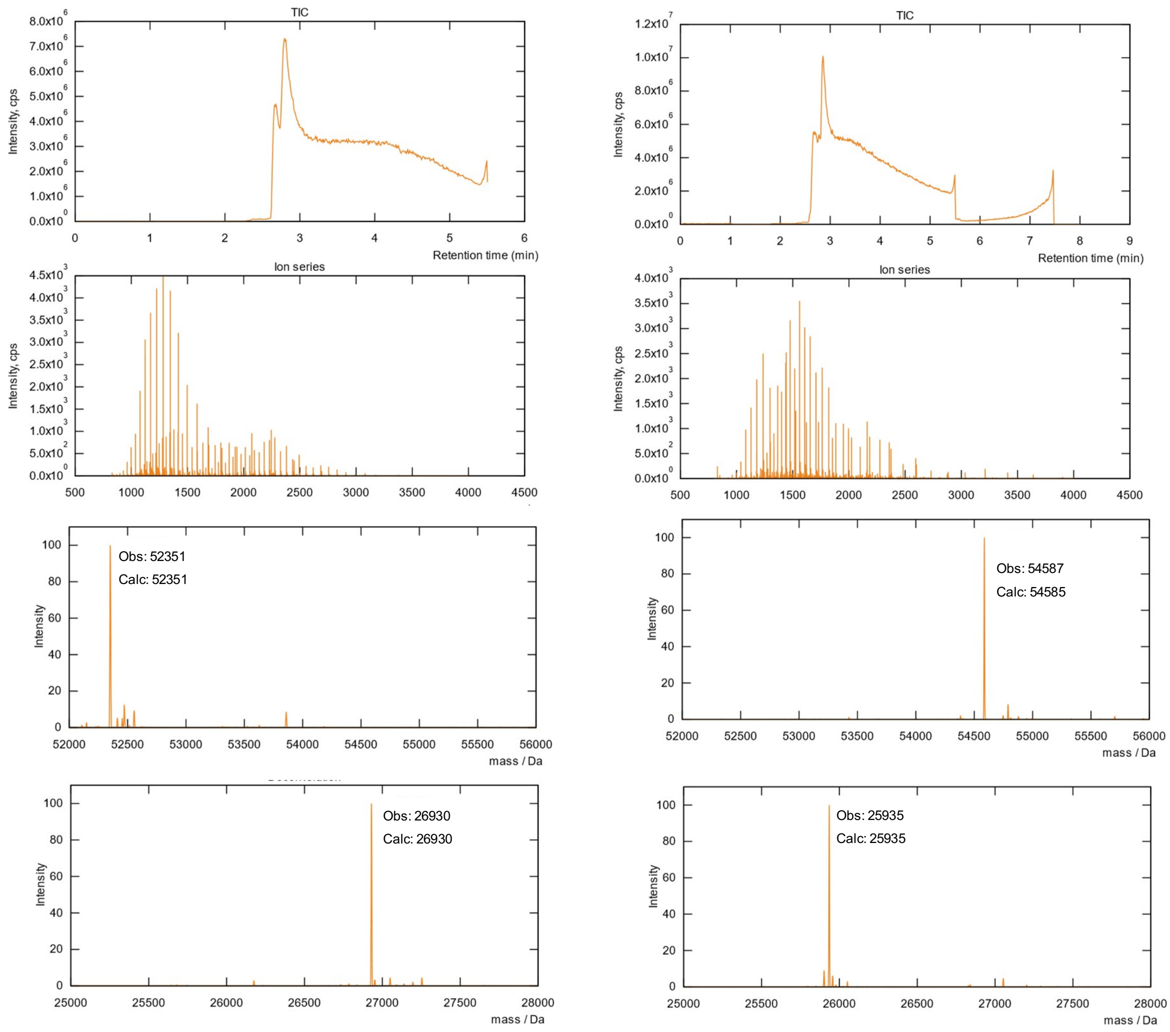

Figure S118. Ion series and deconvoluted mass spectra obtained from LC-MS analysis of AntiCD33- (FLAG-Cys)-(ST)-(239iC)-(GAL4) before and after modification.

7.12.3 AntiCD33-(Cys)-(P4)-(239iC)-(GAL4-AF647)

Conjugation of P4 to antiCD33-(Cys)-(ST)-(239iC)-(GAL4-AF647) was performed following method "Sortase-mediated conjugation" described in S6.6 using using 200 µL of protein solution. The resulting solution was then purified using SEC. The purified product, antiCD33-(Cys)-(P4)-(239iC)-(GAL4-AF647), was subjected to LC-MS analysis, which confirmed successful conjugation of P4.

AntiCD33-(Cys)-(ST)-(239iC)-(GAL4-AF647) —(P4, Sortase; 37 °C, 1 h)→ AntiCD33-(Cys)-(P4)-(239iC)-(GAL4-AF647)

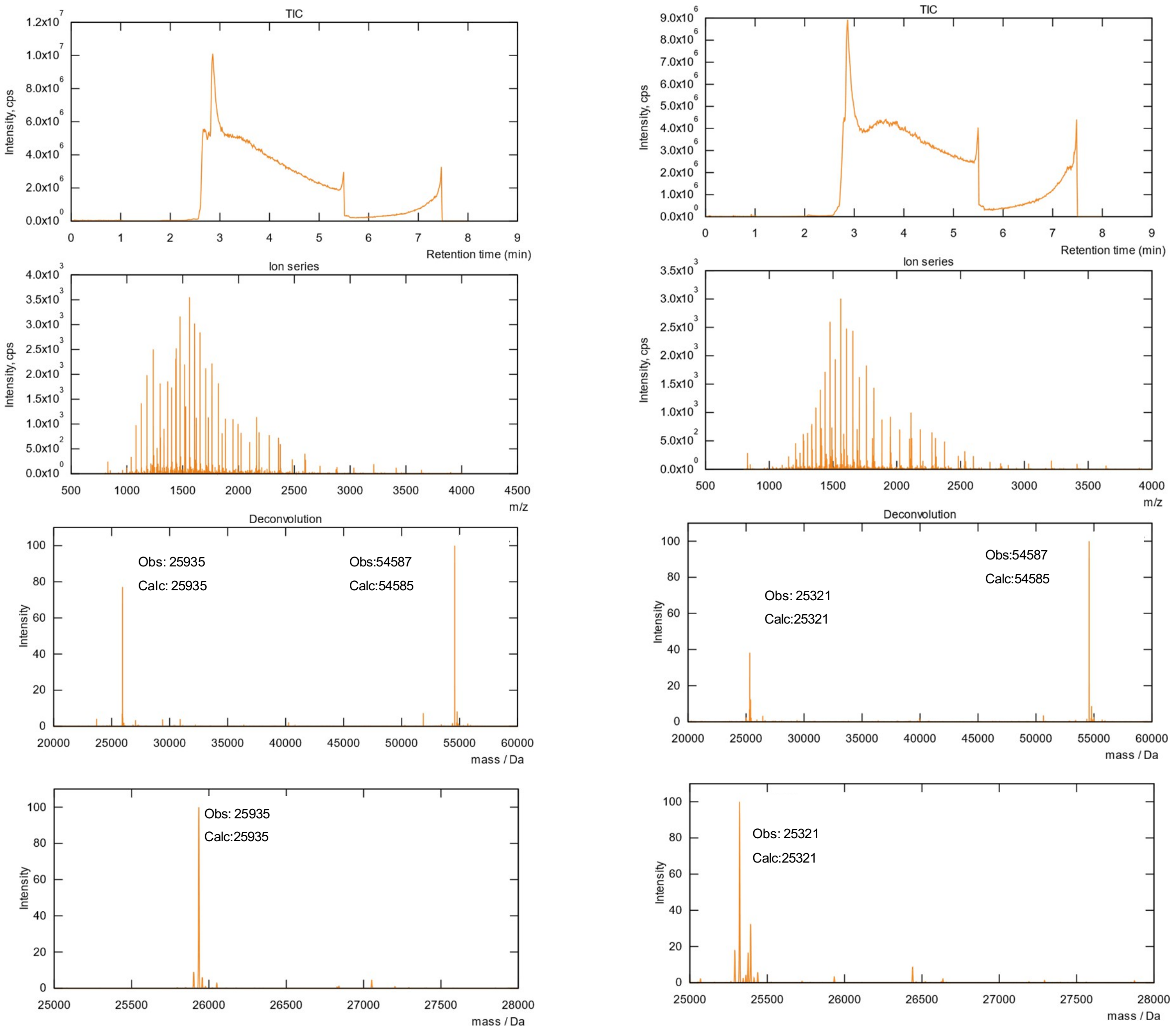

Figure S119. UV chromatogram, ion series and deconvoluted mass spectra obtained from LC-MS analysis of AntiCD33-(Cys)-(ST)-(239iC)-(GAL4-AF647) before and after modification.

7.12.4 AntiCD33-(Cys)-(P4-pHrodo)-(239iC)-(GAL4-AF647)

Conjugation of DBCO-pHrodo to antiCD33-(Cys)-(P4)-(239iC)-(GAL4-AF647) was performed following method “SPAAC conjugation” described in S6.9 using 2 mL of protein solution. The resulting solution was desalted using an Amicon® Ultra 0.5 mL Centrifugal Filter (100K MWCO). The purified protein, termed antiCD33-(Cys)-(P4-pHrodo)-(239iC)-(GAL4-AF647) was characterised by LC-MS, which confirmed successful conjugation.

AntiCD33-(Cys)-(P4)-(239iC)-(GAL4-AF647)   AntiCD33-(Cys)-(P4-pHrodo)-(239iC)-(GAL4-AF647)

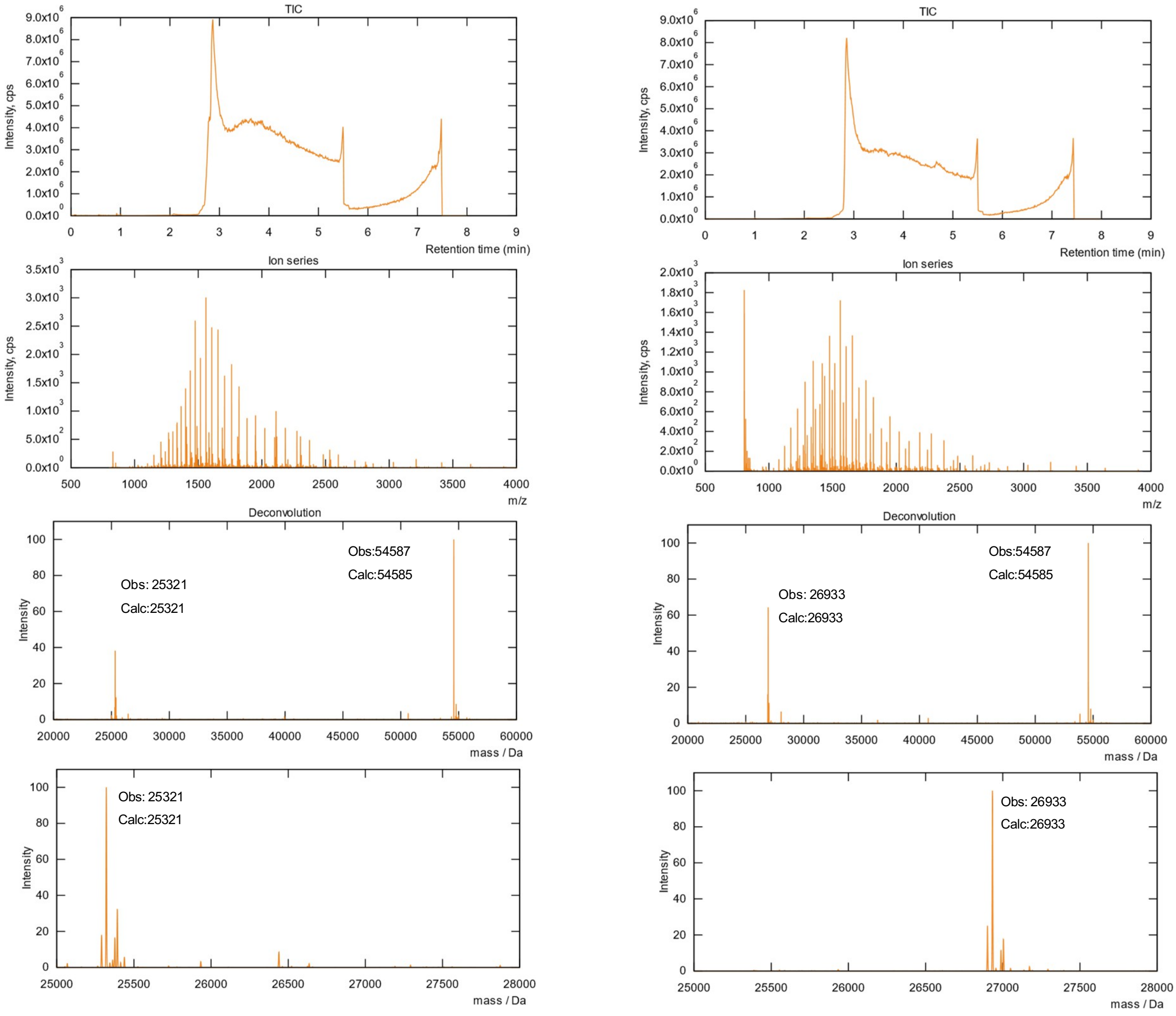

Figure S120. Ion series and deconvoluted mass spectra obtained from LC-MS analysis of AntiCD33- (Cys)-(P4)-(239iC)-(GAL4-AF647) before and after modification.

7.12.5 AntiCD33-(CPO-PEG)-(P4-pHrodo)-(239iC-DL488)-(GAL4-AF647)

Conjugation of CPO-PEG to antiCD33-(Cys)-(P4-pHrodo)-(239iC)-(GAL4-AF647) was performed following method “CPO conjugation” described in S6.5 using 2 mL of protein solution. Conjugation of Mal-DL488 to the resulting construct was then performed following method “Maleimide conjugation” described in S6.10. The subsequent protein, termed antiCD33-(CPO-PEG)-(P4-pHrodo)-(239iC-DL488)-(GAL4-AF647), was desalted and characterised by LC-MS, which confirmed successful CPO-PEG and Mal-DL488 conjugation.

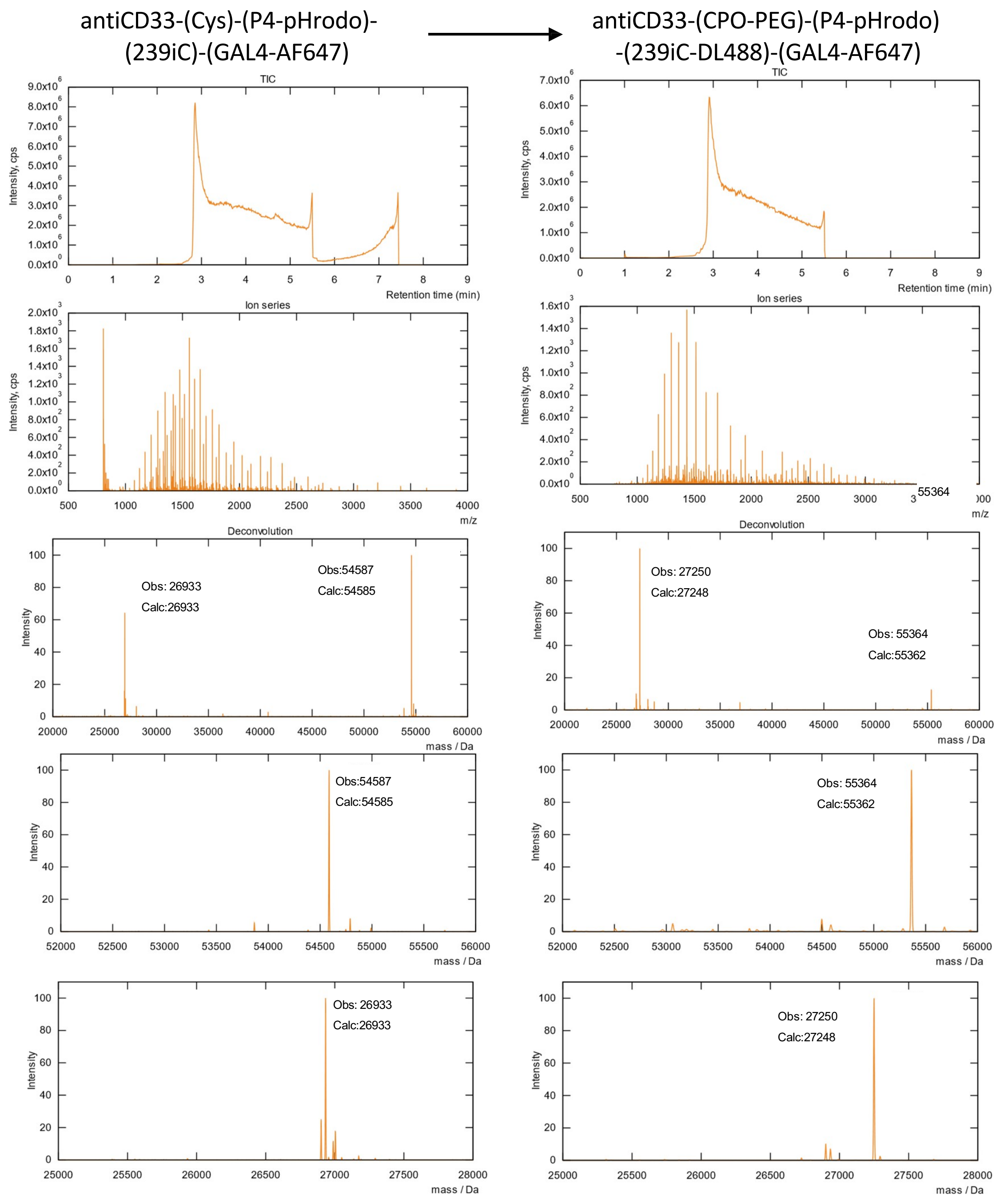

Figure S121. UV chromatogram, ion series and deconvoluted mass spectra obtained from LC-MS analysis of antiCD33-(Cys)-(P4-pHrodo)-(239iC)-(GAL4-AF647) before and after modification.

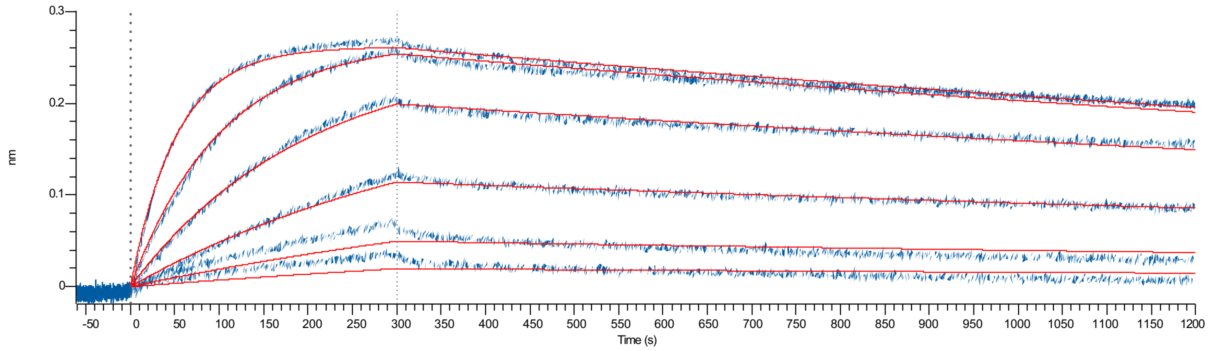

	K_D (M)	K_D Fitting Error(M)	K_{on} (1/Ms)	K_{on} error (1/Ms)	K_{dis} (1/s)	K_{dis} error (1/s)
antiCD33-(CPO-PEG)-(P4-pHrodo)-(239iC-DL488)-(GAL4-AF647)	8.449 E-10	2.872 E-12	3.781 E05	8.985 E02	3.195 E-04	7.763 E-07
antiCD33-(Wildtype)	8.68E-10	4.97E-12	2.63E+05	9.12E+02	2.28E-04	1.04E-06

Figure S122. Binding curves and corresponding kinetic parameters obtained from BLI analysis of antiCD33-(CPO-PEG)-(P4-pHrodo)-(239iC-DL488)-(GAL4-AF647).

S8 NMR

^1H NMR (400 MHz, $CDCl_3$) of perfluorophenyl hex-5-ynoate **2**

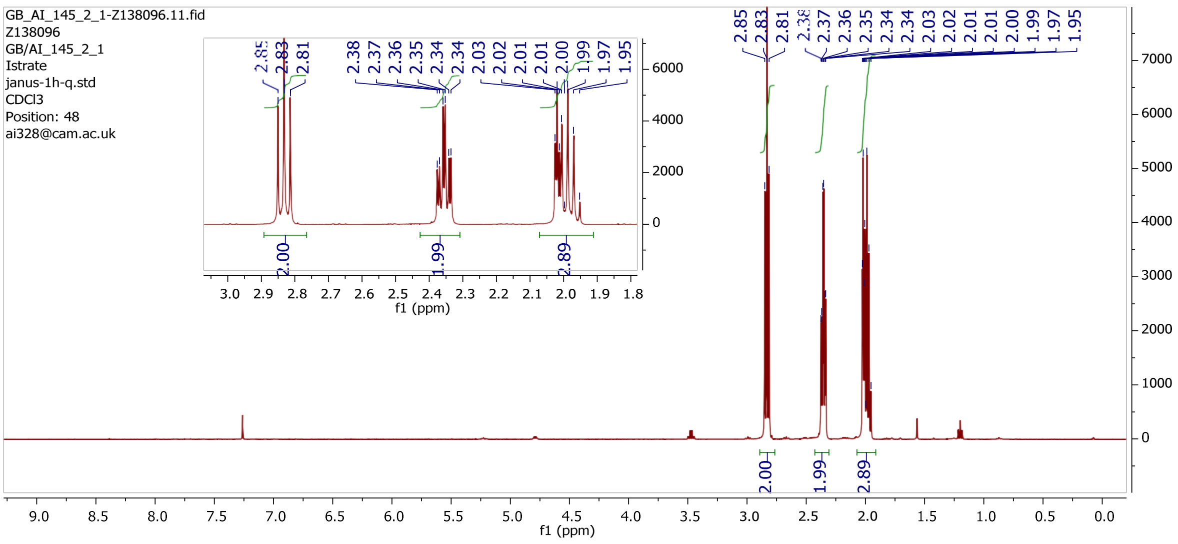

^{13}C NMR (100 MHz, $CDCl_3$) of perfluorophenyl hex-5-ynoate **2**

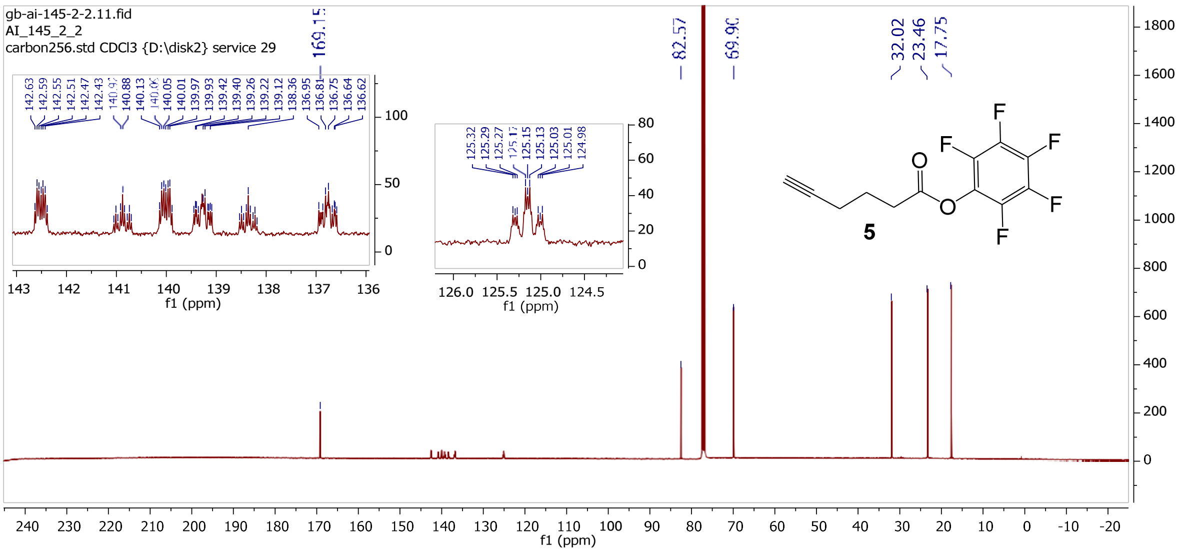

^{19}F NMR (376.5 MHz, $CDCl_3$) of perfluorophenyl hex-5-ynoate **2**

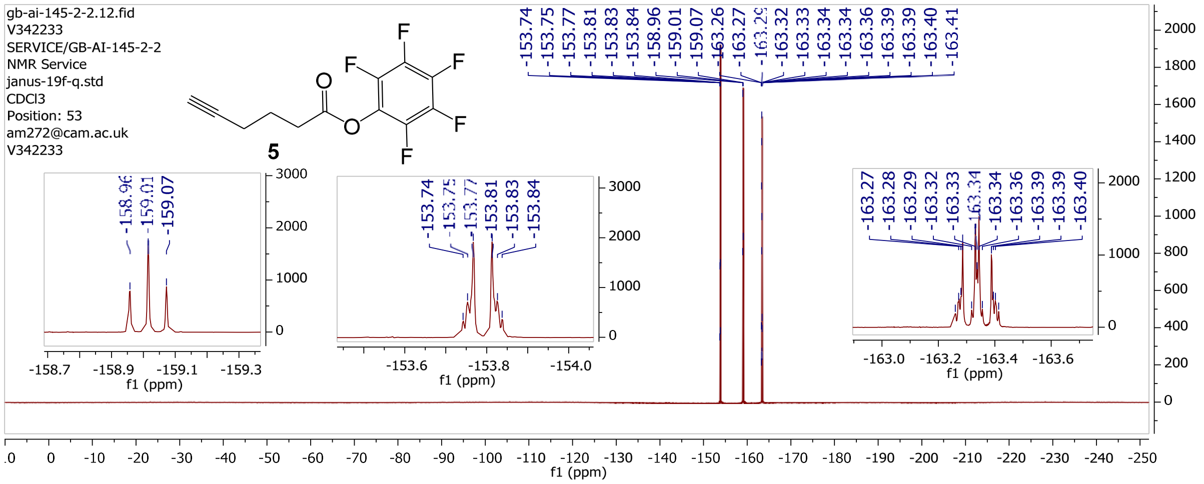

^{1}H NMR (500 MHz, $CDCl_3$) of perfluorophenyl 4-(3'-oxocycloprop-1'-en-1'-yl)butanoate **3**

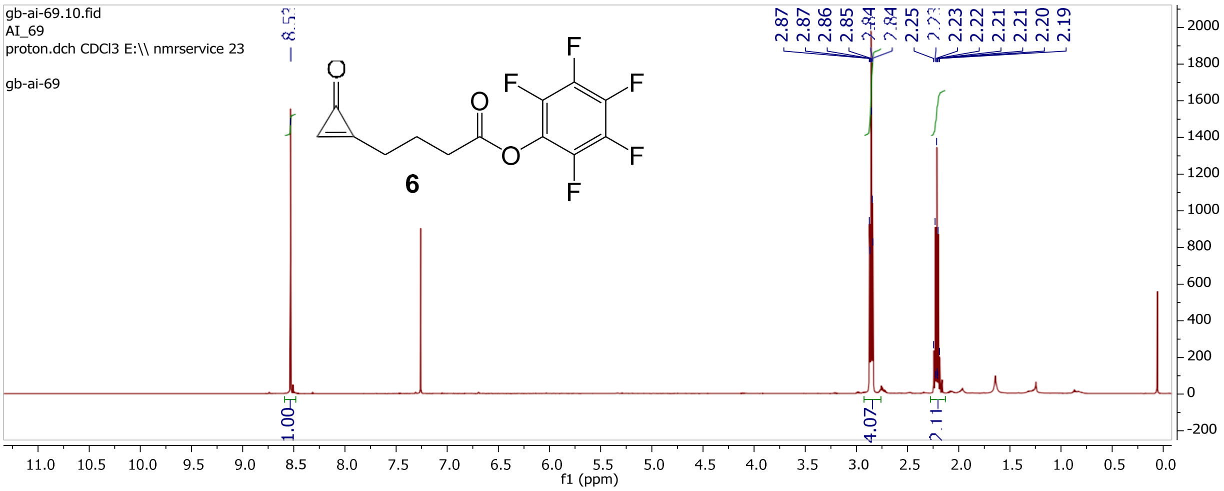

^{13}C NMR (125 MHz, $CDCl_3$) of Perfluorophenyl 4-(3’-oxocycloprop-1’-en-1’-yl)butanoate **3**

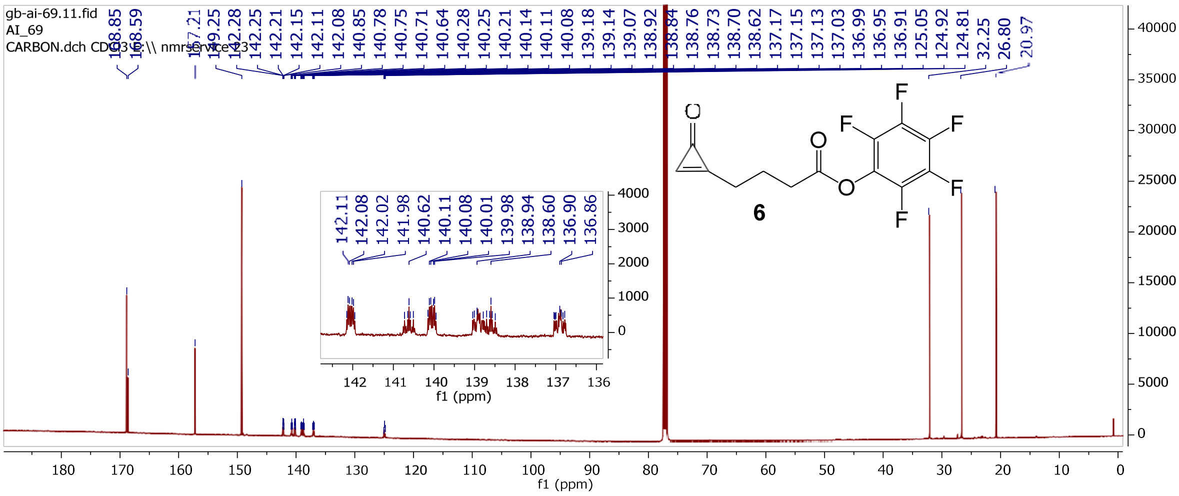

^{19}F NMR (376.5 MHz, $CDCl_3$) of Perfluorophenyl 4-(3’-oxocycloprop-1’-en-1’-yl)butanoate **3**

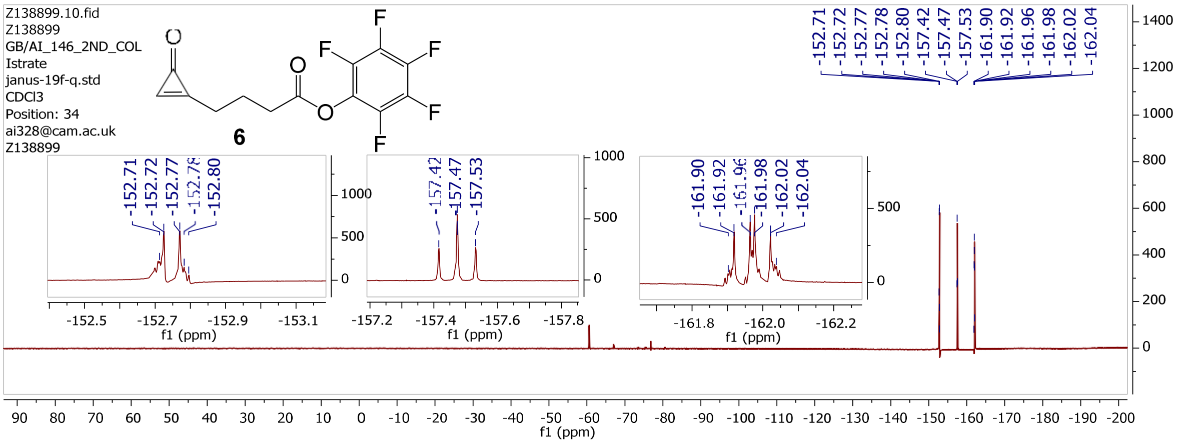

^{1}H NMR (400 MHz, $CDCl_3$) of **CPO-PEG**

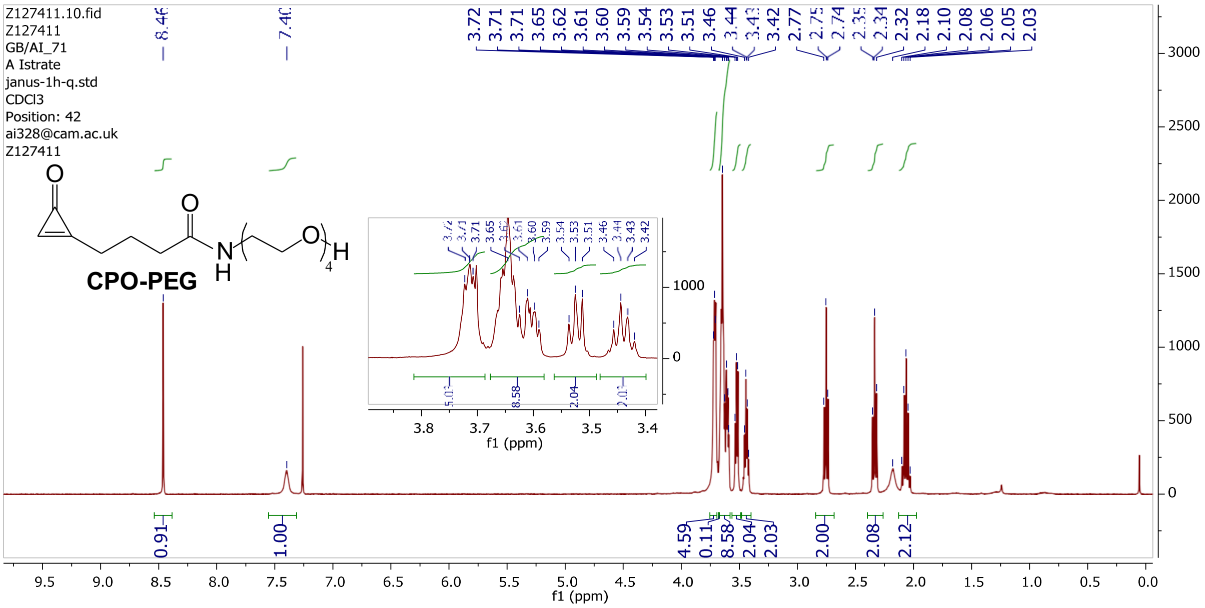

^{13}C NMR (125 MHz, $CDCl_3$) of **CPO-PEG**

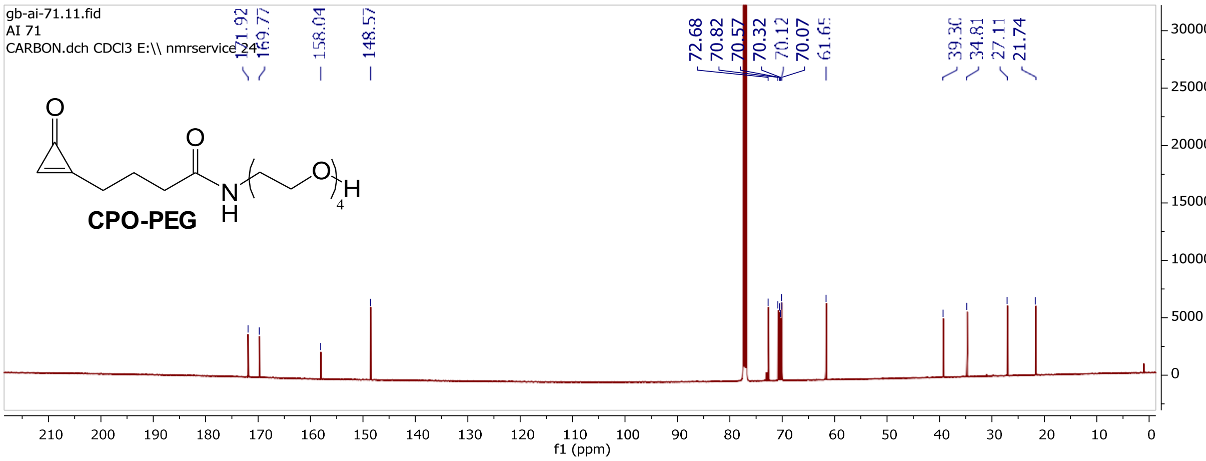

^{13}H NMR (400 MHz, $CDCl_3$) of **CPO-Alkyne**

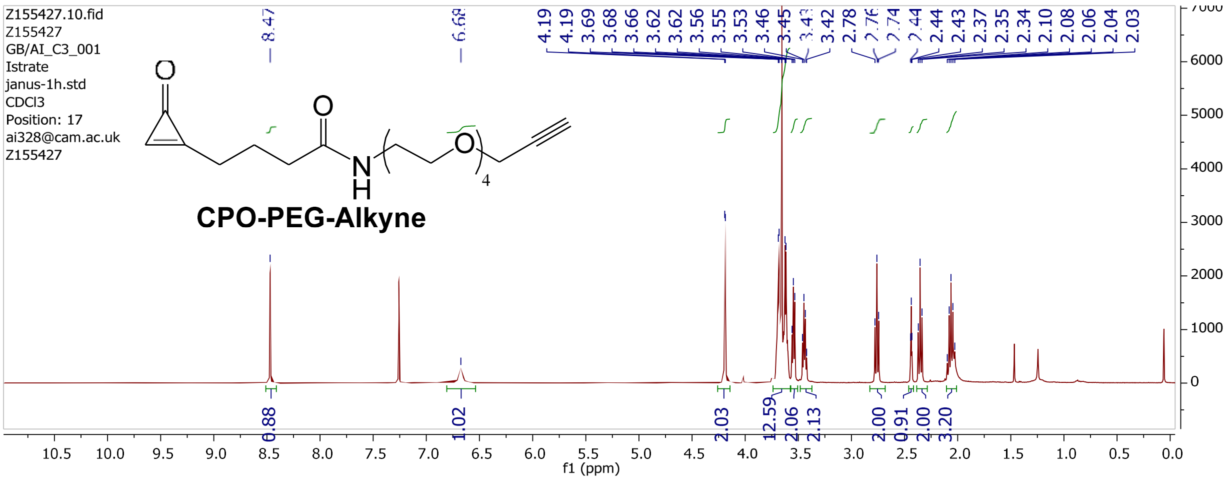

^{13}C NMR (100 MHz, $CDCl_3$) of **CPO-Alkyne**

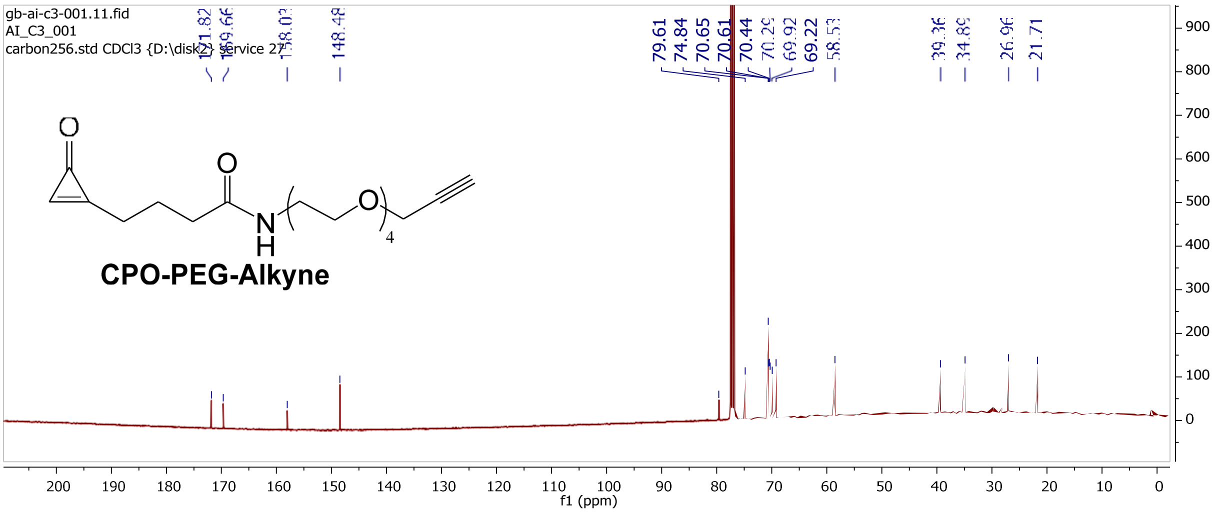

^{1}H NMR spectrum of **CPO-DBCO** in $CDCl_3$ recorded at 400 MHz and 25 ºC.

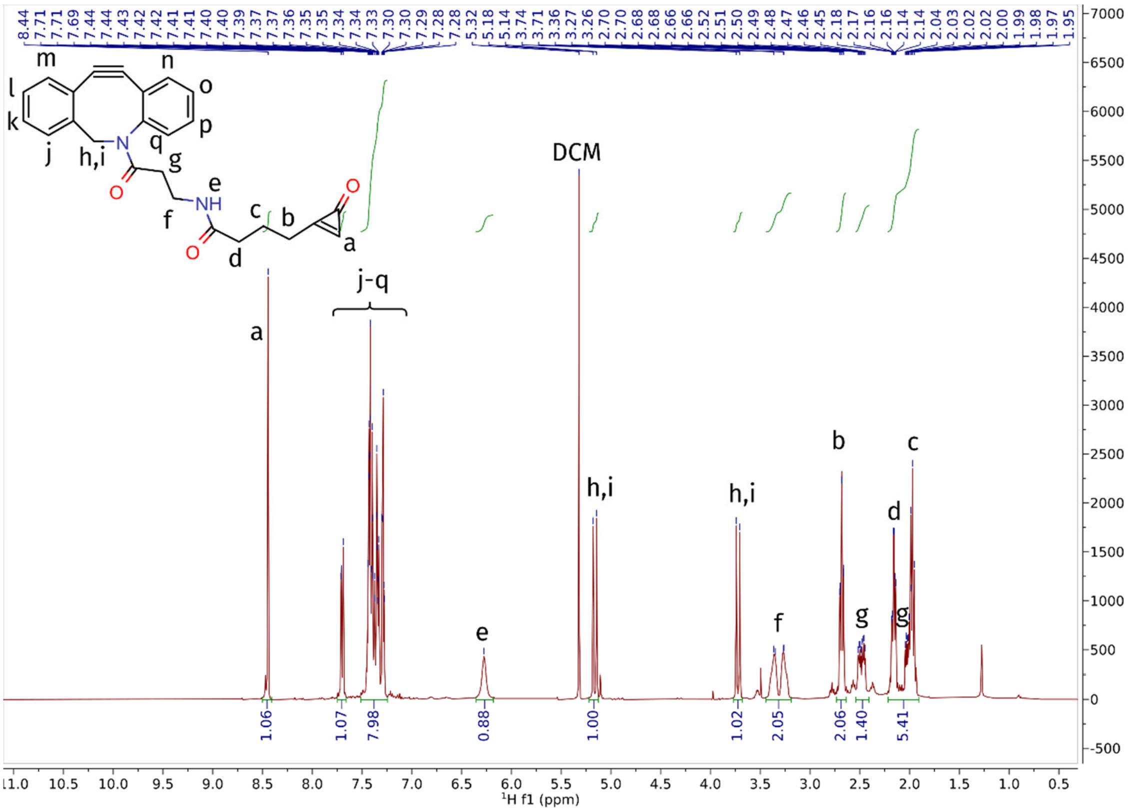

^{13}C NMR spectrum of **CPO-DBCO** in $CDCl_3$ recorded at 101 MHz and 25 ºC.

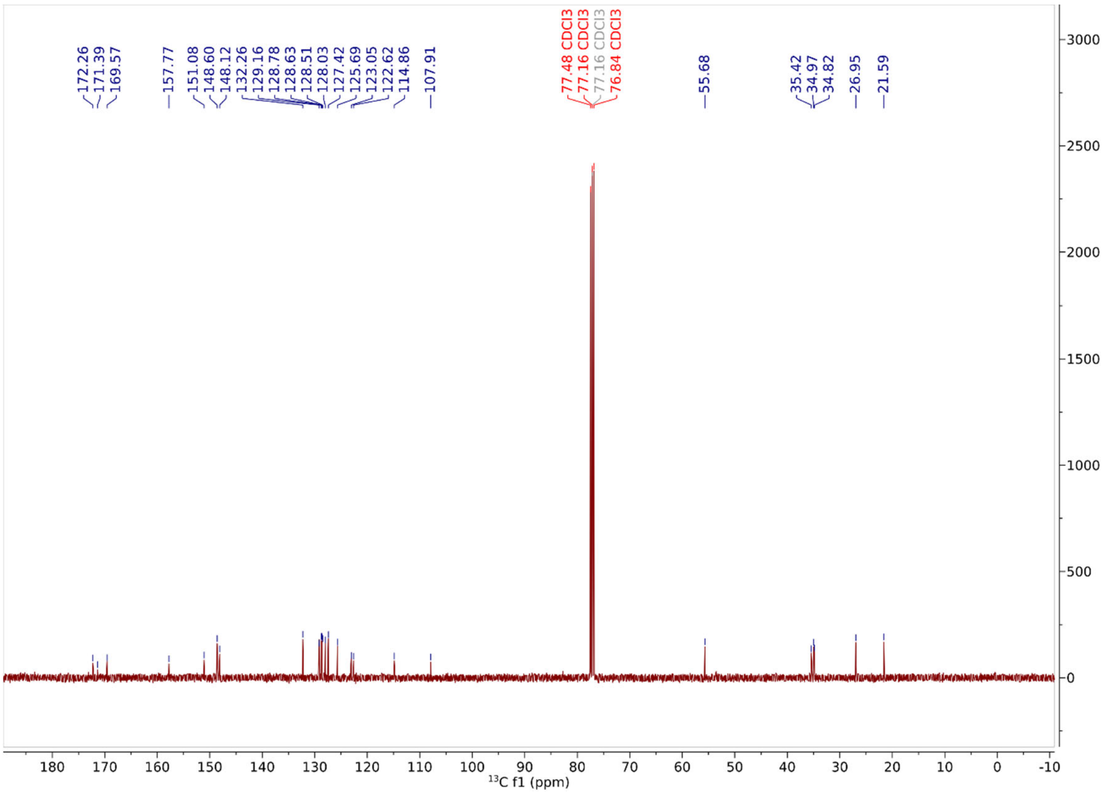

Appendix 1: Sequence and masses of antibody constructs

AntiCD33-(Wildtype)

LC:
DIQLTQSPSTLSASVGDRVTITCRASESLDNYGIRFLTWFQQKPGKAPKLLMYAASNQGSGVPSRFSGSGSGTEFTL
TISSLQPDDFATYYCQQTKEVPWSFGQGTKVEVKRTVAAPSVFIFPPSDEQLKSGTASVVCLLNNFYPREAKVQWK
VDNALQSGNSQESVTEQDSKDSTYSLSSTLTLSKADYEKHKVYACEVTHQGLSSPVTKSFNRGEC
Primary sequence calculated mass: 23828 Da
Intrachain cysteine oxidised calculated mass: 23824 Da

HC:
EVQLVQSGAEVKKPGSSVKVSCKASGYTITDSNIHWVRQAPGQSLEWIGYIYPYNGGTDYNQKFKNRATLTVDNP
TNTAYMELSSLRSEDTAFYYCVNGNPWLAYWGQGTLVTVSSASTKGPSVFPLAPSSKSTSGGTAALGCLVKDYFPE
PVTVSWNSGALTSGVHTFPAVLQSSGLYSLSSVVTVPSSSLGTQTYICNVNHKPSNTKVDKRVEPKSCDKTHTCPPC
PAPEFEGGPSVFLFPPKPKDTLMISRTPEVTCVVVDVSHEDPEVKFNWYVDGVEVHNAKTKPREEQYNSTYRVVSV
LTVLHQDWLNGKEYKCKVSNKALPASIEKTISKAKGQPREPQVYTLPPSREEMTKNQVSLTCLVKGFYPSDIAVEWE
SNGQPENNYKTTPPVLDSDGSFFLYSKLTVDKSRWQQGNVFSCSVMHEALHNHYTQKSLSLSPG
Primary sequence calculated mass: 48930 Da
Intrachain cysteine oxidised calculated mass: 48922 Da

AntiCD33-(TEV-Cys)

LC:
ENLYFQCDIQLTQSPSTLSASVGDRVTITCRASESLDNYGIRFLTWFQQKPGKAPKLLMYAASNQGSGVPSRFSGSG
SGTEFTLTISSLQPDDFATYYCQQTKEVPWSFGQGTKVEVKRTVAAPSVFIFPPSDEQLKSGTASVVCLLNNFYPREA
KVQWKVDNALQSGNSQESVTEQDSKDSTYSLSSTLTLSKADYEKHKVYACEVTHQGLSSPVTKSFNRGEC
Primary sequence calculated mass: 24727 Da
Intrachain cysteine oxidised calculated mass: 24723 Da

HC:
EVQLVQSGAEVKKPGSSVKVSCKASGYTITDSNIHWVRQAPGQSLEWIGYIYPYNGGTDYNQKFKNRATLTVDNP
TNTAYMELSSLRSEDTAFYYCVNGNPWLAYWGQGTLVTVSSASTKGPSVFPLAPSSKSTSGGTAALGCLVKDYFPE
PVTVSWNSGALTSGVHTFPAVLQSSGLYSLSSVVTVPSSSLGTQTYICNVNHKPSNTKVDKRVEPKSCDKTHTCPPC
PAPEFEGGPSVFLFPPKPKDTLMISRTPEVTCVVVDVSHEDPEVKFNWYVDGVEVHNAKTKPREEQYNSTYRVVSV
LTVLHQDWLNGKEYKCKVSNKALPASIEKTISKAKGQPREPQVYTLPPSREEMTKNQVSLTCLVKGFYPSDIAVEWE
SNGQPENNYKTTPPVLDSDGSFFLYSKLTVDKSRWQQGNVFSCSVMHEALHNHYTQKSLSLSPG
Primary sequence calculated mass: 48930 Da
Intrachain cysteine oxidised calculated mass: 48922 Da

AntiCD33-(FLAG-Cys)

LC:
DYKDDDDKCGGSGGDIQLTQSPSTLSASVGDRVTITCRASESLDNYGIRFLTWFQQKPGKAPKLLMYAASNQGSG
VPSRFSGSGSGTEFTLTISSLQPDDFATYYCQQTKEVPWSFGQGTKVEVKRTVAAPSVFIFPPSDEQLKSGTASVVCL
LNNFYPREAKVQWKVDNALQSGNSQESVTEQDSKDSTYSLSSTLTLSKADYEKHKVYACEVTHQGLSSPVTKSFNR
GEC
Primary sequence calculated mass: 25242 Da
Intrachain cysteine oxidised calculated mass: 25238 Da

HC:
EVQLVQSGAEVKKPGSSVKVSCKASGYTITDSNIHWVRQAPGQSLEWIGYIYPYNGGTDYNQKFKNRATLTVDNP
TNTAYMELSSLRSEDTAFYYCVNGNPWLAYWGQGTLVTVSSASTKGPSVFPLAPSSKSTSGGTAALGCLVKDYFPE
PVTVSWNSGALTSGVHTFPAVLQSSGLYSLSSVVTVPSSSLGTQTYICNVNHKPSNTKVDKRVEPKSCDKTHTCPPC
PAPEFEGGPSVFLFPPKPKDTLMISRTPEVTCVVVDVSHEDPEVKFNWYVDGVEVHNAKTKPREEQYNSTYRVVSV
LTVLHQDWLNGKEYKCKVSNKALPASIEKTISKAKGQPREPQVYTLPPSREEMTKNQVSLTCLVKGFYPSDIAVEWE
SNGQPENNYKTTPPVLDSDGSFFLYSKLTVDKSRWQQGNVFSCSVMHEALHNHYTQKSLSLSPG
Primary sequence calculated mass: 48930 Da
Intrachain cysteine oxidised calculated mass: 48922 Da

AntiCD33-(ST)

LC:
DIQLTQSPSTLSASVGDRVTITCRASESLDNYGIRFLTWFQQKPGKAPKLLMYAASNQGSGVPSRFSGSGSGTEFTL
TISSLQPDDFATYYCQQTKEVPWSFGQGTKVEVKRTVAAPSVFIFPPSDEQLKSGTASVVCLLNNFYPREAKVQWK
VDNALQSGNSQESVTEQDSKDSTYSLSSTLTLSKADYEKHKVYACEVTHQGLSSPVTKSFNRGECGGSGGLPETGG
HHHHHH
Primary sequence calculated mass: 25521 Da
Intrachain cysteine oxidised calculated mass: 25517 Da

HC:
EVQLVQSGAEVKKPGSSVKVSCKASGYTITDSNIHWVRQAPGQSLEWIGYIYPYNGGTDYNQKFKNRATLTVDNP
TNTAYMELSSLRSEDTAFYYCVNGNPWLAYWGQGTLVTVSSASTKGPSVFPLAPSSKSTSGGTAALGCLVKDYFPE
PVTVSWNSGALTSGVHTFPAVLQSSGLYSLSSVVTVPSSSLGTQTYICNVNHKPSNTKVDKRVEPKSCDKTHTCPPC
PAPEFEGGPSVFLFPPKPKDTLMISRTPEVTCVVVDVSHEDPEVKFNWYVDGVEVHNAKTKPREEQYNSTYRVVSV
LTVLHQDWLNGKEYKCKVSNKALPASIEKTISKAKGQPREPQVYTLPPSREEMTKNQVSLTCLVKGFYPSDIAVEWE
SNGQPENNYKTTPPVLDSDGSFFLYSKLTVDKSRWQQGNVFSCSVMHEALHNHYTQKSLSLSPG
Primary sequence calculated mass: 48930 Da
Intrachain cysteine oxidised calculated mass: 48922 Da

AntiCD33-(πClampNTerm-LC)

LC:
FCPFDIQLTQSPSTLSASVGDRVTITCRASESLDNYGIRFLTWFQQKPGKAPKLLMYAASNQGSGVPSRFSGSGSGT
EFTLTISSLQPDDFATYYCQQTKEVPWSFGQGTKVEVKRTVAAPSVFIFPPSDEQLKSGTASVVCLLNNFYPREAKV
QWKVDNALQSGNSQESVTEQDSKDSTYSLSSTLTLSKADYEKHKVYACEVTHQGLSSPVTKSFNRGEC
Primary sequence calculated mass: 24323 Da
Intrachain cysteine oxidised calculated mass: 24319 Da

HC:
EVQLVQSGAEVKKPGSSVKVSCKASGYTITDSNIHWVRQAPGQSLEWIGYIYPYNGGTDYNQKFKNRATLTVDNP
TNTAYMELSSLRSEDTAFYYCVNGNPWLAYWGQGTLVTVSSASTKGPSVFPLAPSSKSTSGGTAALGCLVKDYFPE
PVTVSWNSGALTSGVHTFPAVLQSSGLYSLSSVVTVPSSSLGTQTYICNVNHKPSNTKVDKRVEPKSCDKTHTCPPC
PAPEFEGGPSVFLFPPKPKDTLMISRTPEVTCVVVDVSHEDPEVKFNWYVDGVEVHNAKTKPREEQYNSTYRVVSV
LTVLHQDWLNGKEYKCKVSNKALPASIEKTISKAKGQPREPQVYTLPPSREEMTKNQVSLTCLVKGFYPSDIAVEWE
SNGQPENNYKTTPPVLDSDGSFFLYSKLTVDKSRWQQGNVFSCSVMHEALHNHYTQKSLSLSPG
Primary sequence calculated mass: 48930 Da
Intrachain cysteine oxidised calculated mass: 48922 Da

AntiCD33-(πClampCTerm-HC)

LC:
DIQLTQSPSTLSASVGDRVTITCRASESLDNYGIRFLTWFQQKPGKAPKLLMYAASNQGSGVPSRFSGSGSGTEFTL
TISSLQPDDFATYYCQQTKEVPWSFGQGTKVEVKRTVAAPSVFIFPPSDEQLKSGTASVVCLLNNFYPREAKVQWK
VDNALQSGNSQESVTEQDSKDSTYSLSSTLTLSKADYEKHKVYACEVTHQGLSSPVTKSFNRGEC
Primary sequence calculated mass: 23828 Da
Intrachain cysteine oxidised calculated mass: 23824 Da

HC:
EVQLVQSGAEVKKPGSSVKVSCKASGYTITDSNIHWVRQAPGQSLEWIGYIYPYNGGTDYNQKFKNRATLTVDNP
TNTAYMELSSLRSEDTAFYYCVNGNPWLAYWGQGTLVTVSSASTKGPSVFPLAPSSKSTSGGTAALGCLVKDYFPE
PVTVSWNSGALTSGVHTFPAVLQSSGLYSLSSVVTVPSSSLGTQTYICNVNHKPSNTKVDKRVEPKSCDKTHTCPPC
PAPEFEGGPSVFLFPPKPKDTLMISRTPEVTCVVVDVSHEDPEVKFNWYVDGVEVHNAKTKPREEQYNSTYRVVSV
LTVLHQDWLNGKEYKCKVSNKALPASIEKTISKAKGQPREPQVYTLPPSREEMTKNQVSLTCLVKGFYPSDIAVEWE
SNGQPENNYKTTPPVLDSDGSFFLYSKLTVDKSRWQQGNVFSCSVMHEALHNHYTQKSLSLSPGKGFCPF
Primary sequence calculated mass: 49611 Da
Intrachain cysteine oxidised calculated mass: 49603 Da

AntiCD33-(FLAG-Cys)-(239iC)

LC:
DYKDDDDKCGGSGGDIQLTQSPSTLSASVGDRVTITCRASESLDNYGIRFLTWFQQKPGKAPKLLMYAASNQGSG
VPSRFSGSGSGTEFTLTISSLQPDDFATYYCQQTKEVPWSFGQGTKVEVKRTVAAPSVFIFPPSDEQLKSGTASVVCL
LNNFYPREAKVQWKVDNALQSGNSQESVTEQDSKDSTYSLSSTLTLSKADYEKHKVYACEVTHQGLSSPVTKSFNR
GEC
Primary sequence calculated mass: 25242 Da
Intrachain cysteine oxidised calculated mass: 25238 Da

HC:
EVQLVQSGAEVKKPGSSVKVSCKASGYTITDSNIHWVRQAPGQSLEWIGYIYPYNGGTDYNQKFKNRATLTVDNP
TNTAYMELSSLRSEDTAFYYCVNGNPWLAYWGQGTLVTVSSASTKGPSVFPLAPSSKSTSGGTAALGCLVKDYFPE
PVTVSWNSGALTSGVHTFPAVLQSSGLYSLSSVVTVPSSSLGTQTYICNVNHKPSNTKVDKRVEPKSCDKTHTCPPC
PAPEFEGGPSCVFLFPPKPKDTLMISRTPEVTCVVVDVSHEDPEVKFNWYVDGVEVHNAKTKPREEQYNSTYRVVS
VLTVLHQDWLNGKEYKCKVSNKALPASIEKTISKAKGQPREPQVYTLPPSREEMTKNQVSLTCLVKGFYPSDIAVEW
ESNGQPENNYKTTPPVLDSDGSFFLYSKLTVDKSRWQQGNVFSCSVMHEALHNHYTQKSLSLSPG
Primary sequence calculated mass: 49034 Da
Intrachain cysteine oxidised calculated mass: 49026 Da

AntiCD33-(FLAG-Cys)-(T289C)

LC:
DYKDDDDKCGGSGGDIQLTQSPSTLSASVGDRVTITCRASESLDNYGIRFLTWFQQKPGKAPKLLMYAASNQGSG
VPSRFSGSGSGTEFTLTISSLQPDDFATYYCQQTKEVPWSFGQGTKVEVKRTVAAPSVFIFPPSDEQLKSGTASVVCL
LNNFYPREAKVQWKVDNALQSGNSQESVTEQDSKDSTYSLSSTLTLSKADYEKHKVYACEVTHQGLSSPVTKSFNR
GEC
Primary sequence calculated mass: 25242 Da
Intrachain cysteine oxidised calculated mass: 25238 Da

HC:

EVQLVQSGAEVKKPGSSVKVSCKASGYTITDSNIHWVRQAPGQSLEWIGYIYPYNGGTDYNQKFKNRATLTVDNP
TNTAYMELSSLRSEDTAFYYCVNGNPWLAYWGQGTLVTVSSASTKGPSVFPLAPSSKSTSGGTAALGCLVKDYFPE
PVTVSWNSGALTSGVHTFPAVLQSSGLYSLSSVVTVPSSSLGTQTYICNVNHKPSNTKVDKRVEPKSCDKTHTCPPC
PAPEFEGGPSVFLFPPKPKDTLMISRTPEVTCVVVDVSHEDPEVKFNWYVDGVEVHNAKCKPREEQYNSTYRVVSV
LTVLHQDWLNGKEYKCKVSNKALPASIEKTISKAKGQPREPQVYTLPPSREEMTKNQVSLTCLVKGFYPSDIAVEWE
SNGQPENNYKTTPPVLDSDGSFFLYSKLTVDKSRWQQGNVFSCSVMHEALHNHYTQKSLSLSPG
Primary sequence calculated mass: 48932 Da
Intrachain cysteine oxidised calculated mass: 48924 Da

AntiCD33-(FLAG-Cys)-(A327C)

LC:
DYKDDDDKCGGSGGDIQLTQSPSTLSASVGDRVTITCRASESLDNYGIRFLTWFQQKPGKAPKLLMYAASNQGSG
VPSRFSGSGSGTEFTLTISSLQPDDFATYYCQQTKEVPWSFGQGTKVEVKRTVAAPSVFIFPPSDEQLKSGTASVVCL
LNNFYPREAKVQWKVDNALQSGNSQESVTEQDSKDSTYSLSSTLTLSKADYEKHKVYACEVTHQGLSSPVTKSFNR
GEC
Primary sequence calculated mass: 25242 Da
Intrachain cysteine oxidised calculated mass: 25238 Da

HC:
EVQLVQSGAEVKKPGSSVKVSCKASGYTITDSNIHWVRQAPGQSLEWIGYIYPYNGGTDYNQKFKNRATLTVDNP
TNTAYMELSSLRSEDTAFYYCVNGNPWLAYWGQGTLVTVSSASTKGPSVFPLAPSSKSTSGGTAALGCLVKDYFPE
PVTVSWNSGALTSGVHTFPAVLQSSGLYSLSSVVTVPSSSLGTQTYICNVNHKPSNTKVDKRVEPKSCDKTHTCPPC
PAPEFEGGPSVFLFPPKPKDTLMISRTPEVTCVVVDVSHEDPEVKFNWYVDGVEVHNAKTKPREEQYNSTYRVVSV
LTVLHQDWLNGKEYKCKVSNKCLPASIEKTISKAKGQPREPQVYTLPPSREEMTKNQVSLTCLVKGFYPSDIAVEWE
SNGQPENNYKTTPPVLDSDGSFFLYSKLTVDKSRWQQGNVFSCSVMHEALHNHYTQKSLSLSPG
Primary sequence calculated mass: 48963 Da
Intrachain cysteine oxidised calculated mass: 48955 Da

AntiCD33-(ST)-(239iC)

LC:
DIQLTQSPSTLSASVGDRVTITCRASESLDNYGIRFLTWFQQKPGKAPKLLMYAASNQGSGVPSRFSGSGSGTEFTL
TISSLQPDDFATYYCQQTKEVPWSFGQGTKVEVKRTVAAPSVFIFPPSDEQLKSGTASVVCLLNNFYPREAKVQWK
VDNALQSGNSQESVTEQDSKDSTYSLSSTLTLSKADYEKHKVYACEVTHQGLSSPVTKSFNRGECGGSGGLPETGG
HHHHHH
Primary sequence calculated mass: 25521 Da
Intrachain cysteine oxidised calculated mass: 25517 Da

HC:
EVQLVQSGAEVKKPGSSVKVSCKASGYTITDSNIHWVRQAPGQSLEWIGYIYPYNGGTDYNQKFKNRATLTVDNP
TNTAYMELSSLRSEDTAFYYCVNGNPWLAYWGQGTLVTVSSASTKGPSVFPLAPSSKSTSGGTAALGCLVKDYFPE
PVTVSWNSGALTSGVHTFPAVLQSSGLYSLSSVVTVPSSSLGTQTYICNVNHKPSNTKVDKRVEPKSCDKTHTCPPC
PAPEFEGGPSCVFLFPPKPKDTLMISRTPEVTCVVVDVSHEDPEVKFNWYVDGVEVHNAKTKPREEQYNSTYRVVS
VLTVLHQDWLNGKEYKCKVSNKALPASIEKTISKAKGQPREPQVYTLPPSREEMTKNQVSLTCLVKGFYPSDIAVEW
ESNGQPENNYKTTPPVLDSDGSFFLYSKLTVDKSRWQQGNVFSCSVMHEALHNHYTQKSLSLSPG
Primary sequence calculated mass: 49034 Da
Intrachain cysteine oxidised calculated mass: 49026 Da

AntiCD33-(FLAG-Cys)-(ST)-(239iC)

LC:
DYKDDDDKCGGSGGDIQLTQSPSTLSASVGDRVTITCRASESLDNYGIRFLTWFQQKPGKAPKLLMYAASNQGSG
VPSRFSGSGSGTEFTLTISSLQPDDFATYYCQQTKEVPWSFGQGTKVEVKRTVAAPSVFIFPPSDEQLKSGTASVVCL
LNNFYPREAKVQWKVDNALQSGNSQESVTEQDSKDSTYSLSSTLTLSKADYEKHKVYACEVTHQGLSSPVTKSFNR
GECGGSGGLPETGGHHHHHH
Primary sequence calculated mass: 26935 Da
Intrachain cysteine oxidised calculated mass: 26931 Da

HC:
EVQLVQSGAEVKKPGSSVKVSCKASGYTITDSNIHWVRQAPGQSLEWIGYIYPYNGGTDYNQKFKNRATLTVDNP
TNTAYMELSSLRSEDTAFYYCVNGNPWLAYWGQGTLVTVSSASTKGPSVFPLAPSSKSTSGGTAALGCLVKDYFPE
PVTVSWNSGALTSGVHTFPAVLQSSGLYSLSSVVTVPSSSLGTQTYICNVNHKPSNTKVDKRVEPKSCDKTHTCPPC
PAPEFEGGPSCVFLFPPKPKDTLMISRTPEVTCVVVDVSHEDPEVKFNWYVDGVEVHNAKTKPREEQYNSTYRVVS
VLTVLHQDWLNGKEYKCKVSNKALPASIEKTISKAKGQPREPQVYTLPPSREEMTKNQVSLTCLVKGFYPSDIAVEW
ESNGQPENNYKTTPPVLDSDGSFFLYSKLTVDKSRWQQGNVFSCSVMHEALHNHYTQKSLSLSPG
Primary sequence calculated mass: 49034 Da
Intrachain cysteine oxidised calculated mass: 49026 Da

AntiCD33-(FLAG-Cys)-(ST)-(239iC)-(GD4)

LC:
DYKDDDDKCGGSGGDIQLTQSPSTLSASVGDRVTITCRASESLDNYGIRFLTWFQQKPGKAPKLLMYAASNQGSG
VPSRFSGSGSGTEFTLTISSLQPDDFATYYCQQTKEVPWSFGQGTKVEVKRTVAAPSVFIFPPSDEQLKSGTASVVCL
LNNFYPREAKVQWKVDNALQSGNSQESVTEQDSKDSTYSLSSTLTLSKADYEKHKVYACEVTHQGLSSPVTKSFNR
GECGGSGGLPETGGHHHHHH
Primary sequence calculated mass: 26935 Da
Intrachain cysteine oxidised calculated mass: 26931 Da

HC:
EVQLVQSGAEVKKPGSSVKVSCKASGYTITDSNIHWVRQAPGQSLEWIGYIYPYNGGTDYNQKFKNRATLTVDNP
TNTAYMELSSLRSEDTAFYYCVNGNPWLAYWGQGTLVTVSSASTKGPSVFPLAPSSKSTSGGTAALGCLVKDYFPE
PVTVSWNSGALTSGVHTFPAVLQSSGLYSLSSVVTVPSSSLGTQTYICNVNHKPSNTKVDKRVEPKSCDKTHTCPPC
PAPELLGGPSCVFLFPPKPKDTLMISRTPEVTCVVVDVSHEDPEVKFNWYVDGVEVHNAKTKPREEQYNSTYRVVS
VLTVLHQDWLNGKEYKCKVSNKALPAPIEKTISKAKGQPREPQVYTLPPSREEMTKNQVSLTCLVKGFYPSDIAVE
WESNGQPENNYKTTPPVLDSDGSFFLYSKLTVDKSRWQQGNVFSCSVMHEALHNHYTQKSLSLSPGGAPTAEPG
GAPTAEPGA
Primary sequence calculated mass: 50427 Da
Intrachain cysteine oxidised calculated mass: 50419 Da

AntiHER2-(GAL8)-(ST)

HC:
EVQLVESGGGLVQPGGSLRLSCAASGFNIKDTYIHWVRQAPGKGLEWVARIYPTNGYTRYADSVKGRFTISADTSK
NTAYLQMNSLRAEDTAVYYCSRWGGDGFYAMDYWGQGTLVTVSSASTKGPSVFPLAPSSKSTSGGTAALGCLVK
DYFPEPVTVSWNSGALTSGVHTFPAVLQSSGLYSLSSVVTVPSSSLGTQTYICNVNHKPSNTKVDKRVEPKSCDKTH
TCPPCPAPELLGGPSCVFLFPPKPKDTLMISRTPEVTCVVVDVSHEDPEVKFNWYVDGVEVHNAKTKPREEQYNST
YRVVSVLTVLHQDWLNGKEYKCKVSNKALPAPIEKTISKAKGQPREPQVYTLPPSREEMTKNQVSLTCLVKGFYPSD

IAVEWESNGQPENNYKTTPPVLDSDGSFFLYSKLTVDKSRWQQGNVFSCSVMHEALHNHYTQKSLSLSPGGAPTA
EPGGAPTAEPGGAPTAEPGGAPTAEPGA
Primary sequence calculated mass: 52081 Da
Intrachain cysteine oxidised calculated mass: 52073 Da

LC:
DIQMTQSPSSLSASVGDRVTITCRASQDVNTAVAWYQQKPGKVPKLLIYSASFLYSGVPSRFSGSRSGTDFTLTISSL
QPEDFATYYCQQHYTTPPTFGQGTKVEIKRRTVAAPSVFIFPPSDEQLKSGTASVVCLLNNFYPREAKVQWKVDNA
LQSGNSQESVTEQDSKDSTYSLSSTLTLSKADYEKHKVYACEVTHQGLSSPVTKSFNRGECGGSGGLPETGGHHHH
HH
Primary sequence calculated mass: 25320 Da
Intrachain cysteine oxidised calculated mass: 2525316 Da

AntiHER3-(GAL8)-(ST)

HC:
QVQLQQWGAGLLKPSETLSLTCAVYGGSFSGYYWSWIRQPPGKGLEWIGEINHSGSTNYNPSLKSRVTISVETSKN
QFSLKLSSVTAADTAVYYCARDKWTWYFDLWGRGTLVTVSSASTKGPSVFPLAPSSKSTSGGTAALGCLVKDYFPE
PVTVSWNSGALTSGVHTFPAVLQSSGLYSLSSVVTVPSSSLGTQTYICNVNHKPSNTKVDKRVEPKSCDKTHTCPPC
PAPELLGGPSCVFLFPPKPKDTLMISRTPEVTCVVVDVSHEDPEVKFNWYVDGVEVHNAKTKPREEQYNSTYRVVS
VLTVLHQDWLNGKEYKCKVSNKALPAPIEKTISKAKGQPREPQVYTLPPSREEMTKNQVSLTCLVKGFYPSDIAVE
WESNGQPENNYKTTPPVLDSDGSFFLYSKLTVDKSRWQQGNVFSCSVMHEALHNHYTQKSLSLSPGGAPTAEPG
GAPTAEPGGAPTAEPGGAPTAEPGA
Primary sequence calculated mass: 51983 Da
Intrachain cysteine oxidised + pyroglutamic acid calculated mass: 51958

LC:
DIEMTQSPDSLAVSLGERATINCRSSQSVLYSSSNRNYLAWYQQNPGQPPKLLIYWASTRESGVPDRFSGSGSGTD
FTLTISSLQAEDVAVYYCQQYYSTPRTFGQGTKVEIKRRTVAAPSVFIFPPSDEQLKSGTASVVCLLNNFYPREAKVQ
WKVDNALQSGNSQESVTEQDSKDSTYSLSSTLTLSKADYEKHKVYACEVTHQGLSSPVTKSFNRGECGGSGGLPET
GGHHHHHH
Primary sequence calculated mass: 26144 Da
Intrachain cysteine oxidised: 26140 Da

R347-(GAL8)-(ST)

HC:
EVQLLESGGGLVQPGGSLRLSCTTSGFTFNTYAMSWVRQAPGKGLEWLSGINNNGRTAFYADSVKGRFTISRDNS
KNTLYLQINSLRADDTAVYFCAKDVRFIAVPGDSWGQGTLVTVSSASTKGPSVFPLAPSSKSTSGGTAALGCLVKDY
FPEPVTVSWNSGALTSGVHTFPAVLQSSGLYSLSSVVTVPSSSLGTQTYICNVNHKPSNTKVDKRVEPKSCDKTHTC
PPCPAPELLGGPSCVFLFPPKPKDTLMISRTPEVTCVVVDVSHEDPEVKFNWYVDGVEVHNAKTKPREEQYNSTYR
VVSVLTVLHQDWLNGKEYKCKVSNKALPAPIEKTISKAKGQPREPQVYTLPPSREEMTKNQVSLTCLVKGFYPSDIA
VEWESNGQPENNYKTTPPVLDSDGSFFLYSKLTVDKSRWQQGNVFSCSVMHEALHNHYTQKSLSLSPGGAPTAE
PGGAPTAEPGGAPTAEPGGAPTAEPGA
Primary sequence calculated mass: 51881 Da
Intrachain cysteine oxidised + pyroglutamic acid calculated mass: 51873

LC:
ELVLTQPASVSGSPGQSITISCTGTSSDVGGYNYVSWYQQHPGKAPKLMIYDVSKRPSGVSNRFSGSKSGNTASLTI
SGLQAEDEADYYCSSYTSSSTLVFGGGTKLTVLGQPKAAPSVTLFPPSSEELQANKATLVCLISDFYPGAVTVAWKA

DSSPVKAGVETTTPSKQSNNKYAASSYLSLTPEQWKSHRSYSCQVTHEGSTVEKTVAPTECSGGSGGLPETGGHHH
HHH
Primary sequence calculated mass: 24441 Da
Intrachain cysteine oxidised + pyroglutamic acid calculated mass: 24437 Da

Appendix 2: UV-Vis spectra of fluorophore probes

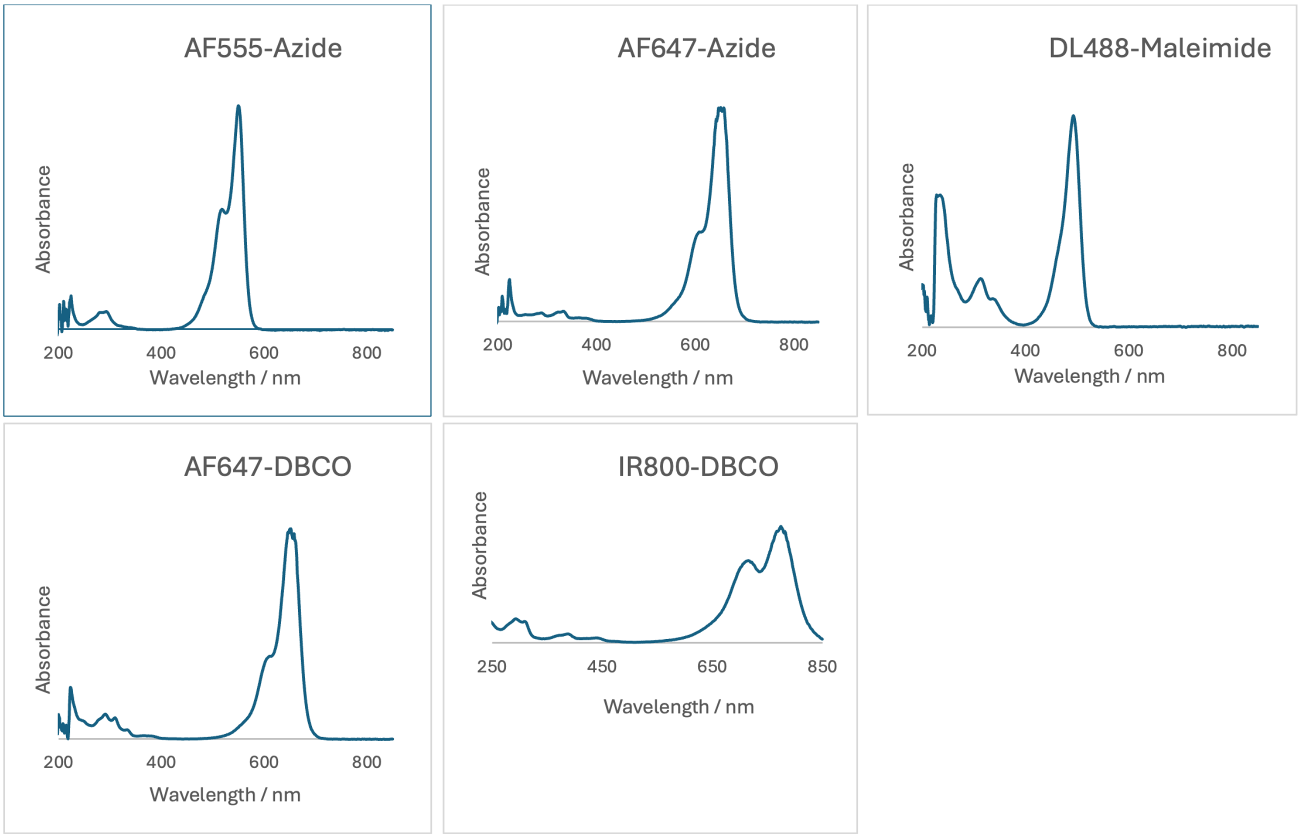

Supplement: Supplementary file 1 — Supporting Information [file ANIE-64-e202417620-s001.pdf]
